# Aldehyde–Olefin Couplings by Photoinduced Reduction of Electron‐Deficient Olefins with Hantzsch Ester Anions

**DOI:** 10.1002/anie.9744019

**Published:** 2026-05-04

**Authors:** Zhihang Li, Adam Noble

**Affiliations:** ^1^ School of Chemistry University of Bristol Bristol UK

**Keywords:** aldehyde–olefin couplings, olefin radical anions, photochemistry, photoreductant, radical chemistry

## Abstract

Reductive couplings between aldehydes and olefins, both of which are low‐cost chemical feedstocks, provide facile access to valuable alcohol products. Recent advances in photocatalytic and electrochemical methods have provided efficient strategies to achieve reductive aldehyde–olefin couplings through nucleophilic ketyl or olefin radical anion intermediates. However, the strongly reducing conditions required for radical formation result in limitations in substrate generality, especially for reactions of unactivated aliphatic aldehydes with electron‐deficient olefins. In this scenario, olefin dimerization or hydrogenation outcompetes aldehyde coupling due to the preferential formation and diminished nucleophilicity of olefin radical anion intermediates. Herein, we report a simple, photocatalyst‐free protocol that overcomes this limitation by using visible light‐activated Hantzsch ester as a photoreductant under mildly basic conditions. Key to the success of the transformation was the use of water as a protic additive, which enabled nucleophilic addition of the olefin radical anions to aldehydes. Mechanistic experiments support olefin radical anions as the key intermediates and offer insight into the important role of water in the transformation.

## Graphical abstract

Single‐electron transfer‐mediated reductive carbonyl–olefin couplings are valuable carbon–carbon bond forming reactions. However, limitations persist for couplings of aliphatic aldehydes with electron‐deficient olefins. Here, we report a simple, photocatalyst‐free protocol that overcomes these limitations by using a Hantzsch ester anion as a photoreductant for olefin radical anion generation, and water as a crucial protic additive for aldehyde activation.

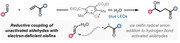

Radical‐mediated reductive carbonyl–olefin couplings offer an attractive approach to transform readily available feedstock aldehydes and ketones into diverse alcohol products (Scheme 1A) [1, 2, 3]. These carbon–carbon bond forming reactions proceed through simple single‐electron transfer (SET)‐induced substrate activation to generate nucleophilic radical intermediates, providing valuable umpolung reactivity that has been used extensively in complex molecule synthesis [4, 5, 6]. Mechanistically, two distinct pathways can operate depending on which substrate is activated via SET: (1) carbonyl reduction forms ketyl radical anions that undergo radical addition to olefins, whereas (2) olefin reduction yields olefin radical anions that react via polar nucleophilic addition to carbonyls (Scheme 1B) [7]. The two pathways converge at distonic radical anion **1**, before further reduction and protonation give the coupled product. Despite their mechanistic simplicity, these SET activation approaches are challenging due to the highly negative reduction potentials of carbonyls and olefins [8], often demanding the use of strong metal reductants, such as SmI_2_ [1, 9, 10, 11, 12], or electrochemistry at deeply reducing potentials [13, 14, 15].

**SCHEME 1:**
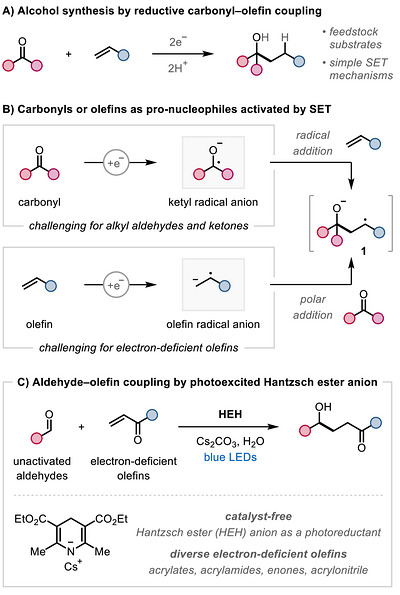
Reductive carbonyl–olefin couplings.

Developments in visible‐light photoredox catalysis have provided new avenues for carbonyl–olefin couplings [1]. These include using Bronsted acid activators to facilitate ketyl radical formation via proton‐coupled electron transfer (PCET) [16, 17, 18, 19]. However, these protocols are typically limited to activated carbonyls, including aromatic aldehydes and ketones, whereas aliphatic carbonyls are incompatible because of their more energetically demanding reduction. Nonetheless, recent reports of anionic or electron‐primed catalysts with highly reducing excited states have demonstrated successful ketyl radical formation from aliphatic ketones [7, 20, 21, 22, 23]. Conversely, reports of aliphatic aldehydes are rare and are either limited to intramolecular reactions or require high energy UV irradiation [7, 24, 25], likely due to their lower stability and greater susceptibility to side‐reactions compared to ketones. As a result, alternative strategies for aldehyde activation have been devised that circumvent the single‐electron reduction pathway, either through initial derivatization into redox‐active species that are more easily converted to ketyl radicals [26, 27, 28], or by using stoichiometric diboron reagents to access *O*‐boryl ketyl‐type radicals through boryl‐radical addition [29, 30].

For photocatalytic couplings of aliphatic aldehydes, the olefin radical anion pathway has proved more successful (Scheme 1B) [2]. This was demonstrated by the Polyzos group, who used an electron‐primed organic photoredox catalyst for the selective generation of olefin radical anions from 1,1‐diarylethylenes, which reacted with a range of aliphatic aldehydes [31]. However, extension to other classes of olefins, namely electron‐deficient examples, was not reported [31, 32, 33]. Very recently, Nicewicz and co‐workers applied a similar strategy to carbonyl–olefin couplings with acrylamides, although acrylates were shown to be ineffective [34]. This limitation of the olefin radical anion pathway for couplings of more electron‐deficient olefins results from the reduced nucleophilicity of the olefin radical anions upon incorporation of electron‐withdrawing groups, which prevents polar addition to the aldehyde [34]. As a result, no general photochemical methods have been reported for intermolecular reactions of aliphatic aldehydes with olefinic esters, ketones, or nitriles. Herein, we report the development of a simple, catalyst‐free photochemical reductive coupling that overcomes these limitations through the use of Hantzsch ester (**HEH**) as an inexpensive photoreductant for olefin radical anion formation, and water as a crucial protic additive for aldehyde activation (Scheme 1C) [35]. Whilst **HEH** is commonly used as a reductive quencher in photoredox‐catalysed carbonyl–olefin couplings via aromatic ketyl radicals [16, 17, 18, 19, 36, 37], we demonstrate that under mildly basic conditions, it functions as a powerful excited state reductant to enable couplings of aliphatic aldehydes with a broad range of electron‐deficient olefins via olefin radical anion intermediates.

We began our studies by investigating the coupling of 3‐phenylpropanal (**2**) and *t*‐butyl acrylate (**3**), using Cs_2_CO_3_ to activate **HEH** though deprotonation (Table 1) [38]. Although no alcohol product **4** was observed after irradiation with blue LEDs for 16 h, we identified di‐*tert*‐butyl adipate (**5**) in a 42% yield (entry 1), which was formed by reductive homo‐coupling of **3** [39, 40, 41]. This suggests that acrylate **3** was successfully reduced to a radical anion, but subsequent homo‐coupling was favored over cross‐coupling with aldehyde **2**. This is in line with previous photocatalytic carbonyl–olefin couplings with acrylates, wherein the low nucleophilicity of the olefin radical anion prevents addition to the carbonyl [34]. We hypothesized that protic additives could facilitate cross‐coupling of the radical anion of olefin **3** with aldehyde **2** through carbonyl activation [20, 25]. To our delight, alcohol **4** was obtained in 56% yield when the reaction was performed in the presence of 50 equivalents of water (entry 2). Conversely, other protic additives, including MeOH and trifluoroethanol (TFE), failed to promote the cross‐coupling, despite successful olefin radical anion generation, as confirmed by the formation of olefin dimerization and hydrogenation products **5** and **6** (entries 3 and 4). Changing the base to 1,1,3,3‐tetramethylguanidine (TMG) gave a lower yield of **4**, whereas KOH inhibited the reaction (entries 5 and 6). The coupling was also solvent‐dependent, with no product observed when DMF was replaced with MeCN (entry 7 and Table S2). Finally, control experiments demonstrated that **HEH**, light, and Cs_2_CO_3_ were all essential for the transformation (entries 8‐10) [42].

**TABLE 1:** Optimization studies.^[^ ^a^ ^].^

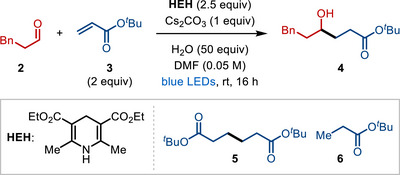			

Entry	Variation from above	4 (%)^b^	5 (%)^b^
1	Without H_2_O	0	42
2	None	56	38
3	MeOH as protic additive	0	10^c^
4	TFE as protic additive	0	0^c^
5	TMG as base	40	15
6	KOH as base	0	32
7	MeCN as solvent	0	0
8	Without **HEH**	0	0
9	Without light	0	0
10	Without Cs_2_CO_3_	0	0

^a^ Reactions performed using 0.1 mmol of **2**.

^b^ Yields (relative to 0.1 mmol) were determined by ^1^H NMR analysis using an internal standard.

^c^ **6** was formed in 22% (entry 3) and 82% (entry 4).

With the optimized conditions in hand, we proceeded to investigate the scope of aldehydes in the coupling with acrylate **3** (Scheme 2). In addition to simple unfunctionalized aliphatic aldehydes (**4**, **7**), primary alkyl aldehydes bearing halides (**8**‐**9**), tetrahydropyrans (**10**), Boc‐protected piperidines (**11**), benzyl ethers (**12**), alkynes (**13**) and alkenes (**14**) gave moderate to good yields. Of note, our strategy is selective for aldehydes over ketones, as demonstrated by lithocholic acid derivative **15**, wherein the ketone group was untouched in the transformation. In addition, the method enabled the synthesis of the tertiary amine‐containing chloroambucil derivative **16**. Secondary alkyl aldehydes were also tolerated, including cyclic (**17**‐**22**) and acyclic (**23**) substrates. Notably, sterically hindered *N*‐Boc‐prolinal (**22**) was successfully coupled with **3**, albeit in low yield. Unfortunately, very low efficiencies were observed for tertiary alkyl aldehydes, such as pivaldehyde (**24**). Finally, aromatic aldehydes (**25**) could also be used, demonstrating the effectiveness of this simple protocol across both activated and unactivated substrates.

**SCHEME 2:**
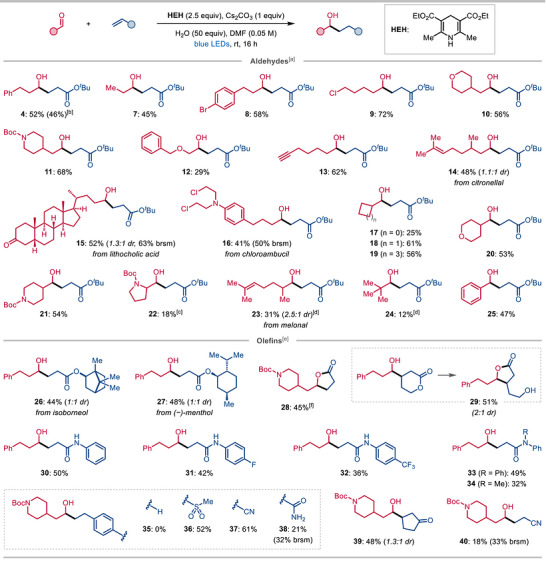
Aldehyde–olefin coupling scope. Reactions performed using aldehyde (0.2 mmol), olefin (2.0‐3.0 equiv), **HEH** (2.5 equiv), Cs_2_CO_3_ (1.0 equiv), and H_2_O (50 equiv) in DMF (0.05 M). Yields are of isolated products after purification. Diastereomeric ratio (dr) determined after purification. brsm = based on recovered starting material. ^[a]^Using olefin (3.0 equiv). ^[b]^Reaction performed on 2 mmol scale. ^[c]^Only the major diastereomer was isolated. ^[d]^Yield was determined by ^1^H NMR analysis using dibromomethane as the internal standard. ^[e]^Using olefin (2.0 equiv). ^[f]^Using ethyl acrylate.

Next, we explored different olefin coupling partners (Scheme 2). Various acrylates were successfully coupled, including those derived from natural products isoborneol (**26**) and menthol (**27**). For acrylates with sterically unhindered *O*‐substituents, lactonization of the initially formed γ‐hydroxy esters occurred under the basic reaction conditions, including ethyl acrylate (**28**) and dihydropyranone, which underwent translactonization to form 5‐membered lactone **29**. These results demonstrate the synthetic utility of the γ‐hydroxy ester products for the formation of medicinally relevant lactones [43]. The successful formation of **29** also demonstrates the applicability of *β*‐substituted acrylates. Pleasingly, acrylamides were also compatible, including secondary (**30**‐**32**) and tertiary (**33**‐**34**) examples [34]. Although unfunctionalized styrene (**35**) failed to react, derivatives bearing electron‐withdrawing groups, such as sulfone (**36**), cyanide (**37**) and amide (**38**), were coupled in moderate to good yields. This reactivity trend is complementary to previously reported methods that are only applicable to electron‐neutral and electron‐rich styrenes [15, 20, 25, 31, 32]. Remarkably, cyclopentenone yielded *γ*‐hydroxy ketone **39** in moderate yield, which represents a rare example of an intermolecular carbonyl–enone cross‐coupling [4, 5, 6]. Finally, acrylonitrile also reacted successfully, although alcohol **40** was formed in a low yield of 18%, accompanied by 45% recovered aldehyde and 32% of the homo‐coupling product adiponitrile, which we attribute to a slower rate of addition of the less nucleophilic acrylonitrile radical anion to the aldehyde.

We then conducted mechanistic studies to determine whether ketyl or olefin radical anions were the key intermediates in our reaction. Whilst the observation of adipate **5** confirms that olefin radical anions are formed (see Table 1), this does not exclude the involvement of ketyl radicals in the aldehyde–olefin coupling. To investigate the formation of ketyl radicals, a radical clock experiment was performed using 2,2‐dimethyl‐cyclopropyl‐carbaldehyde (**41**), which is known to undergo rapid ring‐opening upon ketyl radical anion generation (Scheme 3A) [44]. This gave alcohol **42** with the cyclopropane ring intact, and no ring‐opened products were observed, suggesting that ketyl radicals are not formed and the olefin radical anion pathway dominates. In contrast, the reaction of aldehyde **41** with styrene (**43**) gave only ring‐opened products **44** and **45**, which confirms that ketyl radical pathways can occur when olefin reduction is disfavored due to lower reduction potentials (for comparison, *E* [**3**/**3** ^•−^] = −2.3 V and *E* [**43**/**43** ^•−^] = −2.6 V vs. SCE in DMF) [7]. However, the low yields of **44** and **45** imply that the ketyl radical pathway is inefficient in comparison to the olefin radical anion pathway.

**SCHEME 3:**
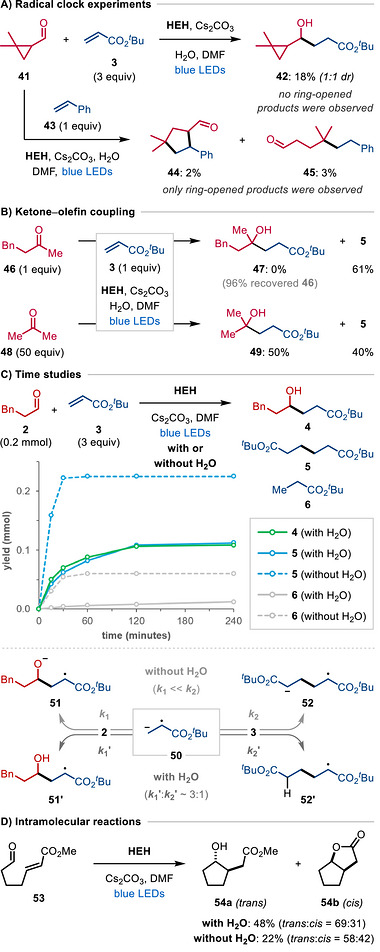
Mechanistic studies.

Further support for the olefin radical anion pathway was provided by the failure of ketones to couple with acrylate **3** (Scheme 3B). When ketone **46** was subjected to the optimized coupling conditions, alcohol **47** was not observed, whereas adipate **5** was formed in 61% yield. This can be explained by the increased steric hindrance and lower electrophilicity of ketones relative to aldehydes, which reduces the rate of reaction with the olefin radical anion to such an extent that it cannot compete with olefin homo‐coupling. To counteract this, we attempted the reaction of **3** with a large excess of acetone (**48**) [33], which led to successful ketone–olefin coupling, with tertiary alcohol **49** formed in 50% yield.

To gain insight into the role of water in the aldehyde–olefin coupling, we measured its effect on the rate of product formation for the reaction of aldehyde **2** with acrylate **3** (Scheme 3C). In the absence of water, alcohol **4** was not formed, but **3** was rapidly transformed to a mixture of adipate **5** and propionate **6**, with full conversion reached after approximately 30 min. This suggests the rate of addition of olefin radical anion **50** to aldehyde **2** to form distonic radical anion **51** (*k*_1_) is negligible in comparison to its addition to **3** to form olefin dimer radical anion **52** (*k*_2_). In contrast, with added water, alcohol **4** was generated at a comparable rate to adipate **5**. Given that 3 equivalents of olefin **3** were used, this means that the rate of formation of **4** from **50** is three times that of **5**. Thus, assuming similar rates for the conversion of *α*‐ester radicals **51ʹ** and **52ʹ** to **4** and **5**, the relative rates of addition of olefin radical anion **50** to aldehyde **2** (*k*_1_ʹ) and olefin **3** (*k*_2_ʹ) are approximately 3:1. We postulate that this dramatic rate acceleration for the formation of **4** in the presence of water could be caused by hydrogen‐bonding activation of the aldehyde [45], which enhances its electrophilicity to facilitate nucleophilic addition of olefin radical anion **50**. Alternatively, water could simply act as an acid to protonate distonic radical anion **51** and prevent fragmentation back to **2** and **50** [46]; however, this is unlikely given the aldehyde–olefin couplings failed when using alternative protic additives with similar p*K*_a_ values to water but lower hydrogen bond donor strengths (see Table S3) [47]. Water was also found to significantly improve the yield and enhance the *trans* diastereoselectivity of the intramolecular reaction of aldehyde **53** (Scheme 3D). This could be caused by hydrogen‐bonding activation of the aldehyde, which promotes addition of the olefin radical anion, whilst also increasing its steric influence during cyclization to disfavor the reactive conformer leading to *cis* diastereomer **54b** [24, 48].

Based on the above, we propose the mechanism shown in Scheme 4. Proton transfer (PT) between **HEH** (p*K*_a_ ∼ 20 in DMSO) [49] and Cs_2_CO_3_ (p*K*_a_ ∼ 21 for HCO_3_^−^ in DMSO) [50] generates the **HEH** anion (**HEH** ^−^), as confirmed by the appearance of a new absorbance feature at 471 nm. Photoexcitation of **HEH** ^−^ generates highly reducing excited state *HEH ^−^ (*E*_1/2_ [**HEH** ^•^/*HEH^−^] = −2.8 V vs. SCE in DMF), which undergoes selective SET with the more easily reduced olefin **55** over aldehyde **56** (*E*_p/2_ [**3**/**3** ^•−^] = −2.3 V; *E*_p/2_ [**2**/**2** ^•−^] = −2.5 V vs. SCE in DMF) to give olefin radical anion **57** and the **HEH** radical (**HEH** ^•^). Alternative activation modes based on electron donor–acceptor complexes were ruled out based on absorption spectroscopy studies (see Figure S8) [35]. Under anhydrous conditions, the low nucleophilicity of olefin radical anions derived from electron‐deficient olefins hinders polar addition of **57** to aldehyde **56**, preventing formation of distonic radical anion **58**. This leads to preferential reaction of **57** with olefin **55** to form olefin dimer radical anion **59**. However, addition of water results in activation of the aldehyde through hydrogen‐bonding, which promotes nucleophilic addition of **57** to generate *γ*‐hydroxy radical **60**. Given the relatively high acidity of *α*‐ester radicals **61** (p*K*_a_ ∼ 6) [51], competitive protonation of **57** is disfavored under our basic reaction conditions, which minimizes the formation of hydrogenation side products (e.g., **6**) [52]. Formation of products **62** and **64** from *α*‐ester radicals **60** and **63** occurs through reaction with **HEH** ^•^, either by hydrogen atom transfer (HAT) or SET/PT. Deuterium labelling studies using **HEH**‐*d*_2_ revealed only moderate levels of deuterium incorporation, which suggests that both HAT and SET/PT pathways occur. Finally, alternative radical chain mechanisms are unlikely given the low quantum yields for the reactions (Φ = 16%).

**SCHEME 4:**
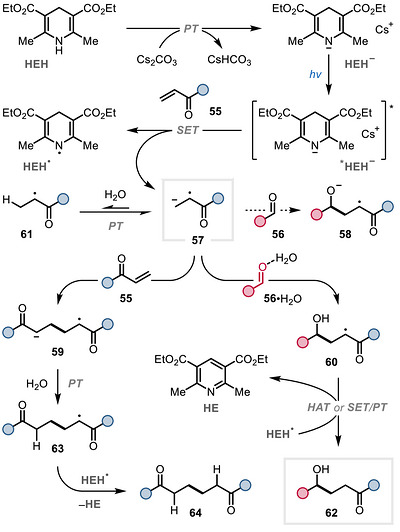
Proposed mechanism.

In conclusion, we have developed a novel method for intermolecular reductive aldehyde–olefin couplings, proceeding through single‐electron reduction of olefins by photoexcited Hantzsch ester anions. Our strategy uses inexpensive reagents, proceeds under mild, photocatalyst‐free conditions, and exhibits high functional group tolerance. This enabled aliphatic aldehydes to be coupled with a broad range of electron‐deficient olefins, including acrylates, acrylamides, styrenes, enones, and acrylonitrile. Crucial to the success of the transformation was the use of water as a protic additive to activate the aldehyde towards nucleophilic addition of olefin radical anions, thus overcoming a fundamental challenge for intermolecular aldehyde–olefin couplings of electron‐deficient olefins.

## Author Contributions

**Zhihang Li**: writing – original draft, investigation, formal analysis. **Adam Noble**: writing – review and editing, supervision, formal analysis, funding acquisition.

## Conflicts of Interest

The authors declare no conflict of interest.

## Supporting information




**Supporting File**: anie72498‐sup‐0001‐SuppMat.pdf.

## Data Availability

The data that supports the findings of this study are available in the supplementary material of this article
