# Supplementary material for: Aldehyde–Olefin Couplings by Photoinduced Reduction of Electron‐Deficient Olefins with Hantzsch Ester Anions
Source: Angew Chem Int Ed Engl. 2026 May 4;65(25):e9744019. doi: 10.1002/anie.9744019 (PMC13266924; doi:10.1002/anie.9744019)
Supplement: Supplementary file 1 — Supporting File: anie72498‐sup‐0001‐SuppMat.pdf. [file ANIE-65-e9744019-s001.pdf]

*SUPPORTING INFORMATION*

# Aldehyde–Olefin Couplings by Photoinduced Reduction of Electron-Deficient Olefins with Hantzsch Ester Anions

Zhihang Li and Adam Noble*

*School of Chemistry, University of Bristol, Cantock's Close, Bristol, BS8 1TS, United Kingdom*

*e-mail: a.noble@bristol.ac.uk

# TABLE OF CONTENTS


LIST OF SUPPLEMENTARY FIGURES AND TABLES ........ 3

LIST OF CHARACTERISED PRODUCTS ........ 3

1. MATERIALS AND GENERAL METHODS ........ 5

1.1. Glassware, Solvents and Reagents ........ 5

1.2. Chromatography and Instrumentation ........ 5

1.3. Naming of Compounds ........ 6

1.4. Photochemical Equipment and Setup ........ 6

2. EXPERIMENTAL DATA ........ 7

2.1. General Procedures ........ 7

2.1.1. General Procedure A: Aldehyde Scope ........ 7

2.1.2. General Procedure B: Olefin Scope ........ 7

2.1.3. General Workup and Analysis Procedure ........ 7

2.2. Reaction Optimization ........ 8

2.3. Synthesis of Starting Materials and Reagents ........ 14

2.4. Substrate Scope ........ 15

2.4.1. Aldehyde scope ........ 15

2.4.2. Olefin scope ........ 26

2.4.3. Unsuccessful and low yielding substrates ........ 34

2.4.4. Scale-up reaction ........ 36

2.4.5. Hydrodimerization of *tert*-butyl acrylate ........ 37

3. MECHANISTIC STUDIES ........ 38

3.1. Control Experiments ........ 38

3.1.1. Aldehyde experiments ........ 38

3.1.2. Olefin experiments ........ 38

3.1.3. Other reaction component experiments ........ 39

3.2. Radical Clock Experiments ........ 40

3.2.1. Experiment using *tert*-butyl acrylate ........ 40

3.2.2. Experiment using styrene ........ 41

3.3. Ketone Experiments ........ 42

3.3.1. Experiment using 1 equivalent of ketone ........ 42

3.3.2. Experiment using 50 equivalents of ketone ........ 42

3.4. Time Studies ........ 43

3.4.1. Aldehyde–olefin coupling ........ 43

3.4.2. Olefin hydrodimerization ........ 44

3.4.3. Comparison of the rate of product formation with and without water ........ 45

3.5. Intramolecular reaction ........ 46

3.6. Deuterium Labelling Experiments ........ 48

3.6.1. $D_2$-HEH as deuterium source ........................................48
3.6.2. Assignment of $^{13}C$ signals of alcohol product ........................................52
3.7. Visible-Light Absorption Spectroscopy ........................................54
3.8. Cyclic Voltammetry ........................................56
3.9. Estimation of the Excited-State Oxidation Potential of HEH Anion ........................................58
3.10. Quantum Yield Measurement ........................................59
4. SPECTROSCOPIC DATA ........................................ 62
5. REFERENCES ........................................ 103

# LIST OF SUPPLEMENTARY FIGURES AND TABLES

Figure S1. Photochemical reaction setup (0.2 mmol scale) ... 6
Table S1. Base screening ... 8
Table S2. Solvent screening ... 9
Table S3. Protic additive screening ... 10
Table S4. HEH analogues screening ... 11
Table S5. Light source screening ... 12
Table S6. Evaluation of aldehyde and olefin stoichiometries ... 12
Table S7. Evaluation of water volume ... 13
Table S8. Evaluation of base equivalents ... 13
Table S9. Evaluation of HEH equivalents ... 13
Figure S2. Photochemical reaction setup (2 mmol scale) ... 36
Table S10. Other reaction component experiments ... 39
Table S11. Time study of the aldehyde–olefin coupling reaction ... 43
Figure S3. Time study of aldehyde–olefin coupling reaction ... 43
Table S12. Time study of acrylate dimerization *with* $H_2O$ ... 44
Table S13. Time study of acrylate dimerization *without* $H_2O$ ... 44
Figure S4. Time study of total product formation with and without water ... 45
Figure S5. Crude $^1H$ NMR spectrum of the intramolecular aldehyde–olefin coupling with $H_2O$. ... 46
Figure S6. Crude $^1H$ NMR spectrum of the intramolecular aldehyde–olefin coupling without $H_2O$. ... 47
Figure S7. Proposed stereochemical model for intramolecular aldehyde–olefin coupling. ... 47
Figure S8. Visible-light absorption spectra of HEH (with reagents) ... 54
Figure S9. Visible-light absorption spectra of HEH (effect of water) ... 54
Figure S10. Cyclic voltammogram of acrylate **3** in anhydrous DMF (vs. $Ag/AgNO_3$) ... 56
Figure S11. Cyclic voltammogram of aldehyde **2** in anhydrous DMF (vs. $Ag/AgNO_3$) ... 57
Figure S12. Cyclic voltammogram of HEH and HEH/$Cs_2CO_3$ (1 equiv) in anhydrous DMF (vs. $Ag/AgNO_3$) ... 57
Figure S13. Normalized absorption and emission spectra of $HEH^-$ ... 58
Figure S14. UV-vis absorption spectra of ferrioxalate/1,10-phenanthroline solutions (after irradiation with Kessil Tuna Blue light for 0–30 s). ... 59
Figure S15. Moles of $Fe^{2+}$ formed vs. irradiation time ... 60

# LIST OF CHARACTERISED PRODUCTS

*tert*-Butyl 4-hydroxy-6-phenylhexanoate (**4**) ... 15
*tert*-Butyl 4-hydroxyhexanoate (**7**) ... 15
*tert*-Butyl 6-(4-bromophenyl)-4-hydroxyhexanoate (**8**) ... 16
*tert*-Butyl 8-chloro-4-hydroxyoctanoate (**9**) ... 16
*tert*-Butyl 4-hydroxy-5-(tetrahydro-2*H*-pyran-4-yl)pentanoate (**10**) ... 17
*tert*-Butyl 4-(5-(*tert*-butoxy)-2-hydroxy-5-oxopentyl)piperidine-1-carboxylate (**11**) ... 17
*tert*-Butyl 5-(benzyloxy)-4-hydroxypentanoate (**12**) ... 18
*tert*-Butyl 4-hydroxydec-9-ynoate (**13**) ... 18
*tert*-Butyl 4-hydroxy-6,10-dimethylundec-9-enoate (**14**) ... 19
*tert*-Butyl(7*R*)-7-((5*R*,8*R*,9*S*,10*S*,13*R*,14*S*,17*R*)-10,13-dimethyl-3-oxohexadecahydro-1*H*-cyclopenta[a]phenanthren-17-yl)-4-hydroxyoctanoate (**15**) ... 19

*tert*-Butyl 7-(4-(bis(2-chloroethyl)amino)phenyl)-4-hydroxyheptanoate (**16**) ..... 20
*tert*-Butyl 4-cyclopropyl-4-hydroxybutanoate (**17**) ..... 21
*tert*-Butyl 4-cyclobutyl-4-hydroxybutanoate (**18**) ..... 21
*tert*-Butyl 4-cyclohexyl-4-hydroxybutanoate (**19**) ..... 22
*tert*-Butyl 4-hydroxy-4-(tetrahydro-2*H*-pyran-4-yl)butanoate (**20**) ..... 22
*tert*-Butyl 4-(4-(*tert*-butoxy)-1-hydroxy-4-oxobutyl)piperidine-1-carboxylate (**21**) ..... 23
*tert*-Butyl 2-(4-(tert-butoxy)-1-hydroxy-4-oxobutyl)pyrrolidine-1-carboxylate (**22**) ..... 23
*tert*-Butyl 4-hydroxy-5,9-dimethyldec-8-enoate (**23**) ..... 24
*tert*-Butyl 4-hydroxy-5,5-dimethylhexanoate (**24**) ..... 24
*tert*-Butyl 4-hydroxy-4-phenylbutanoate (**25**) ..... 25
1,7,7-Trimethylbicyclo[2.2.1]heptan-2-yl 4-hydroxy-6-phenylhexanoate (**26**) ..... 26
(1*R*,2*S*,5*R*)-2-Isopropyl-5-methylcyclohexyl 4-hydroxy-6-phenylhexanoate (**27**) ..... 26
*tert*-Butyl 4-((5-oxotetrahydrofuran-2-yl)methyl)piperidine-1-carboxylate (**28**) ..... 27
4-(2-Hydroxyethyl)-5-phenethyldihydrofuran-2(3*H*)-one (**29**) ..... 27
4-Hydroxy-*N*,6-diphenylhexanamide (**30**) ..... 28
*N*-(4-Fluorophenyl)-4-hydroxy-6-phenylhexanamide (**31**) ..... 28
4-Hydroxy-6-phenyl-*N*-(4-(trifluoromethyl)phenyl)hexanamide (**32**) ..... 29
4-Hydroxy-*N*,*N*,6-triphenylhexanamide (**33**) ..... 30
4-Hydroxy-*N*-methyl-*N*,6-diphenylhexanamide (**34**) ..... 30
*tert*-Butyl 4-(2-hydroxy-4-(4-(methylsulfonyl)phenyl)butyl)piperidine-1-carboxylate (**36**) ..... 31
*tert*-Butyl 4-(4-(4-cyanophenyl)-2-hydroxybutyl)piperidine-1-carboxylate (**37**) ..... 31
*tert*-Butyl 4-(4-(4-carbamoylphenyl)-2-hydroxybutyl)piperidine-1-carboxylate (**38**) ..... 32
*tert*-Butyl 4-(2-hydroxy-2-(3-oxocyclopentyl)ethyl)piperidine-1-carboxylate (**39**) ..... 32
*tert*-Butyl 4-(4-cyano-2-hydroxybutyl)piperidine-1-carboxylate (**40**) ..... 33

# 1. MATERIALS AND GENERAL METHODS

## 1.1. Glassware, Solvents and Reagents

Anhydrous solvents were commercially supplied or dried using an Anhydrous Engineering alumina column drying system (THF, $Et_2O$, DCM, DMF) and stored over 4 Å molecular sieves. All reagents were purchased from commercial sources [Fluorochem Ltd, Sigma Aldrich (Merck), Fischer, TCI, etc.] and were used as received. Water (HPLC grade), MeCN, THF [Sigma Aldrich (Merck)] and DMF (Fischer) were used for the photochemical reactions.

Photochemical reactions were performed in glass vials (7 mL) sealed with B10 Suba-Seals.

## 1.2. Chromatography and Instrumentation

**Thin layer chromatography** (TLC) was performed using Merck Kieselgel 60 F254 fluorescent treated silica, which was visualised under UV light, or by staining with aqueous basic potassium permanganate followed by heating.

**Flash column chromatography** (FCC) was carried out using Sigma-Aldrich silica gel (60 Å, 230–400 mesh, 40–63 µm), eluting with combinations of ethyl acetate (EA)/petroleum ether (PE) or methanol (MeOH)/dichloromethane (DCM), as stated.

**Preparative Thin layer chromatography** (PLC) was performed using Merck Z513032-1PAK silica gel TLC plates, which were visualised under UV light.

**Normal-phase preparative high performance liquid chromatography (prep HPLC)** was performed on a Teledyne ACCQPrep HP150 system with a Phenomenex Luna silica column (5 µm, 100 Å, 250 x 21.2 mm) and hexane/ethyl acetate mobile phase, observing by an Evaporative Light-Scattering Detector (ELSD).

**NMR spectra** were recorded at various field strengths, as indicated, using Bruker 400 MHz, Varian VNMR 400 MHz, Varian VNMR 500 MHz for $^{1}H$, $^{13}C$ and $^{19}F$ acquisitions. All NMR spectra were recorded at 25 °C unless otherwise stated. Chemical shifts (δ) are reported in parts per million (ppm) and referenced to $CDCl_3$ ($^{1}H$: 7.26 ppm; $^{13}C$: 77.0 ppm) or $CD_3CN$ ($^{1}H$: 1.94 ppm; $^{13}C$: 1.32 ppm). Coupling constants (*J*) are given in Hertz (Hz) and refer to apparent multiplicities (s = singlet, d = doublet, t = triplet, q = quartet, quin = quintet, hex = hextet, h = heptet, m = multiplet, br = broad signal, dd = doublet of doublets, etc.). The $^{1}H$ NMR spectra are reported as follows: chemical shift (multiplicity, coupling constants, number of protons).

**High resolution mass spectra (HRMS)** were recorded on a Bruker Daltonics MicrOTOF II by Electrospray Ionisation (ESI); a Thermo Scientific QExactive by Electron Ionisation (EI); a Thermo Scientific Orbitrap Elite by ESI or Atmospheric Pressure Chemical Ionisation (APCI).

**IR spectra** were recorded neat as a thin film on a Perkin Elmer Spectrum One FT-IR. Selected absorption maxima ($\nu_{max}$) are reported in wavenumbers ($cm^{-1}$).

**Cyclic voltametric** (CV) experiments were performed at room temperature using MultiPalmSens 4.

**UV-Vis absorption spectra** were recorded using an Agilent Technologies Cary 300 UV/Vis spectrophotometer, in quartz cuvettes with a path length of 10 mm.

**Gas chromatography–mass spectrometry (GC-MS)** was recorded on an Agilent 6890 Series GC and 5973 detectors using a HP-5MS UI column (15 m × 0.25 mm × 0.25 μm).

## 1.3. Naming of Compounds

Compound names are those generated by ChemDraw Professional 20.0 software (PerkinElmer), following the IUPAC nomenclature.

## 1.4. Photochemical Equipment and Setup

A 40 W Kessil A160WE Tuna Blue LED lamp was used for all the photoredox reactions, with the color dial turned fully anticlockwise, and the intensity dial turned fully clockwise.

The holder for reaction vials was adapted from Leonori and co-workers (**Figure S1**).[1] All photoredox reactions were carried out at room temperature (rt, 25-30 °C) with assistance of fan cooling. The stirring rate was set to 1000 rpm. The distance between the lamp and the vial was approximately 5 cm.

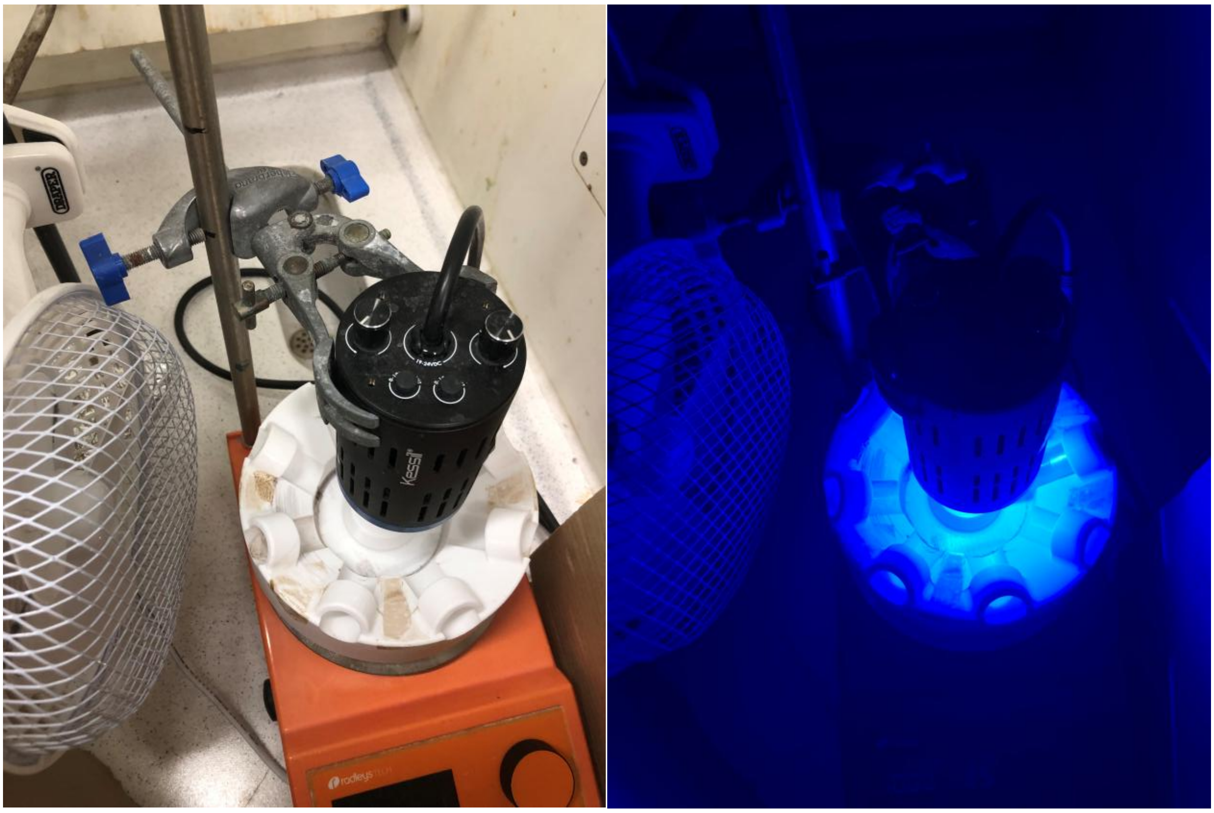


**Figure S1.** Photochemical reaction setup (0.2 mmol scale)

# 2. EXPERIMENTAL DATA

## 2.1. General Procedures

### 2.1.1. General Procedure A: Aldehyde Scope

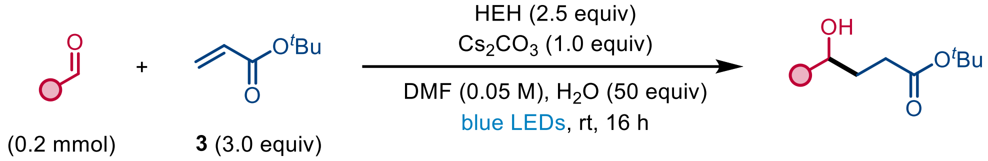


A 7 mL vial was charged with $Cs_2CO_3$ (65 mg, 0.20 mmol, 1.0 equiv), Hantzsch ester (HEH, 127 mg, 0.500 mmol, 2.50 equiv) and aldehyde (if solid, 0.20 mmol, 1.0 equiv). Anhydrous DMF (4.0 mL) and $H_2O$ (180 µL, 10.0 mmol, 50.0 equiv) were then added to the vial. Subsequently, the solution was degassed by sparging with $N_2$ using a balloon for 1 min before adding aldehyde (if liquid) and *tert*-butyl acrylate **3** (88 µL, 77 mg, 0.60 mmol, 3.0 equiv). The mixture was then irradiated with blue LEDs (see Figure S1) at room temperature for 16 h. *Workup*: The reaction mixture was then diluted with $Et_2O$ (30 mL), washed with $H_2O$ (2 × 10 mL), dried with $MgSO_4$, filtered, and concentrated *in vacuo* to give the crude product, which was purified by flash column chromatography.

### 2.1.2. General Procedure B: Olefin Scope

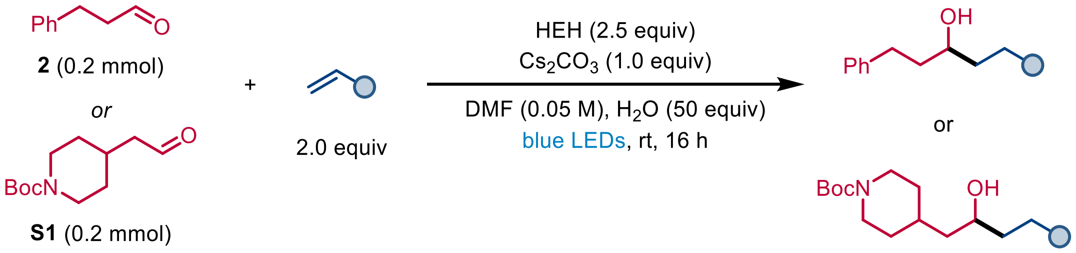


A 7 mL vial was charged with $Cs_2CO_3$ (65 mg, 0.20 mmol, 1.0 equiv), Hantzsch ester (HEH, 127 mg, 0.500 mmol, 2.50 equiv), olefin (if solid, 0.40 mmol, 2.0 equiv) and aldehyde *when N-Boc-4-piperidineacetaldehyde **S1** was used* (45.5 mg, 0.200 mmol, 1.00 equiv). Anhydrous DMF (4.0 mL) and $H_2O$ (180 µL, 10.0 mmol, 50.0 equiv) were then added to the vial. Subsequently, the solution was degassed by sparging with $N_2$ using a balloon for 1 min before adding olefin (if liquid) and aldehyde *when 3-phenylpropanal **2** was used* (26.5 µL, 26.8 mg, 0.200 mmol, 1.00 equiv). The mixture was then irradiated with blue LEDs (see Figure S1) at room temperature for 16 h. *Workup*: The reaction mixture was then diluted with $Et_2O$ (30 mL), washed with $H_2O$ (2 × 10 mL), dried with $MgSO_4$, filtered, and concentrated *in vacuo* to give the crude product, which was purified by flash column chromatography.

### 2.1.3. General Workup and Analysis Procedure

The reaction mixture was diluted with $Et_2O$ (30 mL), washed with $H_2O$ (3 × 10 mL). The organic fractions were combined, dried with $MgSO_4$ and filtered. The dried organic solution was then analysed by GC-MS with 1,4-dioxane as an internal standard to determine the yields of volatile compounds. Subsequently, the organic

solution was concentrated *in vacuo* and analysed by $^{1}$H NMR to determine the yields of non-volatile compounds using $CH_2Br_2$ as an internal standard.

## 2.2. Reaction Optimization

**Procedure:** A 7 mL vial was charged with HEH (or analogues) and a base. Anhydrous solvent and $H_2O$ were then added to the vial. Subsequently, the solution was degassed by sparging with $N_2$ using a balloon for 1 min before adding 3-phenylpropanal **2** (13 µL, 0.10 mmol, 1.0 equiv) and *tert*-butyl acrylate **3**. The mixture was then irradiated with blue LEDs (see Figure S1) at room temperature for 16 h. The reaction mixture was then diluted with $Et_2O$ (30 mL), washed with $H_2O$ (2 × 10 mL), dried with $MgSO_4$, filtered, and concentrated *in vacuo* to give the crude product. All yields were determined by $^1$H NMR analysis of the crude product using $CH_2Br_2$ as an internal standard.

**Note:** In all cases, no reduced products from the aldehyde (primary alcohol or vicinal diol) were observed.

Base screening

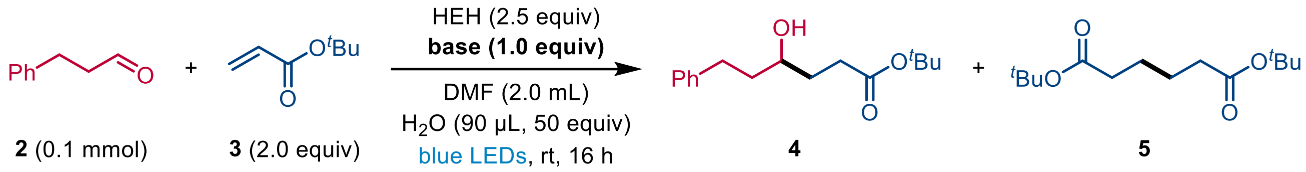


| Entry | Base | Yield of alcohol **4** (%) | Yield of dimer **5** (%) |
|---|---|---|---|
| **1** | **$Cs_2CO_3$** | **56** | **38** |
| 2 | CsOAc | 8 | <5 |
| 3 | $K_2CO_3$ | 51 | 31 |
| 4 | $Na_2CO_3$ | 13 | 6 |
| 5 | KOH | 0 | 32 |
| 6 | TMG | 40 | 15 |

**Table S1.** Base screening

Solvent screening

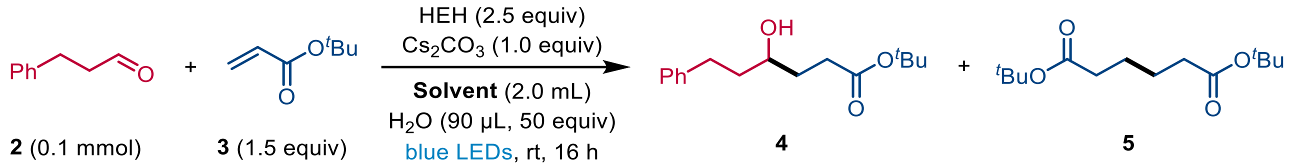


| Entry | Solvent | Yield of alcohol **4** (%) | Yield of dimer **5** (%) |
|---|---|---|---|
| 1 | THF | 0 | 0 |
| 2 | DME | 0 | 0 |
| 3 | $^t$BuOH | 0 | 0 |
| 4 | MeCN | 0 | 0 |
| 5 | DMPU | 39 | 32 |
| **6** | **DMF** | **52** | **30** |
| 7 | DMAc | 50 | 24 |
| 8 | NMP | 48 | 44 |
| 9 | DMSO | 14 | 33 |

**Table S2.** Solvent screening

Protic additive screening

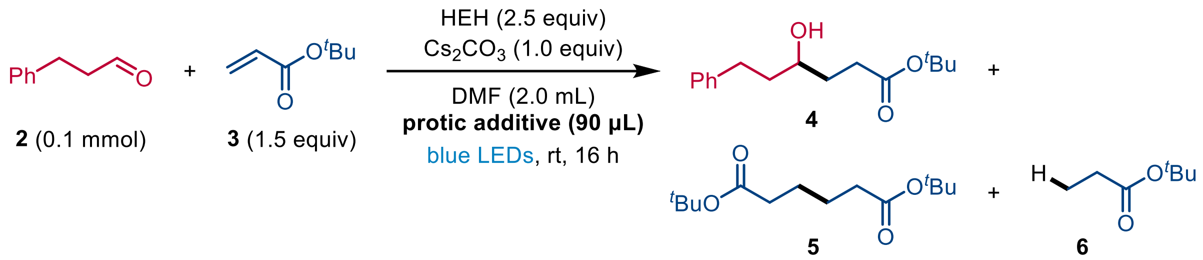


| Entry | Proton source | p$K_a$ (DMSO) | H-bond donor strength $(\alpha_1)^2$ | Yield of alcohol **4** (%) | Yield of dimer **5** (%) | Yield of side-product **6** (%) |
|---|---|---|---|---|---|---|
| 1 | $^t$BuOH | 32.2 | 0.24 | 0 | 33 | 27 |
| **2** | **$H_2O$** | **31.4** | **1.54** | **48** | **25** | **7** |
| 3 | MeCN | 31.3 | 0.23 | 0 | 30 | 26 |
| 4 | $^i$PrOH | 30 | 0.53 | 0 | 32 | 25 |
| 5 | MeOH | 29 | 1.00 | 0[a] | 10 | 22 |
| 6 | 2,2,2-Trifluoroethanol | 24 | 1.36 | 0 | 0 | 82 |
| 7 | dimethyl malonate | 16 | - | 0[b] | 9 | 5 |

**Table S3.** Protic additive screening

[a] The product of MeOH conjugate addition to the acrylate was observed in 13% yield. [b] The product of dimethylmalonate conjugate addition to the acrylate was observed in 75% yield.

Hantzsch ester analogues screening

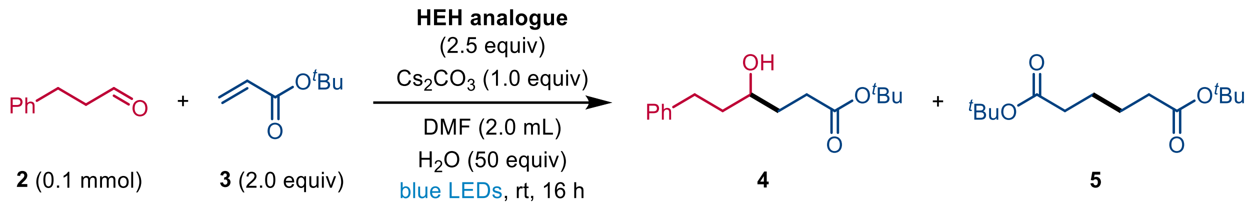


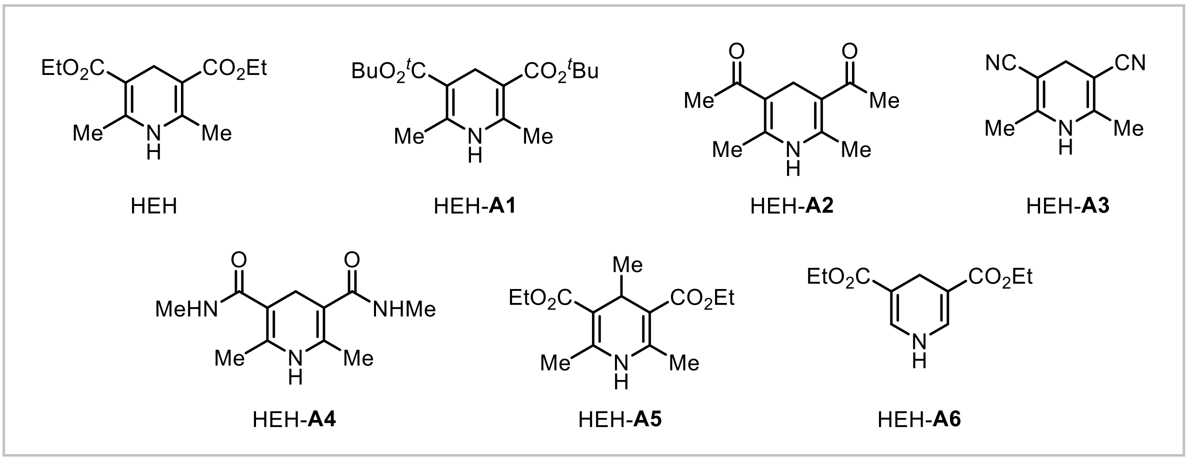


| Entry | HEH derivatives | Yield of alcohol **4** (%) | Yield of dimer **5** (%) |
|---|---|---|---|
| **1** | **HEH** | **56** | **38** |
| 2 | HEH-**A1** | 52 | 33 |
| 3 | HEH-**A2** | <5 | <5 |
| 4 | HEH-**A3** | 0 | 0 |
| 5 | HEH-**A4** | 0 | 0 |
| 6 | HEH-**A5** | 0 | 0 |
| 7 | HEH-**A6** | 0 | 0 |

**Table S4.** HEH analogues screening

Light source screening

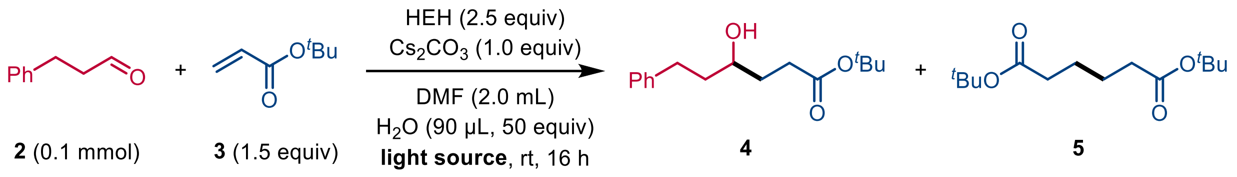


| Entry | Light source | Yield of alcohol **4** (%) | Yield of dimer **5** (%) |
|---|---|---|---|
| 1 | Kessil 390 nm | 16 | 32 |
| 2 | Kessil 427 nm | 44 | 38 |
| 3 | Kessil 456 nm | 54 | 35 |
| **4** | **Kessil Tuna Blue** | **52** | **30** |
| 5 | Merck Photoreactor (450 nm) | 55 | 36 |

**Table S5.** Light source screening

Evaluation of aldehyde and olefin stoichiometries

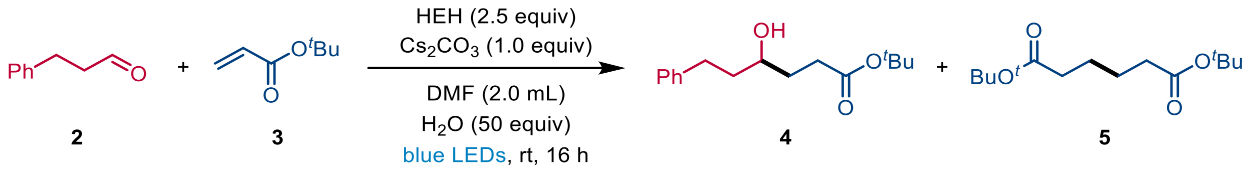


| Entry | Aldehyde **2** | Acrylate **3** | Yield of alcohol **4** (%) | Yield of dimer **5** (%) |
|---|---|---|---|---|
| 1 | 0.1 mmol | **1.5 equiv** | 52 | 30 |
| 2 | 0.1 mmol | **2.0 equiv** | 56 | 38 |
| 3 | 0.1 mmol | **3.0 equiv** | 54 | 76 |
| 4 | **2.0 equiv** | 0.1 mmol | 52 | <5 |
| 5 | **3.0 equiv** | 0.1 mmol | 54 | <5 |

**Table S6.** Evaluation of aldehyde and olefin stoichiometries

Evaluation of water volume

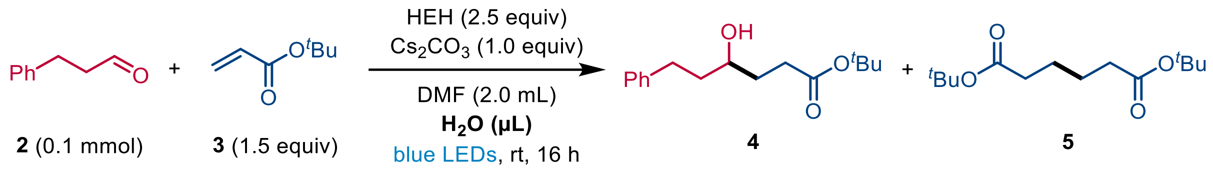


| Entry | $H_2O$ (µL) | Yield of alcohol **4** (%) | Yield of dimer **5** (%) |
|---|---|---|---|
| 1 | 0 | 0 | 28 |
| 2 | 50 | 41 | 31 |
| **3** | **90 (50 equiv)** | **52** | **30** |
| 4 | 120 | 54 | 29 |
| 5 | 200 | 53 | 29 |

**Table S7.** Evaluation of water volume

Evaluation of base equivalents

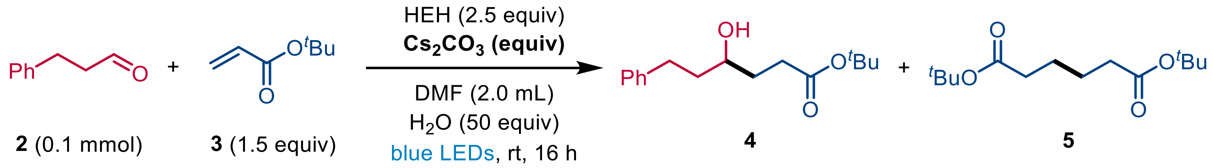


| Entry | $Cs_2CO_3$ (equiv) | Yield of alcohol **4** (%) | Yield of dimer **5** (%) |
|---|---|---|---|
| 1 | 0.5 | 54 | 31 |
| **2** | **1.0** | **52** | **30** |
| 3 | 1.5 | 48 | 28 |

**Table S8.** Evaluation of base equivalents

Evaluation of Hantzsch ester equivalents

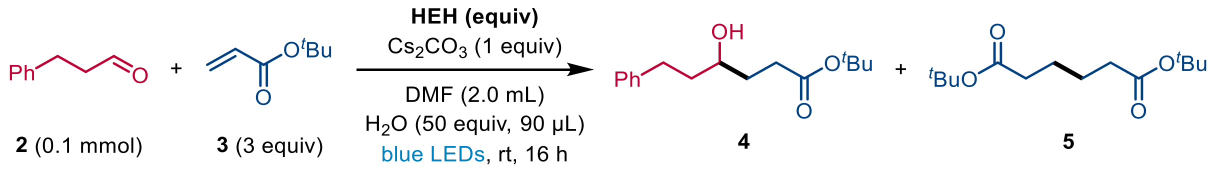


| Entry | HEH (equiv) | Yield of alcohol **4** (%) | Yield of dimer **5** (%) |
|---|---|---|---|
| 1 | 1.0 | 36 | 45 |
| 2 | 2.0 | 56 | 63 |
| 3 | 2.5 | 54 | 76 |

**Table S9.** Evaluation of HEH equivalents

## 2.3. Synthesis of Starting Materials and Reagents

*Aldehydes*

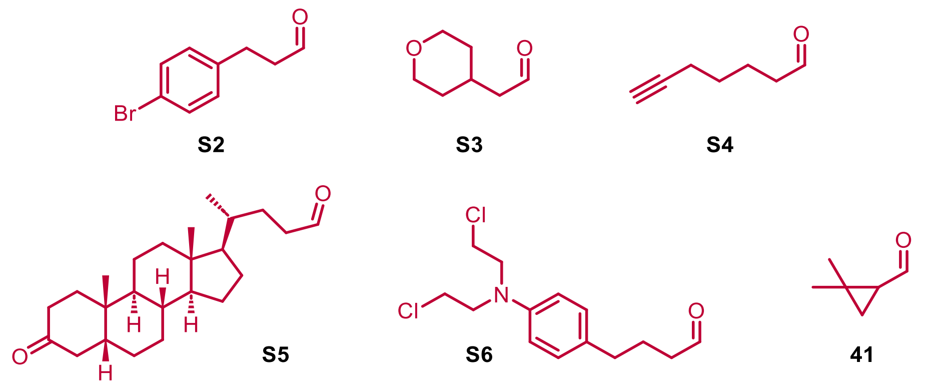


Aldehydes **S2-5** and **41**,[3] and the lithocholic acid derivative **S6**,[4] were synthesized from the corresponding carboxylic acids according to reported procedures.

*Olefins*

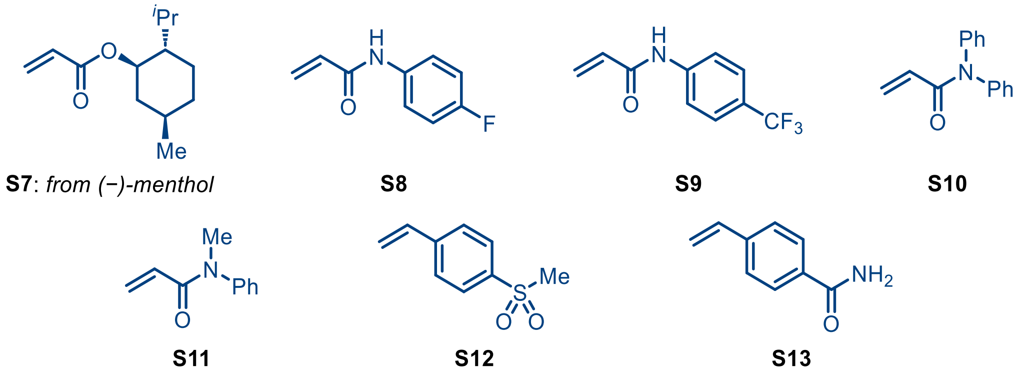


Acrylate **S7** and acrylamides **S8-S11**,[5] styrenyl sulfone **S12**,[6] and styrene amide **S13** were synthesized according to reported procedures.[7]

*HEH analogues*

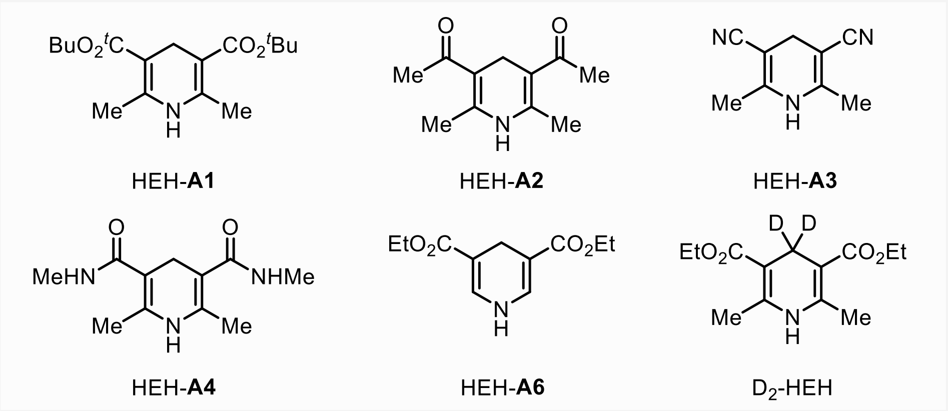


HEH analogues, including **A1** and **A2**,[8] **A3**,[9] **A4**,[10] **A6**,[11] and $D_2$-HEH[12] were all synthesized according to reported procedures. $D_2$-HEH was recrystallized from MeOH before use.

## 2.4. Substrate Scope

### 2.4.1. Aldehyde scope

***tert*-Butyl 4-hydroxy-6-phenylhexanoate (4)**

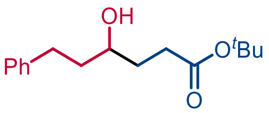


Prepared following **General Procedure A**, using 3-phenylpropanal **2** (26.8 mg). Purification by flash column chromatography (silica gel, 20:80 EA/PE) followed by prep HPLC gave **4** (27.6 mg, 52%) as a colourless oil.

**$R_f$** = 0.37 (20:80 EA/PE, $KMnO_4$).

**NMR Spectroscopy (*see spectra*):**

**$^1$H NMR** (400 MHz, $CDCl_3$): $\delta_H$ 7.30-7.26 (m, 2H), 7.23 – 7.13 (m, 3H), 3.64 (m, 1H), 2.80 (dt, *J* = 13.8, 7.7 Hz, 1H), 2.68 (dt, *J* = 13.8, 8.1 Hz, 1H), 2.37 (t, *J* = 7.1 Hz, 2H), 2.04 (br. s, 1H), 1.87 – 1.68 (m, 4H), 1.43 (s, 9H) ppm.

**$^{13}$C NMR** (101 MHz, $CDCl_3$): $\delta_C$ 173.9, 142.1, 128.6, 126.0, 80.7, 70.9, 39.4, 32.5, 32.23, 32.15, 28.2 ppm.

All recorded spectroscopic data matched those previously reported in the literature.[13]

***tert*-Butyl 4-hydroxyhexanoate (7)**

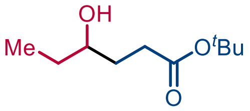


Prepared following **General Procedure A**, using propionaldehyde (11.6 mg). Purification by flash column chromatography (silica gel, 20:80 EA/PE) followed by prep HPLC gave **7** (16.8 mg, 45%) as a colourless oil.

**$R_f$** = 0.52 (20:80 EA/PE, $KMnO_4$).

**NMR Spectroscopy (*see spectra*):**

**$^1$H NMR** (400 MHz, $CDCl_3$): $\delta_H$ 3.54 (dtt, *J* = 9.1, 5.7, 3.5 Hz, 1H), 2.37 (t, *J* = 7.2 Hz, 2H), 1.82 (br.s, 1H), 1.80 (dtd, *J* = 14.5, 7.2, 3.5 Hz, 1H), 1.65 (ddt, *J* = 14.5, 9.1, 7.2 Hz, 1H), 1.54-1.47 (m, 2H), 1.44 (s, 9H), 0.94 (t, *J* = 7.4 Hz, 3H) ppm.

**$^{13}$C NMR** (101 MHz, $CDCl_3$): $\delta_C$ 173.9, 80.6, 72.9, 32.2, 31.9; 30.5, 28.2, 10.1 ppm.

All recorded spectroscopic data matched those previously reported in the literature.[14]

**_tert_-Butyl 6-(4-bromophenyl)-4-hydroxyhexanoate (8)**

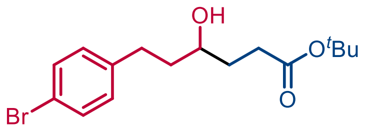


Prepared following **General Procedure A**, using 3-(4-bromophenyl)propanal **S2** (42.6 mg). Purification by flash column chromatography (silica gel, 20:80 EA/PE) followed by prep HPLC gave **8** (39.6 mg, 58%) as a yellow oil.

**$R_f$** = 0.27 (20:80 EA/PE, $KMnO_4$).

**NMR Spectroscopy (_see spectra_):**

**$^1$H NMR** (400 MHz, $CDCl_3$): $\delta_H$ 7.40 – 7.36 (m, 2H), 7.09 – 7.05 (m, 2H), 3.64 – 3.58 (m, 1H), 2.80 – 2.69 (m, 1H), 2.69 – 2.58 (m, 1H), 2.37 (t, $J$ = 7.0 Hz, 2H), 1.81 – 1.66 (m, 4H), 1.43 (s, 9H) ppm.

**$^{13}$C NMR** (101 MHz, $CDCl_3$): $\delta_C$ 173.8, 141.1, 131.6, 130.4, 119.7, 80.8, 70.7, 39.2, 32.5, 32.2, 31.5, 28.2 ppm.

**IR** (film): $v_{max}$ 3439, 2977, 2929, 2861, 1726, 1488, 1367, 1254, 1149 cm$^{-1}$.

**HRMS (ESI$^+$):** calc'd for $[M+Na]^+$, 365.0728; found 365.0736.

**_tert_-Butyl 8-chloro-4-hydroxyoctanoate (9)**

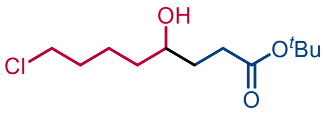


Prepared following **General Procedure A**, using 5-chloro-pentanal (24.1 mg). Purification by flash column chromatography (silica gel, 20:80 EA/PE) followed by prep HPLC gave **9** (36.2 mg, 72%) as a colourless oil.

**$R_f$** = 0.31 (20:80 EA/PE, $KMnO_4$).

**NMR Spectroscopy (_see spectra_):**

**$^1$H NMR** (400 MHz, $CDCl_3$): $\delta_H$ 3.65 – 3.59 (m, 1H), 3.54 (t, $J$ = 6.7 Hz, 2H), 2.37 (t, $J$ = 7.1 Hz, 2H), 1.93 (br. s, 1H), 1.83 – 1.74 (m, 3H), 1.71 – 1.63 (m, 1H), 1.63 – 1.55 (m, 1H), 1.53 – 1.46 (m, 3H), 1.44 (s, 9H) ppm.

**$^{13}$C NMR** (101 MHz, $CDCl_3$): $\delta_C$ 173.8, 80.7, 71.3, 45.1, 36.9, 32.7, 32.4, 32.2, 28.2, 23.2 ppm.

**IR** (film): $v_{max}$ 3433, 2977, 2936, 2869, 1727, 1368, 1256, 1154 cm$^{-1}$.

**HRMS (ESI$^+$):** calc'd for $[M+Na]^+$, 273.1233; found 273.1232.

**_tert_-Butyl 4-hydroxy-5-(tetrahydro-2_H_-pyran-4-yl)pentanoate (10)**

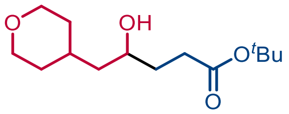


Prepared following **General Procedure A**, using 2-(tetrahydro-2*H*-pyran-4-yl)acetaldehyde **S3** (25.6 mg). Purification by flash column chromatography (silica gel, 40:60 to 60:40 EA/PE) gave **10** (28.8 mg, 56%) as a colourless oil.

**$R_f$** = 0.26 (40:60 EA/PE, $KMnO_4$).

**NMR Spectroscopy (_see spectra_):**

**$^1$H NMR** (500 MHz, $CDCl_3$): $\delta_H$ 3.94 (dt, *J* = 12.0, 5.7 Hz, 2H), 3.78 – 3.68 (m, 1H), 3.42 – 3.33 (m, 2H), 2.37 (t, *J* = 7.1 Hz, 2H), 2.00 (br. s, 1H), 1.81 – 1.60 (m, 5H), 1.47 – 1.41 (m, 1H), 1.44 (s, 9H), 1.35 – 1.20 (m, 3H) ppm.

**$^{13}$C NMR** (126 MHz, $CDCl_3$): $\delta_C$ 173.8, 80.8, 68.4, 68.2, 68.1, 45.0, 34.0, 33.0, 32.9, 32.2, 31.7, 28.2 ppm.

**IR** (film): $v_{max}$ 3441, 2925, 2841, 1727, 1444, 1367, 1256, 1151, 1093 cm$^{-1}$.

**HRMS (ESI$^+$):** calc'd for $[M+H]^+$, 281.1729; found 281.1724.

**_tert_-Butyl 4-(5-(_tert_-butoxy)-2-hydroxy-5-oxopentyl)piperidine-1-carboxylate (11)**

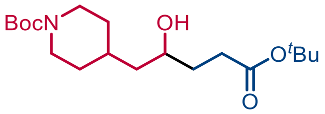


Prepared following **General Procedure A**, using *N*-Boc-4-piperidineacetaldehyde **S1** (45.5 mg). Purification by flash column chromatography (silica gel, 20:80 EA/PE) followed by prep HPLC gave **11** (48.6 mg, 68%) as a colorless oil.

**$R_f$** = 0.44 (40:60 EA/PE, $KMnO_4$).

**NMR Spectroscopy (_see spectra_):**

**$^1$H NMR** (400 MHz, $CDCl_3$): $\delta_H$ 4.14 – 4.00 (m, 2H), 3.74 (tt, *J* = 8.4, 3.8 Hz, 1H), 2.69 (m, 2H), 2.37 (t, *J* = 7.0 Hz, 2H), 1.82 – 1.58 (m, 6H), 1.48 – 1.40 (m, 1H), 1.45 (s, 9H), 1.44 (s, 9H), 1.32 – 1.26 (m, 1H), 1.20 – 0.99 (m, 2H) ppm.

**$^{13}$C NMR** (101 MHz, $CDCl_3$): $\delta_C$ 173.9, 155.0, 80.8, 79.4, 68.6, 44.6, 44.2, 33.0, 32.6, 32.2, 31.8, 28.6, 28.2 ppm.

**IR** (film): $v_{max}$ 3447, 2976, 2927, 2861, 1728, 1695, 1672, 1424, 1366, 1248, 1154 cm$^{-1}$.

**HRMS (ESI$^+$):** calc'd for $[M+Na]^+$, 380.2407; found 380.2408.

**tert-Butyl 5-(benzyloxy)-4-hydroxypentanoate (12)**

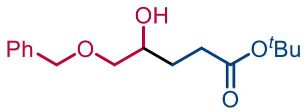


Prepared following **General Procedure A**, using benzyloxy acetaldehyde (30 mg). Purification by flash column chromatography (silica gel, 20:80 EA/PE) followed by prep HPLC gave **12** (16.3 mg, 29%) as a colourless oil.

**R$_f$** = 0.32 (20:80 EA/PE, $KMnO_4$).

**NMR Spectroscopy (*see spectra*):**

**$^1$H NMR** (400 MHz, $CDCl_3$): $\delta_H$ 7.39 – 7.27 (m, 5H), 4.55 (s, 2H), 3.83 (tt, *J* = 7.8, 3.5 Hz, 1H), 3.50 (dd, *J* = 9.4, 3.5 Hz, 1H), 3.36 (dd, *J* = 9.4, 7.4 Hz, 1H), 2.55 (br. s, 1H), 2.46 – 2.29 (m, 2H), 1.82 – 1.65 (m, 2H), 1.44 (s, 9H) ppm.

**$^{13}$C NMR** (101 MHz, $CDCl_3$): $\delta_C$ 173.3, 138.1, 128.6, 127.94, 127.88, 80.5, 74.5, 73.5, 69.9, 31.8, 28.5, 28.2 ppm.

**IR** (film): $\nu_{max}$ 3457, 2977, 2929, 2861, 1728, 1454, 1367, 1256, 1152 cm$^{-1}$.

**HRMS (ESI$^+$):** calc'd for $[M+Na]^+$, 303.1567; found 303.1560.

**tert-Butyl 4-hydroxydec-9-ynoate (13)**

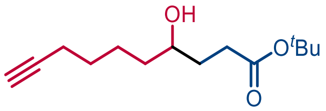


Prepared following **General Procedure A**, using hept-6-ynal **S4** (22 mg). Purification by flash column chromatography (silica gel, 20:80 EA/PE) followed by prep HPLC gave **13** (29.6 mg, 62%) as a colourless oil.

**R$_f$** = 0.33 (20:80 EA/PE, $KMnO_4$).

**NMR Spectroscopy (*see spectra*):**

**$^1$H NMR** (400 MHz, $CDCl_3$): $\delta_H$ 3.65 – 3.58 (m, 1H), 2.37 (t, *J* = 7.2 Hz, 2H), 2.20 (td, *J* = 6.7, 2.6 Hz, 2H), 1.96 (d, *J* = 5.2 Hz, 1H), 1.94 (t, *J* = 2.6 Hz, 1H), 1.79 (dtd, *J* = 14.4, 7.2, 3.6 Hz, 1H), 1.71 – 1.61 (m, 1H), 1.58 – 1.45 (m, 6H), 1.44 (s, 9H) ppm.

**$^{13}$C NMR** (101 MHz, $CDCl_3$): $\delta_C$ 173.8, 84.6, 80.7, 71.4, 68.5, 37.2, 32.4, 32.2, 28.5, 28.2, 24.9, 18.5 ppm.

**IR** (film): $\nu_{max}$ 3445, 3306, 2935, 2863, 1726, 1367, 1255, 1151, 1099 cm$^{-1}$.

**HRMS (ESI$^+$):** calc'd for $[M+Na]^+$, 263.1618 ; found 263.1624.

**_tert_-Butyl 4-hydroxy-6,10-dimethylundec-9-enoate (14)**

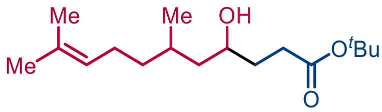


Prepared following **General Procedure A**, using citronellal (30.9 mg). Purification by flash column chromatography (silica gel, 10:90 to 20:80 EA/PE) followed by prep HPLC gave **14** (27.4 mg, 48%, 1.1:1 *dr*) as a colourless oil.

**R$_f$** = 0.15 (10:90 EA/PE, $KMnO_4$).

**NMR Spectroscopy:**

*Diastereomer 1* (***see spectra***)

$^1$**H NMR** (400 MHz, $CDCl_3$): $\delta_H$ 5.09 (t, *J* = 7.1 Hz, 1H), 3.71 (tt, *J* = 8.2, 3.6 Hz, 1H), 2.37 (t, *J* = 7.3 Hz, 2H), 2.06 – 1.91 (m, 2H), 1.83 – 1.62 (m, 4H), 1.68 (s, 3H), 1.60 (s, 3H), 1.51 – 1.40 (m, 1H), 1.45 (s, 9H), 1.36 – 1.11 (m, 3H), 0.90 (d, *J* = 6.6 Hz, 3H) ppm.

$^{13}$**C NMR** (101 MHz, $CDCl_3$): $\delta_C$ 173.8, 131.4, 124.9, 80.6, 69.2, 45.1, 38.0, 33.3, 32.3, 29.0, 28.2, 25.9, 25.6, 19.3, 17.8 ppm.

*Diastereomer 2* (***see spectra***)

$^1$**H NMR** (400 MHz, $CDCl_3$): $\delta_H$ 5.13 – 5.04 (m, 1H), 3.71 (tdd, *J* = 8.4, 5.6, 3.4 Hz, 1H), 2.37 (t, *J* = 7.2 Hz, 2H), 2.08 – 1.87 (m, 2H), 1.84 – 1.75 (m, 1H), 1.70 – 1.57 (m, 3H), 1.68 (s, 3H), 1.60 (s, 3H), 1.45 (s, 9H), 1.41 – 1.25 (m, 3H), 1.20 – 1.05 (m, 1H), 0.91 (d, *J* = 6.6 Hz, 3H) ppm.

$^{13}$**C NMR** (101 MHz, $CDCl_3$): $\delta_C$ 173.8, 131.4, 124.9, 80.6, 69.6, 45.3, 36.9, 32.6, 32.2, 29.4, 28.2, 25.9, 25.5, 20.3, 17.8 ppm.

**IR** (film): $\nu_{max}$ 3437, 2961, 2925, 2865, 1789, 1455, 1367, 1254, 1151, 1104 cm$^{-1}$.

**HRMS (ESI$^+$):** calc'd for [M+Na]$^+$, 307.2244; found 307.2232.

**_tert_-Butyl(7*R*)-7-((5*R*,8*R*,9*S*,10*S*,13*R*,14*S*,17*R*)-10,13-dimethyl-3-oxohexadecahydro-1*H*-cyclopenta[a]phenanthren-17-yl)-4-hydroxyoctanoate (15)**

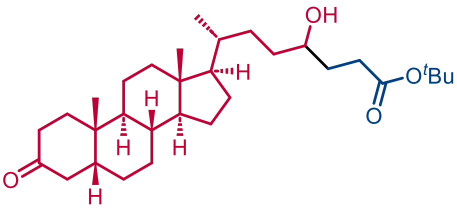


Prepared following **General Procedure A**, using the lithocholic acid-derived aldehyde **S5** (71.7 mg). Purification by flash column chromatography (silica gel, 20:80 EA/PE) followed by prep TLC (20:80 EA/PE) gave **15** (50.9 mg, 52%, 1.3:1 *dr*.) as a yellow oil. The *dr* was determined by quantitative $^{13}$C NMR of the purified product. The yield of unreacted aldehyde **S5** (17%) was determined by $^1$H NMR analysis of the crude product.

**R$_f$** = 0.26 (20:80 EA/PE, $KMnO_4$).

**NMR Spectroscopy (*see spectra*):**

**$^1$H NMR** (400 MHz, $CDCl_3$): $\delta_H$ 3.62 – 3.47 (m, 1H), 2.69 (t, *J* = 14.2 Hz, 1H), 2.36 (t, *J* = 7.2 Hz, 2H), 2.31 (m, 1H), 2.19 – 2.11 (m, 1H), 2.07 – 1.97 (m, 3H), 1.93 – 1.73 (m, 5H), 1.70 – 1.57 (m, 2H), 1.53 – 1.11 (m, 18H), 1.44 (s, 9H), 1.01 (s, 3H), 0.91 (d, *J* = 6.5 Hz; d, *J* = 6.4 Hz; 3H), 0.67 (s, 3H) ppm.

**$^{13}$C NMR** (101 MHz, $CDCl_3$): $\delta_C$ 213.6, 173.9, 173.8, 80.6, 72.2, 71.9, 56.6, 56.2, 44.5, 42.9, 42.5, 40.9, 40.2, 37.4, 37.2, 35.9, 35.73, 35.66, 35.0, 34.2, 34.1, 32.5, 32.24, 32.17, 31.9, 31.8, 28.42, 28.38, 28.2, 26.8, 25.9, 24.3, 22.8, 21.3, 18.84, 18.77, 12.2 ppm.

**IR** (film): $\nu_{max}$ 3452, 2930, 2865, 1716, 1454, 1366, 1255, 1149, 1100 cm$^{-1}$.

**HRMS (ESI$^+$):** calc'd for $[M+Na]^+$, 511.3763; found 511.3755.

### ***tert*-Butyl 7-(4-(bis(2-chloroethyl)amino)phenyl)-4-hydroxyheptanoate (16)**

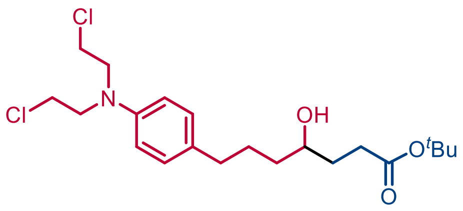


Prepared following **General Procedure A**, using chlorambucil-derived aldehyde **S6** (57.6 mg). Purification by flash column chromatography (silica gel, 30:70 EA/PE) followed by prep HPLC gave **16** (34.3 mg, 41%) as a colourless oil. The yield of unreacted aldehyde **S6** (18%) was determined by $^1$H NMR analysis of the crude product.

**R$_f$** = 0.19 (20:80 EA/PE, $KMnO_4$).

**NMR Spectroscopy (*see spectra*):**

**$^1$H NMR** (400 MHz, $CDCl_3$): $\delta_H$ 7.10 – 7.03 (m, 2H), 6.65 – 6.58 (m, 2H), 3.74 – 3.66 (m, 4H), 3.62 (m, 5H), 2.54 (t, *J* = 7.2 Hz, 2H), 2.36 (t, *J* = 7.2 Hz, 2H), 1.88 (d, *J* = 5.2 Hz, 1H), 1.84 – 1.60 (m, 4H), 1.52 – 1.46 (m, 2H), 1.44 (s, 9H) ppm.

**$^{13}$C NMR** (101 MHz, $CDCl_3$): $\delta_C$ 173.8, 144.3, 131.7, 129.7, 112.3, 80.6, 71.5, 53.8, 40.7, 37.3, 34.8, 32.4, 32.2, 28.2, 27.8 ppm.

**IR** (film): $\nu_{max}$ 3439, 2974, 2932, 2858, 1725, 1615, 1519, 1366, 1250, 1154 cm$^{-1}$.

**HRMS (ESI$^+$):** calc'd for $[M+H]^+$, 418.1910; found 418.1899.

**_tert_-Butyl 4-cyclopropyl-4-hydroxybutanoate (17)**

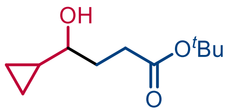


Prepared following **General Procedure A**, using cyclopropane carboxaldehyde (14 mg). Purification by flash column chromatography (silica gel, 20:80 EA/PE) followed by prep TLC (20:80 EA/PE) gave **17** (9.8 mg, 25%) as a colourless oil.

**$R_f$** = 0.29 (20:80 EA/PE, $KMnO_4$).

**NMR Spectroscopy (_see spectra_):**

**$^1$H NMR** (400 MHz, $CDCl_3$): $\delta_H$ 2.90 (td, *J* = 8.1, 4.5 Hz, 1H), 2.40 (t, *J* = 7.3 Hz, 2H), 1.98 – 1.79 (m, 2H), 1.44 (s, 9H), 0.89 (m, 1H), 0.57 – 0.45 (m, 2H), 0.29 (m, 1H), 0.25 – 0.16 (m, 1H) ppm.

**$^{13}$C NMR** (101 MHz, $CDCl_3$): $\delta_C$ 173.7, 80.5, 76.2, 32.18, 32.15, 28.2, 17.8, 2.8, 2.7 ppm.

**IR** (film): $\nu_{max}$ 3437, 2973, 2927, 2861, 1727, 1367, 1263, 1153 cm$^{-1}$.

**HRMS (ESI$^+$):** calc'd for $[M+Na]^+$, 223.1305; found 223.1299.

**_tert_-Butyl 4-cyclobutyl-4-hydroxybutanoate (18)**

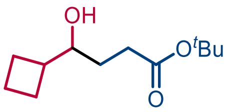


Prepared following **General Procedure A**, using cyclobutyl aldehyde (16.8 mg). Purification by flash column chromatography (silica gel, 20:80 EA/PE) followed by prep TLC (20:80 EA/PE) gave **18** (26.2 mg, 61%) as a colourless oil.

**$R_f$** = 0.41 (20:80 EA/PE, $KMnO_4$).

**NMR Spectroscopy (_see spectra_):**

**$^1$H NMR** (400 MHz, $CDCl_3$): $\delta_H$ 3.50 (ddd, *J* = 9.0, 7.7, 3.1 Hz, 1H), 2.43 – 2.19 (m, 3H), 2.09 – 1.64 (m, 9H), 1.44 (s, 9H) ppm.

**$^{13}$C NMR** (101 MHz, $CDCl_3$): $\delta_C$ 173.9, 80.5, 75.5, 41.6, 32.2, 29.5, 28.2, 24.5, 18.0 ppm.

**IR** (film): $\nu_{max}$ 3445, 2975, 2933, 2865, 1728, 1367, 1251, 1151 cm$^{-1}$.

**HRMS (ESI$^+$):** calc'd for $[M+Na]^+$, 237.1461; found 237.1454.

**_tert_-Butyl 4-cyclohexyl-4-hydroxybutanoate (19)**

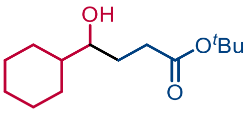

Prepared following **General Procedure A**, using cyclohexane carboxaldehyde (22.4 mg). Purification by flash column chromatography (silica gel, 20:80 EA/PE) followed by prep HPLC gave **19** (27.1 mg, 56%) as a colourless oil.

**$R_f$** = 0.18 (10:90 EA/PE, $KMnO_4$).

**NMR Spectroscopy (_see spectra_):**

$^1$**H NMR** (400 MHz, $CDCl_3$): $\delta_H$ 3.34 (ddd, $J$ = 9.1, 5.6, 3.0 Hz, 1H), 2.47 – 2.28 (m, 2H), 1.88 – 1.71 (m, 5H), 1.69 – 1.59 (m, 3H), 1.44 (s, 9H), 1.37 – 0.93 (m, 6H) ppm.

$^{13}$**C NMR** (101 MHz, $CDCl_3$): $\delta_C$ 174.0, 80.5, 75.9, 44.0, 32.6, 29.3, 29.2, 28.2, 28.1, 26.6, 26.4, 26.3 ppm.

**IR** (film): $\nu_{max}$ 3442, 2977, 2924, 2853, 1727, 1449, 1367, 1255, 1150 cm$^{-1}$.

**HRMS (ESI$^+$):** calc'd for $[M+Na]^+$, 265.1774; found 265.1776.

**_tert_-Butyl 4-hydroxy-4-(tetrahydro-2_H_-pyran-4-yl)butanoate (20)**

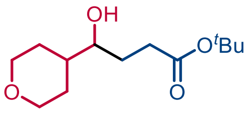

Prepared following **General Procedure A**, using tetrahydro-2*H*-pyran-4-carbaldehyde (22.8 mg). Purification by flash column chromatography (silica gel, 30:70 to 40:60 EA/PE) followed by prep HPLC gave **20** (25.9 mg, 53%) as a colourless oil.

**$R_f$** = 0.25 (40:60 EA/PE, $KMnO_4$).

**NMR Spectroscopy (_see spectra_):**

$^1$**H NMR** (400 MHz, $CDCl_3$): $\delta_H$ 4.00 (m, 2H), 3.42 – 3.31 (m, 3H), 2.41 – 2.37 (m, 2H), 1.86 – 1.78 (m, 1H), 1.77 – 1.71 (m, 1H), 1.68 – 1.60 (m, 1H), 1.60 – 1.49 (m, 2H), 1.44 (s, 9H), 1.46 – 1.36 (m, 2H) ppm.

$^{13}$**C NMR** (101 MHz, $CDCl_3$): $\delta_C$ 174.0, 80.8, 75.2, 68.1, 67.9, 41.4, 32.4, 29.14, 29.05, 28.5, 28.2 ppm.

**IR** (film): $\nu_{max}$ 3441, 2934, 2848, 1727, 1392, 1367, 1257, 1153, 1091 cm$^{-1}$.

**HRMS (ESI$^+$):** calc'd for $[M+H]^+$, 245.1747; found 245.1752.

### *tert*-Butyl 4-(4-(*tert*-butoxy)-1-hydroxy-4-oxobutyl)piperidine-1-carboxylate (21)

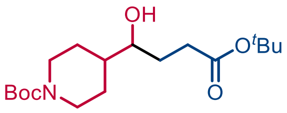


Prepared following **General Procedure A**, using *N*-Boc-4-piperidinecarboxaldehyde (42.7 mg). Purification by flash column chromatography (silica gel, 40:60 EA/PE) followed by prep HPLC gave **21** (37.2 mg, 54%) as a colorless oil.

**$R_f$** = 0.50 (40:60 EA/PE, $KMnO_4$).

**NMR Spectroscopy (*see spectra*):**

$^{1}$**H NMR** (400 MHz, $CDCl_3$): $\delta_H$ 4.20 – 4.07 (m, 2H), 3.37 (ddd, *J* = 9.2, 6.0, 2.9 Hz, 1H), 2.65 (m, 2H), 2.39 (t, *J* = 7.0 Hz, 2H), 1.94 (br.s, 1H), 1.85 – 1.77 (m, 2H), 1.71 – 1.54 (m, 2H), 1.50 – 1.39 (m, 1H), 1.44 (s, 9H), 1.44 (s, 9H), 1.32 – 1.16 (m, 2H) ppm.

$^{13}$**C NMR** (101 MHz, $CDCl_3$): $\delta_C$ 174.0, 155.0, 80.8, 79.5, 75.0, 43.7, 42.5, 32.4, 29.2, 28.6, 28.3, 28.2, 27.5 ppm.

**IR** (film): $v_{max}$ 3448, 2976, 2929, 2861, 1728, 1694, 1674, 1425, 1366, 1251, 1160 $cm^{-1}$.

**HRMS (ESI$^+$):** calc'd for $[M+Na]^+$, 366.2251; found 366.2242.

### *tert*-Butyl 2-(4-(tert-butoxy)-1-hydroxy-4-oxobutyl)pyrrolidine-1-carboxylate (22)

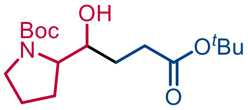


Prepared following **General Procedure A**, using 1-Boc-2-formylpyrrolidine (39.9 mg). Purification by flash column chromatography (silica gel, 20:80 EA/PE) followed by prep HPLC gave the major diastereomer of **22** (12.1 mg, 18%) as a colourless oil.

**$R_f$** = 0.48 (30:70 EA/PE, $KMnO_4$).

**NMR Spectroscopy (*see spectra*):**

$^{1}$**H NMR** (400 MHz, $CDCl_3$): $\delta_H$ 3.78 (td, *J* = 8.3, 4.1 Hz, 1H), 3.54 – 3.41 (m, 2H), 3.28 (ddd, *J* = 10.8, 7.2, 5.6 Hz, 1H), 2.53 – 2.34 (m, 2H), 2.01 – 1.92 (m, 1H), 1.87 – 1.63 (m, 5H), 1.61 – 1.50 (m, 1H), 1.46 (s, 9H), 1.43 (s, 9H) ppm.

$^{13}$**C NMR** (101 MHz, $CDCl_3$): $\delta_C$ 173.6, 158.2, 80.6, 80.2, 75.1, 62.9, 47.4, 31.4, 30.3, 28.8, 28.6, 28.3, 24.3 ppm.

**IR** (film): $v_{max}$ 3428, 2976, 2931, 2885, 1728, 1693, 1667, 1393, 1366, 1255, 1152, 1104 $cm^{-1}$.

**HRMS (ESI$^+$):** calc'd for $[M+H]^+$, 330.2275; found 330.2270.

**tert-Butyl 4-hydroxy-5,9-dimethyldec-8-enoate (23)**

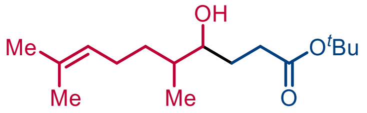


Prepared following **General Procedure A**, using 2,6-dimethyl-5-heptenal (*melonal*, 28.0 mg). The yield of **23** (31%) was determined by $^1$H NMR analysis of the crude product using $CH_2Br_2$ as internal standard. The product **23** was difficult to separate from oxidized Hantzsch ester; thus, an accurate isolated yield could not be obtained. An analytically pure sample (colourless oil, 2.5:1 *dr.*) was obtained after flash column chromatography (silica gel, 20:80 EA/PE) followed by preparative TLC (10:90 EA/PE).

**R$_f$** = 0.17 (10:90 EA/PE, $KMnO_4$).

**NMR Spectroscopy (*see spectra*):**

**$^1$H NMR** (500 MHz, $CDCl_3$): $\delta_H$ 5.10 (t, *J* = 7.3 Hz, 1H), 3.55 – 3.46 and 3.46 – 3.40 (m; m; 1H), 2.46 – 2.30 (m, 2H), 2.13 – 1.87 (m, 2H), 1.77 – 1.66 (m, 2H), 1.68 (s, 3H), 1.60 (s, 3H), 1.52 – 1.42 (m, 2H), 1.45 (s, 9H), 1.23 – 1.13 (m, 1H), 0.91 and 0.90 (d, *J* = 6.6 Hz; d, *J* = 6.6 Hz; 3H) ppm.

**$^{13}$C NMR** (126 MHz, $CDCl_3$): (*chemical shifts in italics correspond to the minor diastereomer*) $\delta_C$ *174.0*, 173.9; *131.68*, 131.67, *124.72*, 124.68; *80.60*, 80.58; *75.7*, 75.0; *38.8*, 38.4; 33.3, 32.9, *32.7*, *32.4*; *29.9*, 29.6; 28.3, 25.9; 25.84, *25.77*; 17.84, *17.83*; 15.1, *13.9* ppm.

**IR** (film): $\nu_{max}$ 3538, 3443, 2930, 2854, 1727, 1611, 1316, 1264, 1096 cm$^{-1}$.

**HRMS (ESI$^+$):** calc'd for [M+Na]$^+$, 293.2087; found 293.2093.

**tert-Butyl 4-hydroxy-5,5-dimethylhexanoate (24)**

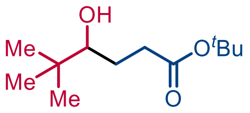


Prepared following **General Procedure A**, using pivaldehyde (17.2 mg). The yield of **24** (12%) was determined by $^1$H NMR analysis of the crude product using $CH_2Br_2$ as internal standard. Purification was not attempted due to the low yield.

**NMR Spectroscopy (*see spectra*):**

**$^1$H NMR** (400 MHz, $CDCl_3$): $\delta_H$ 3.16 (dd, *J* = 10.7, 3,4 Hz, 1H), 0.88 (s, 9H) ppm. The remaining signals could not be determined due to signal overlap.

All recorded spectroscopic data matched those previously reported in the literature.[15]

**tert-Butyl 4-hydroxy-4-phenylbutanoate (25)**

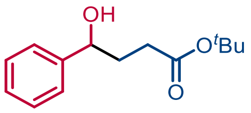


Prepared following **General Procedure A**, using benzaldehyde (21.2 mg). Purification by flash column chromatography (silica gel, 20:80 EA/PE) followed by prep TLC (20:80 EA/PE) gave **25** (22.3 mg, 47%) as a colourless oil.

**$R_f$** = 0.39 (20:80 EA/PE, $KMnO_4$).

**NMR Spectroscopy (*see spectra*):**

**$^1$H NMR** (400 MHz, $CDCl_3$): $\delta_H$ 7.36 – 7.34 (m, 4H), 7.30 – 7.27 (m, 1H), 4.75 (td, J = 6.5, 3.2 Hz, 1H), 2.42 (d, J = 3.2 Hz, 1H), 2.34 (t, J = 7.2 Hz, 2H), 2.05 – 2.01 (m, 2H), 1.45 (s, 9H) ppm.

**$^{13}$C NMR** (101 MHz, $CDCl_3$): $\delta_C$ 173.5, 144.4, 128.6, 127.7, 125.9, 80.7, 73.9, 34.1, 32.1, 28.2 ppm.

All recorded spectroscopic data matched those previously reported in the literature.[16]

### 2.4.2. Olefin scope

**1,7,7-Trimethylbicyclo[2.2.1]heptan-2-yl 4-hydroxy-6-phenylhexanoate (26)**

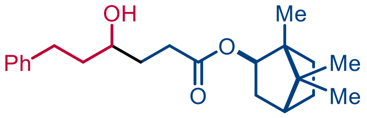


Prepared following **General Procedure B**, using 3-phenylpropanal (26.5 µL, 0.200 mmol) and isobornyl acrylate (83.3 mg, 0.400 mmol). Purification by flash column chromatography (silica gel, 20:80 EA/PE) followed by prep TLC (20:80 EA/PE) gave **26** (30.4 mg, 44%, 1:1 *dr*) as a colourless oil. (*dr*. determined by quantitative $^{13}C$ NMR)

**R$_f$** = 0.48 (20:80 EA/PE, $KMnO_4$).

**NMR Spectroscopy (*see spectra*):**

$^1$**H NMR** (400 MHz, $CDCl_3$): $\delta_H$ 7.31 – 7.26 (m, 2H), 7.21 – 7.15 (m, 3H), 4.69 – 4.62 (m, 1H), 3.70 – 3.60 (m, 1H), 2.80 (dt, *J* = 13.7, 7.7 Hz, 1H), 2.68 (dt, *J* = 13.7, 8.1 Hz, 1H), 2.49 – 2.37 (m, 2H), 1.92 – 1.65 (m, 9H), 1.59 – 1.50 (m, 1H), 1.18 – 1.04 (m, 2H), 0.96 (s×2, 3H), 0.84 – 0.82 (s×3, 6H) ppm.

$^{13}$**C NMR** (101 MHz, $CDCl_3$): $\delta_C$ 173.82, 173.80, 142.1, 128.58, 128.55, 126.0, 81.34, 81.32, 70.8, 48.81, 48.77, 47.1, 45.2, 39.4, 38.9, 33.9, 32.5, 32.2, 31.34, 31.31, 27.2, 20.3, 20.1, 11.6 ppm.

**IR** (film): $\nu_{max}$ 3451, 2952, 2874, 1728, 1454, 1264, 1053 cm$^{-1}$.

**HRMS (ESI$^+$):** calc'd for $[M+Na]^+$, 367.2244; found 367.2230.

**(1*R*,2*S*,5*R*)-2-Isopropyl-5-methylcyclohexyl 4-hydroxy-6-phenylhexanoate (27)**

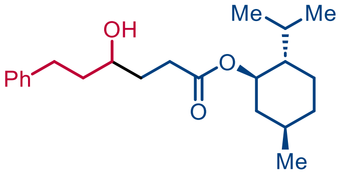


Prepared following **General Procedure B**, using 3-phenylpropanal (26.5 µL, 0.200 mmol) and (−)-menthol derived acrylate **S7** (84.1 mg, 0.400 mmol). Purification by flash column chromatography (silica gel, 10:90 EA/PE) gave **27** (33.2 mg, 48%, 1:1 *dr*) as pale-yellow oil. The *dr* was determined by quantitative $^{13}C$ NMR.

**R$_f$** = 0.29 (10:90 EA/PE, $KMnO_4$).

**NMR Spectroscopy (*see spectra*):**

$^1$**H NMR** (400 MHz, $CDCl_3$): $\delta_H$ 7.30 – 7.25 (m, 2H), 7.23 – 7.15 (m, 3H), 4.73 – 4.63 (m, 1H), 3.70 – 3.60 (m, 1H), 2.84 – 2.75 (m, 1H), 2.73 – 2.63 (m, 1H), 2.48 – 2.38 (m, 2H), 1.99 – 1.63 (m, 9H), 1.52 – 1.43 (m, 1H), 1.40 – 1.31 (m, 1H), 1.05 (qd, *J* = 13.4, 3.8 Hz, 1H), 0.95 (qd, *J* = 12.1, 4.1 Hz, 1H), 0.93 – 0.80 (m, 7H), 0.76 and 0.74 (d, *J* = 1.8 Hz; d, *J* = 1.8 Hz; 3H) ppm.

$^{13}$**C NMR** (101 MHz, $CDCl_3$): $\delta_C$ 173.94, 173.91, 142.1, 128.6, 128.5, 126.0, 74.5, 70.90, 70.85, 47.2, 47.1, 41.1, 41.0, 39.4, 39.3, 34.4, 32.51, 32.49, 32.19, 32.17, 31.5, 31.3, 26.5, 26.4, 23.60, 23.57, 22.2, 20.9, 16.49, 16.46 ppm.

**IR** (film): $v_{max}$ 3444, 2953, 2927, 2869, 1728, 1454, 1264, 1150, 1096 cm$^{-1}$.

**HRMS (ESI$^+$):** calc'd for $[M+Na]^+$, 369.2400; found 369.2394.

### *tert*-Butyl 4-((5-oxotetrahydrofuran-2-yl)methyl)piperidine-1-carboxylate (28)

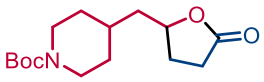


Prepared following **General Procedure B**, using *N*-Boc-4-piperidineacetaldehyde (45.5 mg, 0.200 mmol) and ethyl acrylate (43 µL, 0.40 mmol). Purification by flash column chromatography (silica gel, 40:60 to 50:50 EA/PE) gave **28** (25.6 mg, 45%) as pale-yellow oil..

**R$_f$** = 0.21 (5:95 MeOH/DCM, $KMnO_4$).

**NMR Spectroscopy (*see spectra*):**

$^1$**H NMR** (400 MHz, $CDCl_3$): $\delta_H$ 4.59 (dddd, *J* = 9.2, 8.1, 6.5, 4.0 Hz, 1H), 4.21 – 3.96 (m, 2H), 2.75 – 2.60 (m, 2H), 2.56 – 2.49 (m, 2H), 2.40 – 2.27 (m, 1H), 1.89 – 1.79 (m, 1H), 1.77 – 1.62 (m, 4H), 1.52 – 1.46 (m, 1H), 1.44 (s, 9H), 1.19 – 1.06 (m, 2H) ppm.

$^{13}$**C NMR** (101 MHz, $CDCl_3$): $\delta_C$ 177.2, 155.0, 79.5, 78.5, 43.9, 42.8, 33.1, 32.7, 31.7, 28.84, 28.75, 28.6 ppm.

**IR** (film): $v_{max}$ 2975, 2927, 2854, 1775, 1688, 1422, 1278, 1172, 1152 cm$^{-1}$.

**HRMS (ESI$^+$):** calc'd for $[M+Na]^+$, 306.1676; found 303.1664.

### 4-(2-Hydroxyethyl)-5-phenethyldihydrofuran-2(3*H*)-one (29)

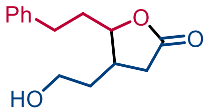


Prepared following **General Procedure B**, using 3-phenylpropanal (26.5 µL, 0.200 mmol) and 5,6-dihydro-pyran-2-one (39.2 mg, 0.400 mmol). Purification by flash column chromatography (silica gel, 5:95 MeOH/DCM) followed by prep TLC (5:95 MeOH/DCM) gave **29** (24.1 mg, 51%, 2:1 *dr)* as pale-yellow oil.

**R$_f$** = 0.20 (5:95 MeOH/DCM, $KMnO_4$).

**NMR Spectroscopy (*see spectra*):**

**$^{1}$H NMR** (400 MHz, $CDCl_3$): $\delta_H$ 7.33 – 7.27 (m, 2H), 7.23 – 7.18 (m, 3H), 4.52 and 4.14 (ddd, $J$ = 10.3, 6.6, 3.7 Hz; ddd, $J$ = 9.0, 7.2, 3.5 Hz; 1H), 3.75 – 3.59 (m, 2H), 2.95 – 2.86 (m, 1H), 2.77 – 2.67 (m, 2H), 2.62 – 2.25 (m, 2H), 2.08 – 1.74 (m, 3H), 1.61 – 1.45 (m, 2H) ppm.

**$^{13}$C NMR** (101 MHz, $CDCl_3$): $\delta_C$ 176.7, 176.6, 141.0, 128.7, 128.6, 126.4, 126.3, 84.9, 82.3, 60.8, 60.7, 38.4, 36.4, 35.7, 35.4, 34.4, 32.13, 32.09, 31.1 ppm.

**IR** (film): $\nu_{max}$ 3444, 2933, 2870, 1770, 1455, 1174, 1053, 1020 cm$^{-1}$.

**HRMS (ESI$^+$):** calc'd for $[M+H]^+$, 235.1329; found 235.1320.

**4-Hydroxy-*N*,6-diphenylhexanamide (30)**

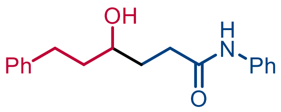


Prepared following **General Procedure B**, using 3-phenylpropanal (26.5 µL, 0.200 mmol) and *N*-phenyl acrylamide (58.9 mg, 0.400 mmol). Purification by flash column chromatography (silica gel, 5:95 MeOH/DCM) followed by prep TLC (5:95 MeOH/DCM) gave **30** (28.4 mg, 50%) as a white solid.

**$R_f$** = 0.21 (5:95 MeOH/DCM, $KMnO_4$).

**NMR Spectroscopy (*see spectra*):**

**$^{1}$H NMR** (400 MHz, $CDCl_3$): $\delta_H$ 7.63 (br.s, 1H), 7.49 (d, $J$ = 8.0 Hz, 2H), 7.33 – 7.27 (m, 4H), 7.20 – 7.17 (m, 3H), 7.10 (t, $J$ = 7.4 Hz, 1H), 3.77 – 3.67 (m, 1H), 2.83 – 2.74 (m, 1H), 2.74 – 2.62 (m, 2H), 2.59 – 2.47 (m, 2H), 1.97 (m, 1H), 1.89 – 1.74 (m, 3H) ppm.

**$^{13}$C NMR** (101 MHz, $CDCl_3$): $\delta_C$ 172.1, 142.0, 137.9, 129.1, 128.59, 128.55, 126.0, 124.5, 120.1, 71.0, 39.6, 34.3, 32.6, 32.2 ppm.

**IR** (film): $\nu_{max}$ 3311, 2927, 2858, 1725, 1664, 1599, 1542, 1498, 1442, 1263, 1096 cm$^{-1}$.

**HRMS (ESI$^+$):** calc'd for $[M+H]^+$, 284.1645; found 284.1639.

***N*-(4-Fluorophenyl)-4-hydroxy-6-phenylhexanamide (31)**

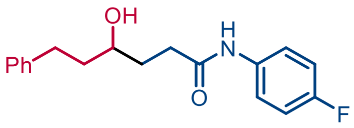


Prepared following **General Procedure B**, using 3-phenylpropanal (26.5 µL, 0.200 mmol) and *N*-(4-fluorophenyl)acrylamide **S8** (66.0 mg, 0.400 mmol). Purification by flash column chromatography (silica gel, 5:95 MeOH/DCM) followed by prep TLC (5:95 MeOH/DCM) gave **31** (25.6 mg, 42%) as a white solid.

**$R_f$** = 0.19 (5:95 MeOH/DCM, $KMnO_4$).

**NMR Spectroscopy (*see spectra*):**

**$^{1}$H NMR** (400 MHz, $CDCl_3$): $\delta_H$ 7.54 (br. s, 1H), 7.47 – 7.41 (m, 2H), 7.30 – 7.27 (m, 2H), 7.22 – 7.16 (m, 3H), 7.03 – 6.96 (m, 2H), 3.77 – 3.68 (m, 1H), 2.84 – 2.75 (m, 1H), 2.74 – 2.67 (m, 1H), 2.59 – 2.46 (m, 2H), 2.45 (d, *J* = 4.8 Hz, 1H), 2.02 – 1.91 (m, 1H), 1.86 – 1.75 (m, 3H) ppm.

**$^{13}$C NMR** (101 MHz, $CDCl_3$): $\delta_C$ 171.9, 159.5 (d, *J* = 244 Hz), 141.9, 133.9, 128.62, 128.55, 126.1, 121.9 (d, *J* = 8 Hz), 115.8 (d, *J* = 23 Hz), 71.1, 39.6, 34.2, 32.5, 32.2 ppm.

**$^{19}$F NMR** (377 MHz, not $^{19}$F{$^{1}$H} decoupled, $CDCl_3$): $\delta_F$ −117.93 (tt, *J* = 8.5, 5.3 Hz) ppm.

**IR** (film): $v_{max}$ 3299, 2925, 2860, 1739, 1660, 1615, 1547, 1508, 1407, 1214, 1085 cm$^{-1}$.

**HRMS (ESI$^+$):** calc'd for [M+H]$^+$, 302.1551; found 302.1540.

**4-Hydroxy-6-phenyl-*N*-(4-(trifluoromethyl)phenyl)hexanamide (32)**

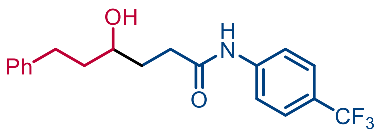


Prepared following **General Procedure B**, using 3-phenylpropanal (26.5 µL, 0.200 mmol) and *N*-(4-(trifluoromethyl)phenyl)acrylamide **S9** (86.1 mg, 0.400 mmol). Purification by flash column chromatography (silica gel, 5:95 MeOH/DCM) followed by prep TLC (5:95 MeOH/DCM) gave **32** (25.2 mg, 36%) as a white solid.

**$R_f$** = 0.20 (5:95 MeOH/DCM, $KMnO_4$).

**NMR Spectroscopy (*see spectra*):**

**$^{1}$H NMR** (400 MHz, $CD_3CN$): $\delta_H$ 8.62 (br.s, 1H), 7.77 – 7.71 (m, 2H), 7.61 – 7.58 (m, 2H), 7.29 – 7.25 (m, 2H), 7.24 – 7.20 (m, 2H), 7.19 – 7.14 (m, 1H), 3.61 – 3.51 (m, 1H), 2.95 (d, *J* = 5.3 Hz, 1H), 2.76 (ddd, *J* = 13.6, 10.0, 5.6 Hz, 1H), 2.64 (ddd, *J* = 13.6, 9.9, 6.6 Hz, 1H), 2.53 – 2.37 (m, 2H), 1.86 (dddd, *J* = 13.9, 8.2, 7.1, 3.8 Hz, 1H), 1.79 – 1.63 (m, 3H) ppm.

**$^{13}$C NMR** (101 MHz, $CD_3CN$): $\delta_C$ 173.4, 143.7, 143.6, 129.4, 129.3, 127.0 (q, *J* = 3.8 Hz), 126.6, 125.6 (q, *J* = 270.6 Hz), 125.2 (q, *J* = 32.6 Hz), 120.0, 70.7, 40.2, 34.3, 33.4, 32.7 ppm.

**$^{19}$F NMR** (377 MHz, $CD_3CN$): $\delta_F$ -62.43 ppm.

**IR** (film): $v_{max}$ 3316, 2928, 2858, 1738, 1670, 1604, 1534, 1409, 1366, 1324, 1217, 1113, 1068 cm$^{-1}$.

**HRMS (ESI$^+$):** calc'd for [M+H]$^+$, 352.1519; found 352.1513.

**4-Hydroxy-*N*,*N*,6-triphenylhexanamide (33)**

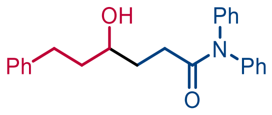


Prepared following **General Procedure B**, using 3-phenylpropanal (26.5 µL, 0.200 mmol) and *N*,*N*-diphenylacrylamide **S10** (89.3 mg, 0.400 mmol). Purification by flash column chromatography (silica gel, 40:60 EA/PE) followed by prep TLC (5:95 MeOH/DCM) gave **33** (35.2 mg, 49%) as a colourless oil.

**$R_f$** = 0.36 (5:95 MeOH/DCM, $KMnO_4$).

**NMR Spectroscopy (*see spectra*):**

> **$^1$H NMR** (400 MHz, $CDCl_3$): $\delta_H$ 7.46 – 7.21 (m, 12H), 7.20 – 7.16 (m, 3H), 3.64 (tq, *J* = 8.2, 4.2 Hz, 1H), 2.78 (m, 2H), 2.66 (ddd, *J* = 13.8, 9.6, 6.7 Hz, 1H), 2.52 – 2.36 (m, 2H), 1.91 – 1.66 (m, 4H) ppm.
>
> **$^{13}$C NMR** (101 MHz, $CDCl_3$): $\delta_C$ 174.2, 142.8, 142.4, 130.0, 129.2, 128.6, 128.5, 128.1, 126.6, 125.9, 71.0, 39.6, 32.6, 32.3, 32.2 ppm.

**IR** (film): $\nu_{max}$ 3427, 3061, 3026, 2925, 2855, 1726, 1668, 1594, 1492, 1452, 1380, 1273, 1075 cm$^{-1}$.

**HRMS (ESI$^+$):** calc'd for $[M+H]^+$, 360.1958; found 360.1950.

**4-Hydroxy-*N*-methyl-*N*,6-diphenylhexanamide (34)**

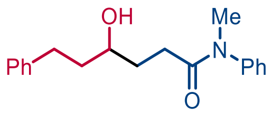


Prepared following **General Procedure B**, using 3-phenylpropanal (26.5 µL, 0.200 mmol) and *N*-methyl-*N*-phenyl acrylamide **S11** (64.5 mg, 0.400 mmol). Purification by flash column chromatography (silica gel, 40:60 EA/PE to 5:95 MeOH/DCM) followed by prep TLC (5:95 MeOH/DCM) gave **34** (19.2 mg, 32%) as a colourless oil.

**$R_f$** = 0.19 (5:95 MeOH/DCM, $KMnO_4$).

**NMR Spectroscopy (*see spectra*):**

> **$^1$H NMR** (400 MHz, $CDCl_3$): $\delta_H$ 7.46 – 7.39 (m, 2H), 7.35 (t, *J* = 7.3 Hz, 1H), 7.29 – 7.23 (m, 2H), 7.21 – 7.12 (m, 5H), 3.57 (tt, *J* = 8.1, 4.0 Hz, 1H), 3.27 (s, 3H), 2.77 (ddd, *J* = 13.7, 9.8, 5.6 Hz, 1H), 2.64 (ddd, *J* = 13.7, 9.7, 6.7 Hz, 1H), 2.34 – 2.17 (m, 2H), 1.96 – 1.57 (m, 5H) ppm.
>
> **$^{13}$C NMR** (101 MHz, $CDCl_3$): $\delta_C$ 174.1, 144.0, 142.4, 130.0, 128.6, 128.5, 128.1, 127.4, 125.8, 71.1, 39.6, 37.7, 32.5, 32.2, 31.4 ppm.

**IR** (film): $\nu_{max}$ 3418, 2924, 2858, 1737, 1638, 1594, 1496, 1453, 1388, 1124 cm$^{-1}$.

**HRMS (ESI$^+$):** calc'd for $[M+H]^+$, 298.1802; found 298.1796.

**tert-Butyl 4-(2-hydroxy-4-(4-(methylsulfonyl)phenyl)butyl)piperidine-1-carboxylate (36)**

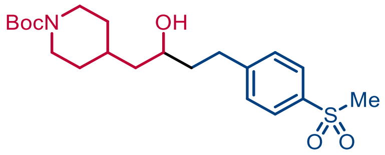


Prepared following **General Procedure B**, using *N*-Boc-4-piperidineacetaldehyde (45.5 mg, 0.200 mmol) and 1-(methylsulfonyl)-4-vinylbenzene **S12** (72.9 mg, 0.400 mmol). Purification by flash column chromatography (silica gel, 1:20 MeOH/DCM) followed by prep TLC (1:20 MeOH/DCM) gave **36** (42.9 mg, 52%) as a colourless oil.

**R$_f$** = 0.26 (1:20 MeOH/DCM, $KMnO_4$).

**NMR Spectroscopy (*see spectra*):**

**$^1$H NMR** (400 MHz, $CDCl_3$): $\delta_H$ 7.87 – 7.82 (m, 2H), 7.41 – 7.36 (m, 2H), 4.19 – 3.92 (m, 2H), 3.77 – 3.68 (m, 1H), 3.03 (s, 3H), 2.92 – 2.85 (m, 1H), 2.80 – 2.73 (ddd, *J* = 13.8, 9.7, 6.8 Hz, 1H), 2.73 – 2.60 (m, 2H), 1.83 – 1.57 (m, 6H), 1.48 – 1.42 (m, 1H), 1.44 (s, 9H), 1.37 – 1.29 (m, 1H), 1.14 (qd, *J* = 12.3, 4.2 Hz, 1H), 1.04 (qd, *J* = 12.2, 4.2 Hz, 1H) ppm.

**$^{13}$C NMR** (101 MHz, $CDCl_3$): $\delta_C$ 155.0, 149.0, 138.3, 129.5, 127.7, 79.4, 68.3, 44.7, 44.6, 43.9, 39.5, 33.1, 32.7, 32.1, 31.8, 28.6 ppm.

**IR** (film): $\nu_{max}$ 3457, 2926, 2850, 1726, 1686, 1424, 1366, 1304, 1279, 1247, 1149, 1090 cm$^{-1}$.

**HRMS (ESI$^+$):** calc'd for [M+Na]$^+$, 434.1972; found 434.1968.

**tert-Butyl 4-(4-(4-cyanophenyl)-2-hydroxybutyl)piperidine-1-carboxylate (37)**

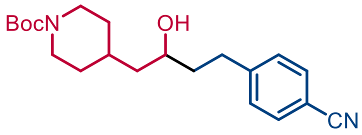


Prepared following **General Procedure B**, using *N*-Boc-4-piperidineacetaldehyde (45.5 mg, 0.200 mmol) and 4-cyanostyrene (51.7 mg, 0.400 mmol). Purification by flash column chromatography (silica gel, 1:20 MeOH/DCM) followed by prep TLC (1:20 MeOH/DCM) gave **37** (43.6 mg, 61%) as pale-yellow oil.

**R$_f$** = 0.14 (1:20 MeOH/DCM, $KMnO_4$).

**NMR Spectroscopy (*see spectra*):**

**$^1$H NMR** (400 MHz, $CDCl_3$): $\delta_H$ 7.59 – 7.55 (m, 2H), 7.32 – 7.28 (m, 2H), 4.24 – 3.88 (m, 2H), 3.74 – 3.68 (m, 1H), 2.86 (ddd, *J* = 13.9, 9.7, 5.8 Hz, 1H), 2.73 (ddd, *J* = 13.9, 9.7, 6.8 Hz, 1H), 2.72 – 2.61 (m, 2H), 1.81 – 1.57 (m, 5H), 1.48 – 1.43 (m, 1H), 1.44 (s, 9H), 1.37 – 1.29 (m, 1H), 1.14 (qd, *J* = 12.3, 4.3 Hz, 1H), 1.04 (qd, *J* = 12.3, 4.2 Hz, 1H) ppm.

**$^{13}$C NMR** (101 MHz, $CDCl_3$): $\delta_C$ 155.0, 147.9, 132.4, 129.3, 119.2, 109.9, 79.5, 68.4, 44.7, 44.3, 39.4, 33.1, 32.7, 32.3, 31.8, 28.6 ppm.

**IR** (film): $\nu_{max}$ 3448, 2975, 2924, 2854, 2226, 1727, 1686, 1666, 1365, 1278, 1246, 1162, 1090 cm$^{-1}$.

**HRMS (ESI$^+$):** calc'd for $[M+Na]^+$, 381.2149; found 381.2136.

***tert*-Butyl 4-(4-(4-carbamoylphenyl)-2-hydroxybutyl)piperidine-1-carboxylate (38)**

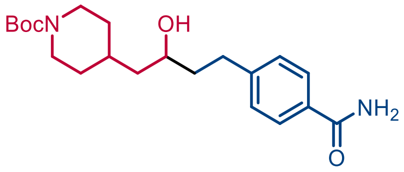


Prepared following **General Procedure B**, using *N*-Boc-4-piperidineacetaldehyde (45.5 mg, 0.200 mmol) and 4-vinylbenzamide **S13** (58.9 mg, 0.400 mmol). Purification by flash column chromatography (silica gel, 5:95 MeOH/DCM) followed by prep TLC (5:95 MeOH/DCM) gave **38** (15.7 mg, 21%) as a white solid. The yield of unreacted aldehyde (35%) was determined by $^1$H NMR analysis of the crude product.

**R$_f$** = 0.14 (5:95 MeOH/DCM, $KMnO_4$).

**NMR Spectroscopy (*see spectra*):**

**$^1$H NMR** (400 MHz, $CDCl_3$): $\delta_H$ 7.76 – 7.71 (m, 2H), 7.29 – 7.26 (m, 2H), 4.23 – 3.90 (m, 2H), 3.77 – 3.64 (m, 1H), 2.84 (ddd, *J* = 14.9, 9.4, 6.1 Hz, 1H), 2.76 – 2.59 (m, 2H), 2.73 (ddd, *J* = 14.9, 9.4, 6.8 Hz, 1H), 1.82 – 1.56 (m, 6H), 1.49 – 1.44 (m, 1H), 1.45 (s, 9H), 1.33 (ddd, *J* = 14.1, 8.6, 3.9 Hz, 1H), 1.14 (qd, *J* = 12.2, 3.3 Hz, 1H), 1.03 (qd, *J* = 12.2, 3.6 Hz, 1H) ppm.

**$^{13}$C NMR** (101 MHz, $CDCl_3$): $\delta_C$ 169.3, 155.0, 146.7, 131.1, 128.8, 127.7, 79.4, 68.5, 44.6, 44.1, 39.6, 33.1, 32.7, 32.0, 31.8, 28.6 ppm.

**IR** (film): $\nu_{max}$ 3411, 3357, 3186, 2971, 2923, 2850, 1725, 1672, 1612, 1417, 1365, 1279, 1247, 1172 cm$^{-1}$.

**HRMS (ESI$^+$):** calc'd for $[M+H]^+$, 377.2435; found 377.2437.

***tert*-Butyl 4-(2-hydroxy-2-(3-oxocyclopentyl)ethyl)piperidine-1-carboxylate (39)**

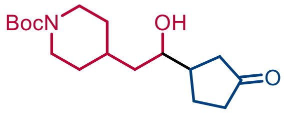


Prepared following **General Procedure B**, using *N*-Boc-4-piperidineacetaldehyde (45.5 mg, 0.200 mmol) and cyclopent-2-enone (32.8 mg, 0.400 mmol). Purification by flash column chromatography (silica gel, 5:95 MeOH/DCM) followed by prep TLC (5:95 MeOH/DCM) gave **39** (30.1 mg, 48%, 1.3:1 *dr*) as a yellow oil. The *dr* was determined by quantitative $^{13}$C NMR.

**$R_f$** = 0.29 (5:95 MeOH/DCM, $KMnO_4$).

**NMR Spectroscopy (*see spectra*):**

**$^1$H NMR** (400 MHz, $CDCl_3$): (*The signals of the two diastereomers were overlapping and indistinguishable)* $\delta_H$ 4.20 – 3.93 (m, 2H), 3.75 – 3.61 (m, 1H), 2.80 – 2.58 (m, 2H), 2.40 – 1.95 (m, 6H), 1.80 – 1.58 (m, 5H), 1.50 – 1.39 (m, 1H), 1.44 (s, 9H), 1.38 – 1.23 (m, 1H), 1.17 (qd, *J* = 12.3, 4.2 Hz, 1H), 1.04 (m, 1H) ppm.

**$^{13}$C NMR** (101 MHz, $CDCl_3$): $\delta_C$ 219.1, 218.9, 155.0, 79.5, 71.6, 71.5, 44.2, 43.6, 43.5, 42.83, 42.78, 41.9, 40.5, 38.7, 38.6, 33.3, 32.62, 32.55, 31.6, 28.6, 26.1, 24.7 ppm.

**IR** (film): $\nu_{max}$ 3456, 2972, 2922, 2854, 1740, 1689, 1668, 1426, 1365, 1279, 1245, 1167, 1133 cm$^{-1}$.

**HRMS (ESI$^+$):** calc'd for $[M+Na]^+$, 334.1989; found 334.1987.

### ***tert*-Butyl 4-(4-cyano-2-hydroxybutyl)piperidine-1-carboxylate (40)**

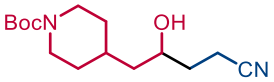


Prepared following **General Procedure B**, using *N*-Boc-4-piperidineacetaldehyde (45.5 mg, 0.200 mmol) and acrylonitrile (21.2 mg, 0.400 mmol). Purification by flash column chromatography (silica gel, 1:20 MeOH/DCM) followed by prep TLC (1:20 MeOH/DCM) gave **40** (10.1 mg, 18%) as pale-yellow oil. The yield of unreacted aldehyde (45%) was determined by $^1$H NMR analysis of the crude product.

**$R_f$** = 0.24 (1:20 MeOH/DCM, $KMnO_4$).

**NMR Spectroscopy (*see spectra*):**

**$^1$H NMR** (400 MHz, $CDCl_3$): $\delta_H$ 4.19 – 4.00 (m, 2H), 3.89 – 3.80 (m, 1H), 2.79 – 2,59 (m, 2H), 2.56 – 2.46 (m, 2H), 1.83 (dtd, *J* = 14.1, 7.9, 3.2 Hz, 1H), 1.77 – 1.61 (m, 4H), 1.56 (d, *J* = 5.7 Hz, 1H), 1.49 – 1.46 (m, 1H), 1.45 (s, 9H), 1.34 (ddd, *J* = 13.9, 8.3, 4.1 Hz, 1H), 1.18 (qd, *J* = 12.2, 4.2 Hz, 1H), 1.08 (qd, *J* = 12.2, 4.2 Hz, 1H) ppm.

**$^{13}$C NMR** (101 MHz, $CDCl_3$): $\delta_C$ 155.0, 119.9, 79.5, 67.4, 44.4, 43.7, 33.3, 32.9, 32.7, 31.8, 28.6, 13.8 ppm.

**IR** (film): $\nu_{max}$ 3445, 2974, 2928, 2850, 2246, 1726, 1690, 1667, 1426, 1366, 1280, 1248, 1167, 1135 cm$^{-1}$.

**HRMS (ESI$^+$):** calc'd for $[M+Na]^+$, 305.1836; found 305.1827.

### 2.4.3. Unsuccessful and low yielding substrates

No desired product or low yields were observed for the following substrates. Yields were determined by $^{1}$H NMR analysis using an internal standard.

***Aldehydes and ketones*** *(reaction with tert-butyl acrylate **3**):*

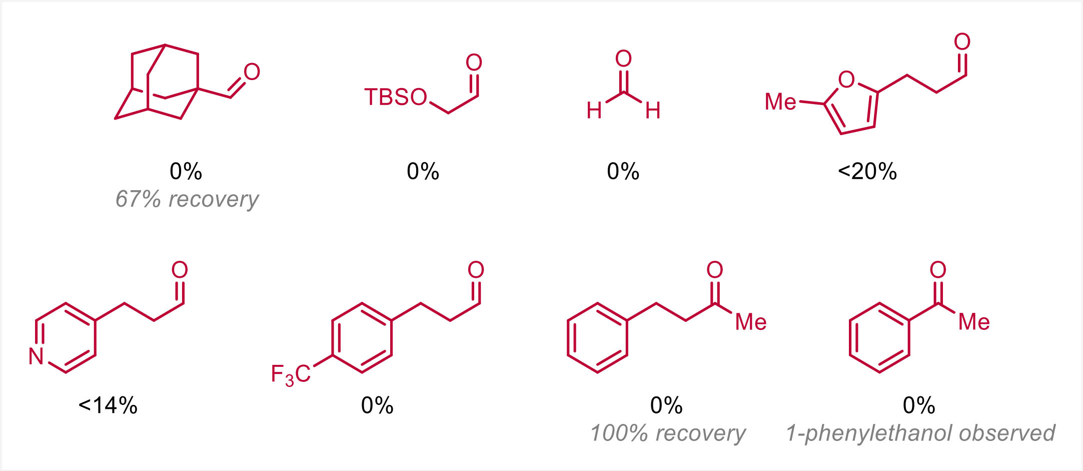


***Olefins*** *(reaction with 3-phenylpropanal **2**):*

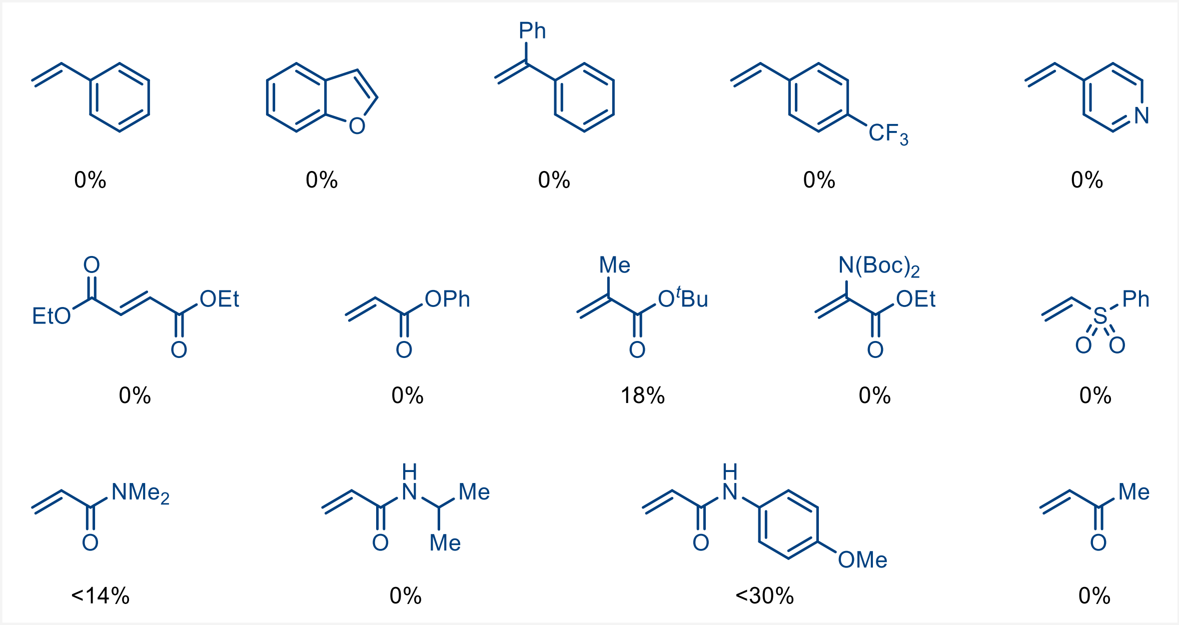

<u>Mass balance of selected failed olefin substrates</u>

**Procedure:** Following **General Procedure B** using 3-phenylpropanal **2** (26.5 μL, 26.8 mg, 0.200 mmol, 1.00 equiv). The reaction mixture was diluted with $Et_2O$ (30 mL), washed with $H_2O$ (2 × 10 mL). The organic fractions were combined, dried with $MgSO_4$ and filtered. The dried organic solution was then analysed by GC-MS using 1,4-dioxane as an internal standard to identify volatile compounds. Subsequently, the organic solution was concentrated *in vacuo* and analysed by $^1H$ NMR using $CH_2Br_2$ as an internal standard. *The yields of products were calculated relative to 0.2 mmol.* In all these cases, no alcohol products formed from aldehyde–olefin cross-coupled were detected.

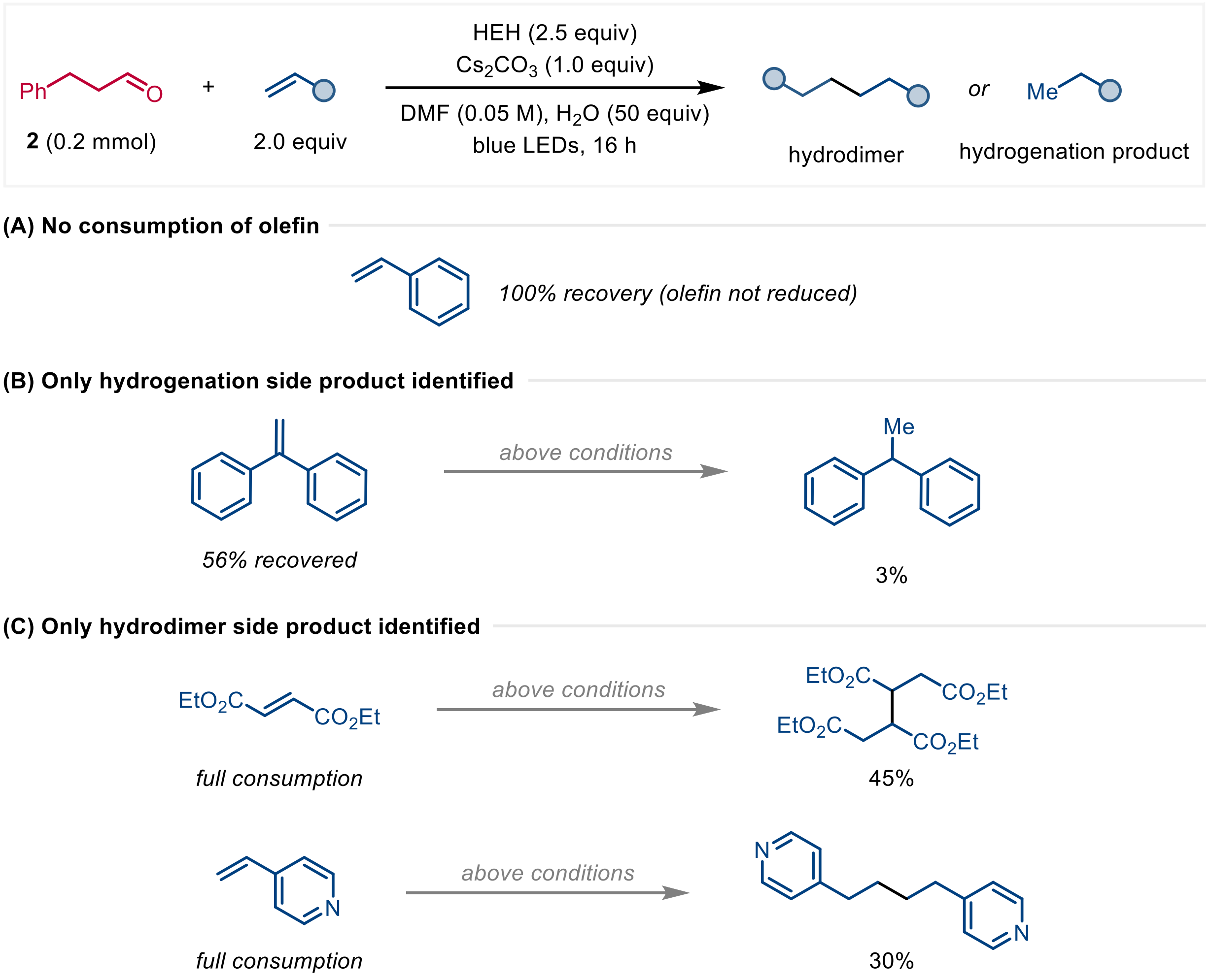


**Comments:**

1) Unsubstituted styrene was unreactive and quantitatively recovered. This is because of its more negative reduction potential ($E_{p/2}$ = −2.58 V vs. SCE)[17] compared to *tert*-butyl acrylate ($E_{p/2}$ = −2.26 V vs. SCE).

2) 1,1-Diphenylethylene reacted slowly due to its less negative reduction potential ($E_{p/2}$ = −2.38 V vs. SCE)[18] compared to unsubstituted styrene, with only 56% recovered. The hydrogenated side product was identified, albeit in a low yield of 3%. No hydrodimer product was observed.

3) Diethyl fumarate and 4-vinyl pyridine were fully consumed and underwent reductive homocouplings to give the hydrodimer products in 45% and 30% yields, respectively. However, no hydrogenation products were detected.

### 2.4.4. Scale-up reaction

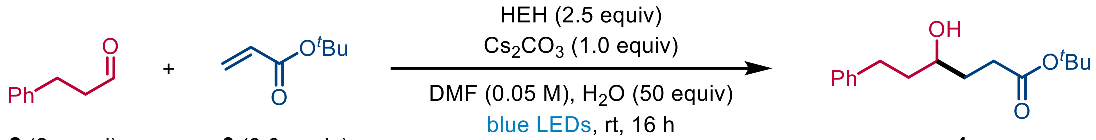


**2** (2 mmol) **3** (3.0 equiv) **4**

A 100 mL round bottom flask was charged with $Cs_2CO_3$ (650 mg, 2.0 mmol, 1.0 equiv) and Hantzsch ester (HEH, 1.27 g, 5.0 mmol, 2.5 equiv) before anhydrous DMF (40 mL) and $H_2O$ (1.8 mL, 100 mmol, 50 equiv) were added to the flask. 3-Phenylpropanal (268 mg, 2.0 mmol, 1.0 equiv) and *tert*-butyl acrylate (880 μL, 770 mg, 6.0 mmol, 3.0 equiv) were then added to the solution. Subsequently, the solution was degassed by sparging with $N_2$ using a balloon for 5 min. The mixture was then irradiated with two 40 W Kessil A160WE Tuna Blue LED lamps (color dial turned fully anticlockwise, and the intensity dial turned fully clockwise) at room temperature for 16 h (see **Figure S2** for photochemical reaction set-up). The reaction mixture was then diluted with $Et_2O$ (300 mL), washed with $H_2O$ (2 × 100 mL), dried with $MgSO_4$, filtered, and concentrated in vacuo to give the crude product, which was purified by flash column chromatography (silica gel, 20:80 EA/PE) to give product **4** (243 mg, 46%) as a colourless oil.

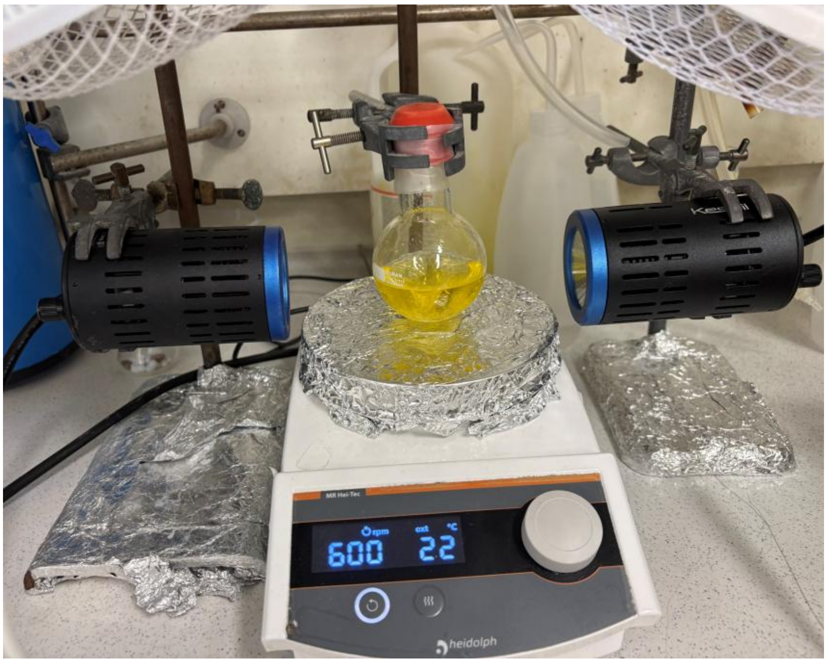


**Figure S2.** Photochemical reaction setup (2 mmol scale)

### 2.4.5. Hydrodimerization of *tert*-butyl acrylate

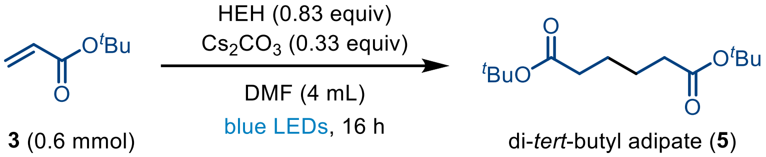


**3** (0.6 mmol) di-*tert*-butyl adipate (**5**)

**Procedure:** A 7 mL vial was charged with $Cs_2CO_3$ (65 mg, 0.20 mmol, 0.33 equiv), Hantzsch ester (HEH, 127 mg, 0.500 mmol, 0.833 equiv). Anhydrous DMF (4.0 mL) was then added to the vial. Subsequently, the solution was degassed by sparging with $N_2$ using a balloon for 1 min before adding *tert*-butyl acrylate **3** (88 μL, 0.60 mmol, 1.0 equiv). The mixture was then irradiated with blue LEDs (see Figure S1) at room temperature for 16 h. The reaction mixture was then diluted with $Et_2O$ (30 mL), washed with $H_2O$ (3 × 10 mL), dried with $MgSO_4$, filtered, and concentrated *in vacuo* to give the crude product. Purification by flash column chromatography (silica gel, 10:90 EA/PE) gave di-*tert*-butyl adipate **5** (56.9 mg, 73%) as a pale-yellow oil.

**$R_f$** = 0.50 (10:90 EA/PE, $KMnO_4$).

**NMR Spectroscopy (*see spectra*):**

**$^1$H NMR** (400 MHz, $CDCl_3$): $\delta_H$ 2.25 – 2.18 (m, 4H), 1.63 – 1.57 (m, 4H), 1.43 (s, 18H) ppm.

**$^{13}$C NMR** (101 MHz, $CDCl_3$): $\delta_C$ 173.0, 80.2, 35.4, 28.2, 24.7 ppm.

All recorded spectroscopic data matched those previously reported in the literature.[12]

# 3. MECHANISTIC STUDIES

## 3.1. Control Experiments

### 3.1.1. Aldehyde experiments

**Procedure** (*without olefin*): A 7 mL vial was charged with $Cs_2CO_3$ (65 mg, 0.20 mmol, 1.0 equiv) and Hantzsch ester (HEH, 127 mg, 0.500 mmol, 2.50 equiv). Anhydrous DMF (4.0 mL) and $H_2O$ (180 µL, 10.0 mmol, 50.0 equiv) were then added to the vial. Subsequently, the solution was degassed by sparging with $N_2$ using a balloon for 1 min before addition of 3-phenylpropanal **2** (26.5 µL, 26.8 mg, 0.200 mmol, 1.00 equiv). The mixture was then irradiated with blue LEDs (see Figure S1) at room temperature for 16 h. The **General workup and analysis procedure** was then followed.

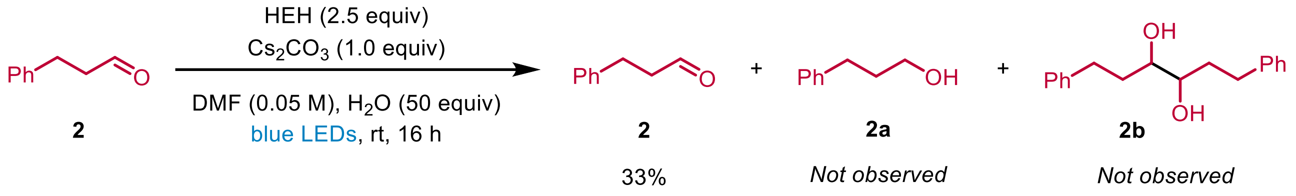


### 3.1.2. Olefin experiments

**Procedure** (*without aldehyde*): A 7 mL vial was charged with $Cs_2CO_3$ (65 mg, 0.20 mmol) and Hantzsch ester (HEH, 127 mg, 0.500 mmol). Anhydrous DMF (4.0 mL) and $H_2O$ (180 µL, 10.0 mmol) were then added to the vial. Subsequently, the solution was degassed by sparging with $N_2$ using a balloon for 1 min before addition of *tert*-butyl acrylate **3** (88 µL, 77 mg, 0.60 mmol). The mixture was then irradiated with blue LEDs (see Figure S1) at room temperature for 16 h. The **General workup and analysis procedure** was then followed. *Note:* The yield of **5** was calculated relative to 0.3 mmol.

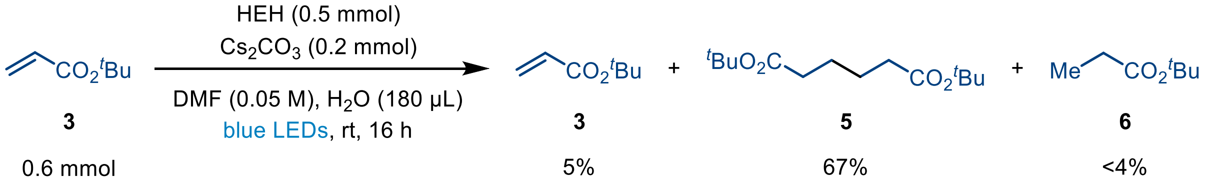

### 3.1.3. Other reaction component experiments

**Procedure** *Standard Conditions*: A 7 mL vial was charged with $Cs_2CO_3$ (65 mg, 0.20 mmol, 1.0 equiv) and Hantzsch ester (HEH, 127 mg, 0.500 mmol, 2.50 equiv). Anhydrous DMF (4.0 mL) and $H_2O$ (180 µL, 10.0 mmol, 50.0 equiv) were then added to the vial. Subsequently, the solution was degassed by sparging with $N_2$ using a balloon for 1 min before addition of 3-phenylpropanal **2** (26.5 µL, 26.8 mg, 0.200 mmol, 1.00 equiv) and *tert*-butyl acrylate **3** (88 µL, 77 mg, 0.60 mmol, 3.0 equiv). The mixture was then irradiated with blue LEDs (see Figure S1) at room temperature for 16 h. The variations shown in Table S10 were made based on these standard conditions. The **General workup and analysis procedure** was then followed.

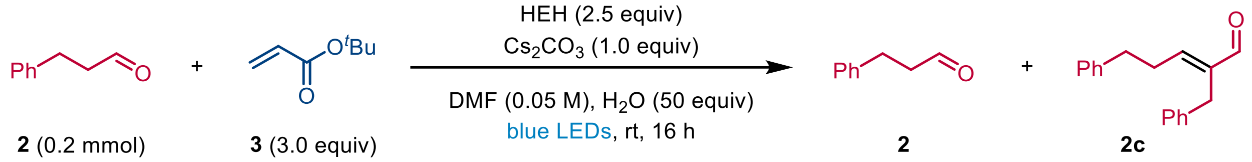


| Entry | Variation from standard conditions | Recovered aldehyde **2**[a] | Aldol product **2c**[b] |
|---|---|---|---|
| 1 | None | <2% | 0% |
| 2 | No light | 26% | 30% |
| 3 | No HEH | 29% | 28% |
| 4 | No acrylate | 33% | 0% |
| 5 | No base | 89% | 0% |

[a] Yield of recovered **2** was determined by GC-MS. [b] Yield of aldol product **2c** was determined relative to 0.1 mmol by $^1H$ NMR analysis.

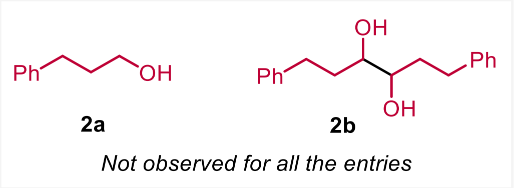


**Table S10.** Other reaction component experiments

**Comments:**

1) Partial consumption of the aldehyde **2** (ca. 70%) was observed without light, HEH or acrylate (entries 2-4). The aldol product **2c** was observed in approximately 30% yield without light or HEH (entries 2-3). Aldol product **2c** was likely formed in the absence of acrylate (entry 4), but was unstable under the reducing conditions and therefore was not detected in the crude product mixture.

2) Aldehyde **2** was relatively stable in the absence of base (entry 5) and 89% of it was recovered after reaction.

3) No evidence of aldehyde reduction was found, as the reduction products – primary alcohol **2a** or vicinal diol **2b** – from the aldehyde **2** were not observed.

## 3.2. Radical Clock Experiments

### 3.2.1. Experiment using *tert*-butyl acrylate

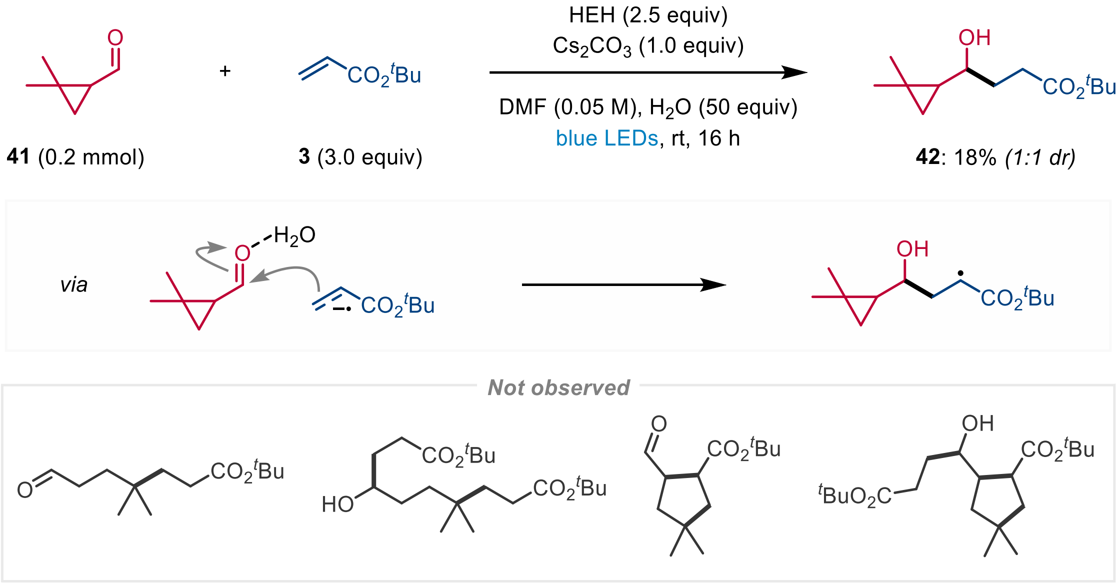


Procedure: **General Procedure A** was followed, using 2,2-dimethylcyclopropanaldehyde **41** (19.6 mg, 0.200 mmol). Purification by flash column chromatography (silica gel, 20:80 EA/PE) followed by prep HPLC gave **42** (8.1 mg, 18%, 1:1 *dr*) as a colourless oil.

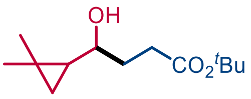

**42**

**R$_f$** = 0.39 and 0.46 (20:80 EA/PE, $KMnO_4$).

**NMR Spectroscopy:**

*Diastereomer 1* (***see spectra***)

**$^1$H NMR** (400 MHz, $CDCl_3$): $\delta_H$ 3.29 – 3.17 (m, 1H), 2.38 (t, *J* = 7.3 Hz, 2H), 1.97 – 1.77 (m, 2H), 1.70 (d, *J* = 2.9 Hz, 1H), 1.44 (s, 9H), 1.17 (s, 3H), 1.07 (s, 3H), 0.69 (td, *J* = 8.8, 5.3 Hz, 1H), 0.49 (dd, *J* = 8.8, 4.5 Hz, 1H), 0.10 (t, *J* = 4.9 Hz, 1H) ppm.

**$^{13}$C NMR** (101 MHz, $CDCl_3$): $\delta_C$ 173.6, 80.4, 73.6, 32.6, 32.3, 31.3, 28.3, 27.5, 20.2, 18.6, 15.9 ppm.

*Diastereomer 2* (***see spectra***)

**$^1$H NMR** (400 MHz, $CDCl_3$): $\delta_H$ 3.25 – 3.15 (m, 1H), 2.38 (td, *J* = 7.5, 3.7 Hz, 2H), 1.84 (q, *J* = 7.1 Hz, 2H), 1.69 (d, *J* = 3.0 Hz, 1H), 1.45 (s, 9H), 1.07 (s, 3H), 1.04 (s, 3H), 0.66 (td, *J* = 8.8, 5.3 Hz, 1H), 0.51 (dd, *J* = 8.8, 4.4 Hz, 1H), 0.25 (t, *J* = 5.0 Hz, 1H) ppm.

**$^{13}$C NMR** (101 MHz, $CDCl_3$): $\delta_C$ 173.6, 80.4, 72.8, 33.2, 32.0, 31.6, 28.3, 27.5, 20.7, 18.5, 16.8 ppm.

**IR** (film): $\nu_{max}$ 3433, 2977, 2931, 2865, 1729, 1367, 1256, 1151 cm$^{-1}$.

**HRMS (ESI$^+$):** calc'd for $[M+Na]^+$, 251.1623; found 251.1614.

### 3.2.2. Experiment using styrene

The unsubstituted styrene was also tested, which precluded the olefin radical anion pathway since unsubstituted styrene could not be reduced under our conditions (see Section 2.4.3).

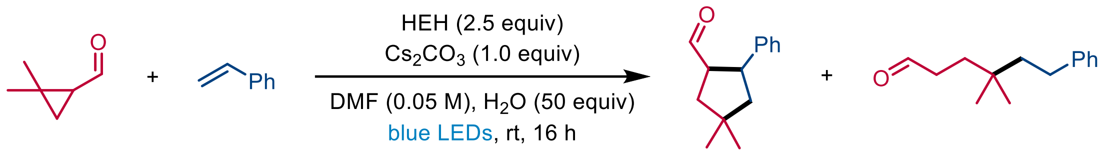


**41** (0.2 mmol) **43** (1 equiv) **44**: 2% **45**: 3%

*only ring-opened products were observed*

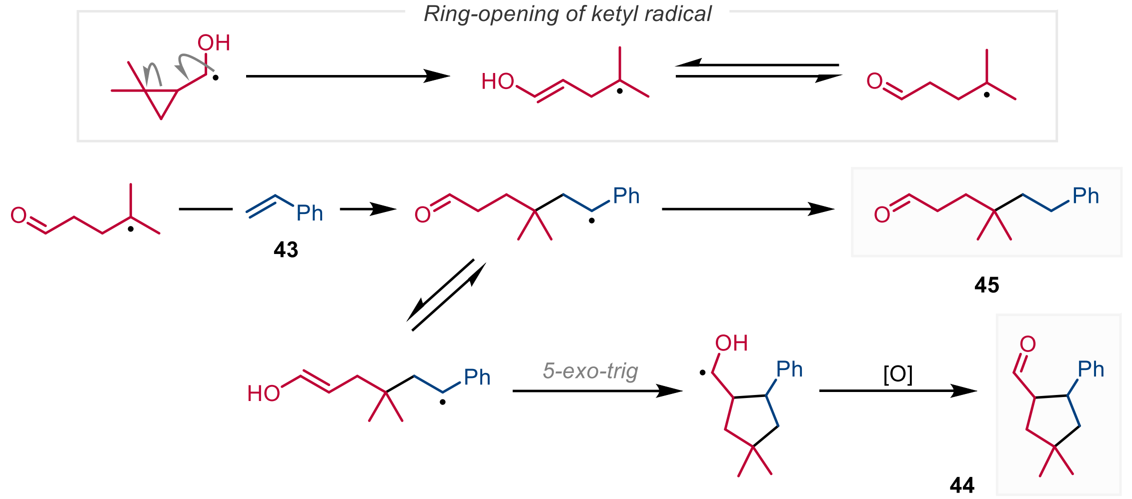


**¹H NMR spectrum** of the rearranged products after silica gel column chromatography ($CDCl_3$):

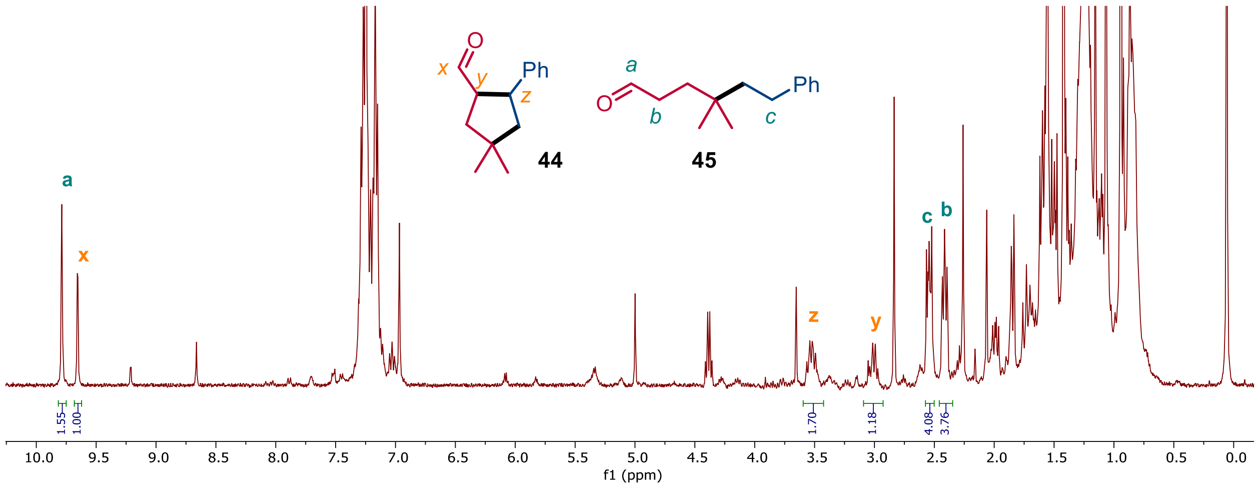


**Comments:**

Low yields of the ring-opened products were detected. This suggests that the ketyl radical was generated and led to the ring-opened products via a ketyl radical mechanism. However, this ketyl radical pathway was rather inefficient.

## 3.3. Ketone Experiments

### 3.3.1. Experiment using 1 equivalent of ketone

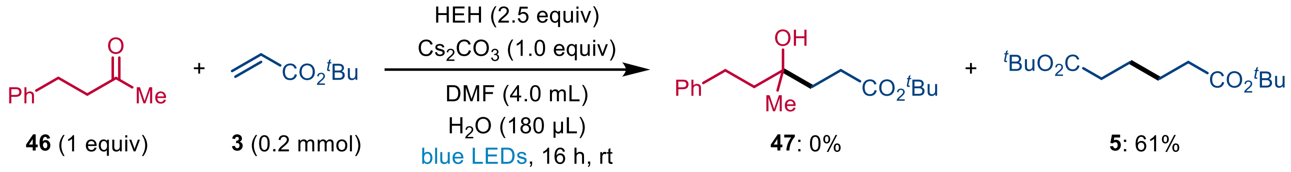


**46** (1 equiv) (96% recovery) **3** (0.2 mmol) **47**: 0% **5**: 61%

**Procedure: General Procedure A** was followed. The yields of **47** and recovered **5** were determined by $^{1}$H NMR analysis using $CH_2Br_2$ as an internal standard.

### 3.3.2. Experiment using 50 equivalents of ketone

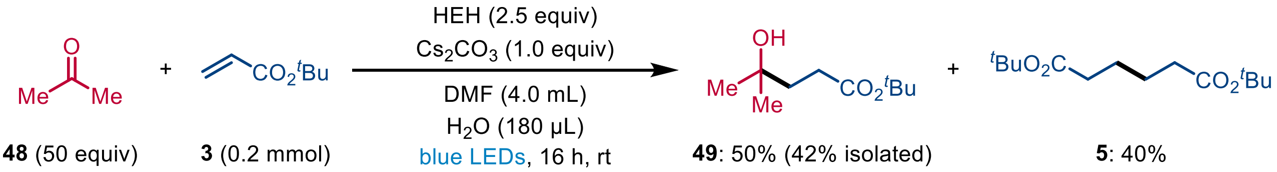


**48** (50 equiv) **3** (0.2 mmol) **49**: 50% (42% isolated) **5**: 40%

**Procedure:** A 7 mL vial was charged with $Cs_2CO_3$ (65 mg, 0.20 mmol, 1.0 equiv), Hantzsch ester (HEH, 127 mg, 0.500 mmol, 2.50 equiv). Anhydrous DMF (4.0 mL) and $H_2O$ (180 µL, 10.0 mmol, 50.0 equiv) were then added to the vial. Subsequently, the solution was degassed by sparging with $N_2$ using a balloon for 1 min before adding acetone **48** (740 µL, 580 mg, 10.0 mmol, 50.0 equiv) and *tert*-butyl acrylate **3** (29 µL, 0.20 mmol, 1.0 equiv). The mixture was then irradiated with blue LEDs (see Figure S1) at room temperature for 16 h. The reaction mixture was then diluted with $Et_2O$ (30 mL), washed with $H_2O$ (2 × 10 mL), dried with $MgSO_4$, filtered, and concentrated *in vacuo*. The yield of alcohol **49** and adipate **5** were determined by $^{1}$H NMR analysis using $CH_2Br_2$ as an internal standard. Purification by flash column chromatography (silica gel, 20:80 EA/PE) followed by prep HPLC gave **49** (15.9 mg, 42%) as a pale-yellow oil.

**R$_f$** = 0.25 (EA/PE, $KMnO_4$).

**NMR Spectroscopy: (*see spectra*)**

$^{1}$**H NMR** (400 MHz, $CDCl_3$): $\delta_H$ 2.35 (t, $J$ = 7.6 Hz, 2H), 1.77 (t, $J$ = 7.7 Hz, 2H), 1.68 (s, 1H), 1.44 (s, 9H), 1.22 (s, 6H) ppm.

$^{13}$**C NMR** (101 MHz, $CDCl_3$): $\delta_C$ 174.0, 80.5, 70.4, 38.2, 30.8, 29.4, 28.2 ppm.

**IR** (film): $\nu_{max}$ 3441, 2974, 2933, 2869, 1729, 1367, 1256, 1153 cm$^{-1}$.

**HRMS (ESI$^+$):** calc'd for $[M+Na]^+$, 211.1305; found 211.1314.

## 3.4. Time Studies

### 3.4.1. Aldehyde–olefin coupling

**Procedure: General Procedure A** (with different reactions times, as shown in Table S11) and the **General workup and analysis procedure** were followed. *Note:* All yields were calculated relative to aldehyde **2** (0.2 mmol).

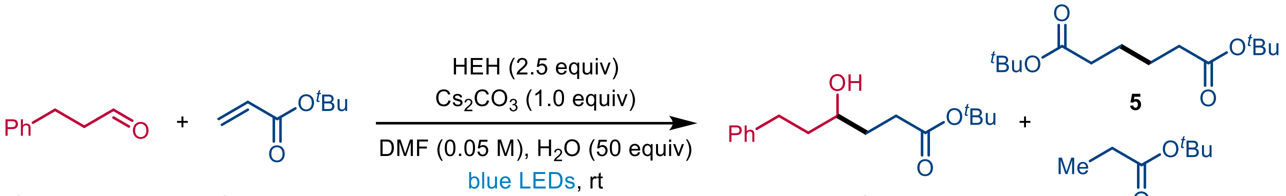


**2** (0.2 mmol) **3** (3.0 equiv) **4** **5** **6**

| Entry | Time | Yield of **4** (%) | Yield of **5** (%) | Yield of **6** (%) | Recovered **2** (%) |
|---|---|---|---|---|---|
| 1 | 15 min | 25 | 21 | 1 | 50 |
| 2 | 30 min | 35 | 31 | 2 | 34 |
| 3 | 1 h | 44 | 41 | 3 | 22 |
| **4** | **2 h** | **53** | **54** | **4** | **11** |
| 5 | 4 h | 54 | 56 | 6 | 6 |
| 6 | 24 h | 55 | 59 | 10 | 3 |

**Table S11.** Time study of the aldehyde–olefin coupling reaction

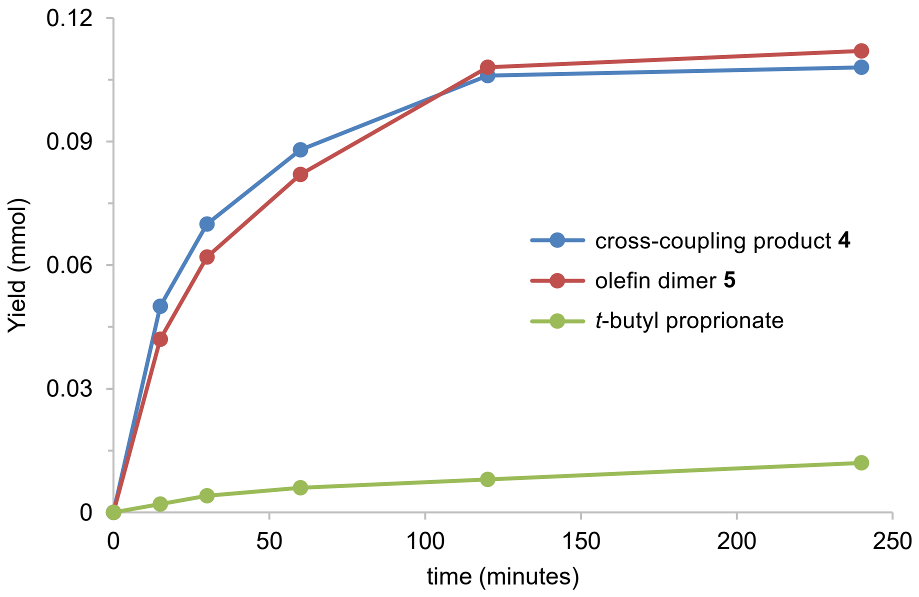


**Figure S3.** Time study of aldehyde–olefin coupling reaction

### 3.4.2. Olefin hydrodimerization

**Procedure:** A 7 mL vial was charged with $Cs_2CO_3$ (65 mg, 0.20 mmol) and Hantzsch ester (HEH, 127 mg, 0.500 mmol). Anhydrous DMF (4.0 mL) and $H_2O$ *if required* (180 µL, 10.0 mmol) were then added to the vial. Subsequently, the solution was degassed by sparging with $N_2$ using a balloon for 1 min before addition of *tert*-butyl acrylate **3** (88 µL, 77 mg, 0.60 mmol). The mixture was then irradiated with blue LEDs (see Figure S1) at room temperature (see Tables S12 and S13 for reaction times). The **General workup and analysis procedure** was then followed. *Note:* The yield of **5** was calculated relative to 0.3 mmol.

With $H_2O$

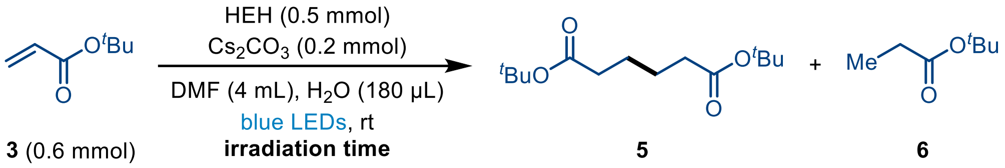


| Entry | Time | Yield of **5** (%) | Yield of **6** (%) | Recovered **3** (%) |
|---|---|---|---|---|
| 1 | 15 min | 26 | 1 | 64 |
| 2 | 30 min | 42 | 1 | 45 |
| 3 | 1 h | 51 | 2 | 31 |
| **4** | **2 h** | **57** | **3** | **22** |
| 5 | 3 h | 59 | 3 | 20 |
| 6 | 4 h | 61 | 3 | 15 |
| 7 | 16 h | 67 | 4 | 5 |

**Table S12.** Time study of acrylate dimerization *with* $H_2O$

Without $H_2O$

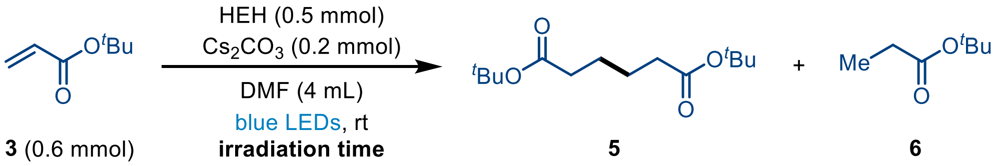


| Entry | Time | Yield of **5** (%) | Yield of **6** (%) | Recovered **3** (%) |
|---|---|---|---|---|
| 1 | 15 min | 53 | 5 | 29 |
| **2** | **30 min** | **74** | **9** | **4** |
| 3 | 1 h | 75 | 10 | <1 |
| 4 | 2 h | 75 | 10 | <1 |
| 5 | 16 h | 75 | 10 | <1 |

**Table S13.** Time study of acrylate dimerization *without* $H_2O$

### 3.4.3. Comparison of the rate of product formation with and without water

The graph in Figure S4 shows the results of the time studies shown in section 3.4.1 and 3.4.2 plotted as total yields (in mmol) of all olefin-derived products (alcohol **4**, olefin dimer **5**, and olefin hydrogenation product **6**) versus time. These show that the rate of product formation is significantly faster in the absence of water. This is likely a result of the lower concentration of HEH anion in the presence of water (see section 3.7).

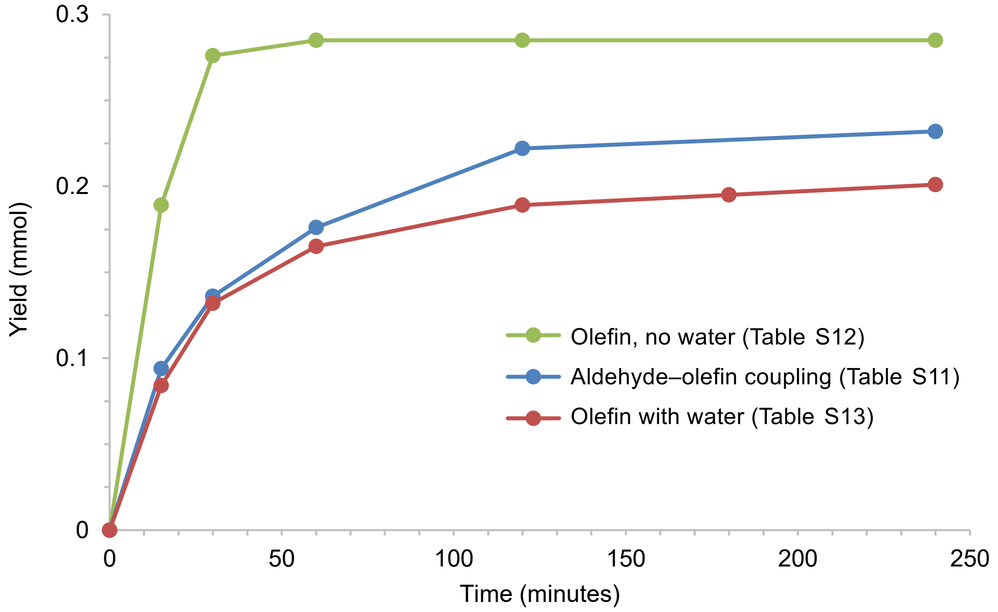


**Figure S4.** Time study of total product formation with and without water

### 3.5. Intramolecular reaction

Substrate **53** was synthesized according to a reported procedure.[19] Products **54a** and **54b** are both known compounds in literature.[20]

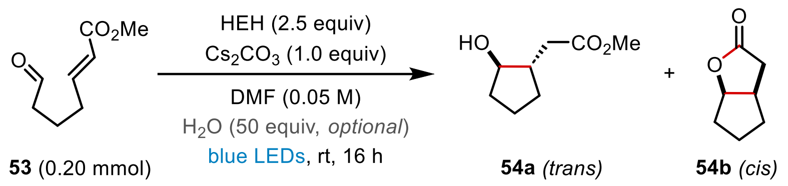


| variations | *trans:cis* | NMR yield |
|---|---|---|
| $H_2O$ (50 equiv) | 69:31 | 48% |
| No $H_2O$ | 58:42 | 22% |

**Procedure:** A 7 mL vial was charged with $Cs_2CO_3$ (65 mg, 0.20 mmol) and Hantzsch ester (HEH, 127 mg, 0.500 mmol). Anhydrous DMF (4.0 mL) and $H_2O$ *if required* (180 µL, 10.0 mmol) were then added to the vial. Subsequently, the solution was degassed by sparging with $N_2$ using a balloon for 1 min before addition of **53** (31.2 mg, 0.20 mmol). The mixture was then irradiated with blue LEDs at room temperature for 16 h. The **General workup and analysis procedure** was then followed to determine the NMR yields of **54a** and **54b** with 0.10 mmol $CH_2Br_2$ as internal standard. See Figures S5 and S6 for the crude $^1$H NMR spectra.

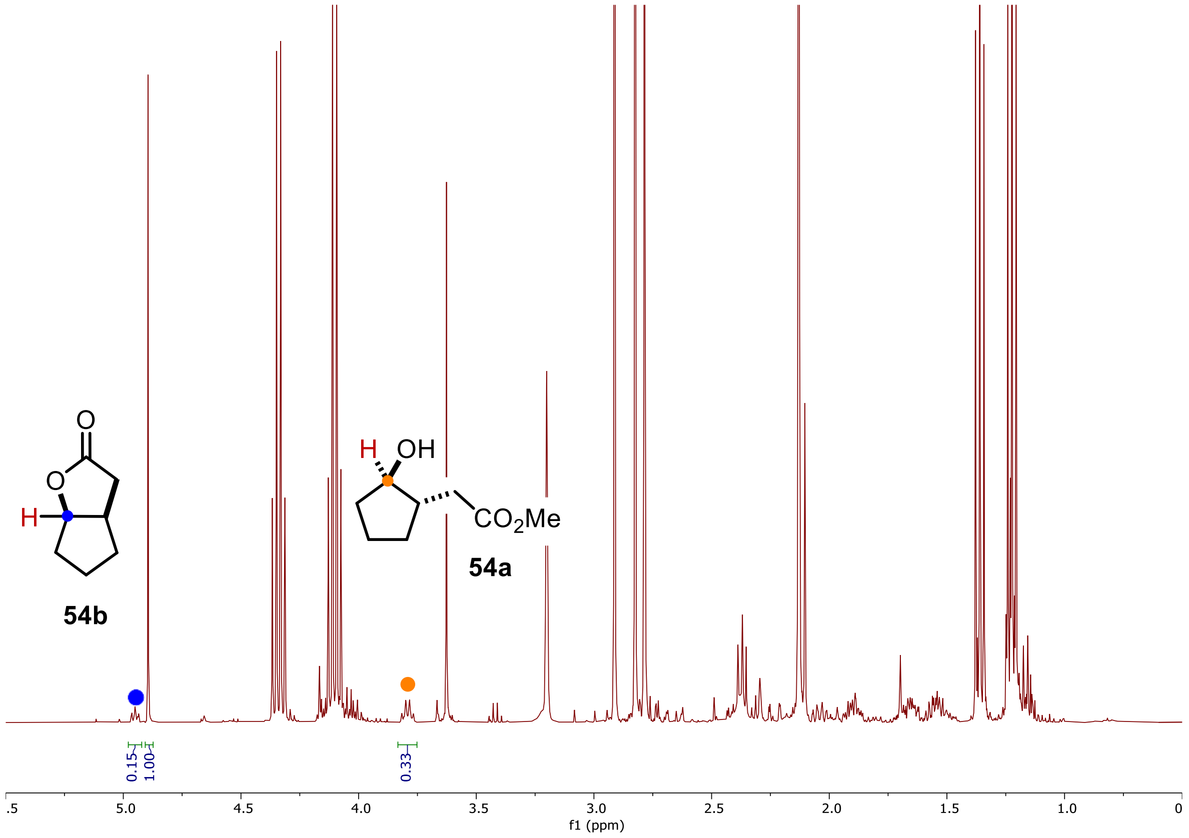


**Figure S5.** Crude $^1$H NMR spectrum of the intramolecular aldehyde–olefin coupling with $H_2O$.

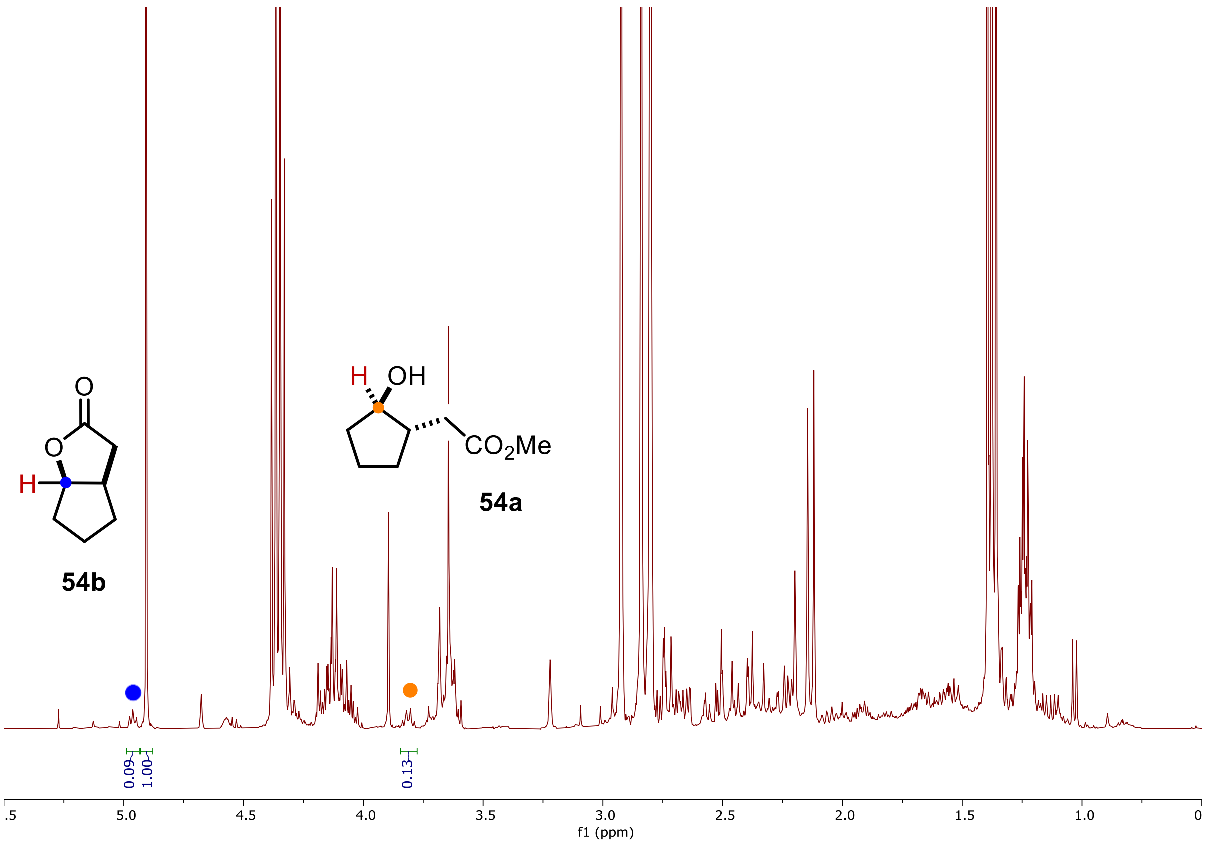


**Figure S6.** Crude $^{1}H$ NMR spectrum of the intramolecular aldehyde–olefin coupling without $H_2O$.

**Comments:**

1) The higher yield of **54** in the presence of water is possibly due to activation of the aldehyde through hydrogen-bonding.

2) The hydrogen-bonding activation could also lead to higher *trans* diastereoselectivity due to the increased steric influence of the hydrogen-bonded aldehyde, which could increase the energy difference between the reactive conformers leading to the *trans* and *cis* diastereomers (Figure S7).

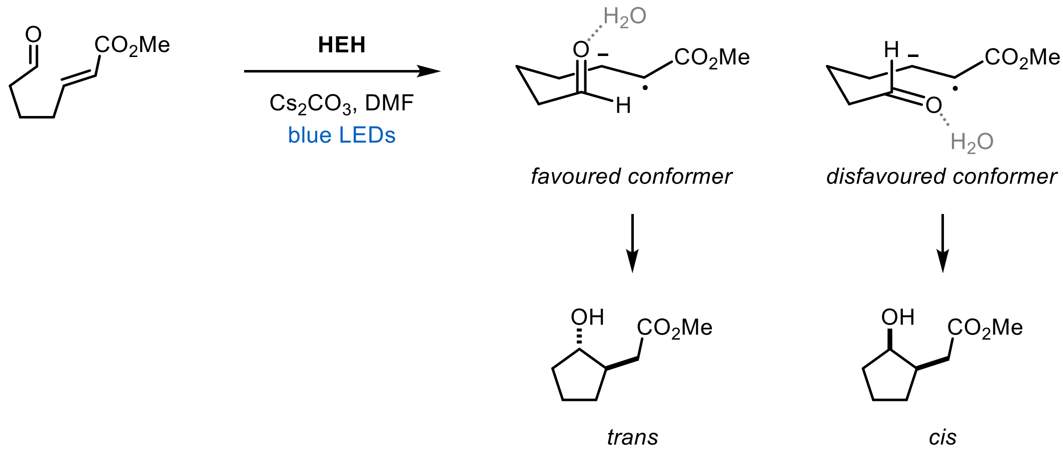


**Figure S7.** Proposed stereochemical model for intramolecular aldehyde–olefin coupling.

## 3.6. Deuterium Labelling Experiments

### 3.6.1. $D_2$-HEH as deuterium source

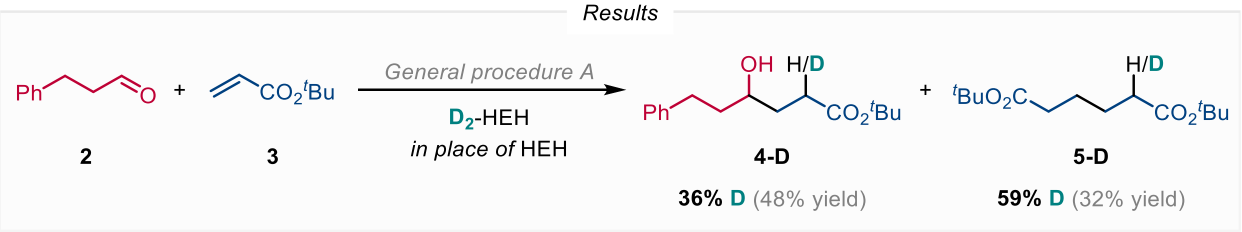


**Procedure: General procedure A** was followed but using $D_2$-HEH in place of HEH. The crude product was purified by flash column chromatography (silica gel, 10:90 to 20:80 EA/PE) to give **4-D** and **5-D** in 48% and 32% yield, respectively.

The deuteration ratio was reported based on integration of peaks in $^{13}C$ IGD (inverse-gated-decoupling) experiment (relaxation time = 10 s) on a Bruker cyro-500 MHz NMR instrument. $^{13}C$ signals of alcohol **4-D** were assigned by analysing the HSQC (heteronuclear single quantum coherence) and HMBC (heteronuclear multiple bond correlation) spectra.

Quantitative $^{13}C$ NMR of alcohol **4-D** ($CDCl_3$, 126 MHz, cryo NMR) $\delta_C$ 173.83, 142.14, 128.56, 125.98, 80.72, [70.89 (0.64C), 70.86 (0.36C)], 39.36, [32.49 (0.64C), 32.42 (0.36C)], 32.24 (0.64C), 32.16, 31.95 (t, $J$ = 19.6 Hz, 0.36C), 28.22.

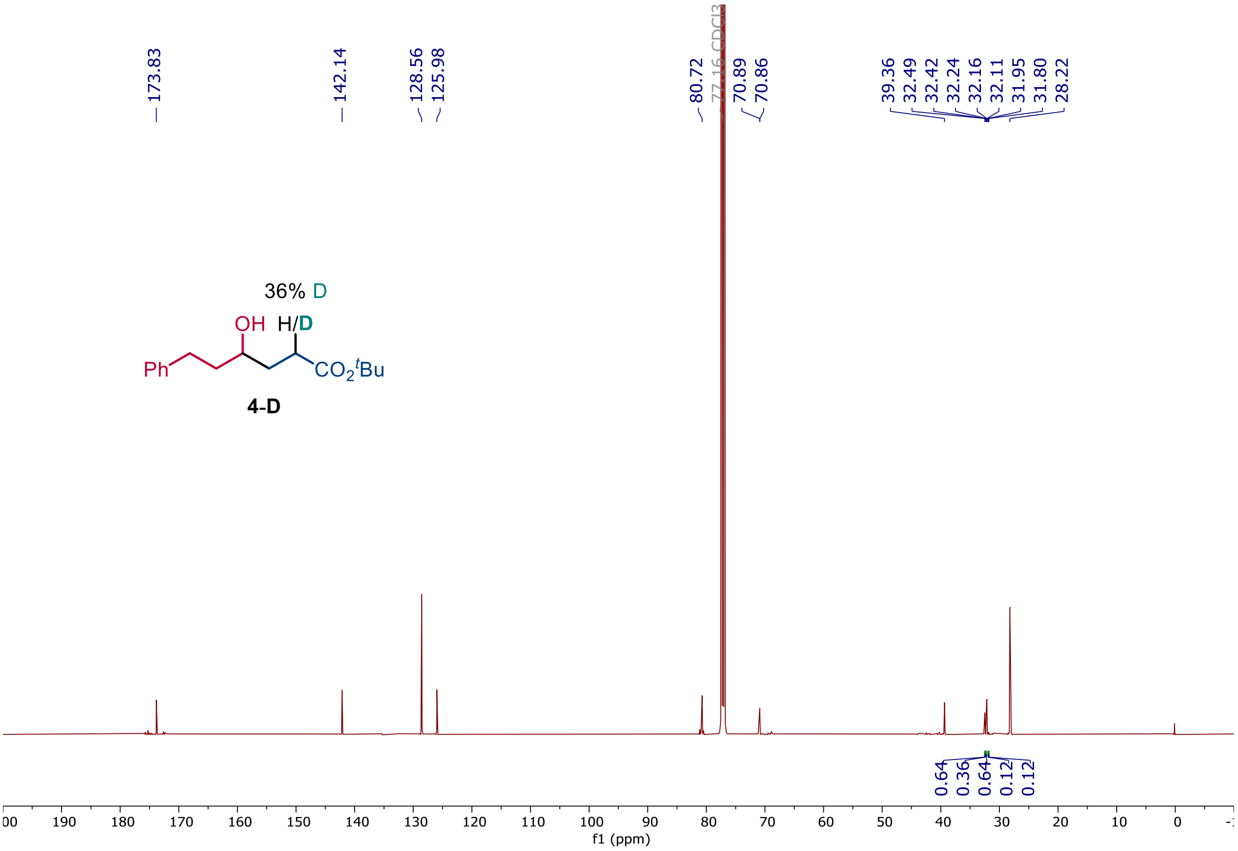

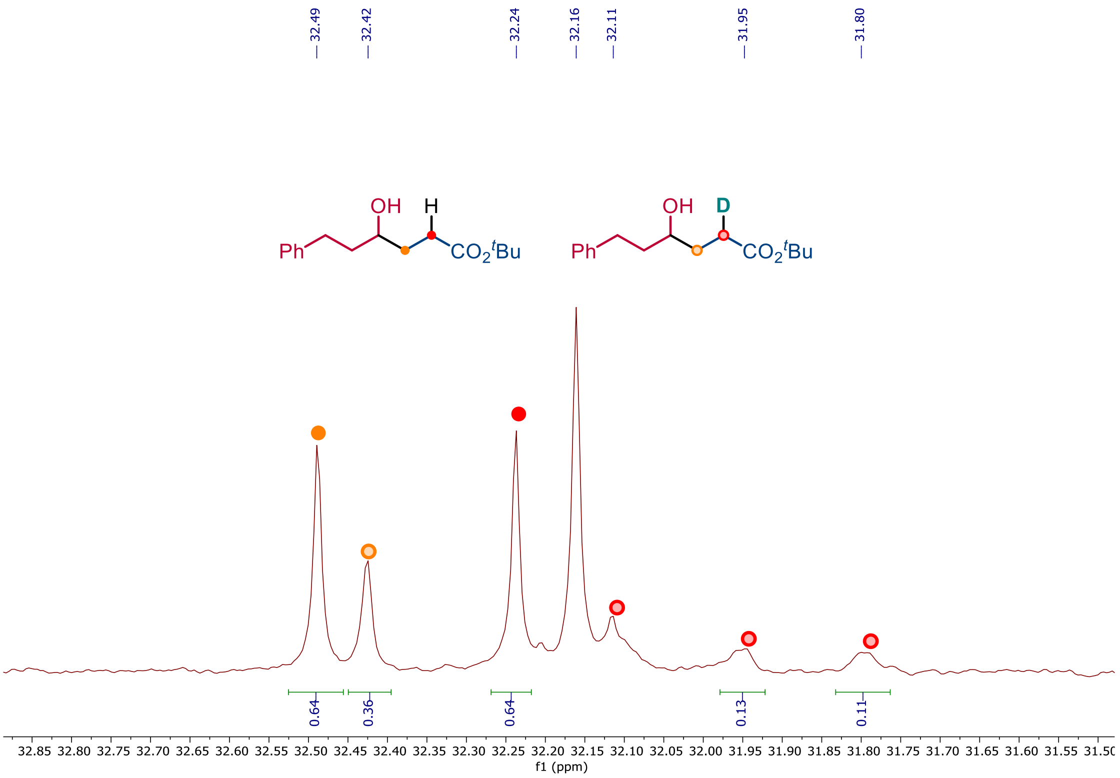


$^{1}$H NMR of **4-D** (500 MHz, $CDCl_3$) $\delta_H$ 7.30-7.26 (m, 2H), 7.23 – 7.13 (m, 3H), 3.64 (m, 1H), 2.80 (dt, $J$ = 13.8, 7.7 Hz, 1H), 2.68 (dt, $J$ = 13.8, 8.1 Hz, 1H), 2.37 (t, $J$ = 7.1 Hz, 1.58H), 1.87 – 1.68 (m, 4H), 1.44 (s, 9H) ppm.

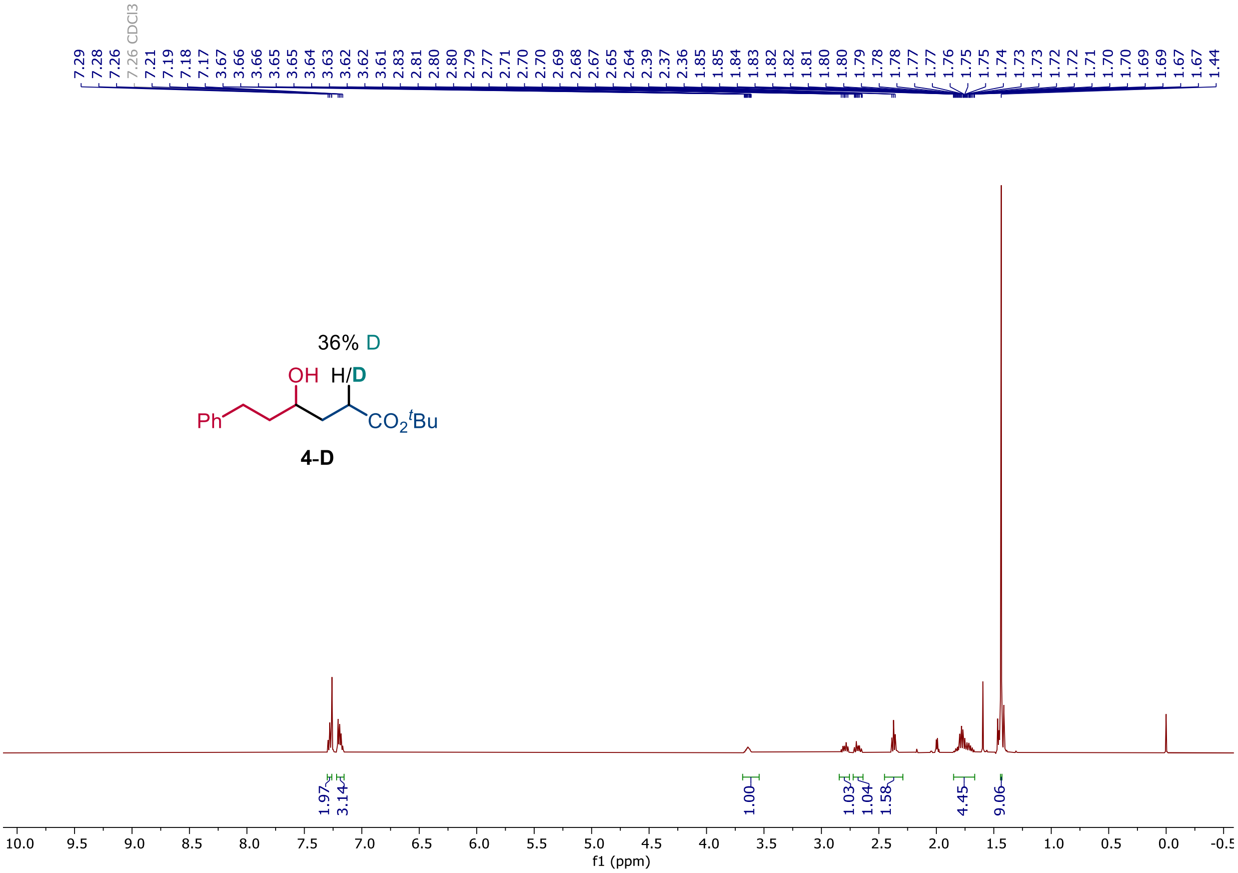

Quantitative $^{13}$C NMR of **5-D** ($CDCl_3$, 126 MHz, cryo NMR) $\delta_C$ 173.00, 80.26, [35.38 (1.41 C), 35.08 (t, *J* = 19.6 Hz, 0.59C)], 28.26, [24.68 (0.82C), 24.66 (0.59C), 24.61 (0.59C)].

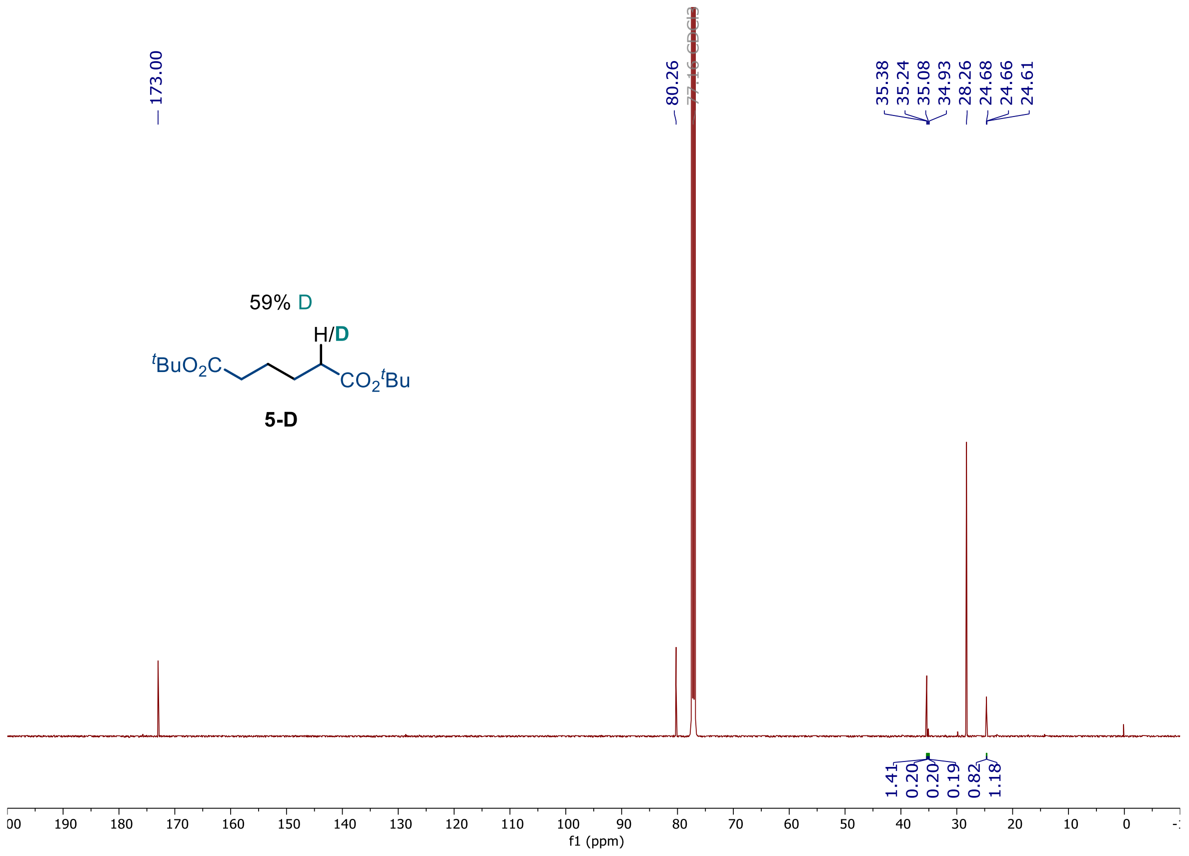


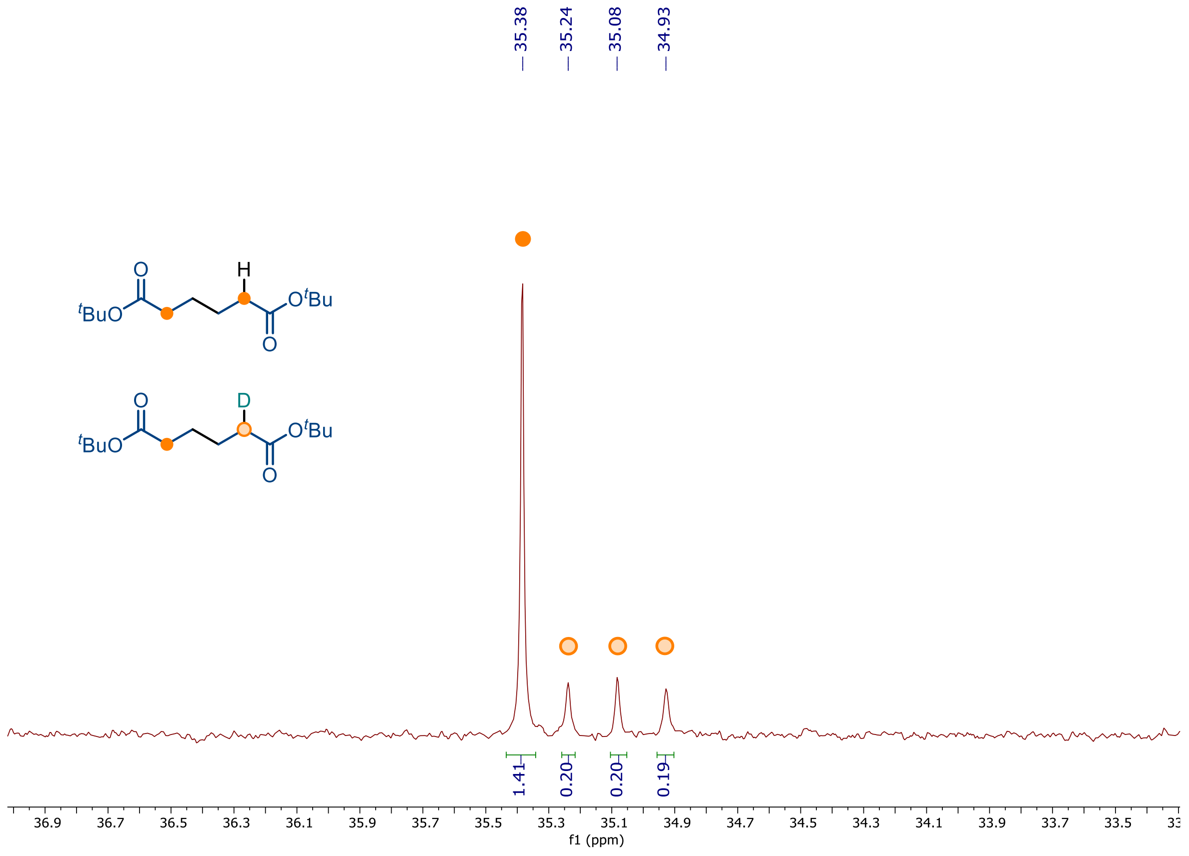

Only mono-deuteration was observed in **5-D**:

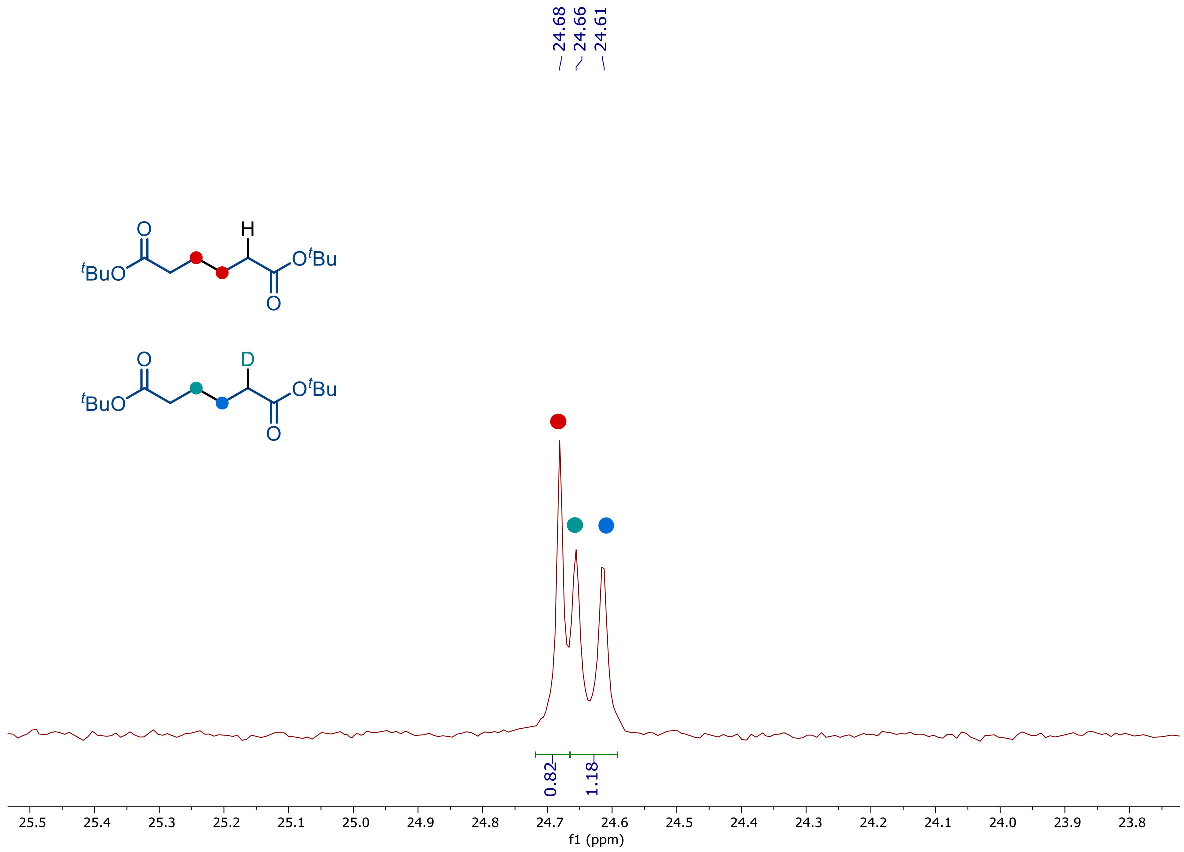


$^1$H NMR of **5-D** (500 MHz, $CDCl_3$)

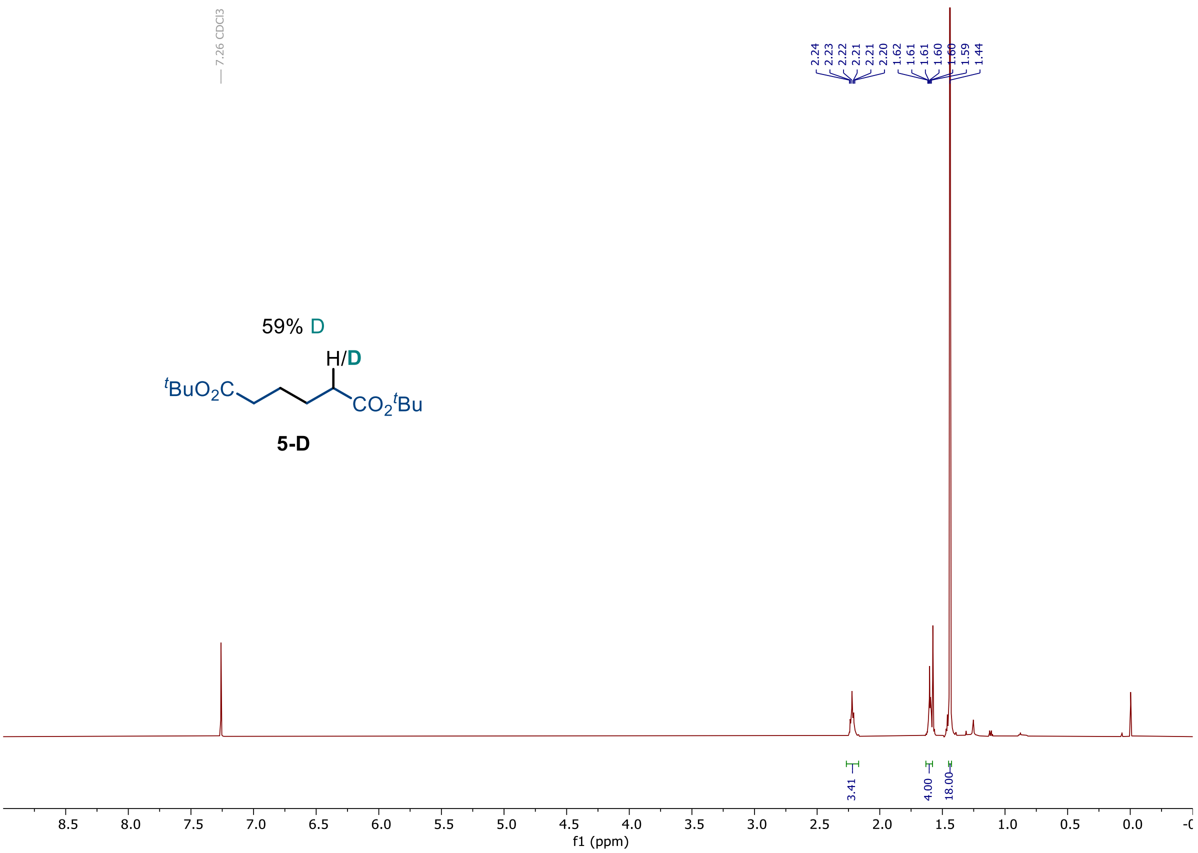

### 3.6.2. Assignment of $^{13}C$ signals of alcohol product

$^{13}C$ NMR (CDCl3, 126 MHz, cryo-NMR)

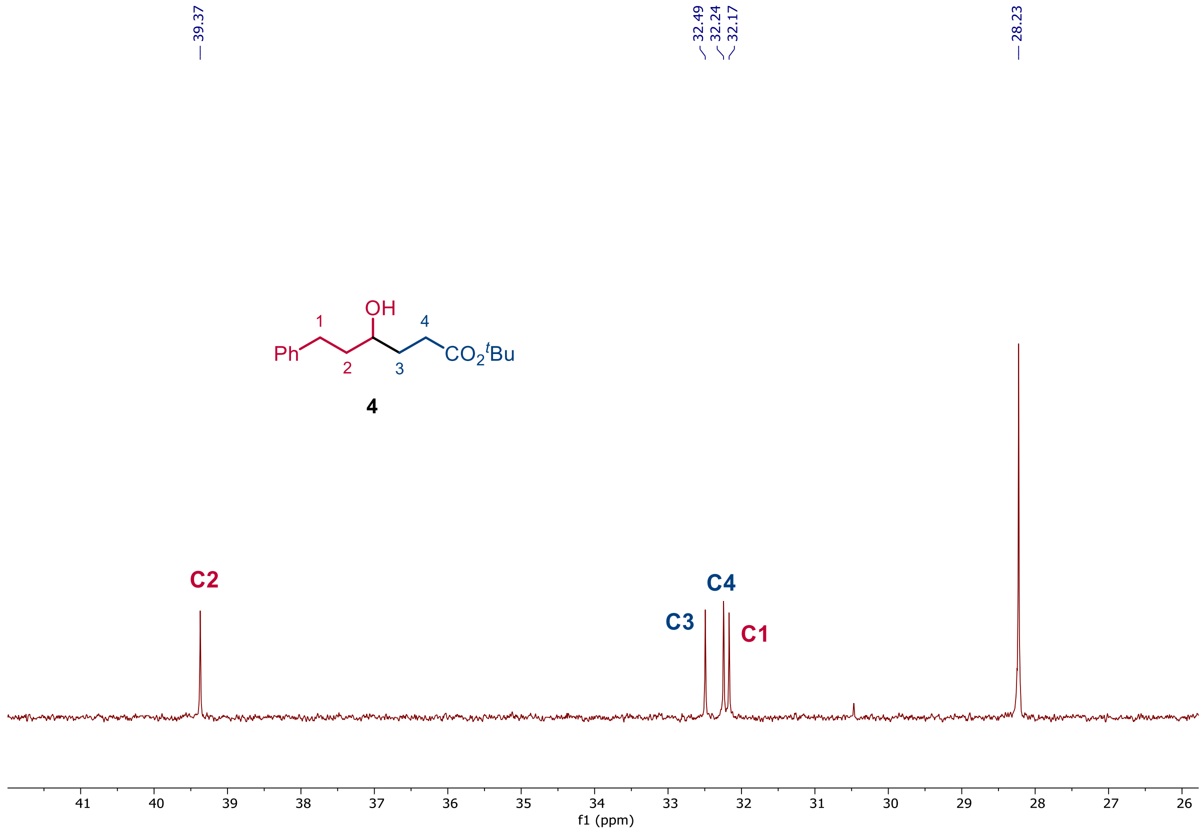


HSQC

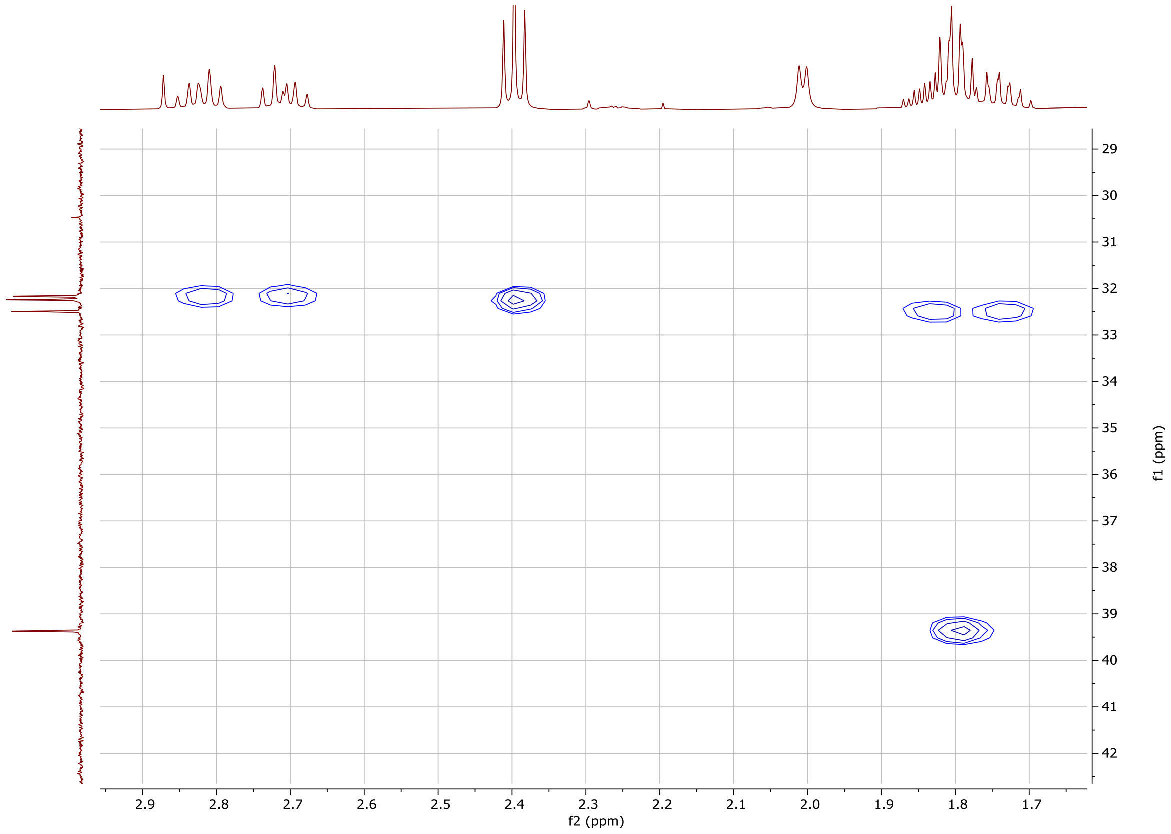

HMBC

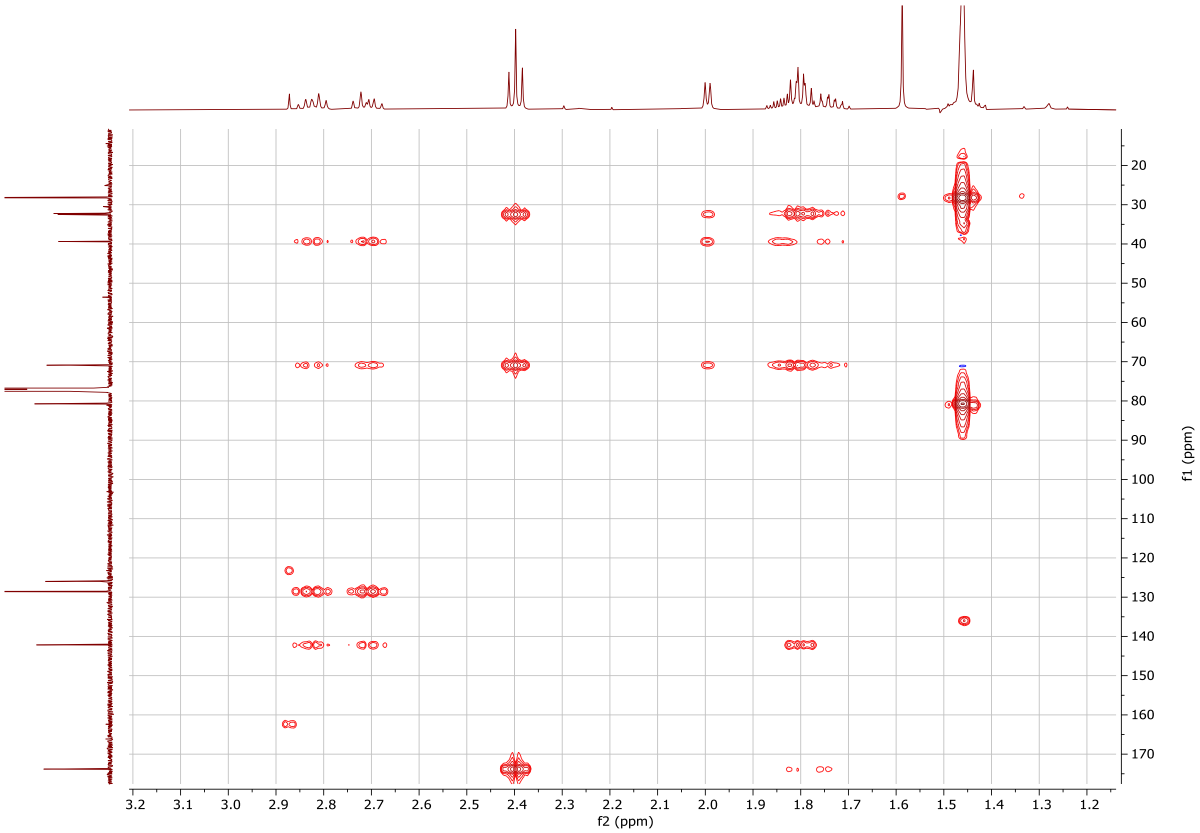
3.2
3.1
3.0
2.9
2.8
2.7
2.6
2.5
2.4
2.3
2.2
2.1
2.0
1.9
1.8
1.7
1.6
1.5
1.4
1.3
1.2
f2 (ppm)
20
30
40
50
60
70
80
90
100
110
120
130
140
150
160
170
f1 (ppm)

### 3.7. Visible-Light Absorption Spectroscopy

**Procedure:** A stock solution of HEH in anhydrous DMF (125 mM) was prepared. Due to the limited solubility of $Cs_2CO_3$ in DMF, the HEH stock solution was added to a vial with pre-weighed $Cs_2CO_3$, followed by addition of the other reagents *if required*. The solution was then mixed with a pipette, and only the clear solution was transferred to a cuvette for analysis in the spectrometer.

Reagents: HEH in DMF (125 mM, 2 mL), $Cs_2CO_3$ (32.6 mg, 0.100 mmol), KOH (5.6 mg, 0.10 mmol), aldehyde (3-phenylpropanal **2**, 13.4 mg, 0.100 mmol, 50 mM), acrylate (*tert*-butyl acrylate **3**, 25.6 mg, 0.200 mmol, 100 mM), $H_2O$ (90 µL, 5.0 mmol, 2.5 M).

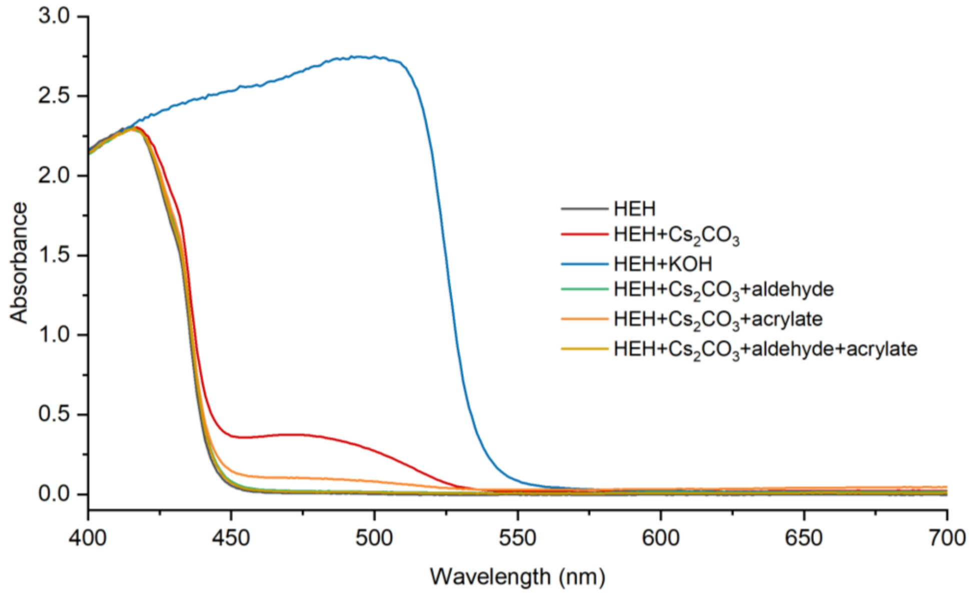


**Figure S8.** Visible-light absorption spectra of HEH (with reagents)

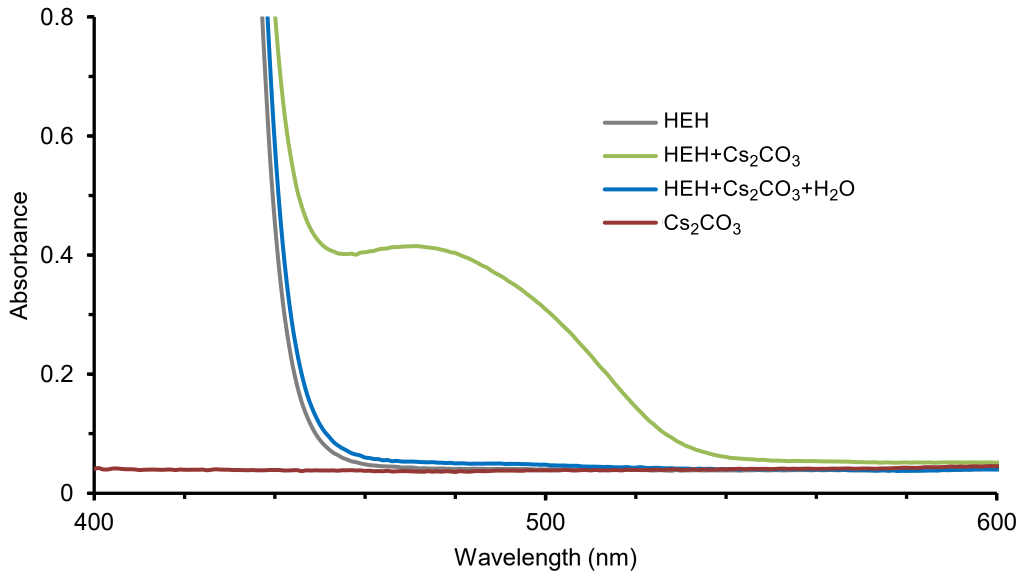


**Figure S9.** Visible-light absorption spectra of HEH (effect of water)

**Comments:**

1) An additional absorption feature (471 nm) was observed with the addition of a base, $Cs_2CO_3$ or KOH (Figure S8). This peak was attributed to the formation of the HEH anion through deprotonation of HEH.[21]

2) No additional peaks were observed when the aldehyde and acrylate were added (Figure S8). This suggests that no electron donor-acceptor (EDA) complex formation occurred between these substrates and HEH and $Cs_2CO_3$.

3) Addition of water to the mixture of HEH and $Cs_2CO_3$ resulted in a decrease in intensity of the absorption feature at 471 nm (Figure S9). This was attributed to a decrease in concentration of HEH anion due to the presence of the additional proton source.

### 3.8. Cyclic Voltammetry

The following procedure was adapted from a previous report by Nicewicz and co-workers.[22] Cyclic voltammograms were collected with a PalmSens multi-channel potentiostat. Samples were prepared with 0.1 mmol of substrate in 10 mL of 0.1 M tetra-*n*-butylammonium hexafluorophosphate ($^{n}Bu_4NPF_6$) in anhydrous and degassed DMF. Measurements were conducted using a Pt working electrode, Pt wire counter electrode, $Ag/AgNO_3$ (0.1 M) reference electrode, and a scan rate of 100 mV/s. Reduction potentials of the substrates (Figures S10 and S11) were measured by scanning potentials in the negative direction with a starting point of 0.0 V and a vertex potential of −3.0 V. The oxidation potentials of HEH and $HEH^-$ (Figure S12) were measured by scanning potentials initially in the positive direction with a starting point of 0.0 V and vertex potentials of +1.5 V and −3.0 V.

The background current (only solvent and electrolyte) was subtracted from the collected data (in the presence of the substate) before analysis. The maximum current ($C_p$) was identified, and the $E_{p/2}$ was estimated at half this value ($C_{p/2}$).

The reference electrode was calibrated with the internal reference $Fc^+/Fc$, which was measured to be +0.13 V vs. $Ag/AgNO_3$ in DMF. The reported conversion for $Fc^+/Fc$ to SCE is +0.38 V, resulting in +0.25 V when converting from our $Ag/AgNO_3$ reference to SCE. *Note:* $E_{p/2}$ values of substrates in the presence of 50 equiv $H_2O$ could not be obtained due to the significant reduction wave of $H_2O$.

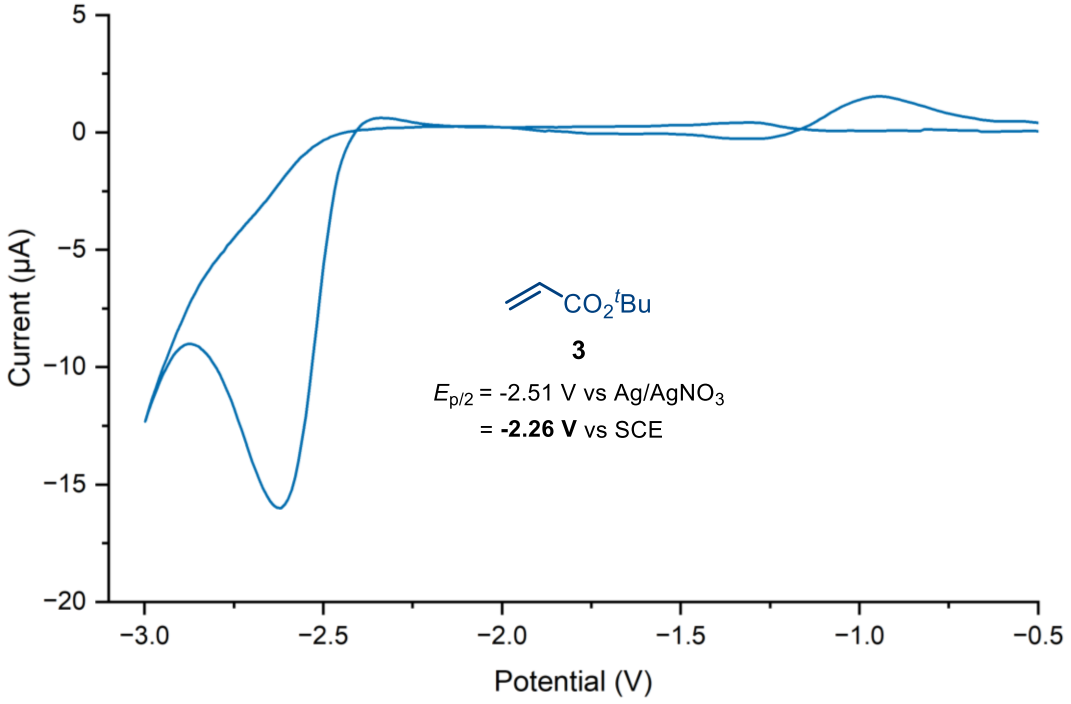


**Figure S10.** Cyclic voltammogram of acrylate **3** in anhydrous DMF (vs. $Ag/AgNO_3$)

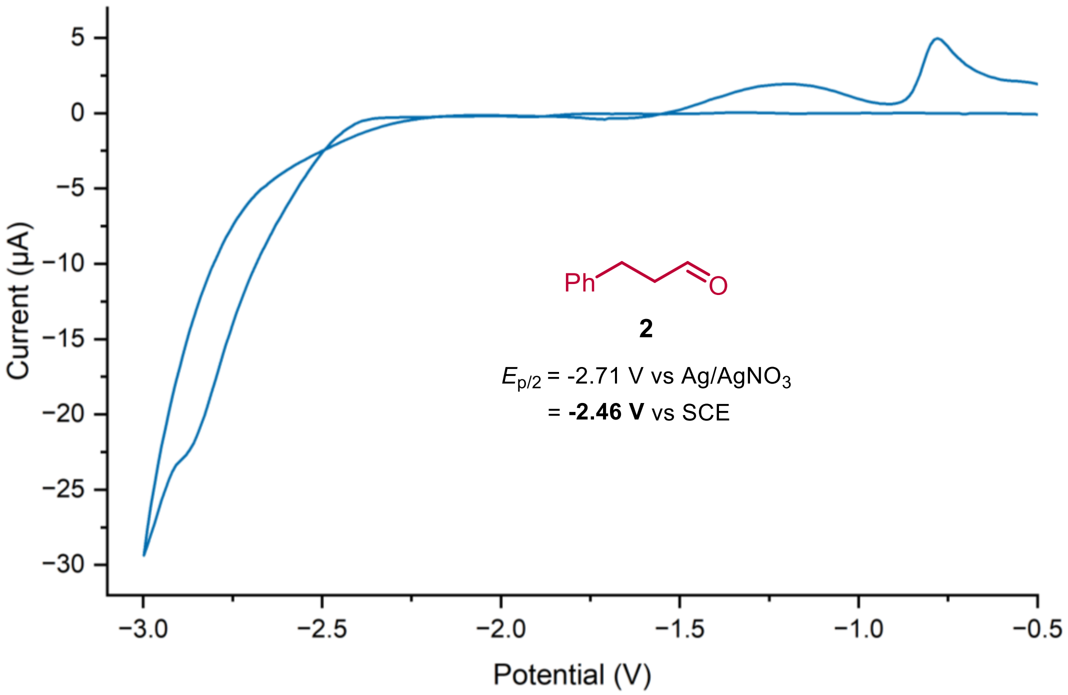


**Figure S11.** Cyclic voltammogram of aldehyde **2** in anhydrous DMF (vs. Ag/AgNO$_3$)

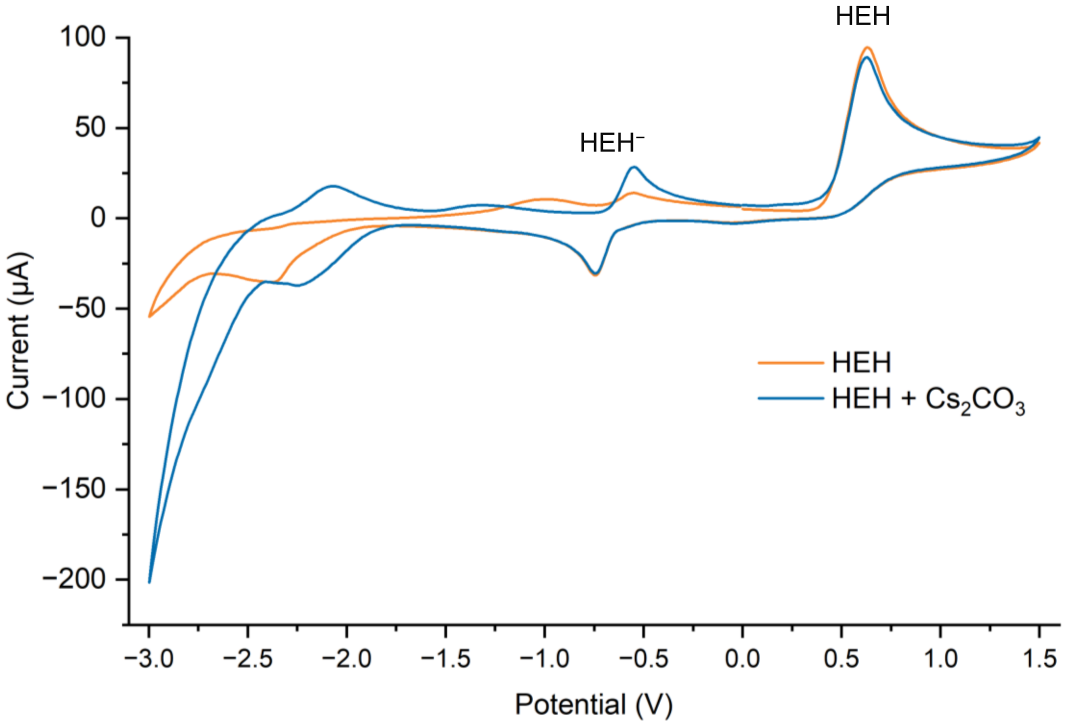


**Figure S12.** Cyclic voltammogram of HEH and HEH/Cs$_2$CO$_3$ (1 equiv) in anhydrous DMF (vs. Ag/AgNO$_3$)

$E_{1/2}$ (HEH$^•$/HEH$^–$) = −0.65 V vs. Ag/AgNO$_3$

= **−0.40 V** vs. SCE

### 3.9. Estimation of the Excited-State Oxidation Potential of HEH Anion

The excited-state oxidation potential of HEH anion, $E_{1/2}$ ($HEH^{\bullet}$/$*HEH^{-}$), was estimated following the reported procedures.[21,23]

The fluorescence emission spectrum of $HEH^{-}$ (formed in situ upon addition of $Cs_2CO_3$ in DMF) was recorded from 476 nm to 700 nm after excitation at 456 nm (Figure S13). The UV-Vis *absorption* and fluorescence *emission* spectra were normalized and the intersection wavelength (512 nm) was used to estimate the value of excited-state energy, $E_{0\text{-}0}$ ($HEH^{-}$), to be 2.42 eV.

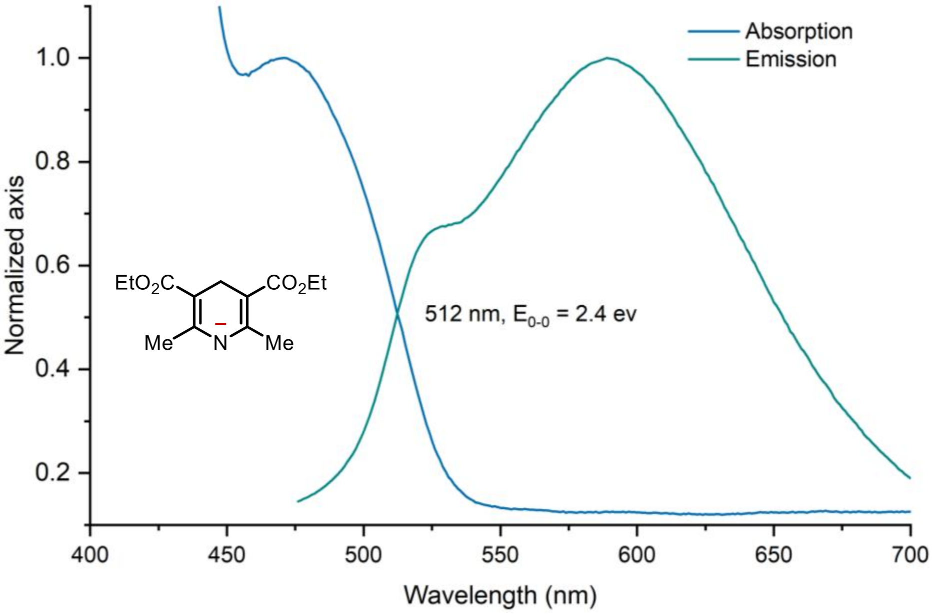


**Figure S13.** Normalized absorption and emission spectra of $HEH^{-}$

Combing the data collected from the CV studies (Figure S12), the reduction potential of excited-state $HEH^{-}$ could be estimated using the following equation:

$$E_{1/2}(HEH^{\bullet}/*HEH^{-}) = E_{1/2}(HEH^{\bullet}/HEH^{-}) - E_{0\text{-}0}(HEH^{-})$$
$$= -0.40\text{ V} - 2.42\text{ V}$$
$$= \mathbf{-2.82\ V}\text{ vs. SCE (in DMF)}$$

This estimated value is comparable to the literature value for $E_p$ ($HEH^{\bullet}$/$*HEH^{-}$) of −2.49 V vs. SCE in DMSO.[21]

## 3.10. Quantum Yield Measurement

We conducted quantum yield measurements to investigate whether our reactions involved radical chains.[24] The following procedure was adapted from a previous report by Noble and Aggarwal.[25]

*Determination of the Photon Flux:*

The photon flux of the Kessil Tuna Blue lamp setup was determined using standard ferrioxalate actinometry. A 0.018 M ferrioxalate solution was prepared by dissolving 178 mg of potassium ferrioxalate trihydrate {$K_3[Fe(C_2O_4)_3]·3H_2O$} and 84 µL of $H_2SO_4$ (95–98%) in 20 mL of water. This stock solution was stored in a foil-covered bottle in the dark. A buffer solution was prepared by dissolving 2.5 g of sodium acetate (NaOAc) and 0.50 mL of $H_2SO_4$ (95–98%) in 50 mL of water.

With exposure to background light minimized, 4.0 mL of the 0.018 M ferrioxalate solution was added to a 7 mL vial. The vial was positioned in the vial holder about 5 cm from a single Kessil Tuna Blue LED lamp (see Figure S1) and was irradiated for a short period of time (0-30 s). Immediately after irradiation, 100 µL of the solution was transferred to a foil-covered 10 mL volumetric flask containing 15 mg of 1,10-phenanthroline dissolved in 3.0 mL of the buffer solution. Water was then added to the flask to make a total volume of 10 mL. The flask was shaken to ensure sufficient mixing, and the solution was stored in the dark for around 20 min. 2.0 mL of the solution was then transferred to a quartz cuvette (1.0 mL path length) and the absorbance at λ = 510 nm was measured by UV/Vis spectroscopy (Figure S14). This procedure was implemented for 15 s and 30 s of irradiation, and the absorbance of a non-irradiated sample was also measured.

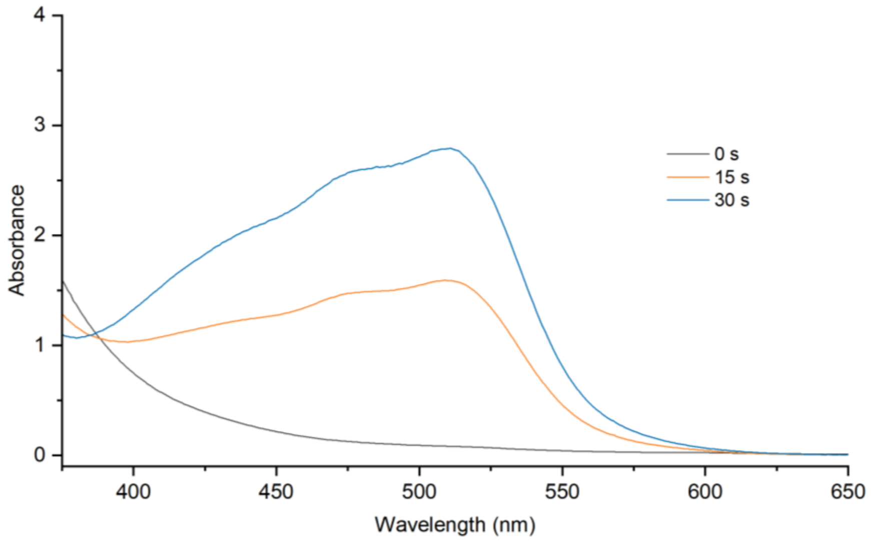


**Figure S14.** UV-vis absorption spectra of ferrioxalate/1,10-phenanthroline solutions (after irradiation with Kessil Tuna Blue light for 0–30 s).

The number of moles of $Fe^{2+}$ formed was calculated using:

$$mol\ of\ Fe^{2+} = \frac{V_1\ V_3\ \Delta A(510\ nm)}{V_2\ l\ \varepsilon(510\ nm)}$$

Where $V_1$ is the volume of ferrioxalate solution irradiated (4.0 × $10^{-3}$ L), $V_2$ is the volume of the aliquot taken for measurement of the concentration of $Fe^{2+}$ ions (1.0 × $10^{-4}$ L), $V_3$ is the final volume after complexation with 1,10-phenanthroline (1.0 × $10^{-2}$ L), $\Delta A$(510 nm) is the difference in absorbance at λ = 510 nm between the

irradiated and non-irradiated ferrioxalate/1,10-phenanthroline solutions, *l* is the optical path length of the irradiation cell (1.0 cm), and $\varepsilon$(510 nm) is the molar absorptivity of the $Fe(phen)_3^{2+}$ complex at λ = 510 nm (11,100 L $mol^{-1}$ $cm^{-1}$).

The moles of $Fe^{2+}$ were plotted as a function of time (Figure S15):

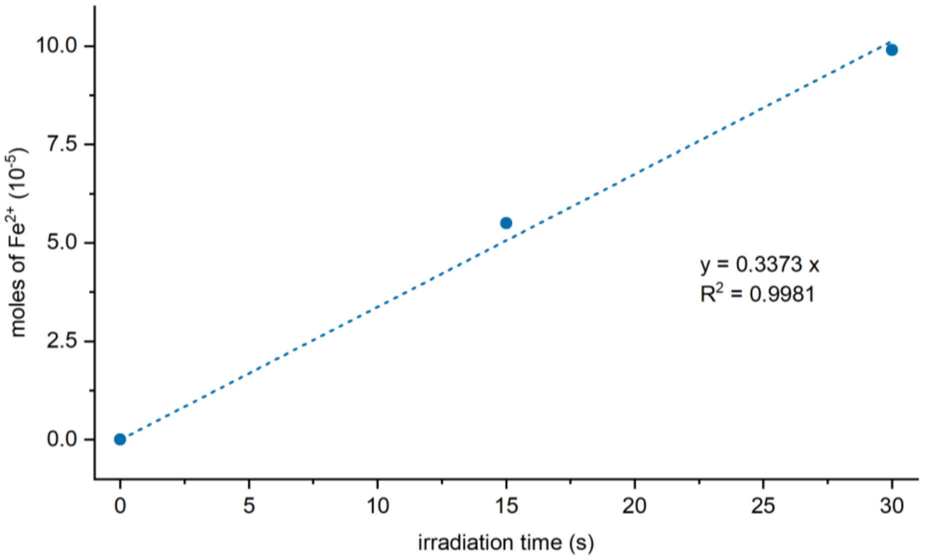


**Figure S15.** Moles of $Fe^{2+}$ formed vs. irradiation time

The photon flux was then calculated using:

$$photon\ flux = \frac{\mathrm{mol\ Fe}^{2+}}{\phi \cdot f \ \cdot t}$$

Where mol $Fe^{2+}$/t is the slope fitted in Figure S15 ($3.37\times10^{-6}$). $\Phi$ is the quantum yield of the ferrioxalate actinometer (0.84 at λ = 456 nm),[26] and *f* is the fraction of absorbed light at λ = 456 nm, where $f = 1 - 10^{-A}$. The absorbance (A) of the 0.018 M ferrioxalate stock solution at λ = 456 nm was measured by UV/Vis spectroscopy to be 0.27, therefore *f* = 0.46.

$$photon\ flux = \frac{3.37\ \times 10^{-6}}{0.84 \times 0.46} = 8.72 \times 10^{-6}\ \mathrm{einstein}\ s^{-1}$$

*Determination of the Quantum Yield:*

The quantum yield ($\Phi$) was then calculated using:

$$\Phi = \frac{\text{mol product}}{photon\ flux \cdot f \cdot t}$$

**Aldehyde–olefin coupling:**

$A$ = 0.024 at 456 nm, thus $f = 1 - 10^{-A} = 0.054$. When $t$ = 30 min (1800 s), the yield of **4** was 0.070 mmol, the yield of **5** was 0.062 mmol, and the yield of **6** was 0.004 mmol (*total yield = 0.136 mmol*).

$$\Phi_1 = \frac{1.36 \times 10^{-4}}{8.72 \times 10^{-6} \cdot 0.054 \cdot 1800} = 0.158 = 15.8\%$$

**Olefin-olefin coupling (*with* $H_2O$):**

$A$ = 0.04 at 456 nm, thus $f = 1 - 10^{-A} = 0.088$. When $t$ = 30 min (1800 s), the yield of **5** was 0.126 mmol, and the yield of **6** was 0.006 mmol (*total yield = 0.132 mmol*).

$$\Phi_2 = \frac{1.32 \times 10^{-4}}{8.72 \times 10^{-6} \cdot 0.088 \cdot 1800} = 0.094 = 9.4\%$$

**Olefin-olefin coupling (*no* $H_2O$):**

$A$ = 0.39 at 456 nm, thus $f = 1 - 10^{-A} = 0.59$. When $t$ = 30 min (1800 s), the yield of **5** was 0.222 mmol, and the yield of **6** was 0.054 mmol (*total yield = 0.276 mmol*)..

$$\Phi_3 = \frac{2.76 \times 10^{-4}}{8.72 \times 10^{-6} \cdot 0.59 \cdot 1800} = 0.029 = 2.9\%$$

**Comments:**

1) No evidence for chain processes was found since the quantum yields were all below 20%.[24]

2) The relatively low quantum yields could be attributed to non-productive photochemical processes (e.g., phosphorescence) or back electron transfer from the olefin radical anion.

3) The higher quantum yields observed for reactions performed in the presence of water could result from a stabilising interaction between water and the excited state HEH anion; or through reduced back electron transfer caused by the increased rate of coupling of the olefin radical anion with the aldehyde and olefin.

# 4. SPECTROSCOPIC DATA

$^{1}$H NMR (400 MHz, $CDCl_3$) of **4** (*see procedure*)

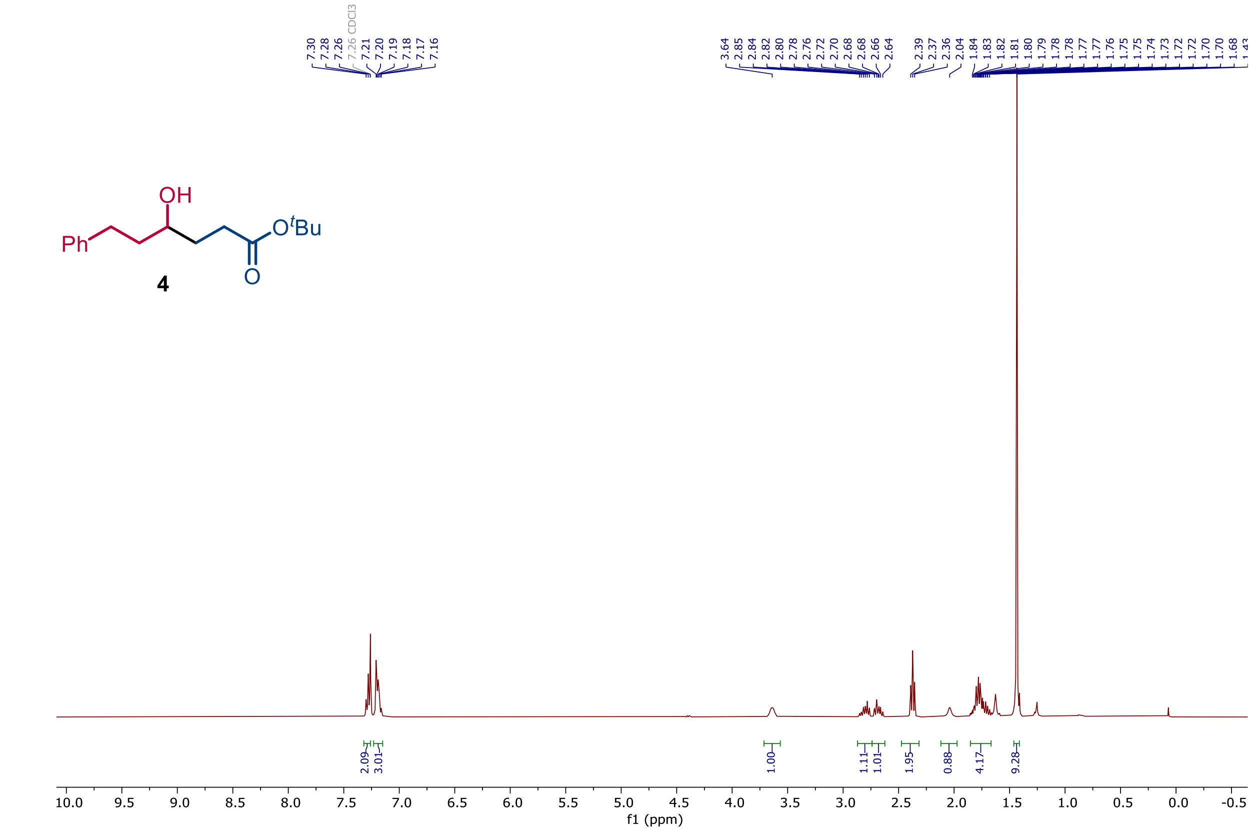


$^{13}$C NMR (101 MHz, $CDCl_3$) of **4**

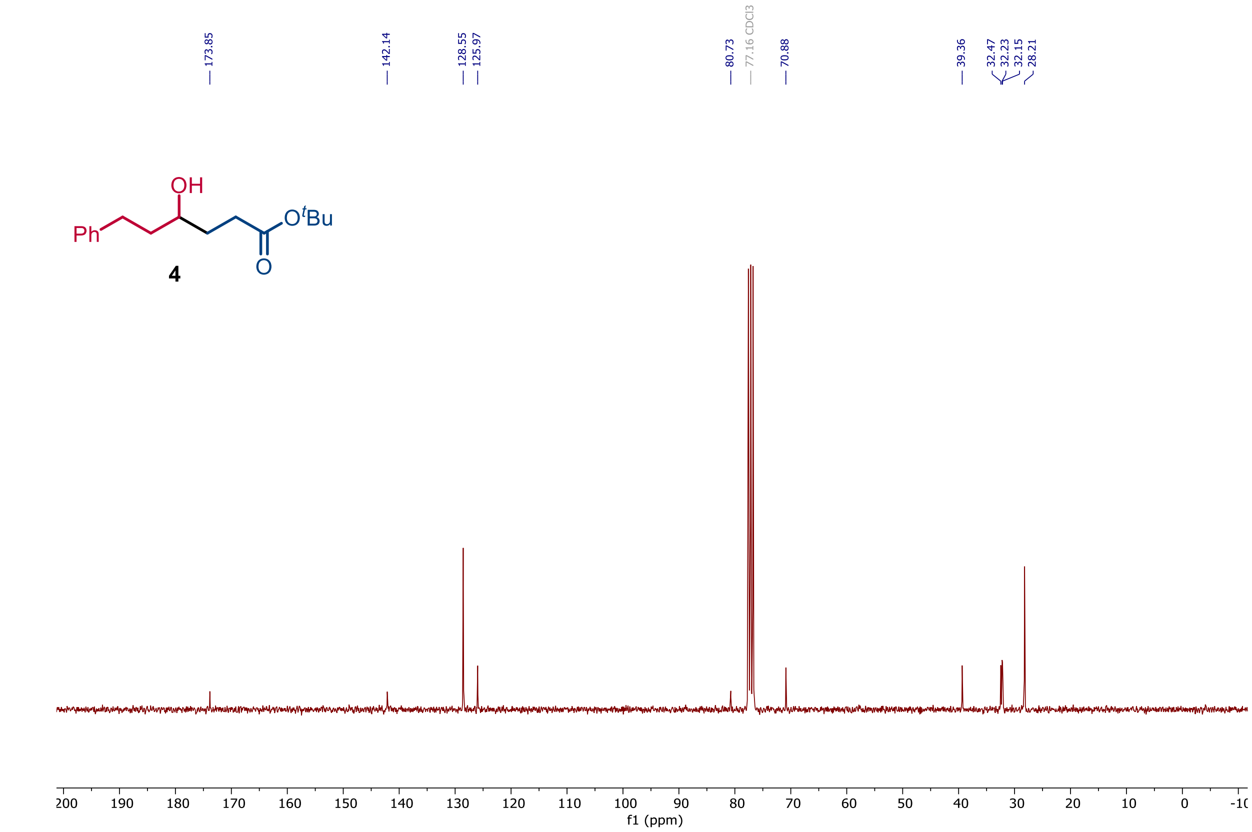

$^{1}$H NMR (400 MHz, $CDCl_3$) of **7** (*see procedure*)

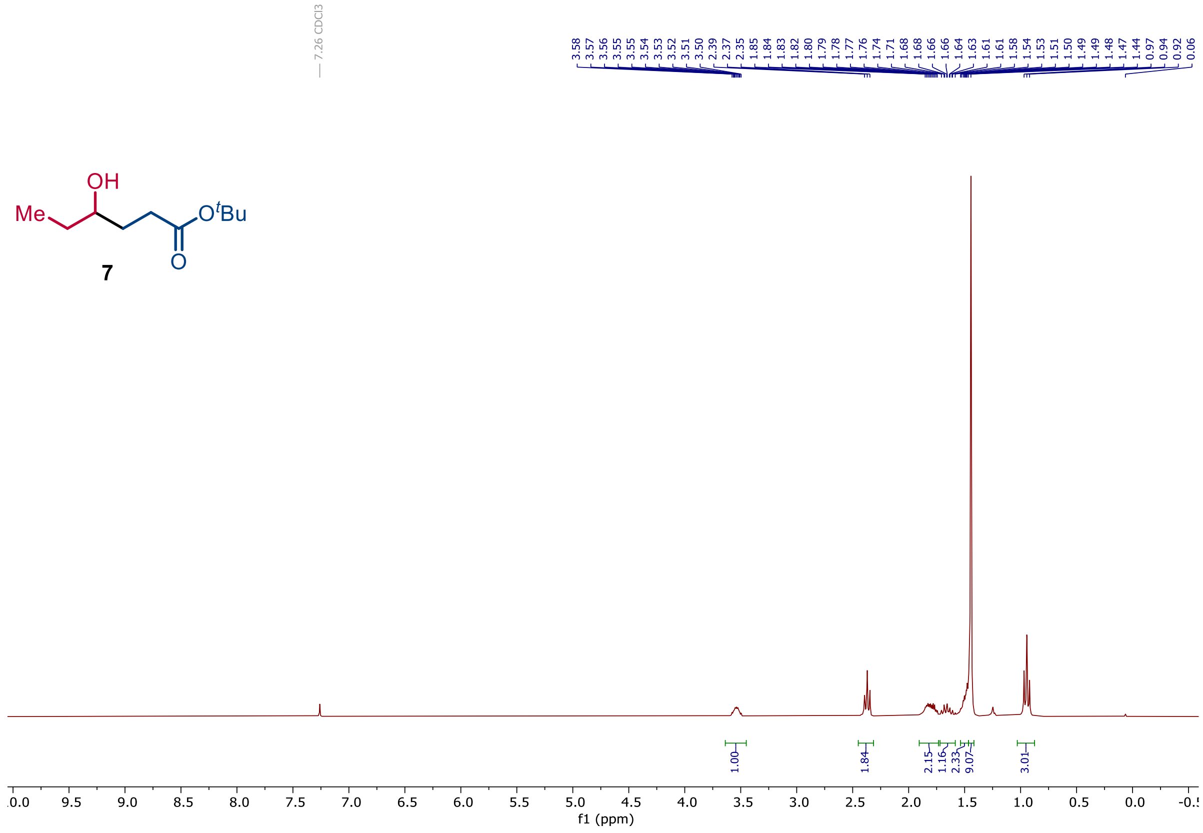


$^{13}$C NMR (101 MHz, $CDCl_3$) of **7**

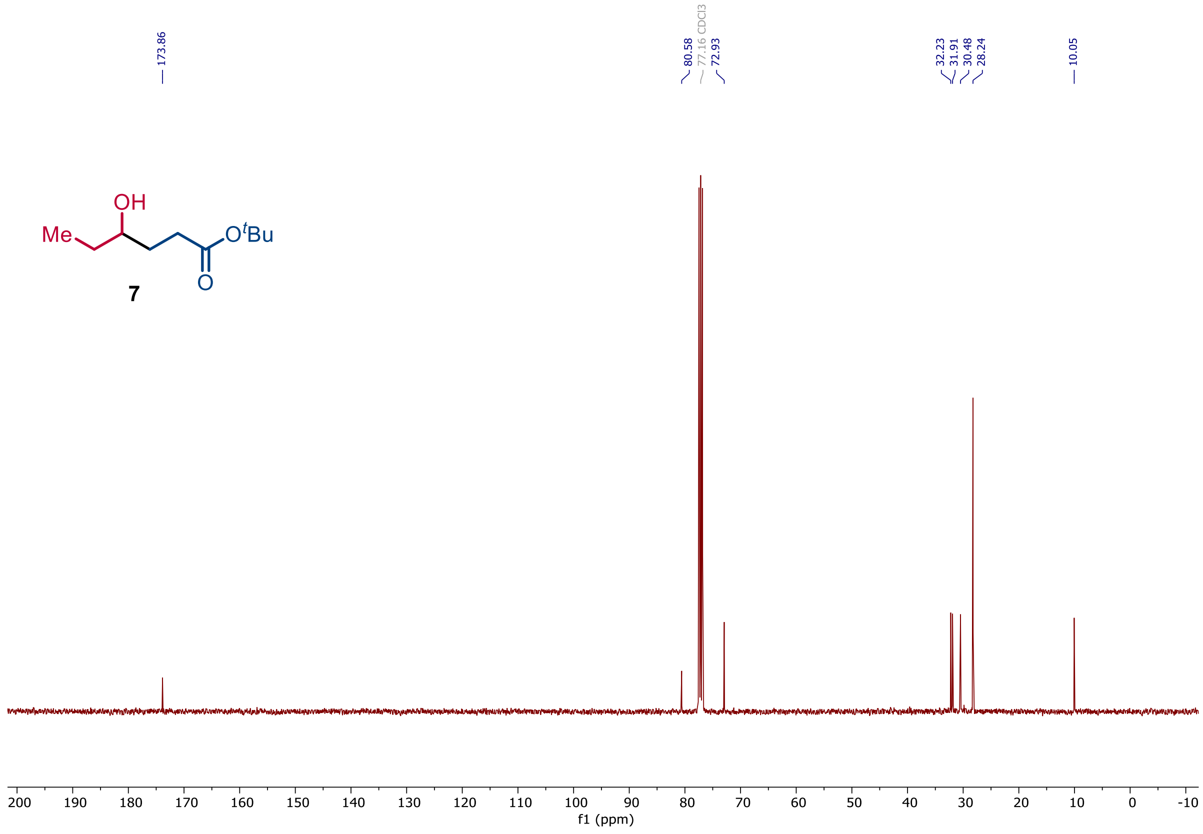

$^{1}$H NMR (400 MHz, $CDCl_3$) of **8** (*see procedure*)

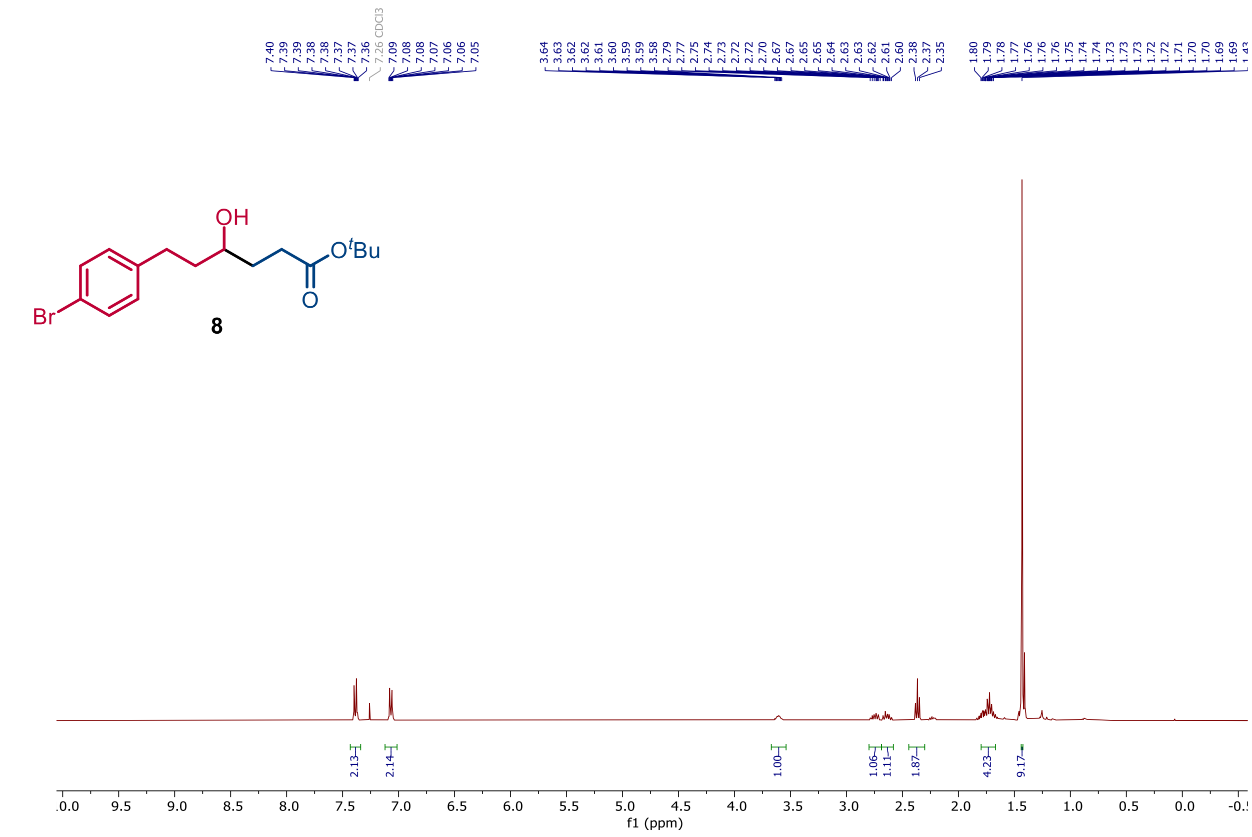


$^{13}$C NMR (101 MHz, $CDCl_3$) of **8**

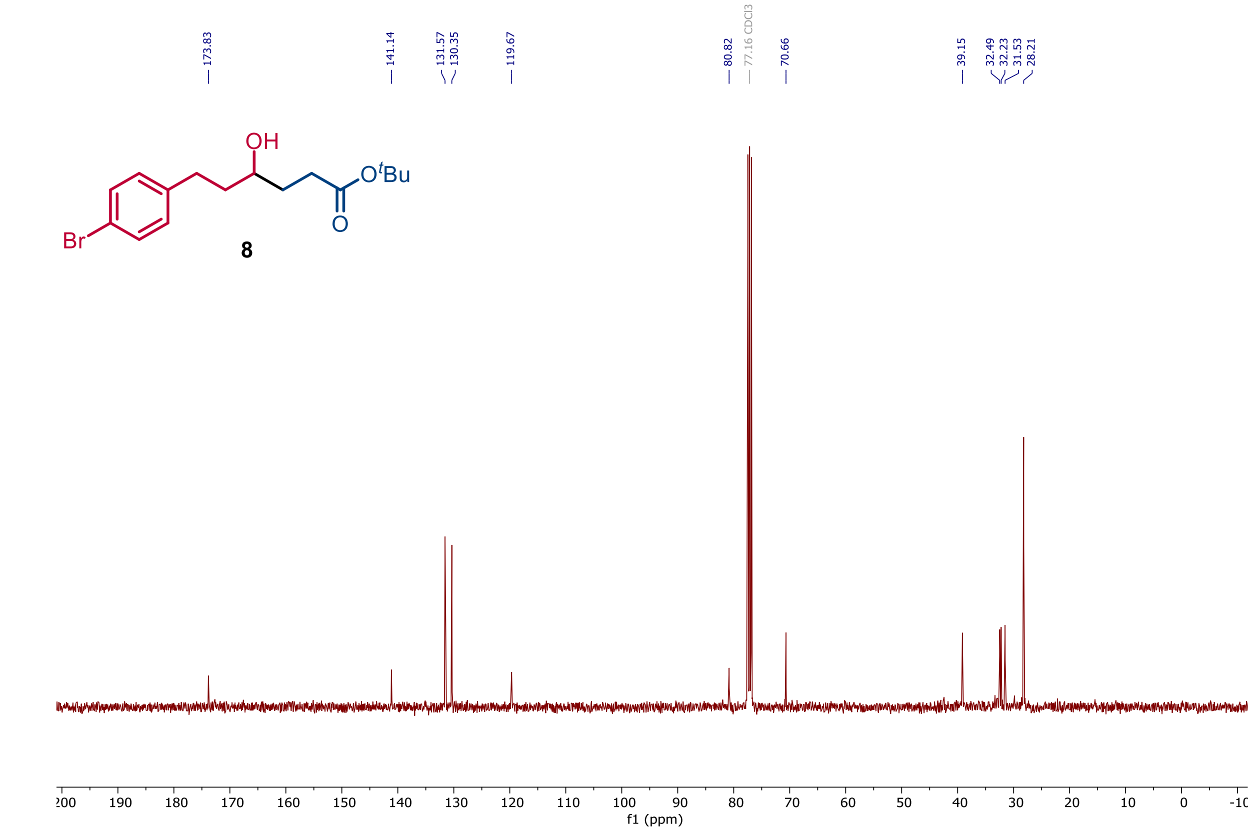

$^{1}H$ NMR (400 MHz, $CDCl_3$) of **9** (*see procedure*)

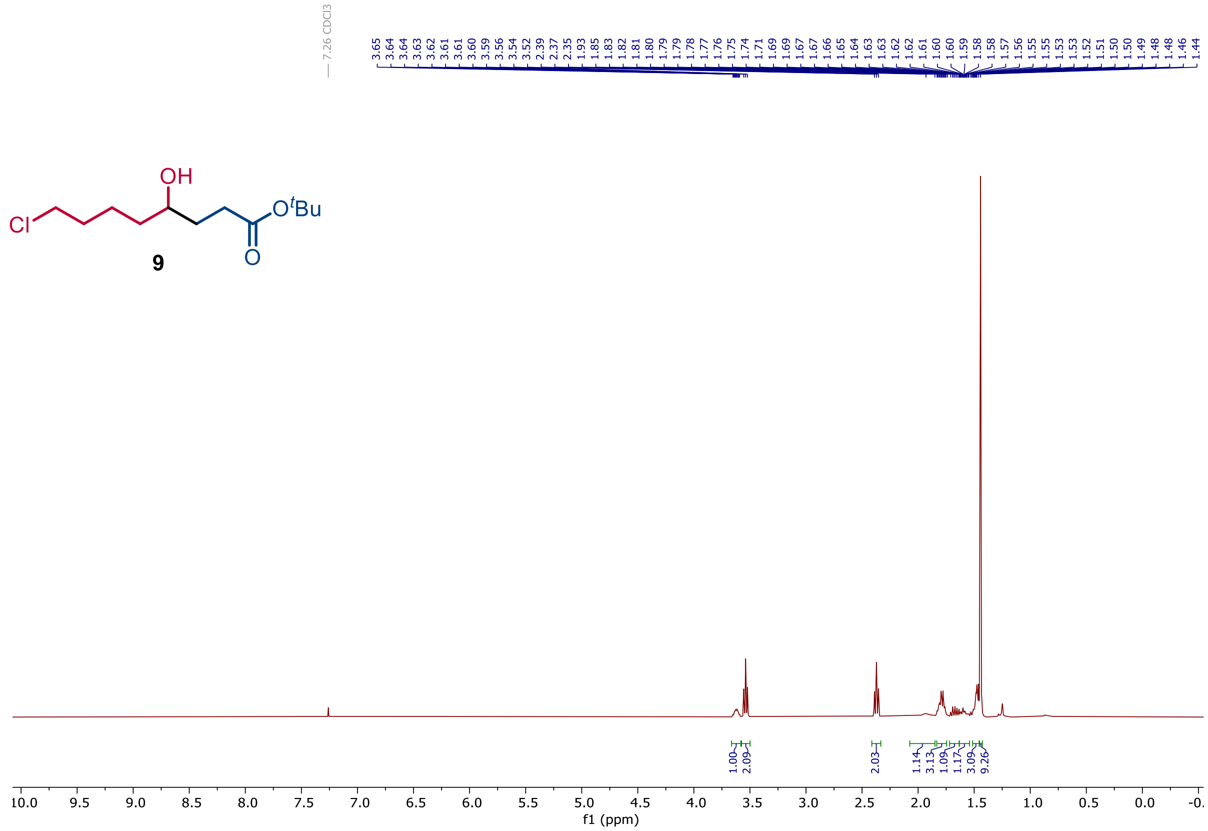

$^{13}C$ NMR (101 MHz, $CDCl_3$) of **9**

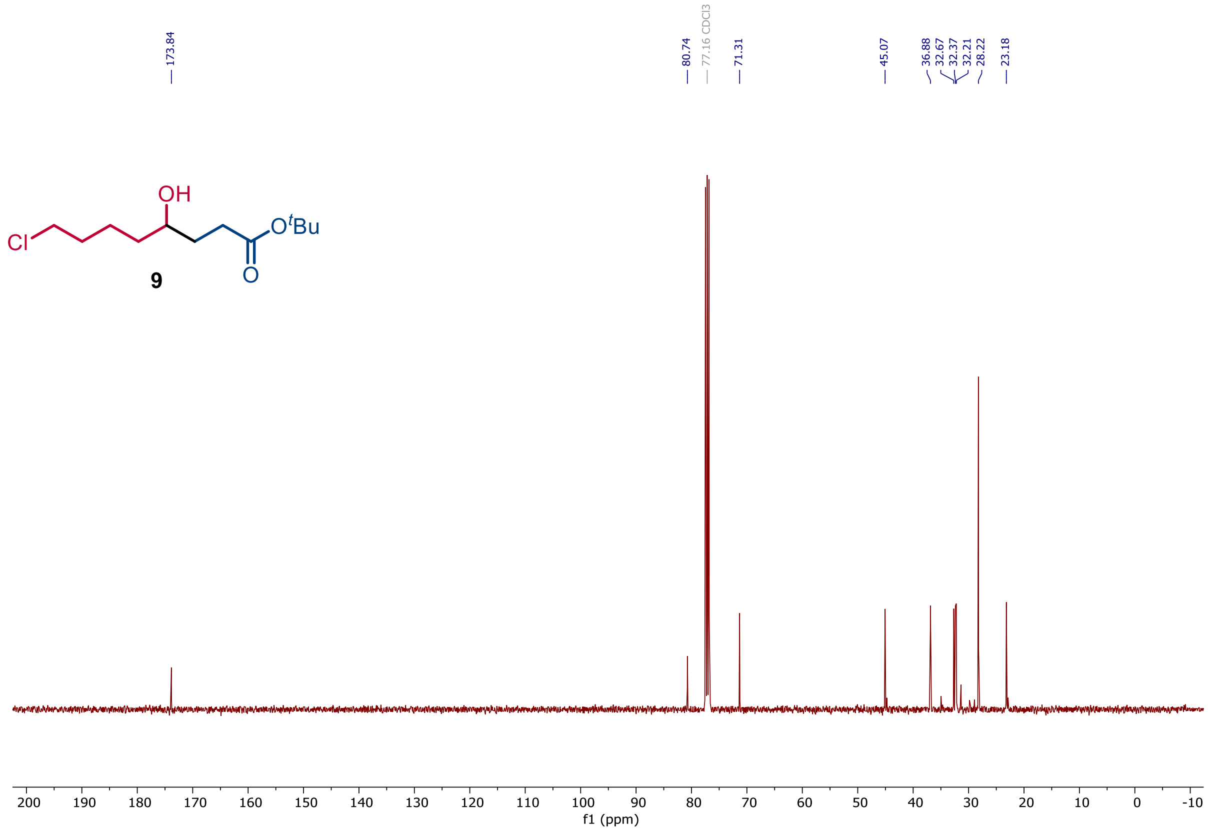

$^{1}$H NMR (500 MHz, $CDCl_3$) of **10** (*see procedure*)

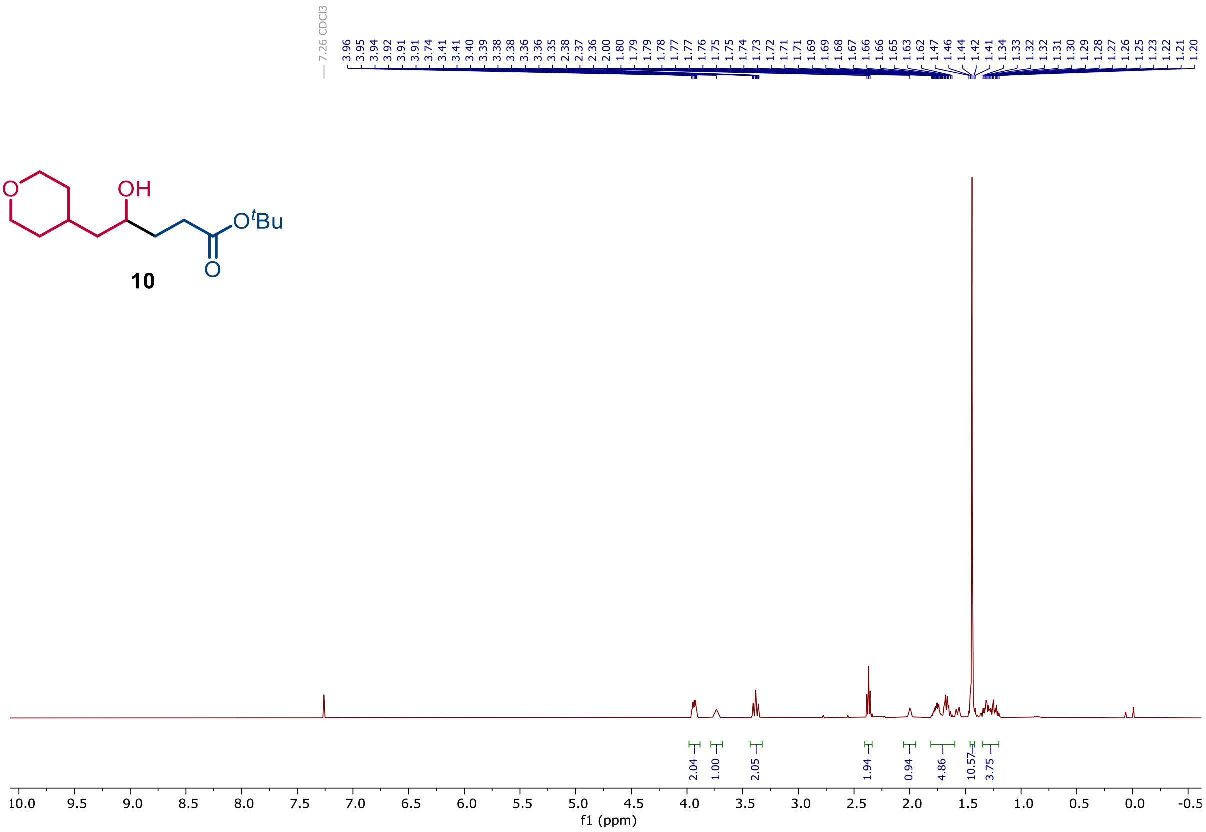

$^{13}$C NMR (126 MHz, $CDCl_3$) of **10**

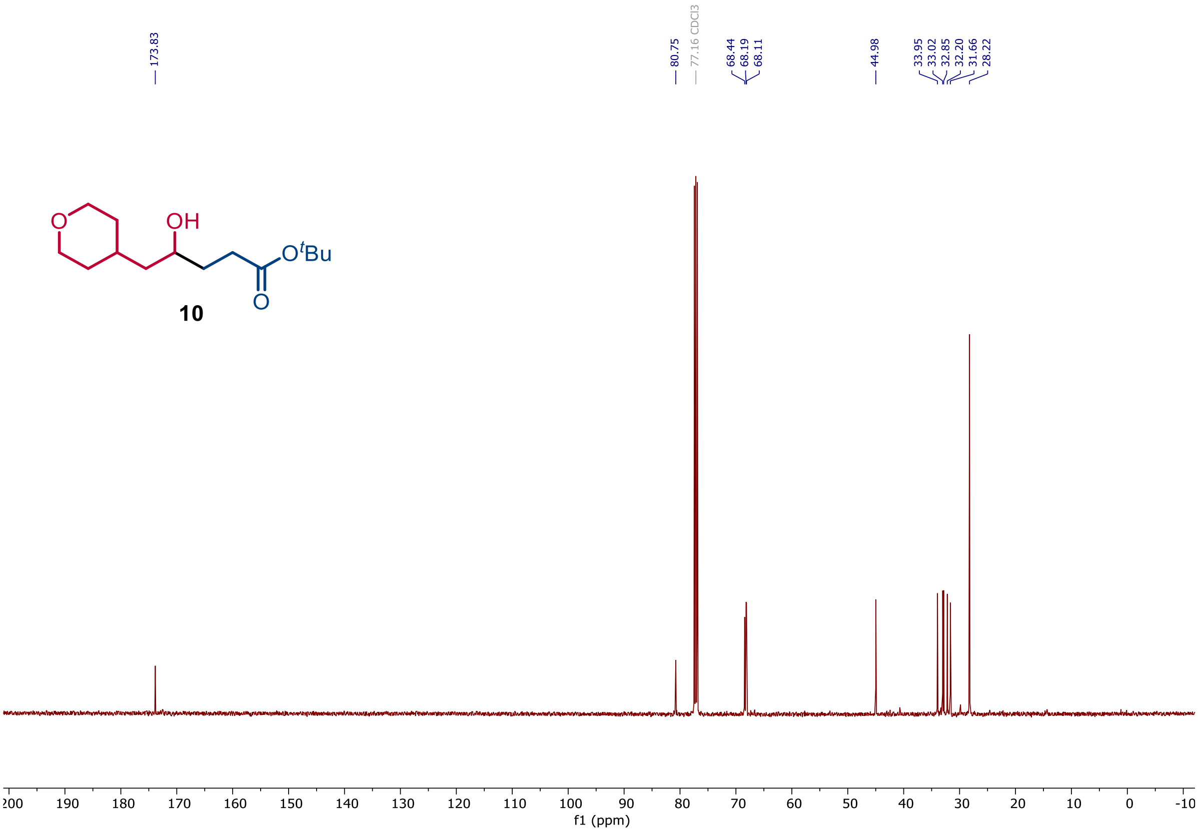

$^{1}$H NMR (400 MHz, $CDCl_3$) of **11** (*see procedure*)

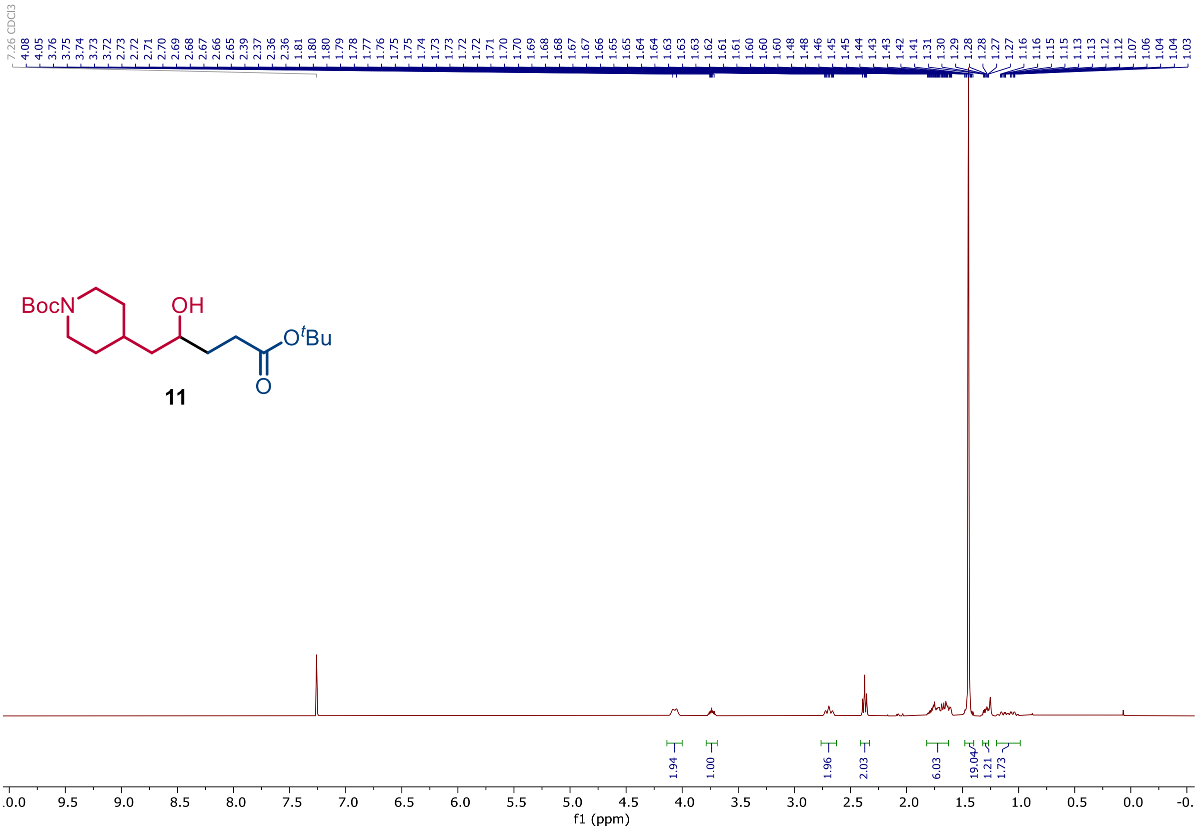

$^{13}$C NMR (101 MHz, $CDCl_3$) of **11**

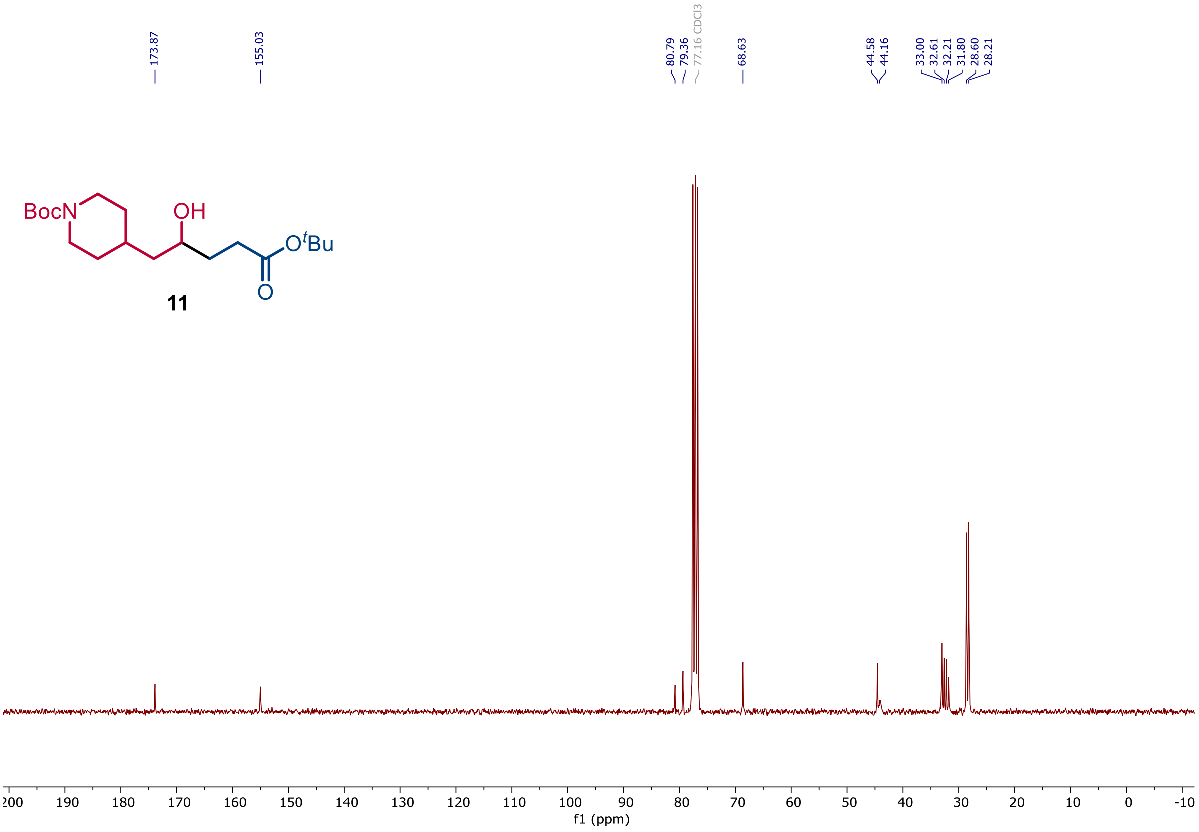

$^{1}$H NMR (400 MHz, $CDCl_3$) of **12** (*see procedure*)

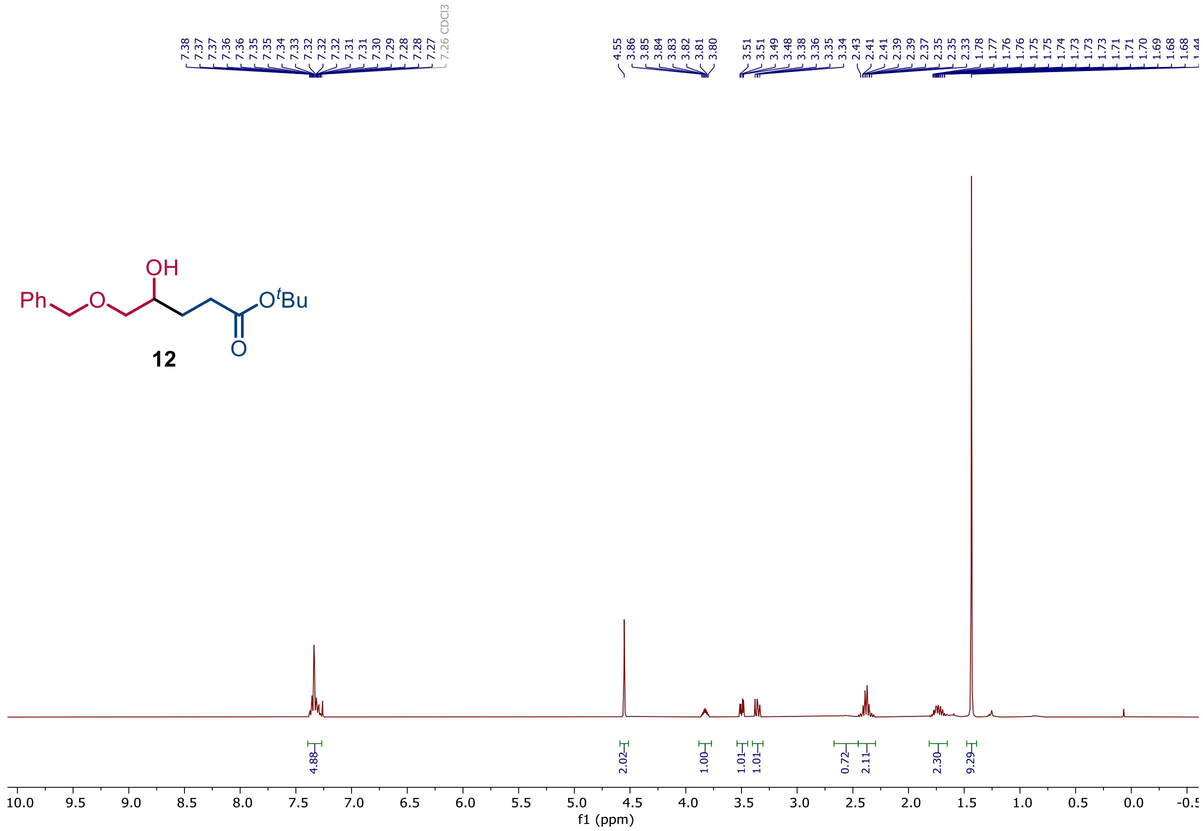


$^{13}$C NMR (101 MHz, $CDCl_3$) of **12**

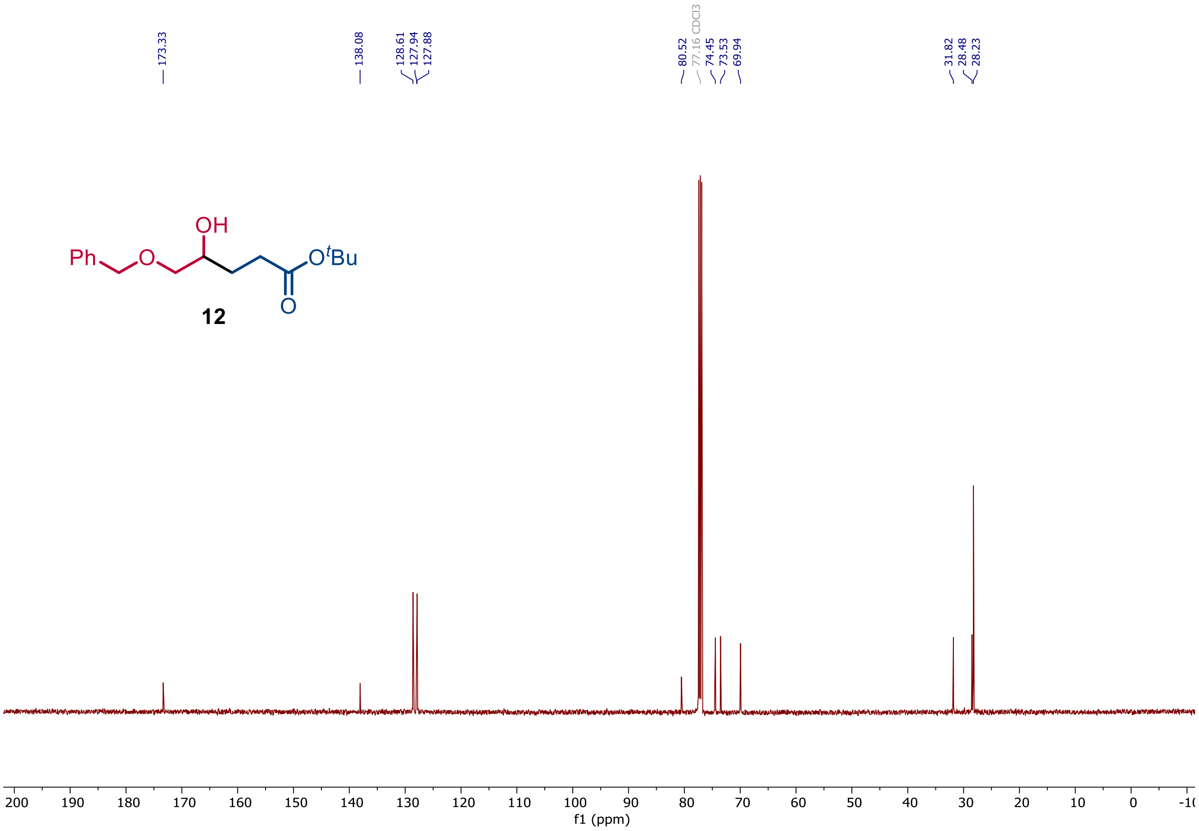

$^{1}H$ NMR (400 MHz, $CDCl_3$) of **13** (*see procedure*)

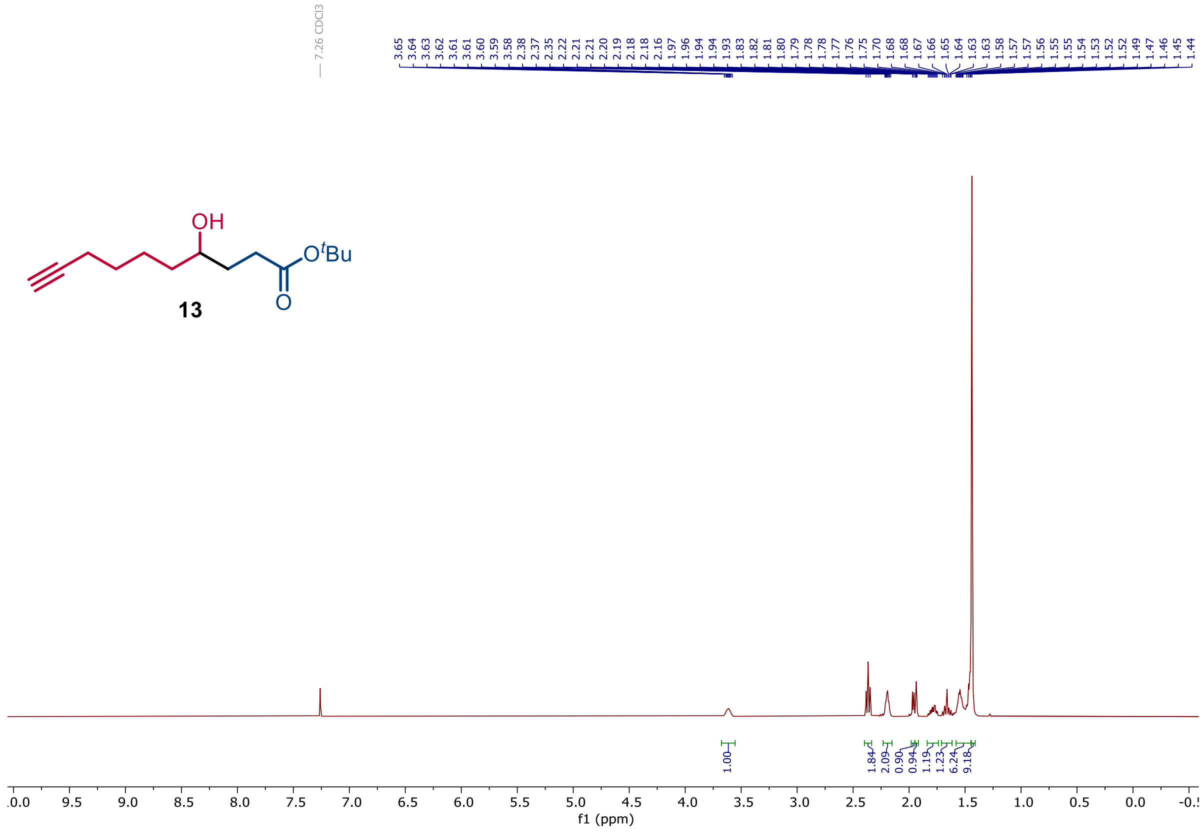


$^{13}C$ NMR (101 MHz, $CDCl_3$) of **13**

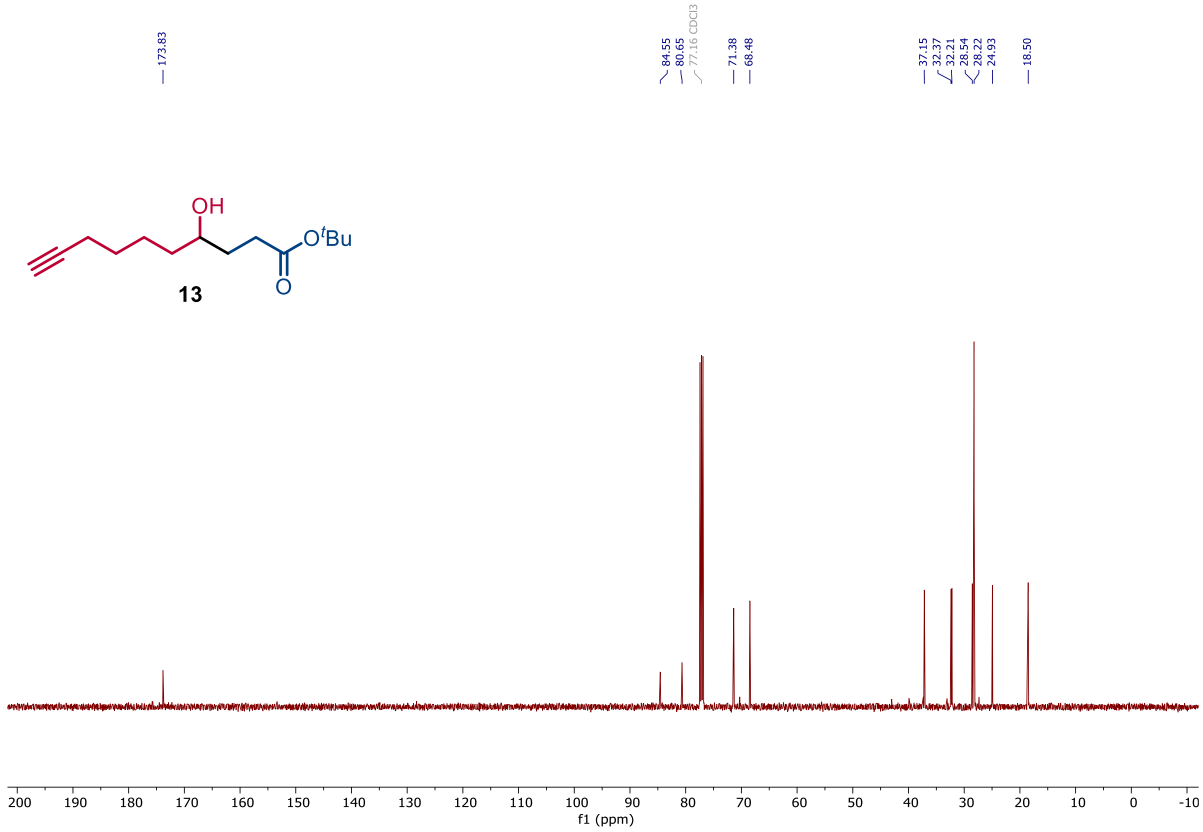

$^1$H NMR (400 MHz, $CDCl_3$) of **14** (diastereomer 1) (*see procedure*)

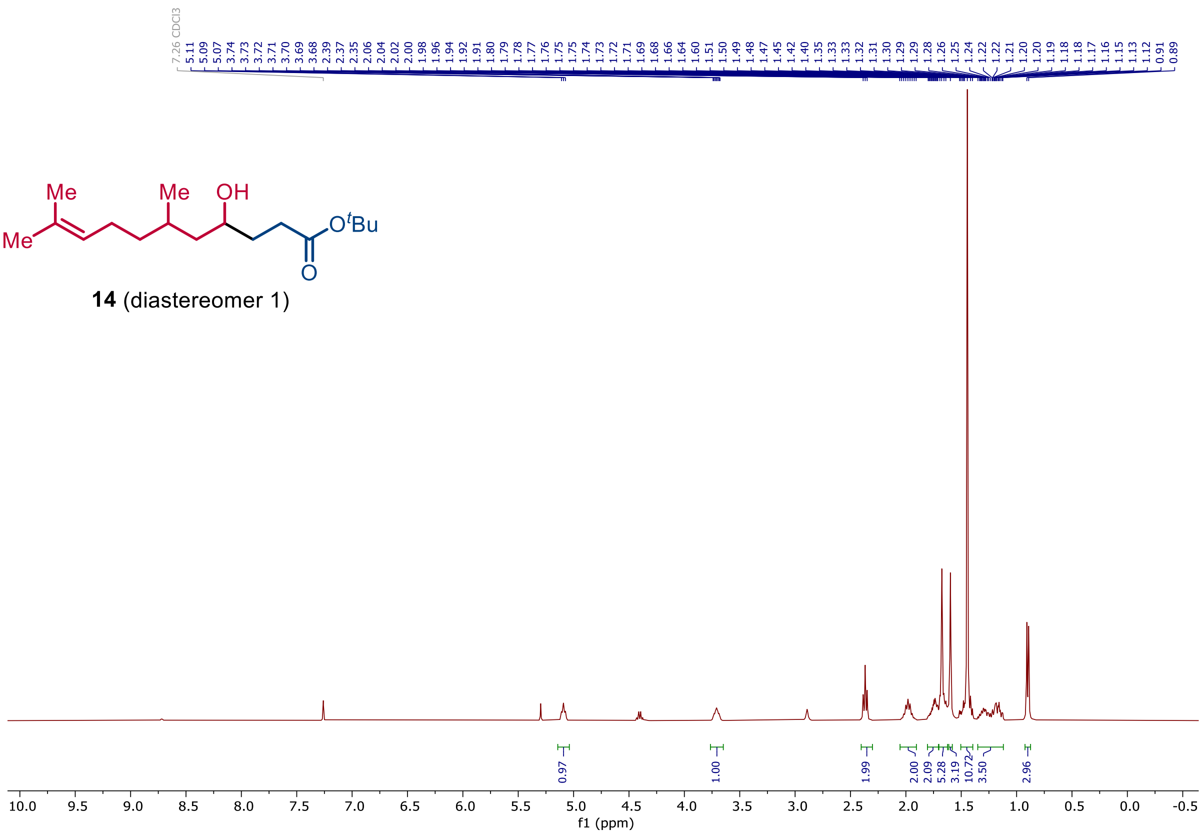


$^{13}$C NMR (101 MHz, $CDCl_3$) of **14** (diastereomer 1)

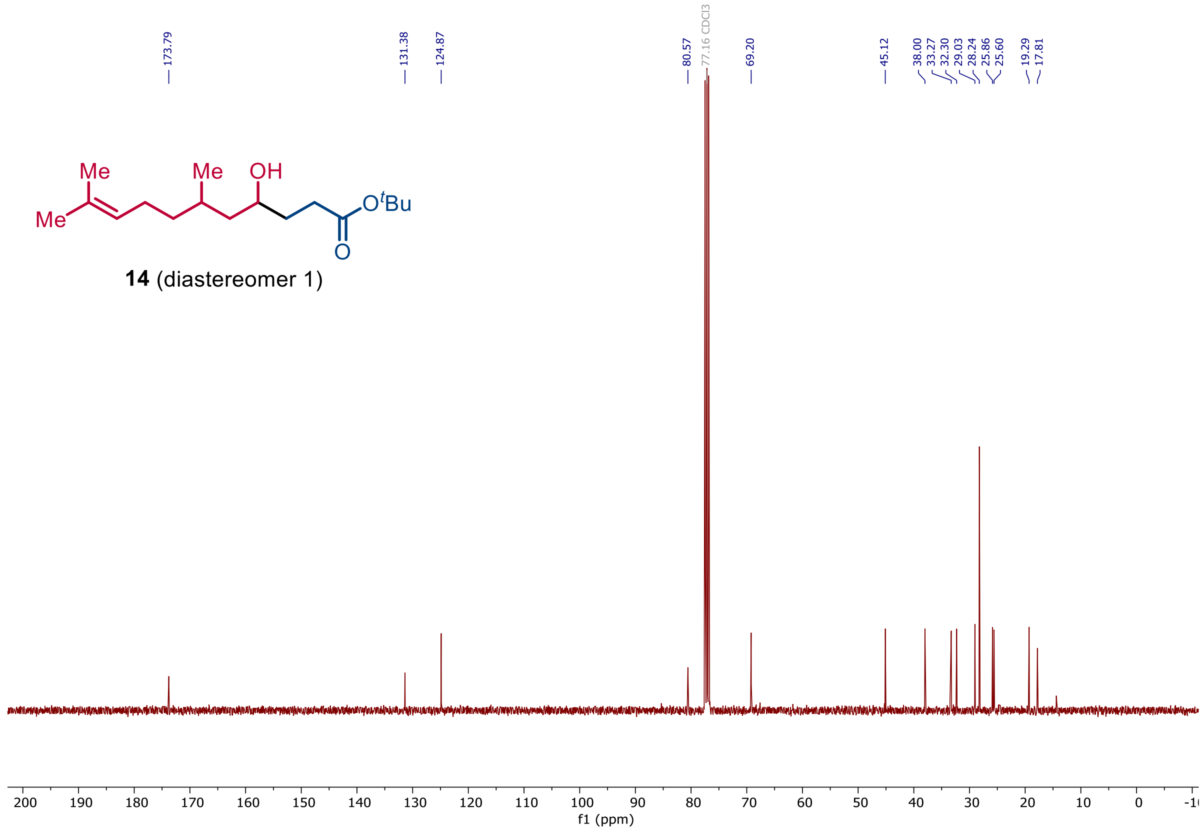

$^{1}$H NMR (400 MHz, $CDCl_3$) of **14** (diastereomer 2) (*see procedure*)

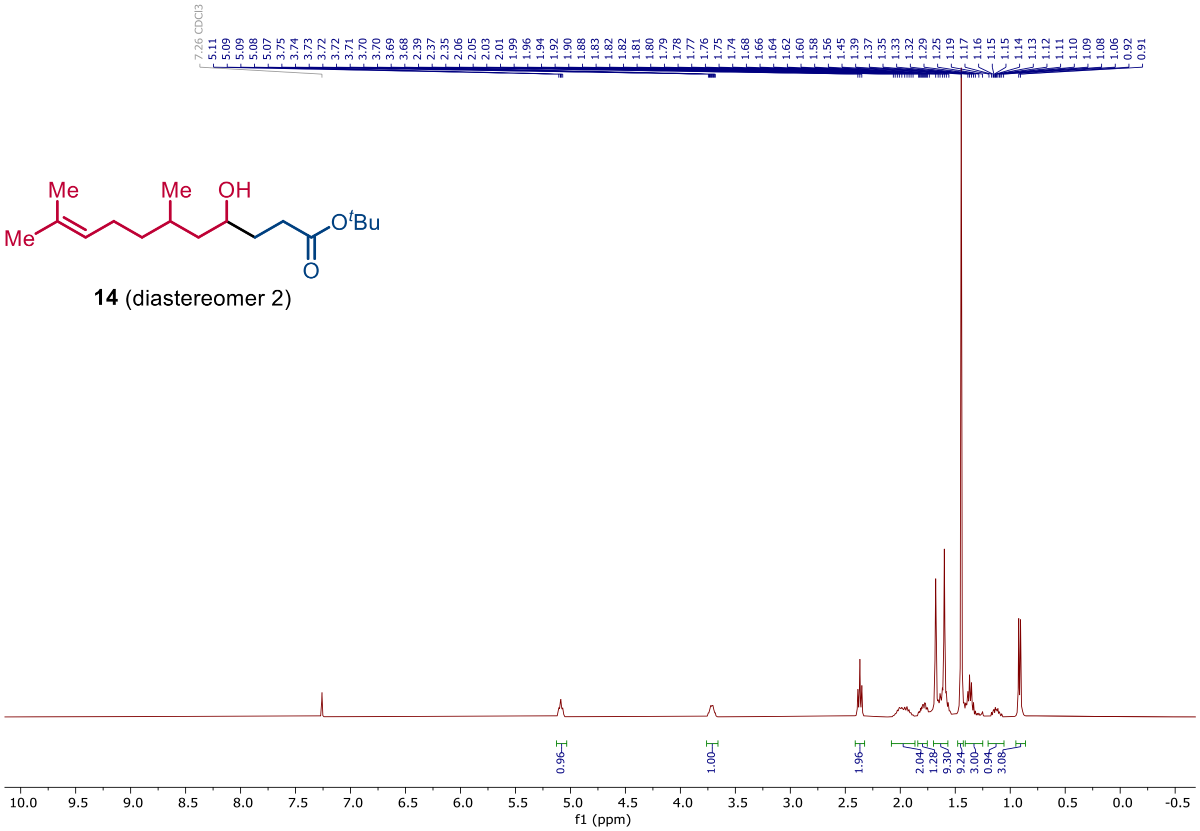


$^{13}$C NMR (101 MHz, $CDCl_3$) of **14** (diastereomer 2)

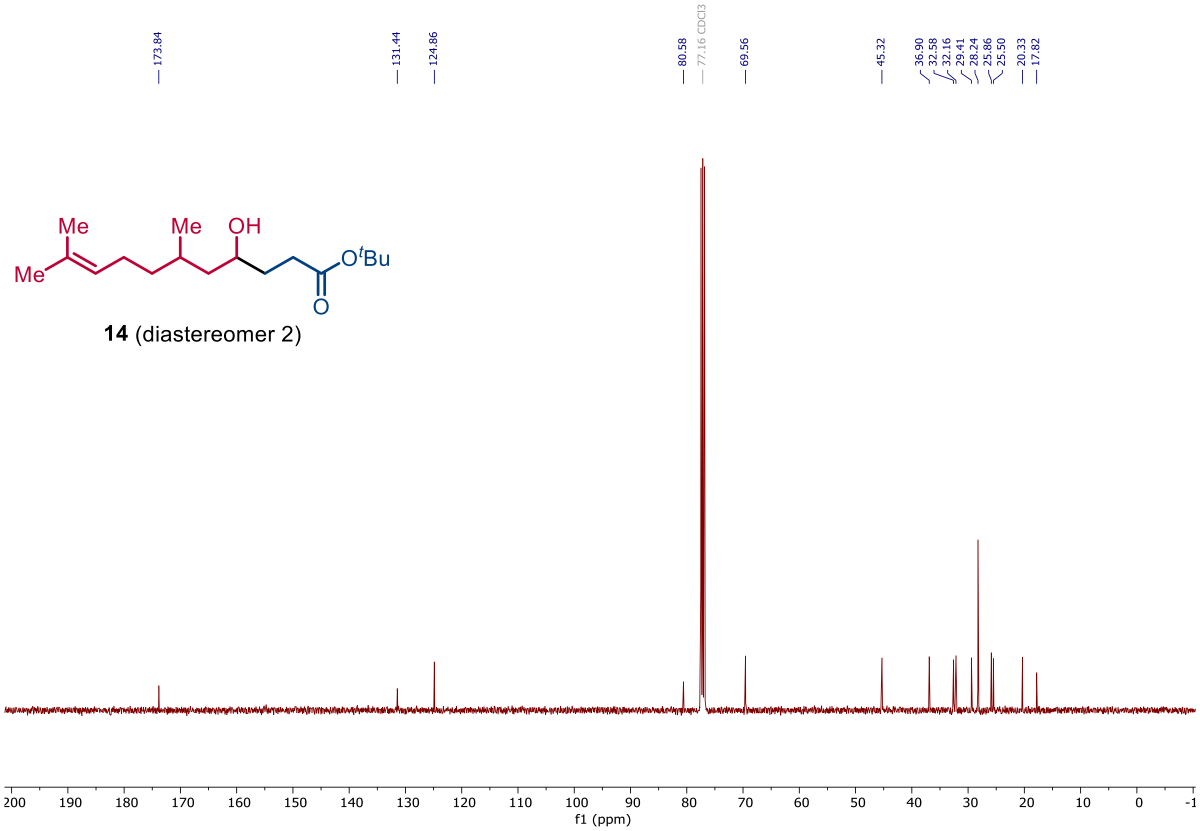

$^{1}$H NMR (400 MHz, $CDCl_3$) of **15** (*see procedure*)

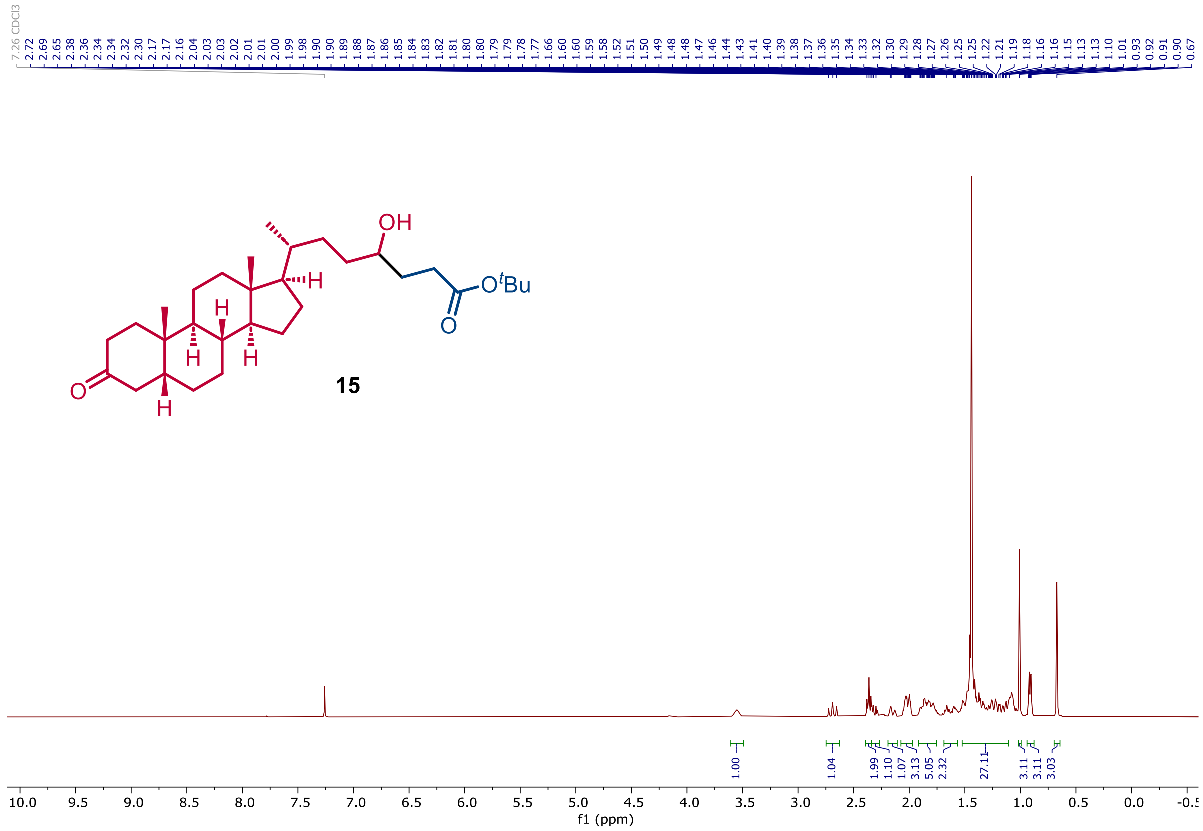


$^{13}$C NMR (101 MHz, $CDCl_3$) of **15**

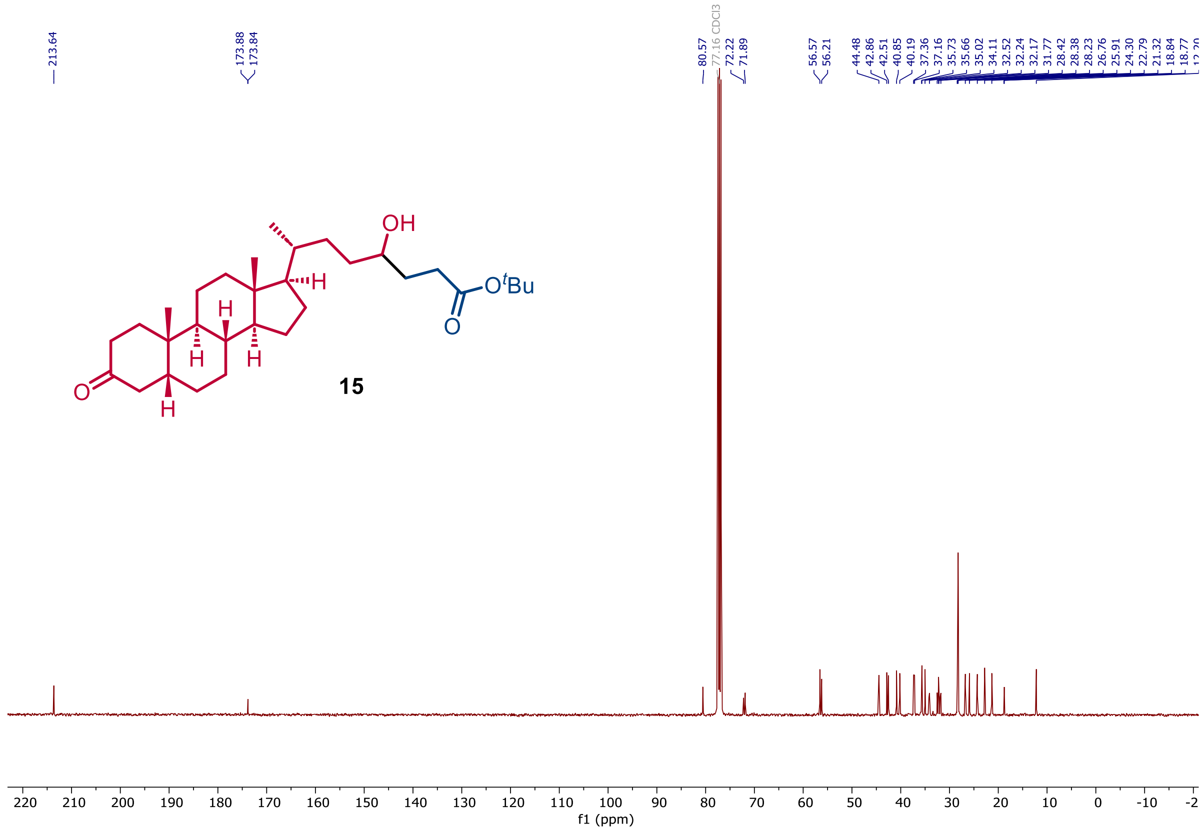

$^1$H NMR (400 MHz, $CDCl_3$) of **16** (*see procedure*)

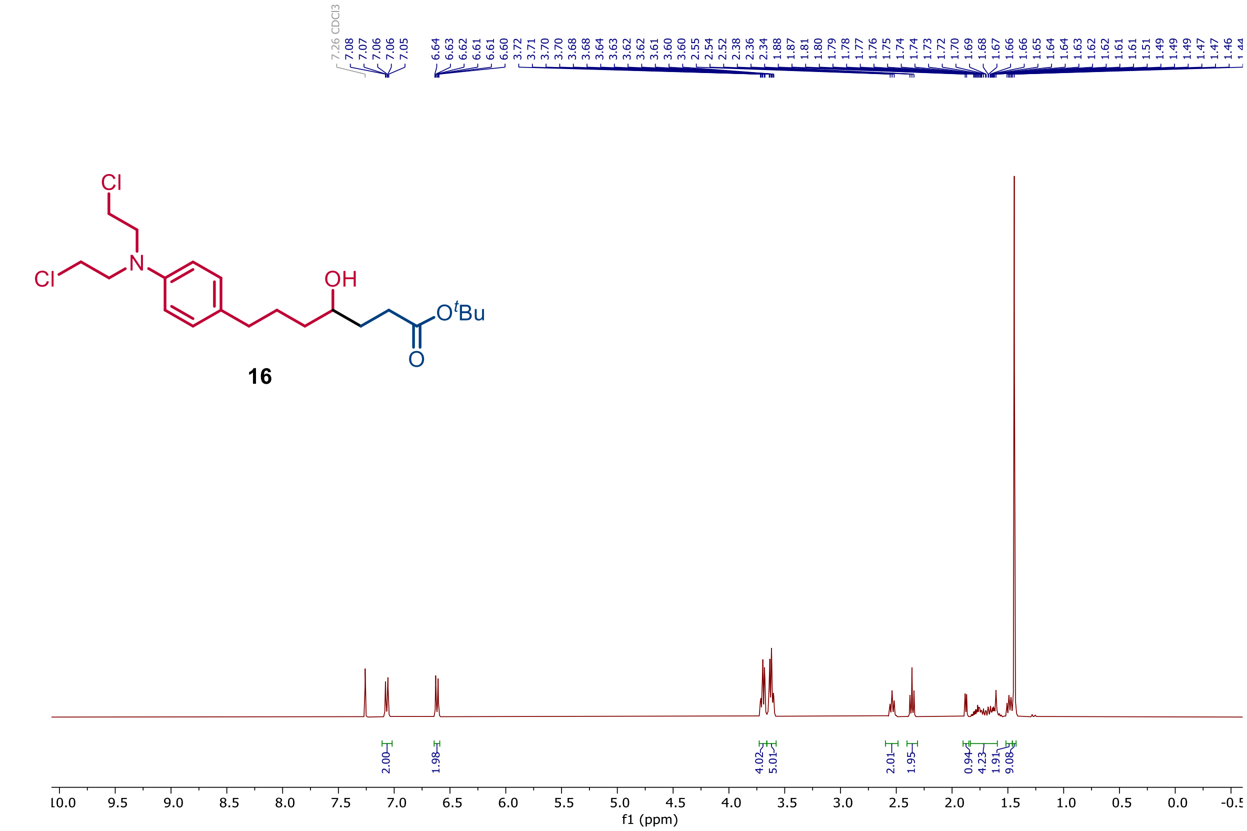


$^{13}$C NMR (101 MHz, $CDCl_3$) of **16**

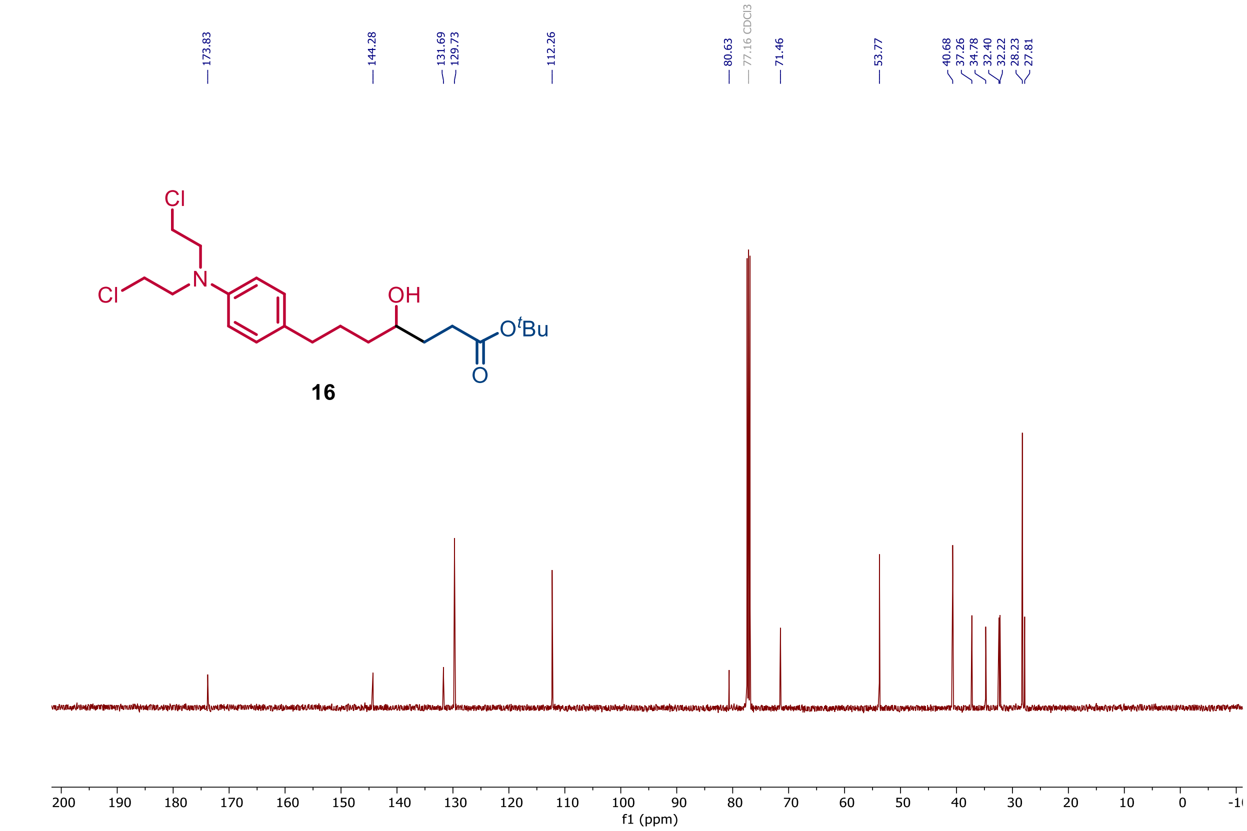

$^{1}$H NMR (400 MHz, $CDCl_3$) of **17** (*see procedure*)

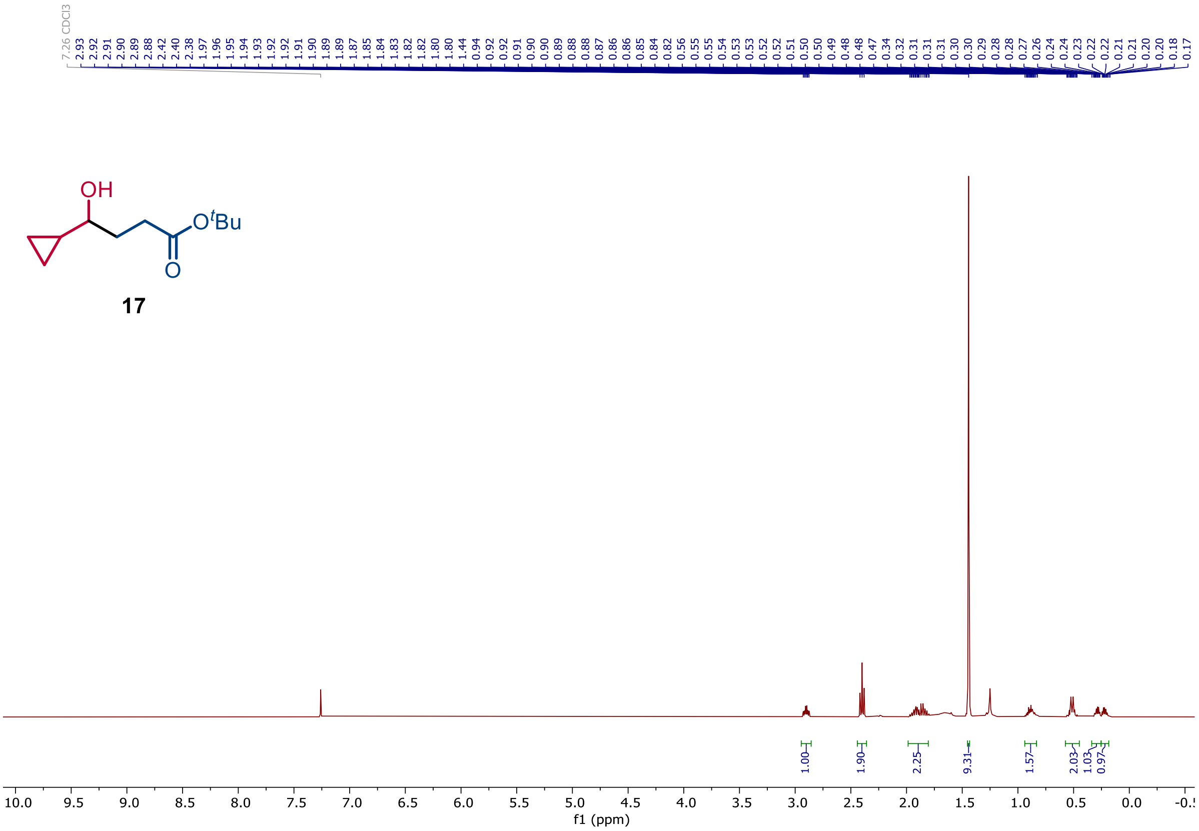


$^{13}$C NMR (101 MHz, $CDCl_3$) of **17**

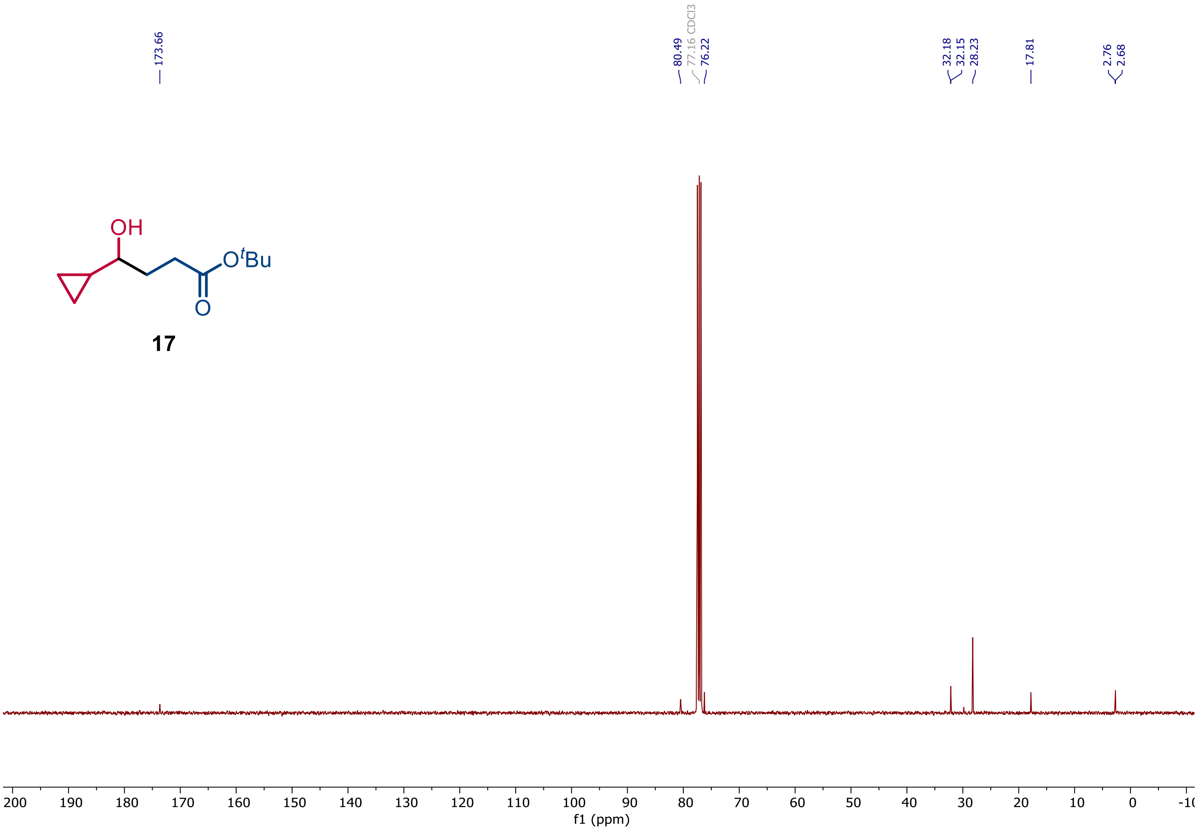

$^1$H NMR (400 MHz, $CDCl_3$) of **18** (see procedure)

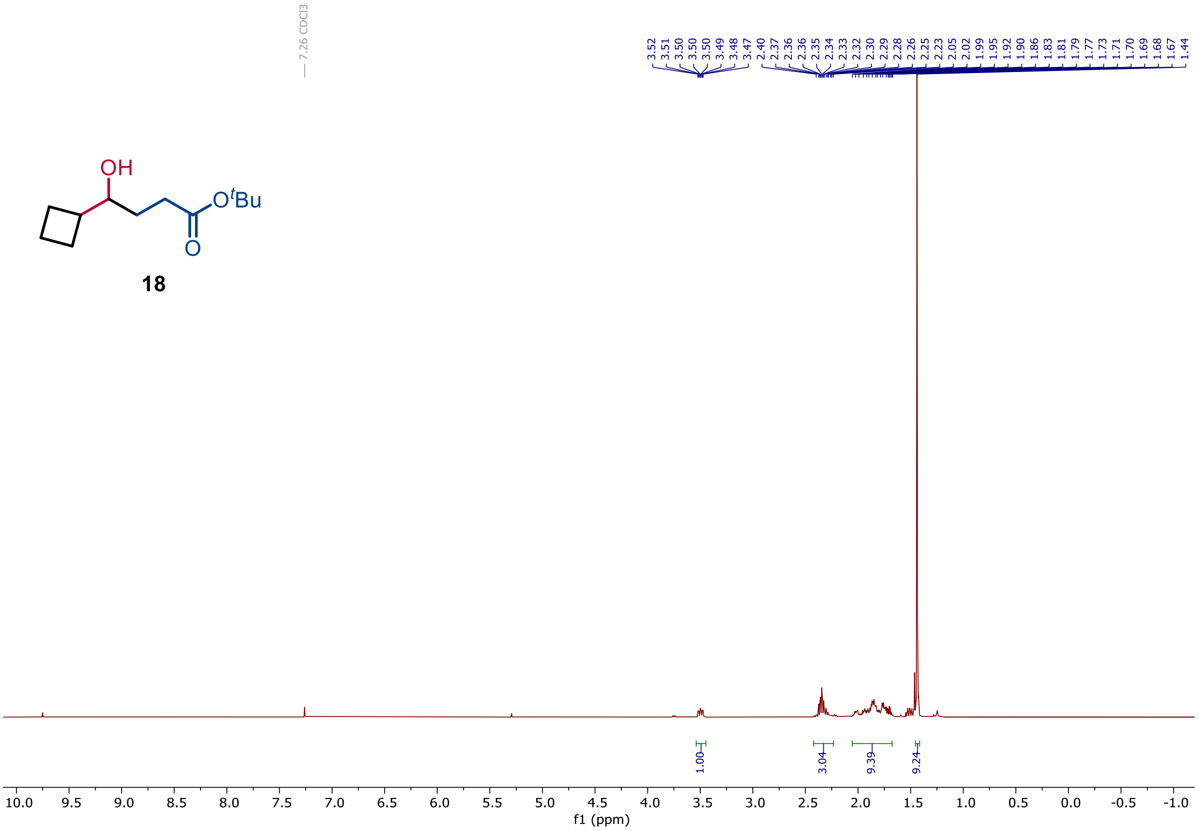


$^{13}$C NMR (101 MHz, $CDCl_3$) of **18**

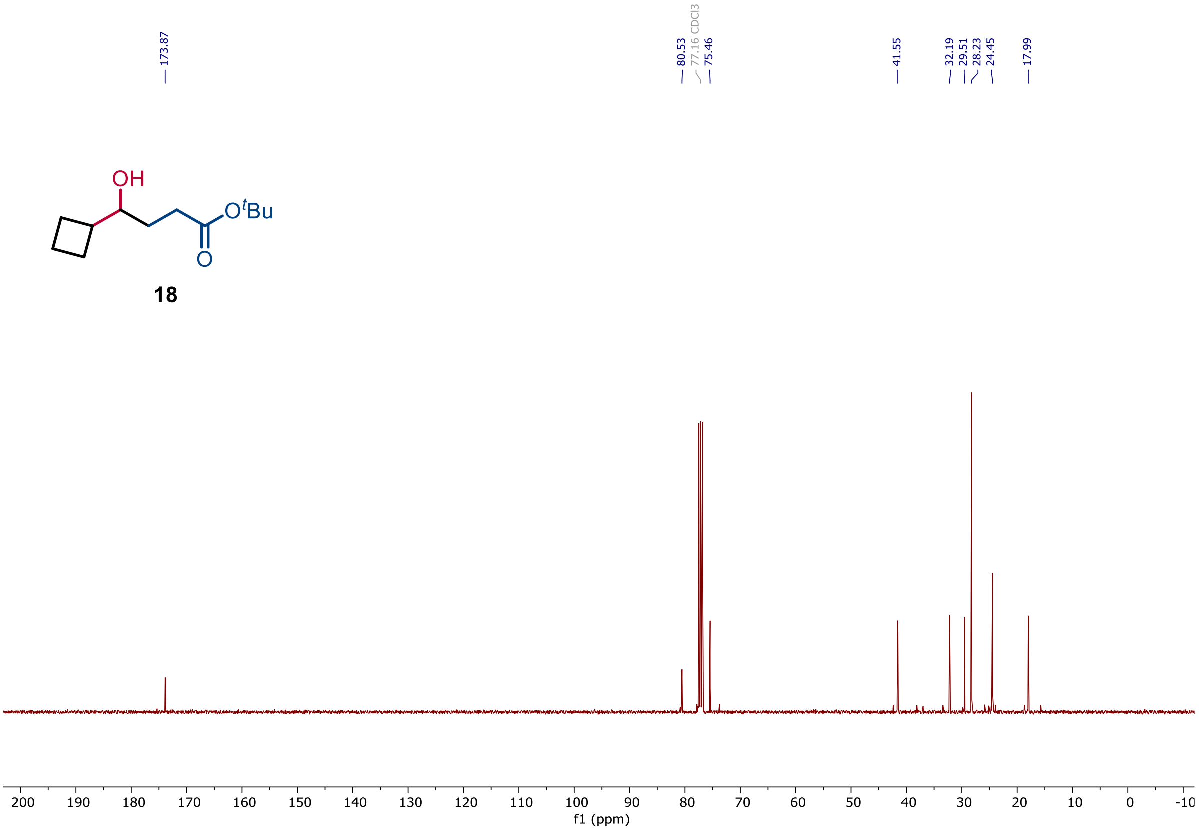

$^{1}$H NMR (400 MHz, $CDCl_3$) of **19** (*see procedure*)

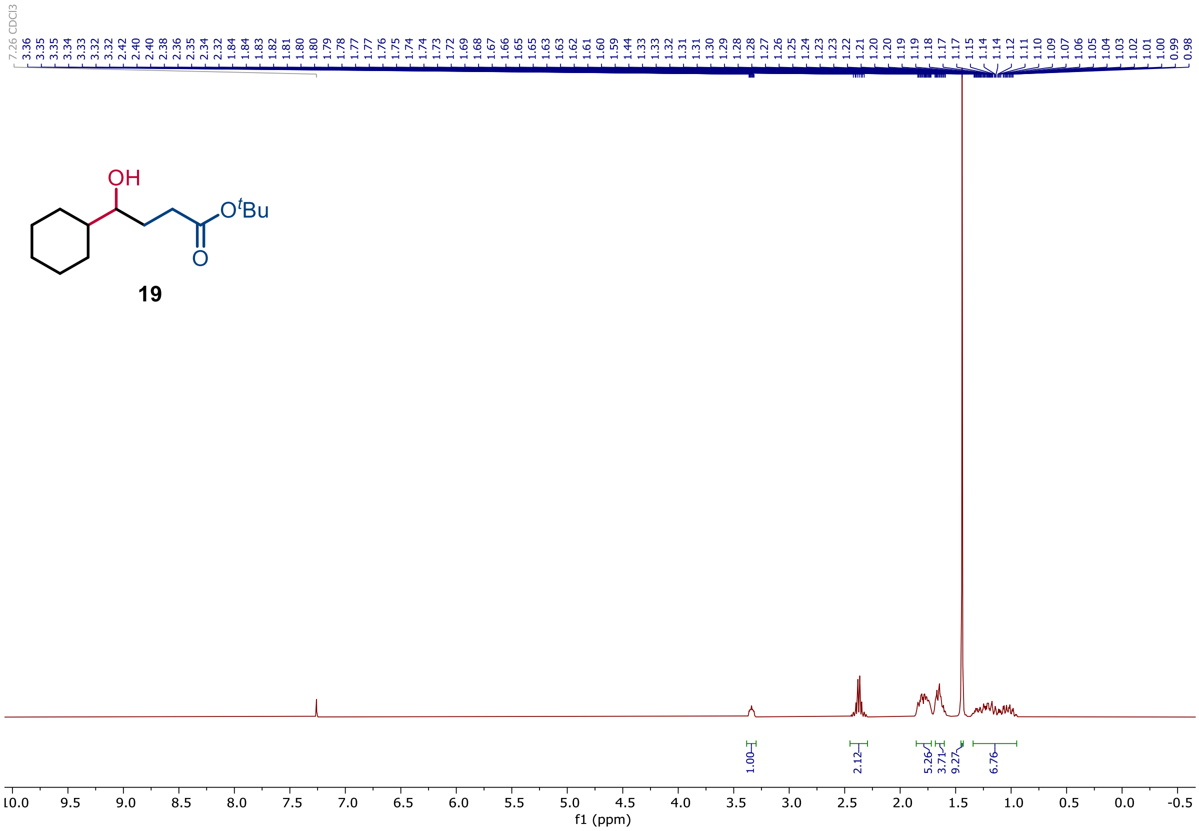


$^{13}$C NMR (101 MHz, $CDCl_3$) of **19**

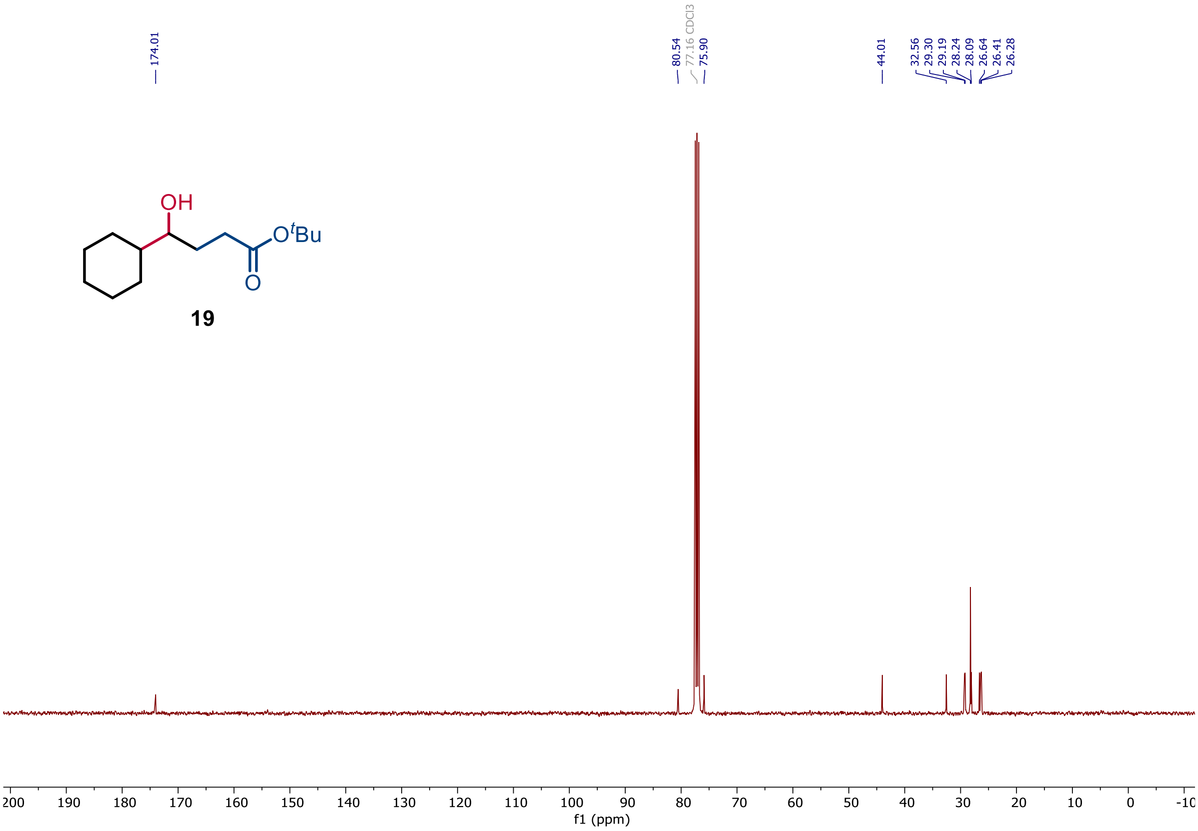

$^{1}$H NMR (400 MHz, $CDCl_3$) of **20** (<u>*see procedure*</u>)

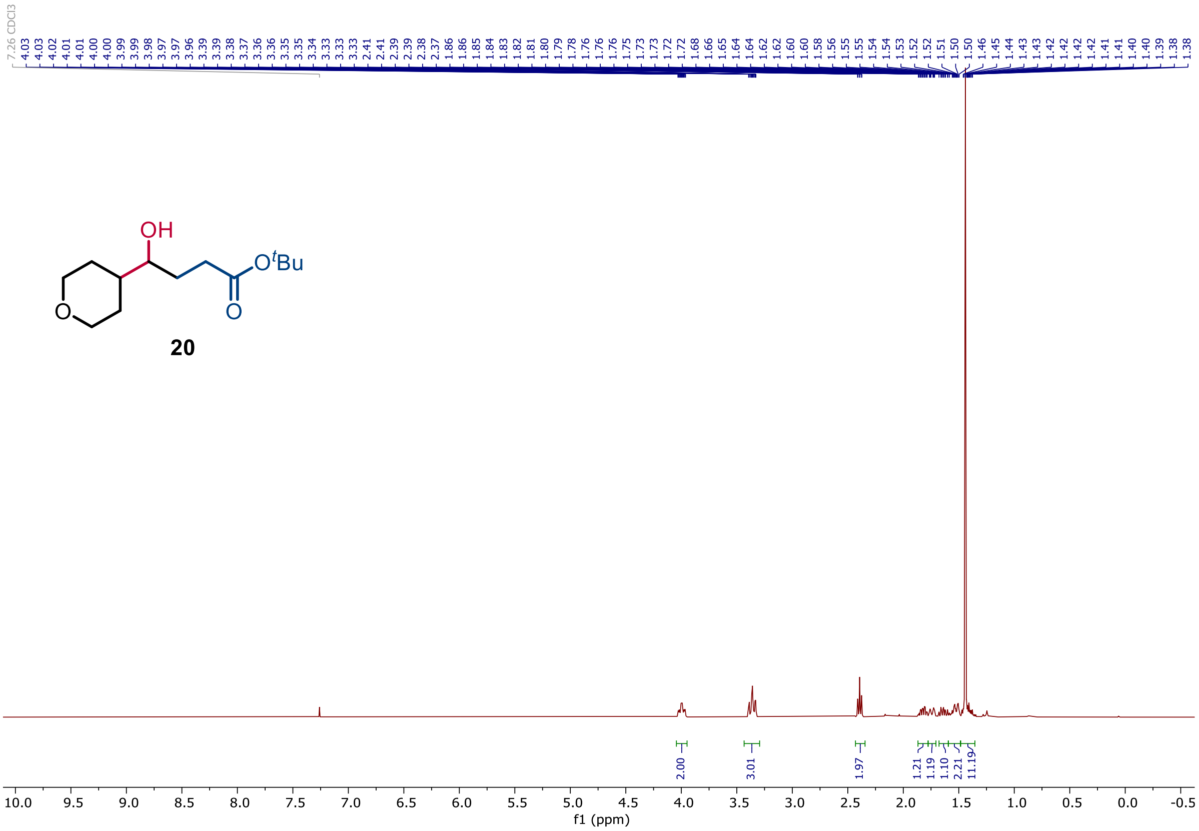

$^{13}$C NMR (101 MHz, $CDCl_3$) of **20**

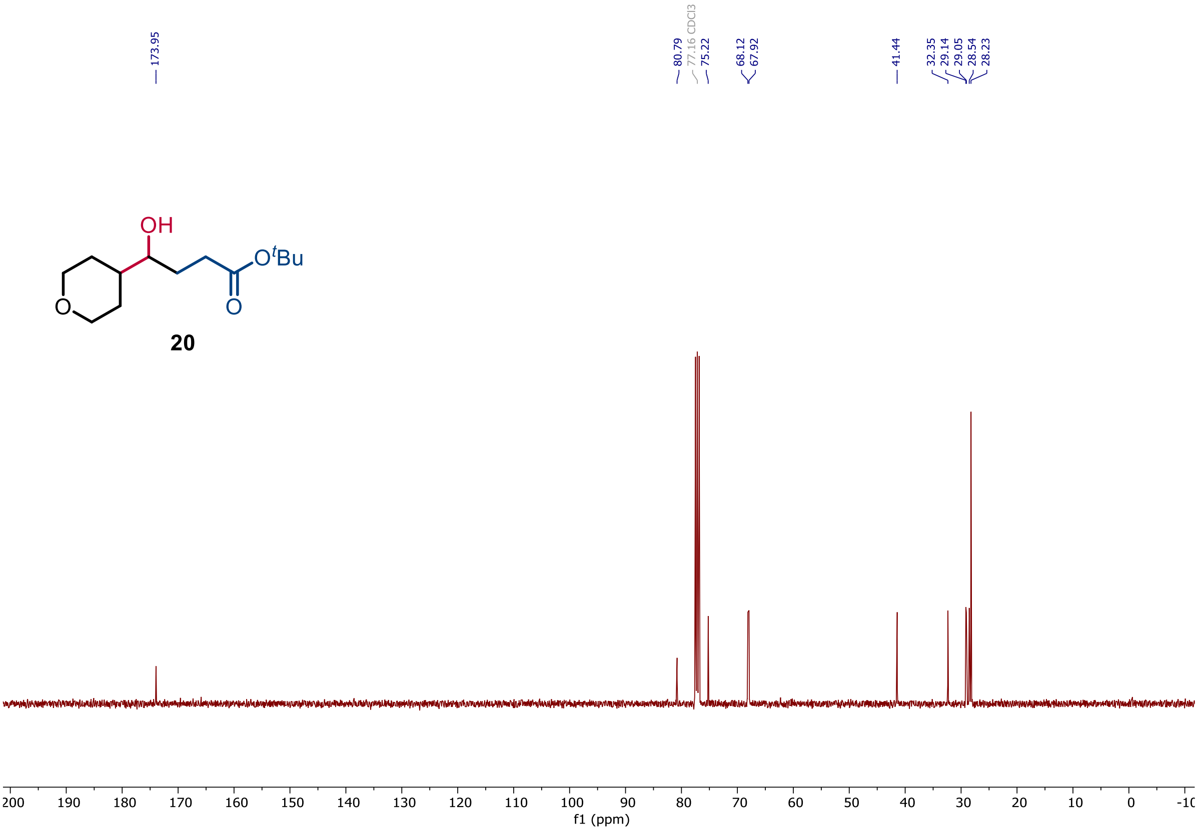

$^{1}$H NMR (400 MHz, $CDCl_3$) of **21** (*see procedure*)

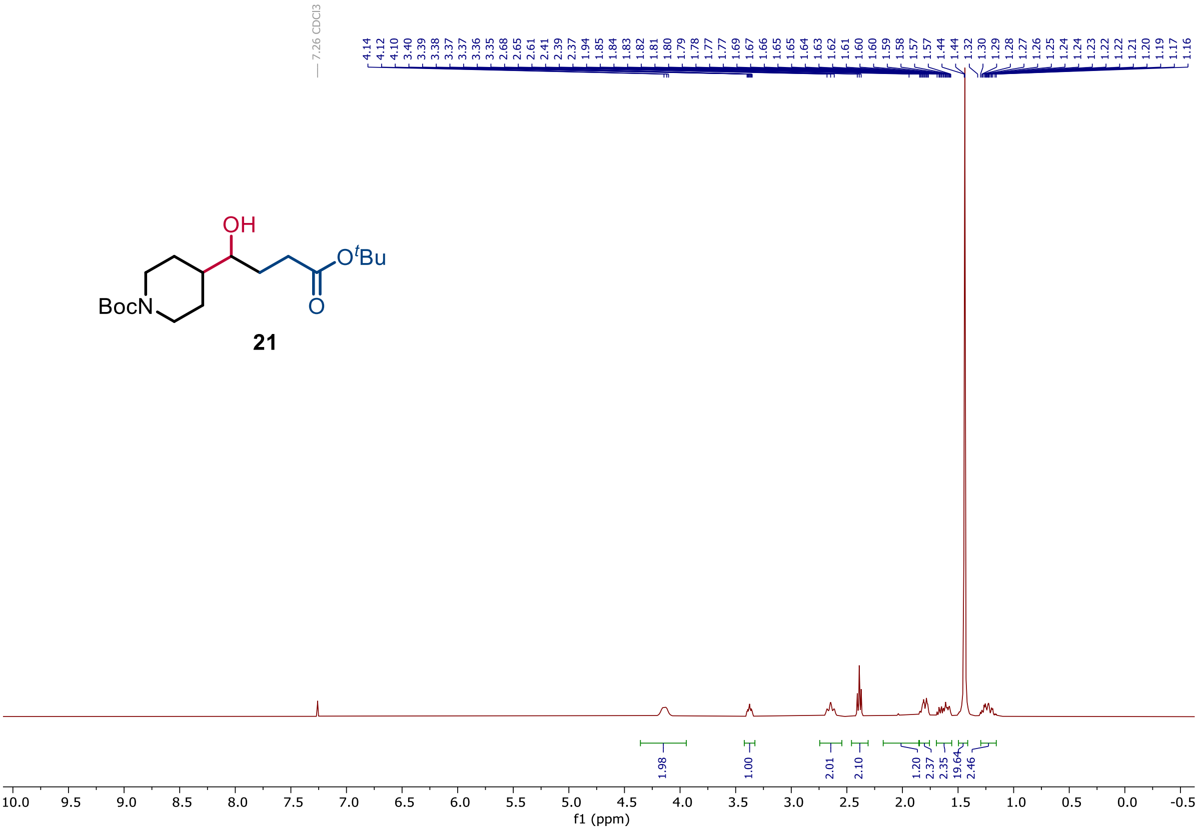


$^{13}$C NMR (101 MHz, $CDCl_3$) of **21**

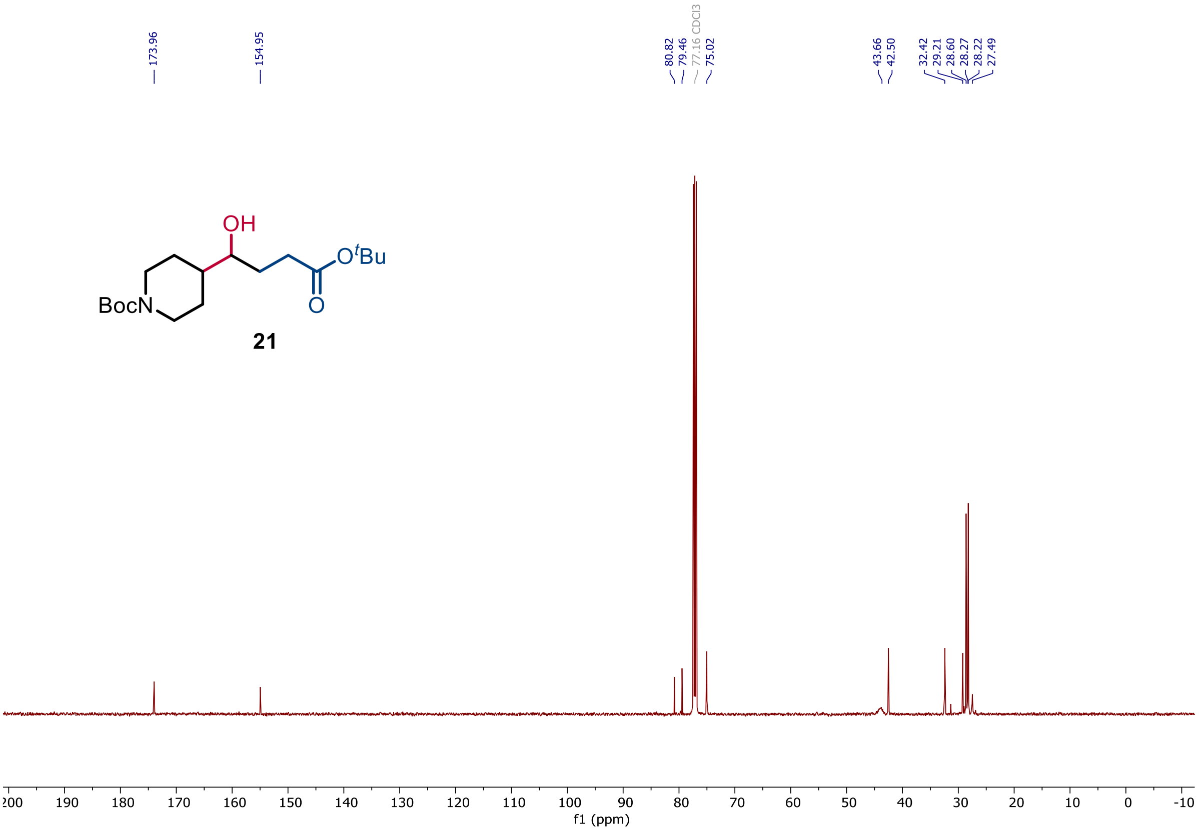

$^{1}H$ NMR (400 MHz, $CDCl_3$) of **22** (see procedure)

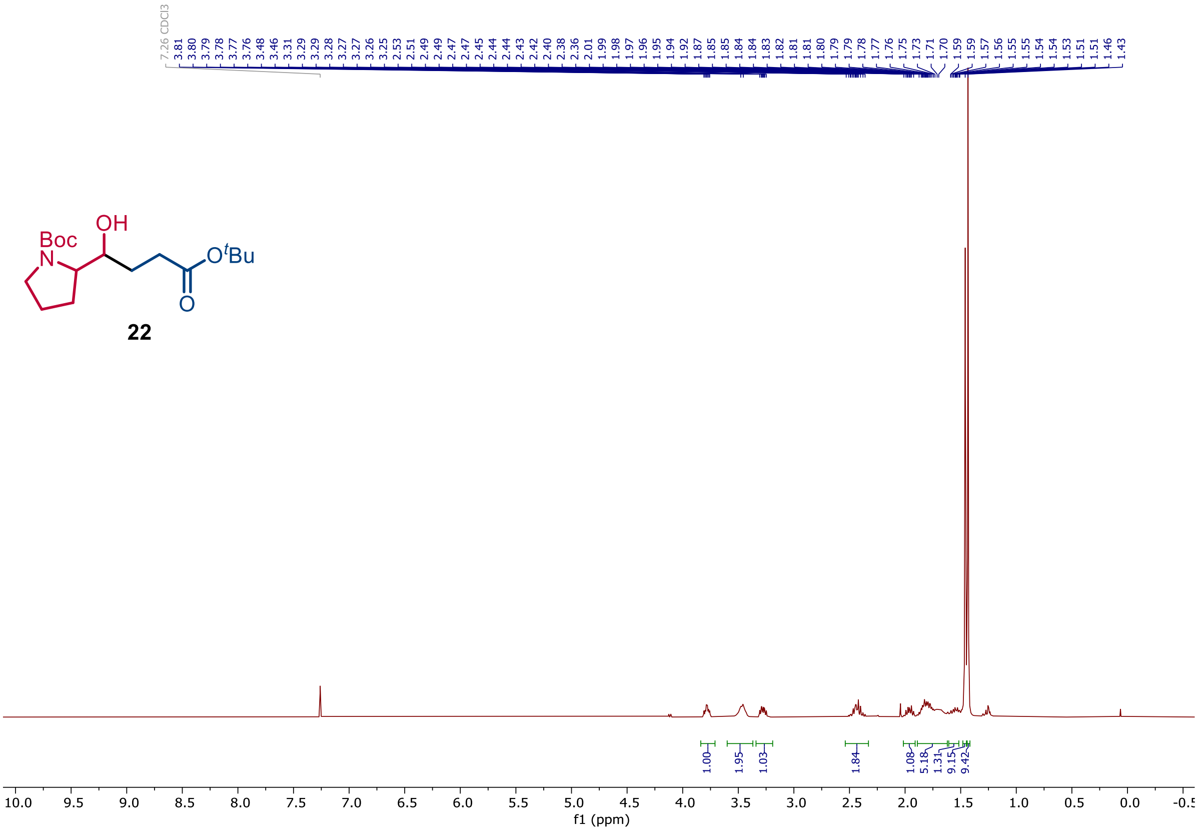


$^{13}C$ NMR (101 MHz, $CDCl_3$) of **22**

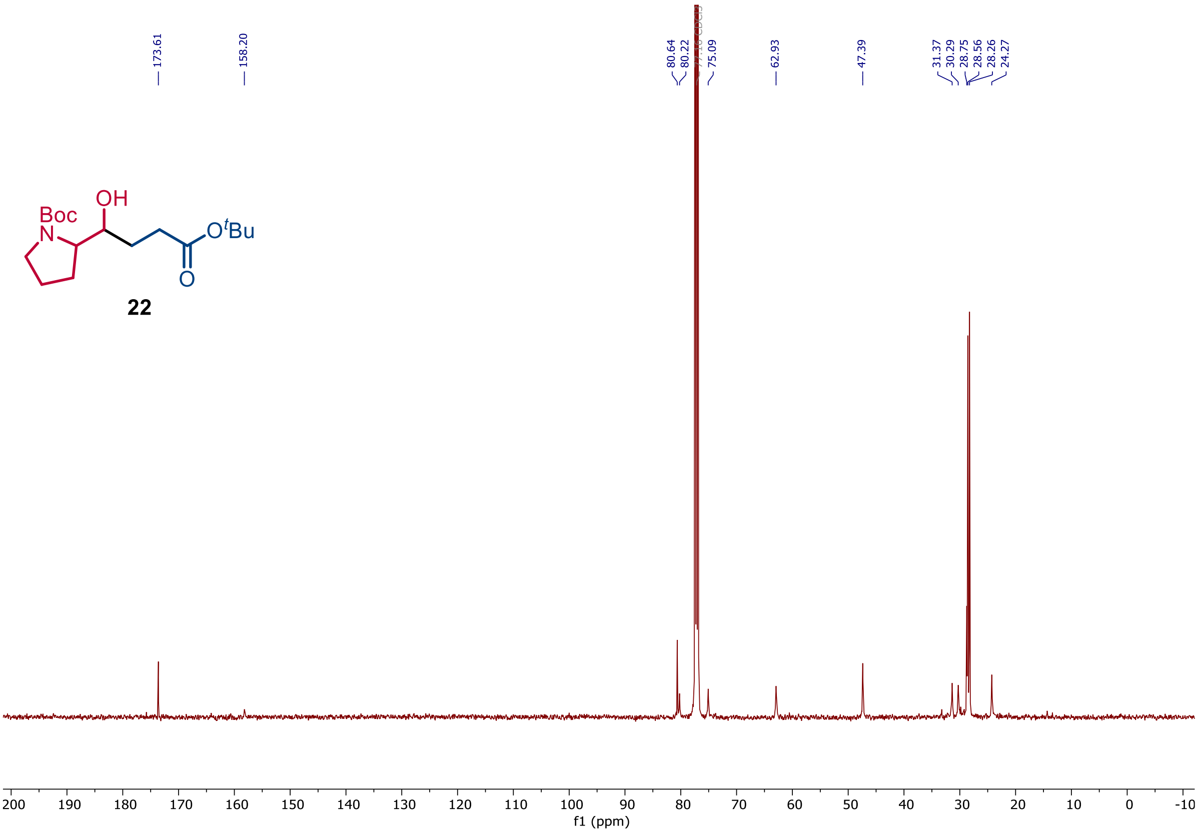

$^{1}$H NMR (500 MHz, CDCl$_3$) of **23** (*see procedure*)

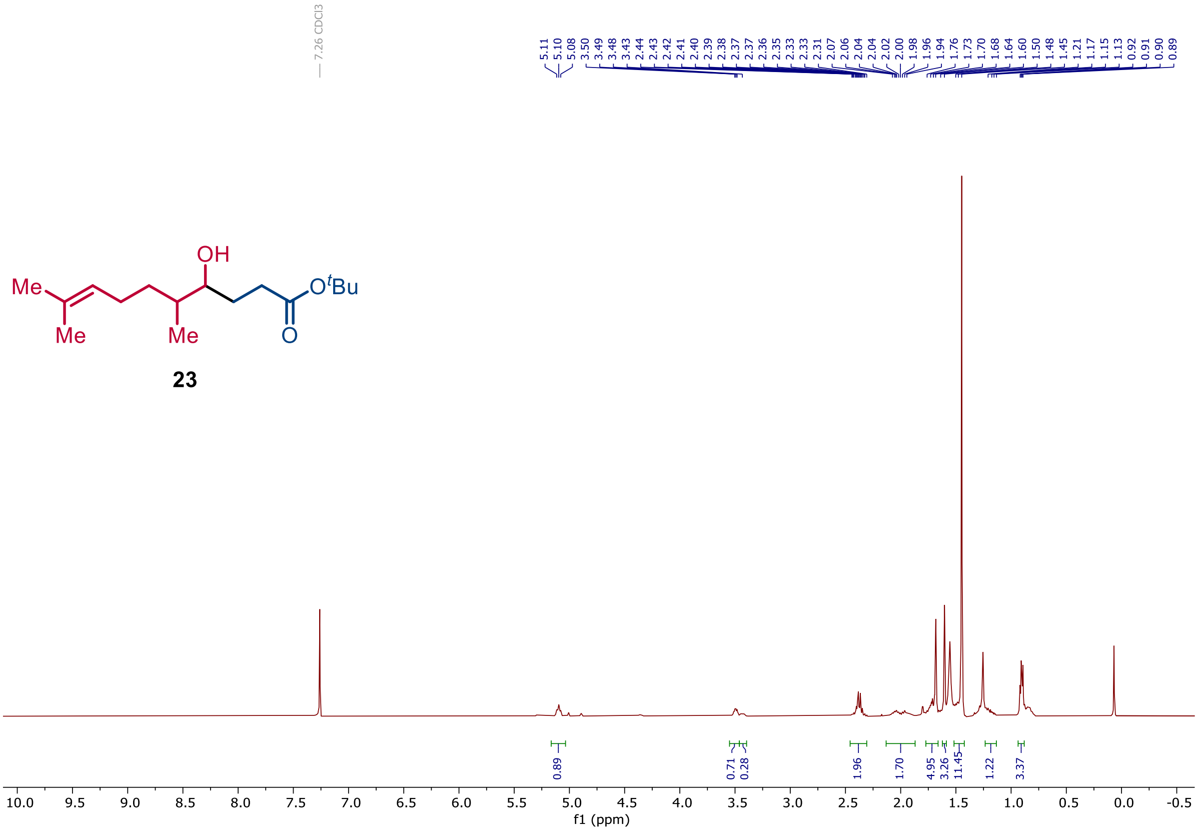


$^{13}$C NMR (126 MHz, CDCl$_3$) of **23**

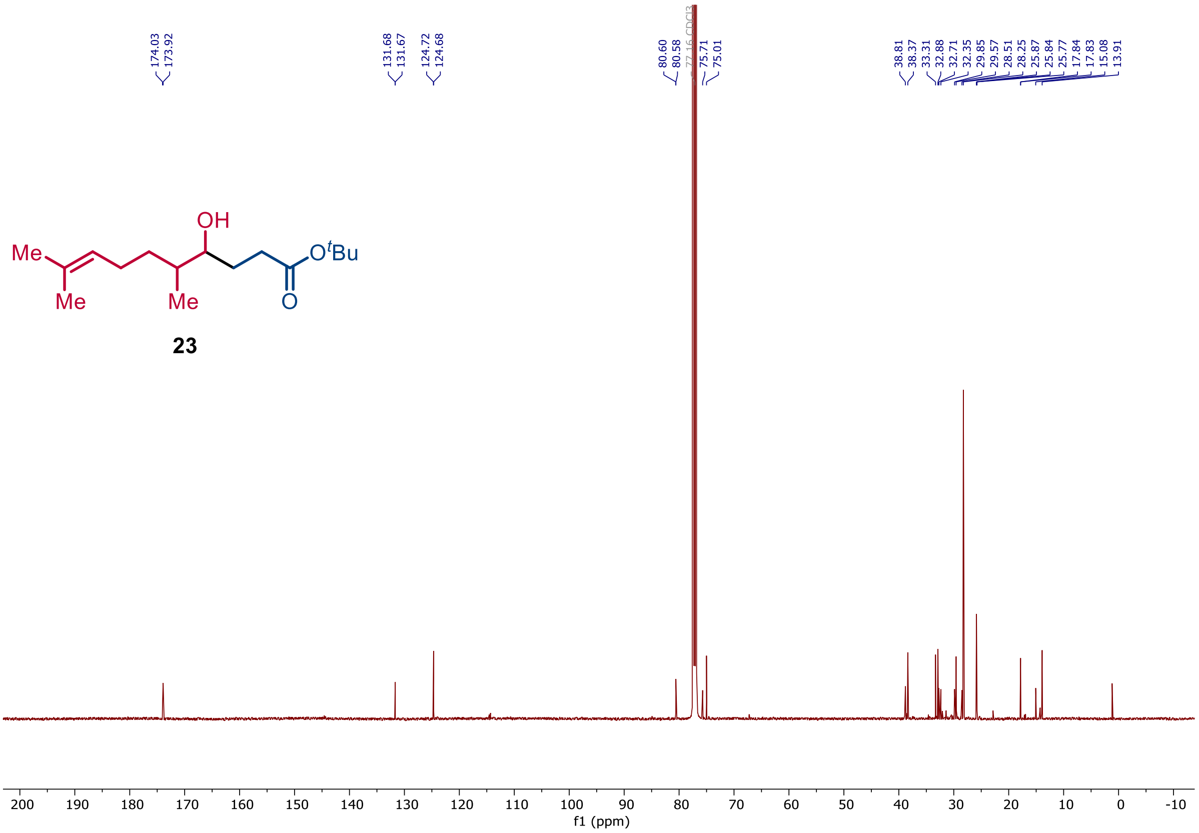

$^1$H NMR (400 MHz, CDCl$_3$) of crude **24** (*see procedure*)

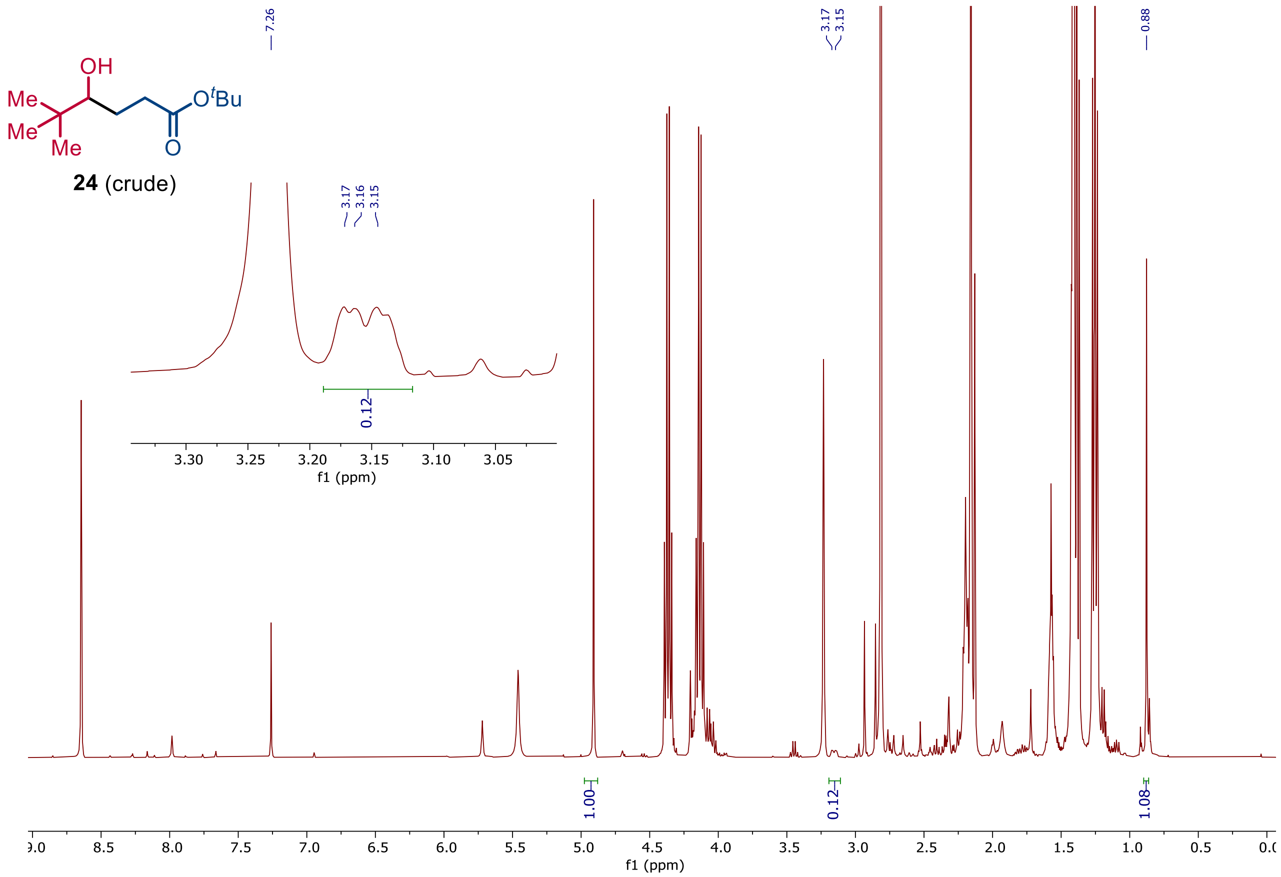

$^{1}$H NMR (400 MHz, $CDCl_3$) of **25** (*see procedure*)

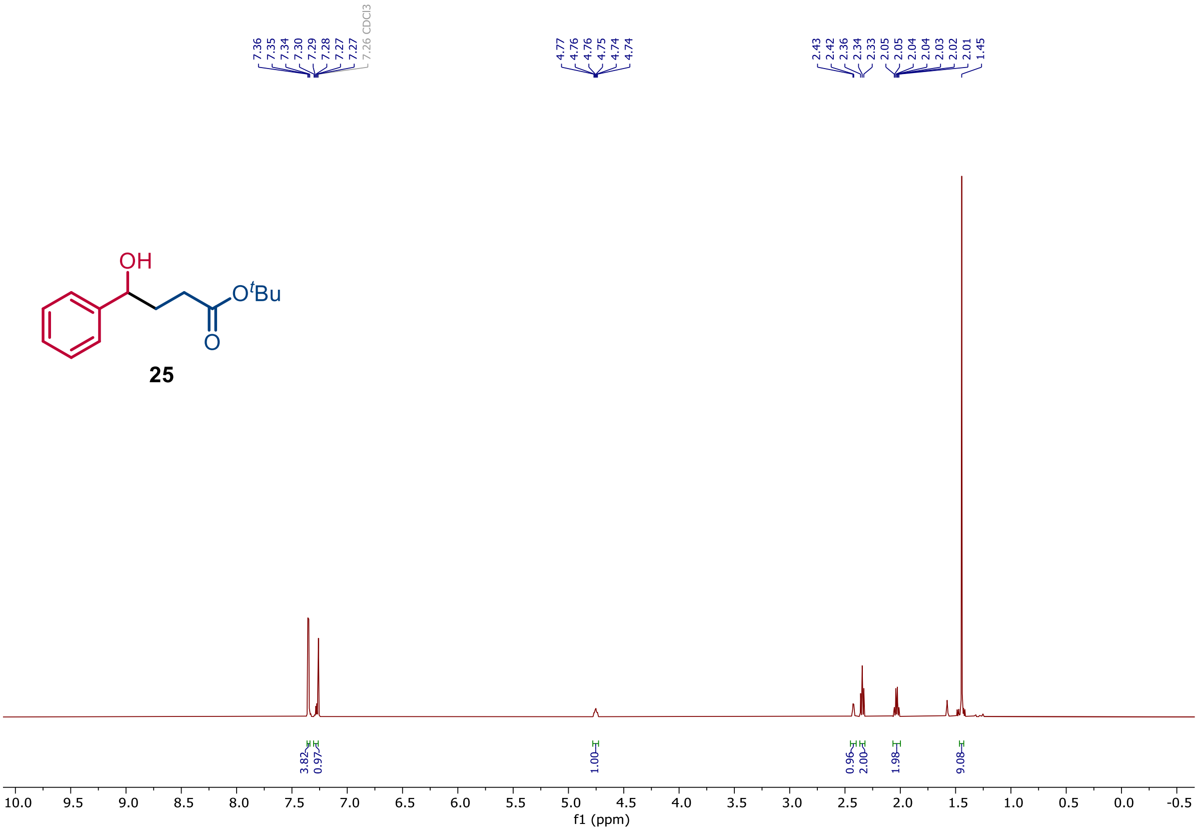


$^{13}$C NMR (101 MHz, $CDCl_3$) of **25**

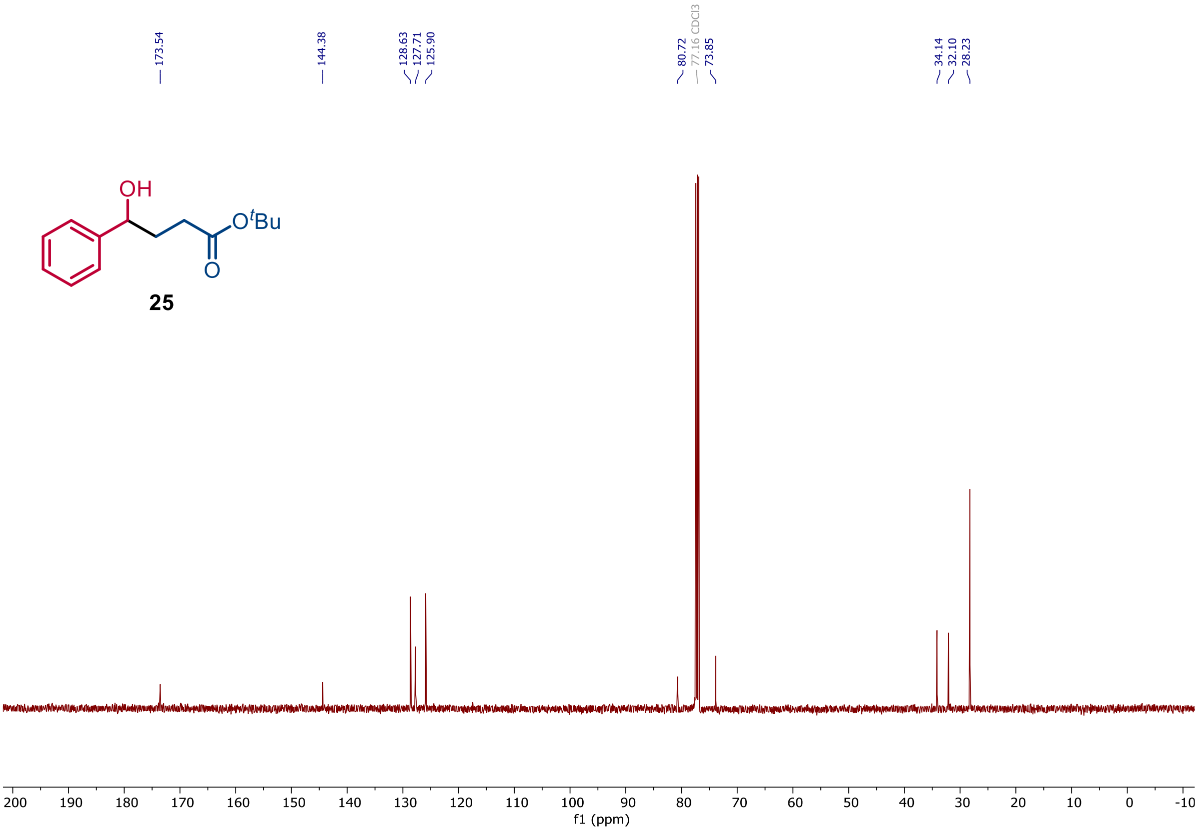

$^{1}$H NMR (400 MHz, $CDCl_3$) of **26** (*see procedure*)

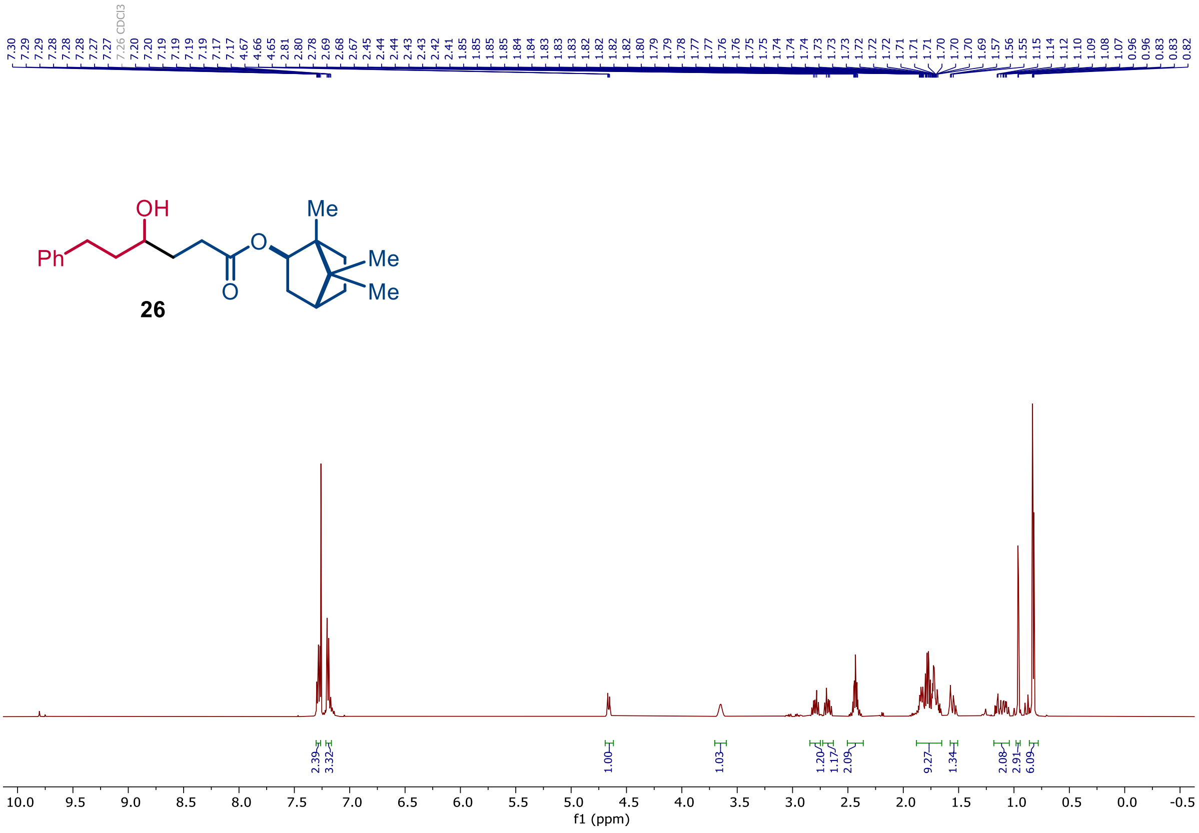


$^{13}$C NMR (101 MHz, $CDCl_3$) of **26**

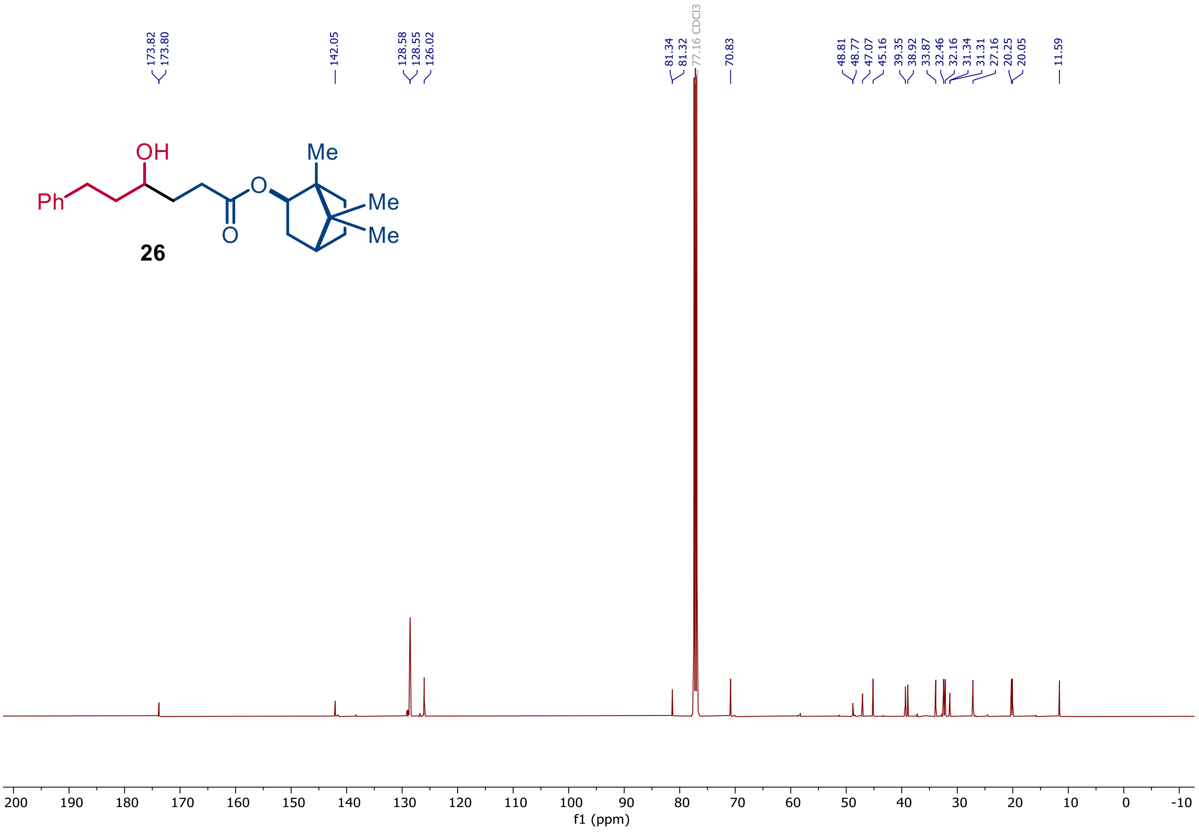

$^{1}$H NMR (400 MHz, $CDCl_3$) of **27** (<u>*see procedure*</u>)

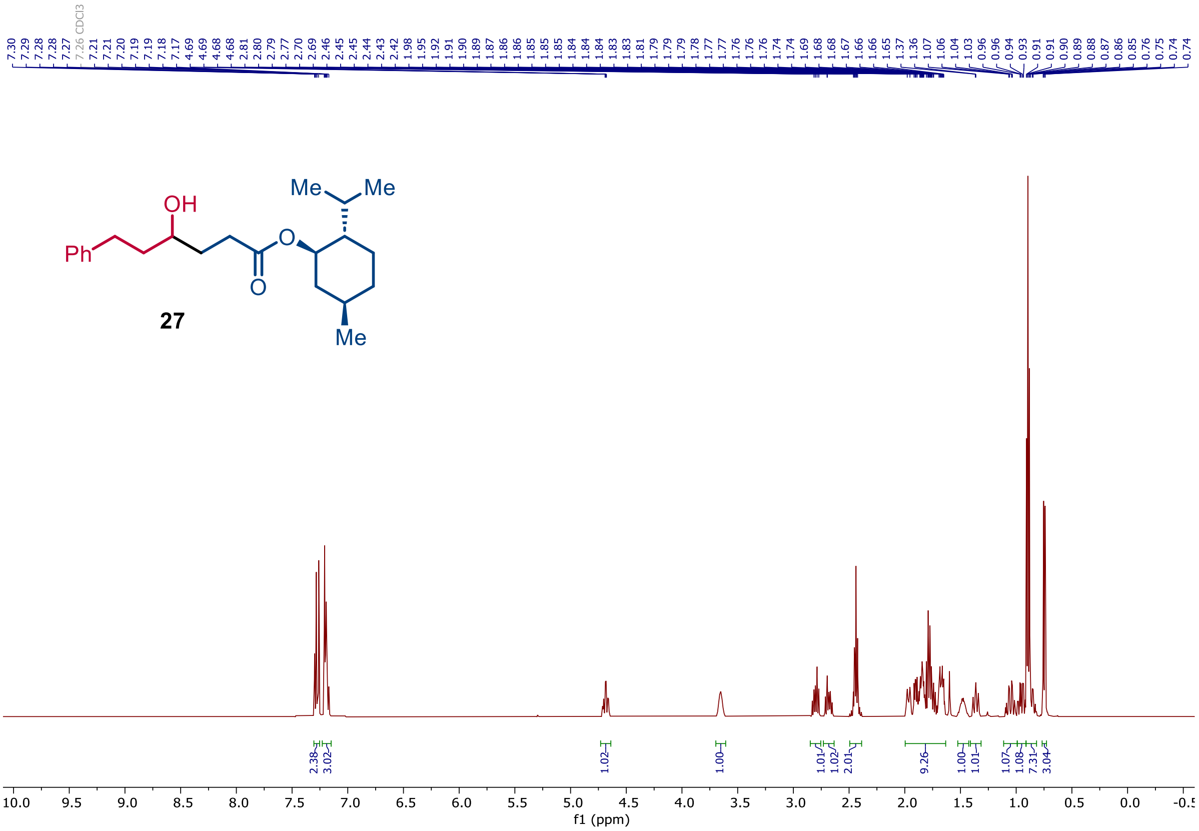


$^{13}$C NMR (101 MHz, $CDCl_3$) of **27**

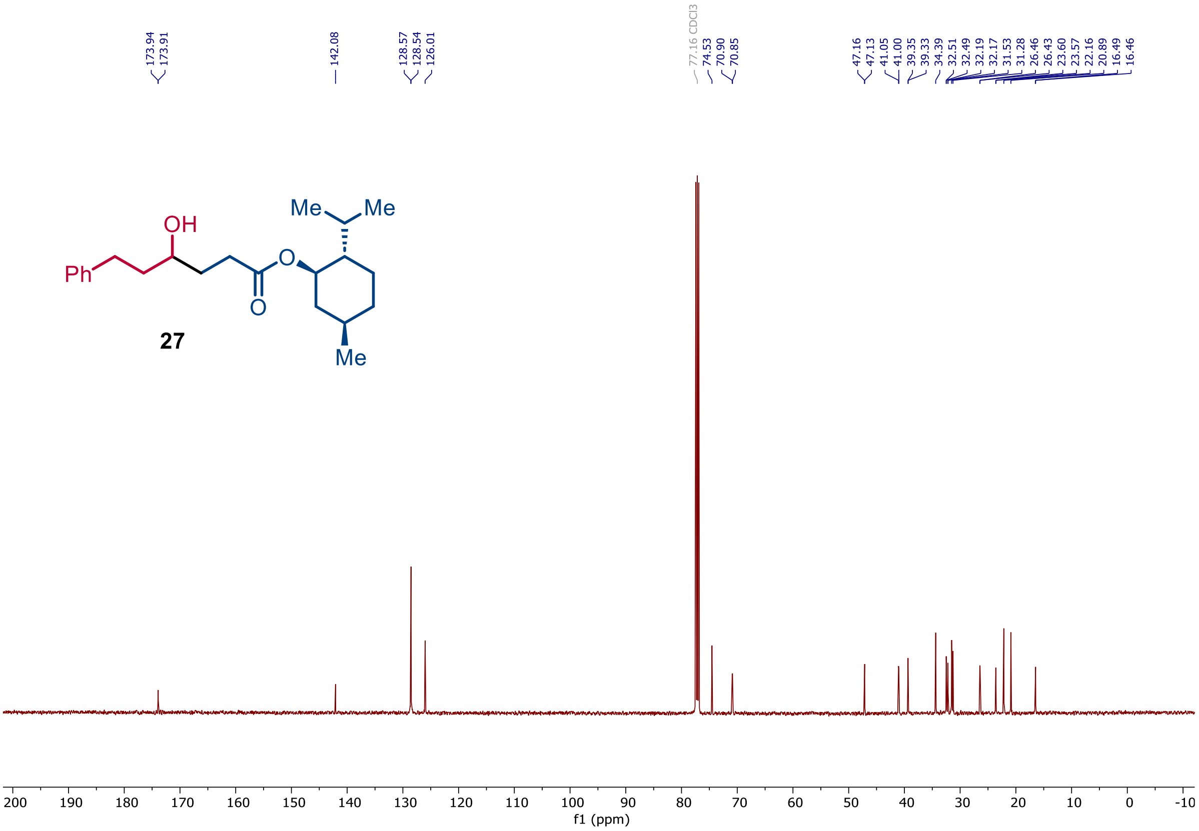

$^{1}$H NMR (400 MHz, $CDCl_3$) of **28** (*see procedure*)

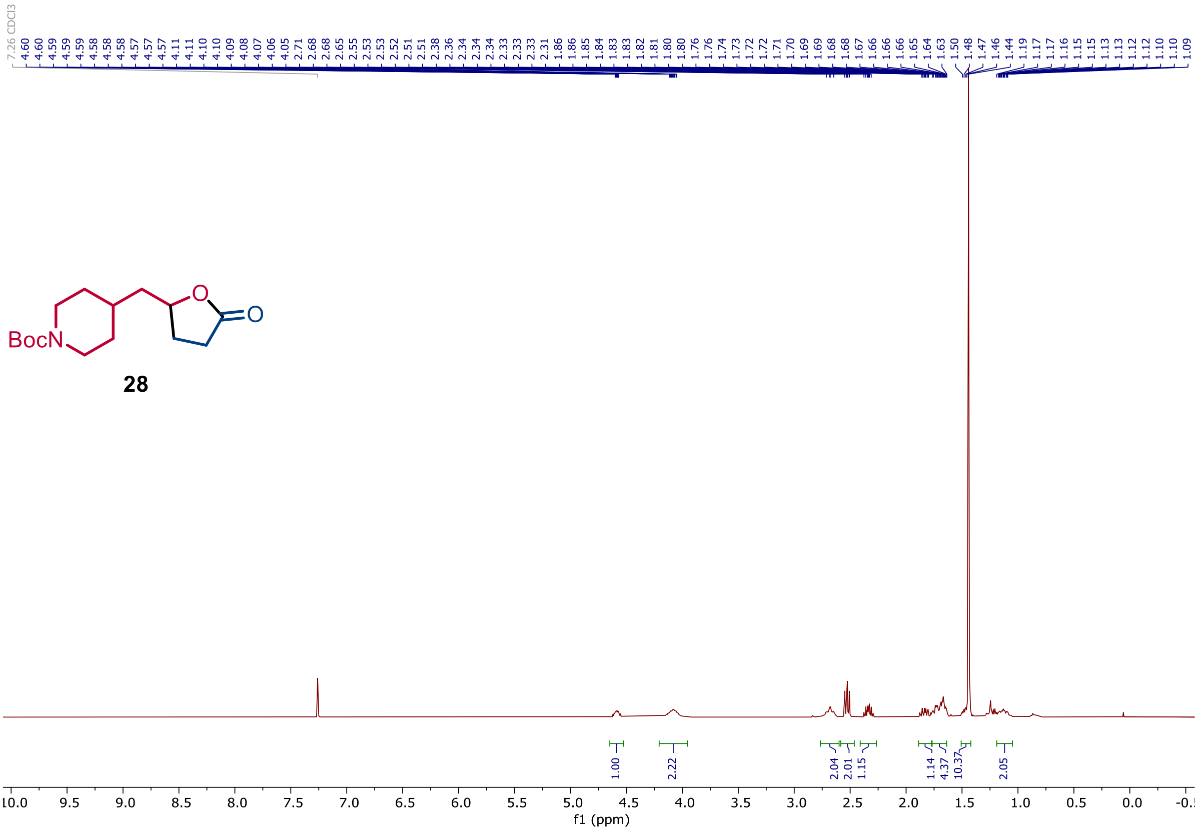


$^{13}$C NMR (101 MHz, $CDCl_3$) of **28**

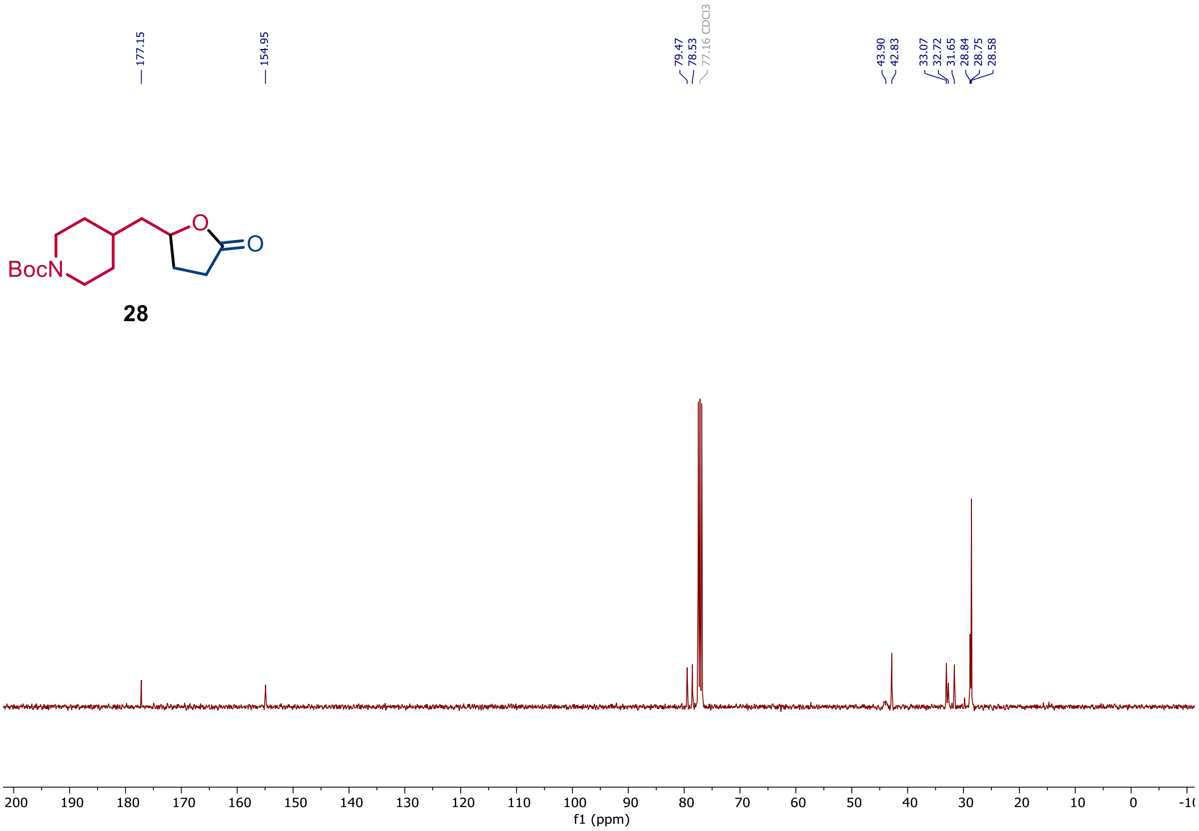

$^{1}$H NMR (400 MHz, $CDCl_3$) of **29** (*see procedure*)

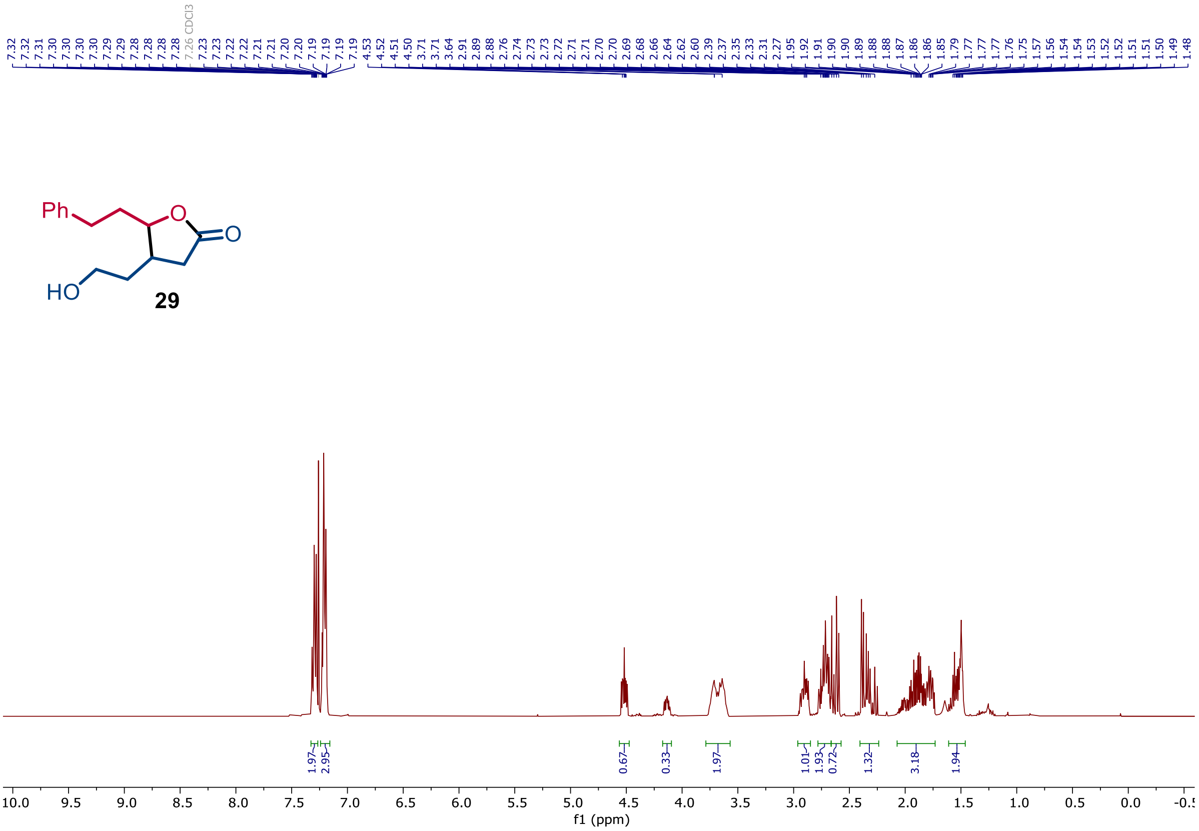


$^{13}$C NMR (101 MHz, $CDCl_3$) of **29**

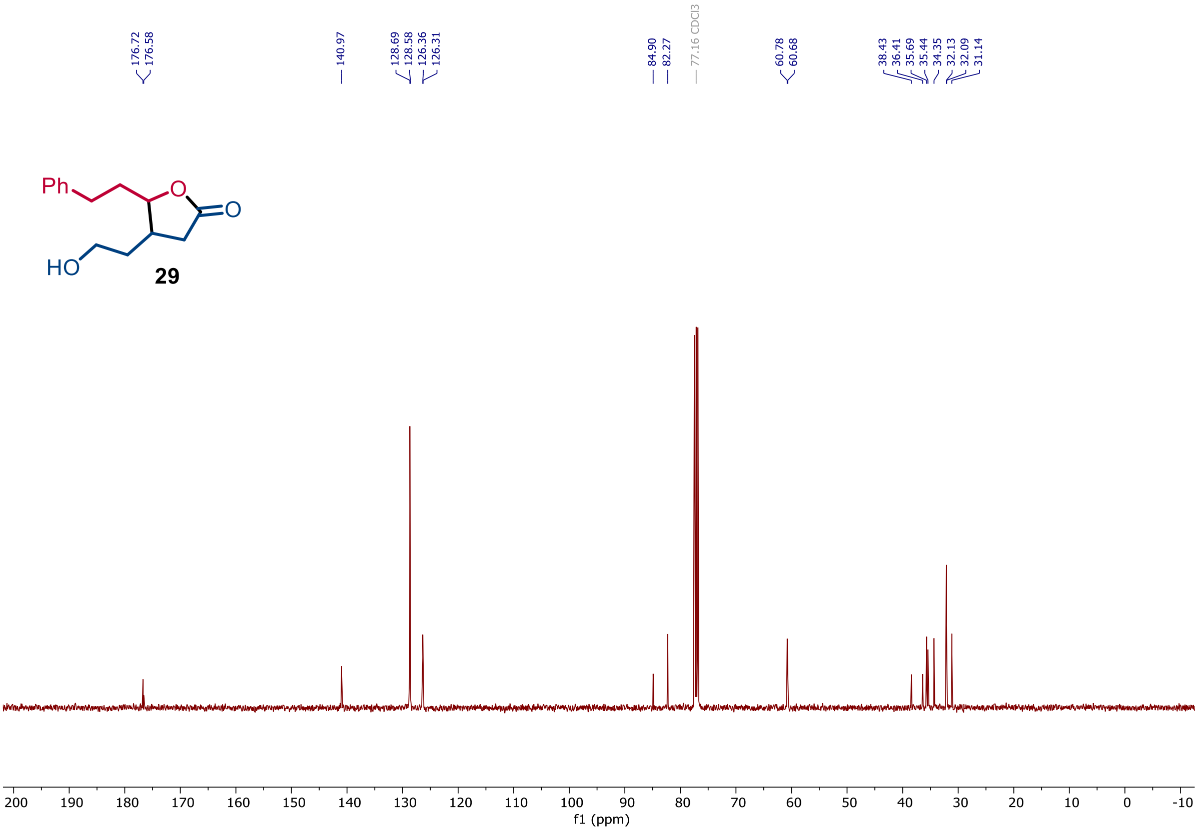

$^{1}H$ NMR (400 MHz, $CDCl_3$) of **30** (*see procedure*)

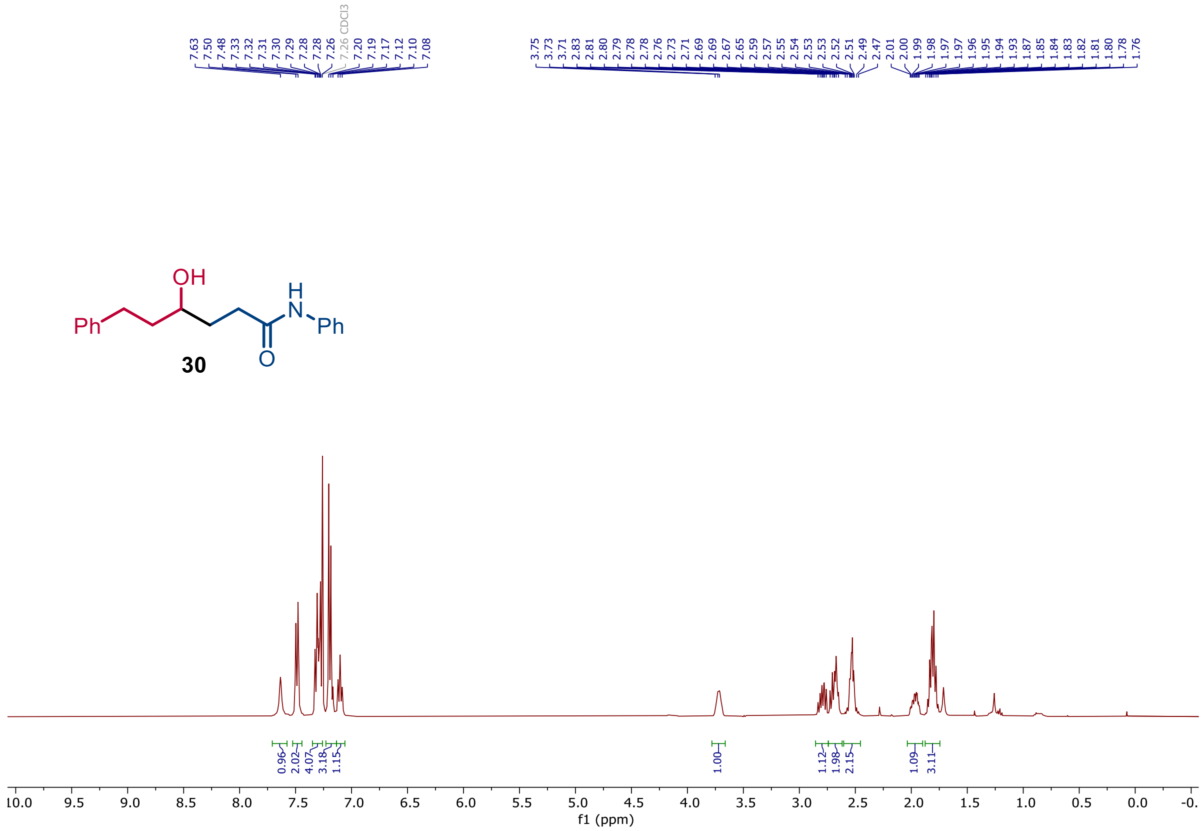


$^{13}C$ NMR (101 MHz, $CDCl_3$) of **30**

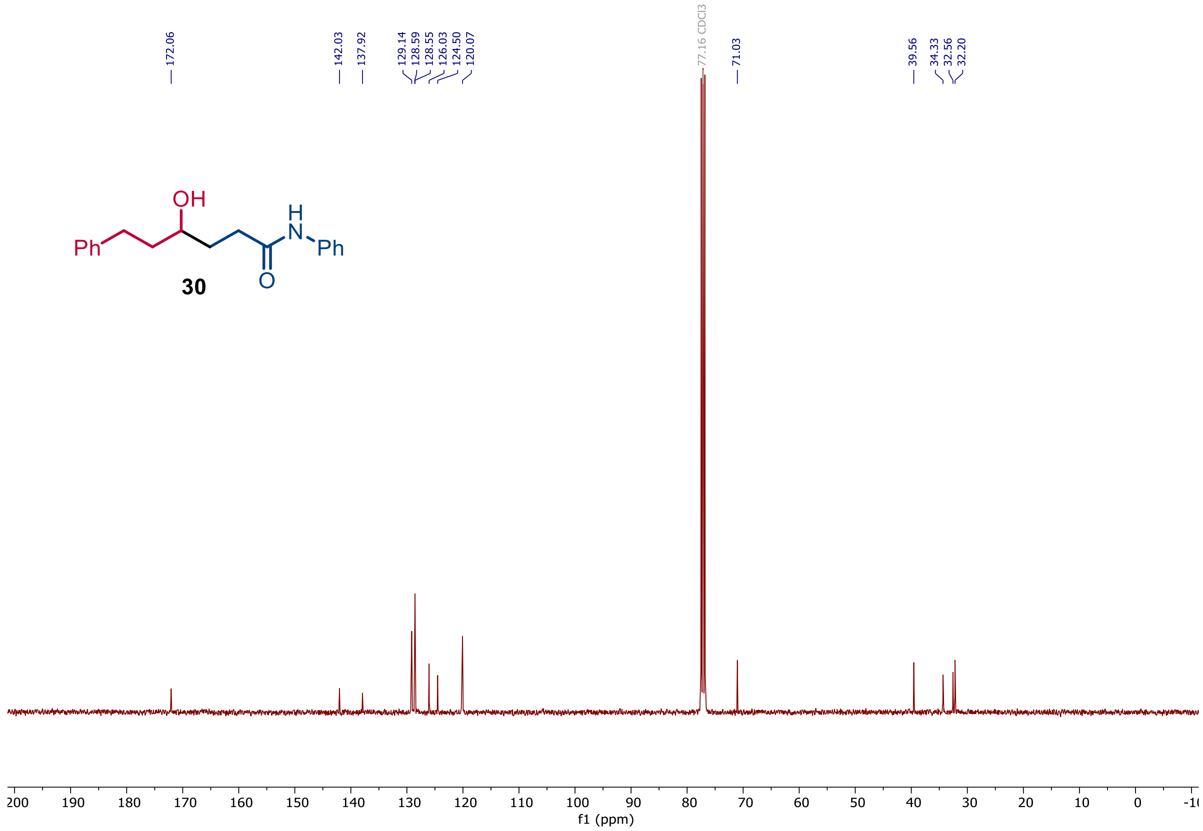

$^1$H NMR (400 MHz, $CDCl_3$) of **31** (*see procedure*)

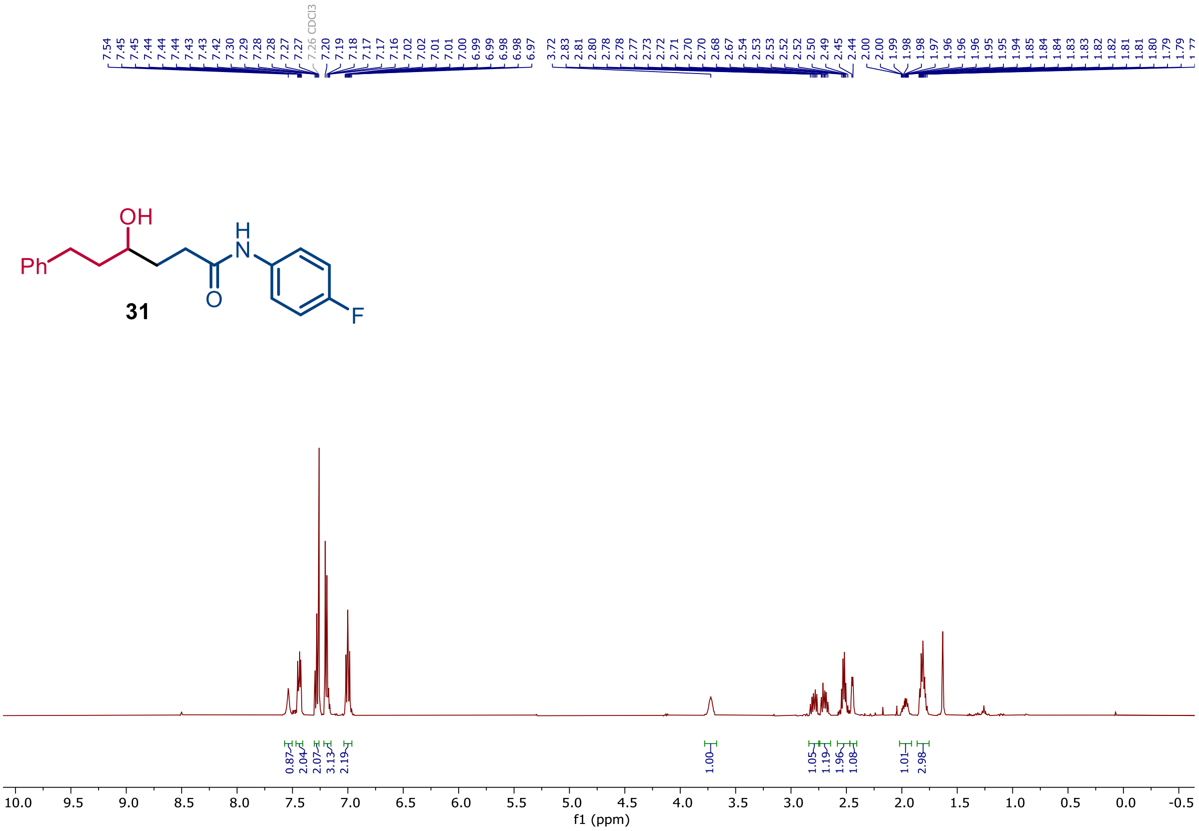

$^{13}$C NMR (101 MHz, $CDCl_3$) of **31**

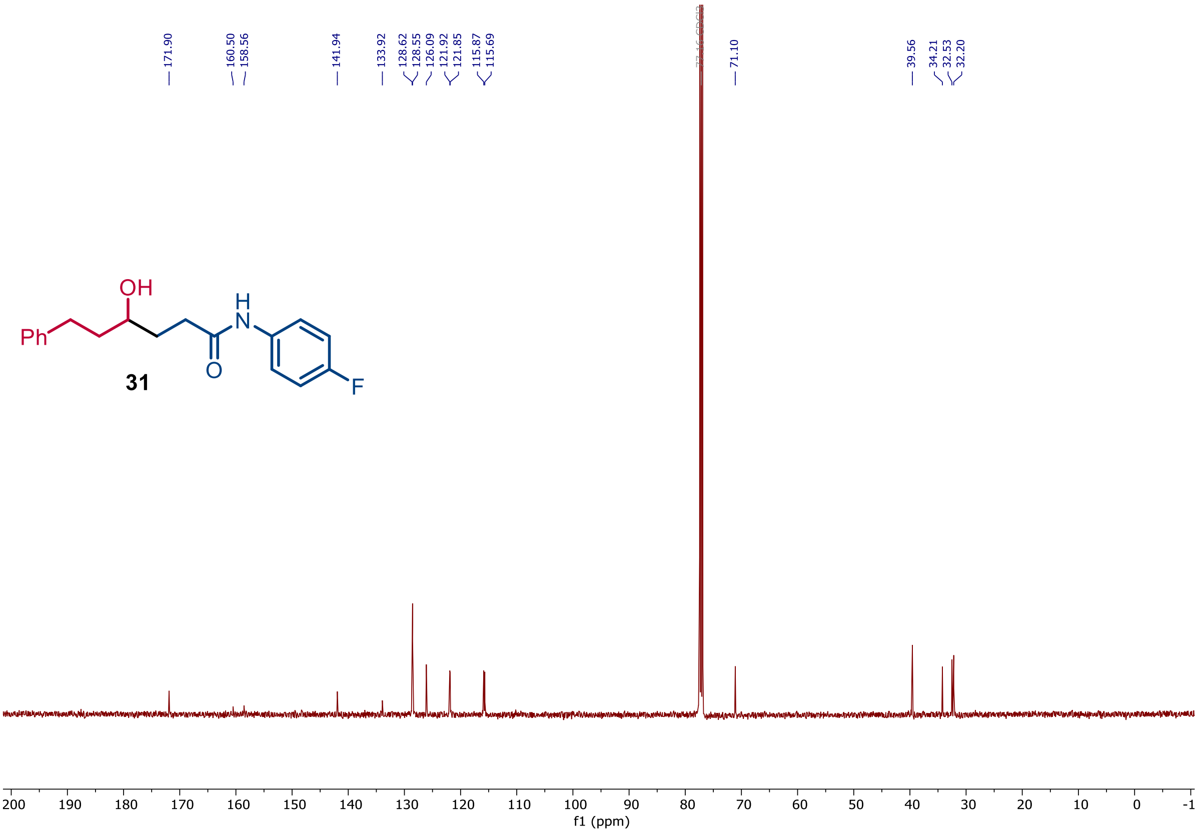

$^{19}$F NMR (377 MHz, $CDCl_3$) of **F-acrylamide**

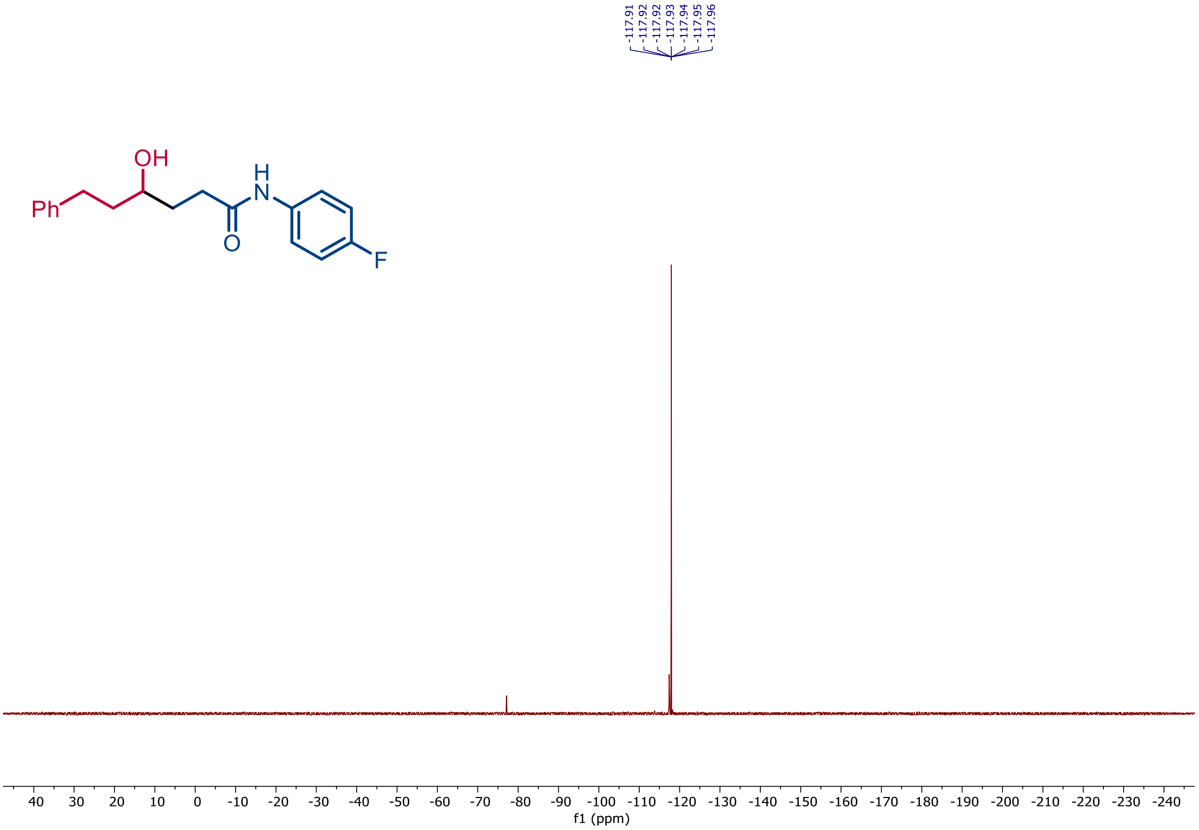

$^{1}$H NMR (400 MHz, $CD_3CN$) of **32** (*see procedure*)

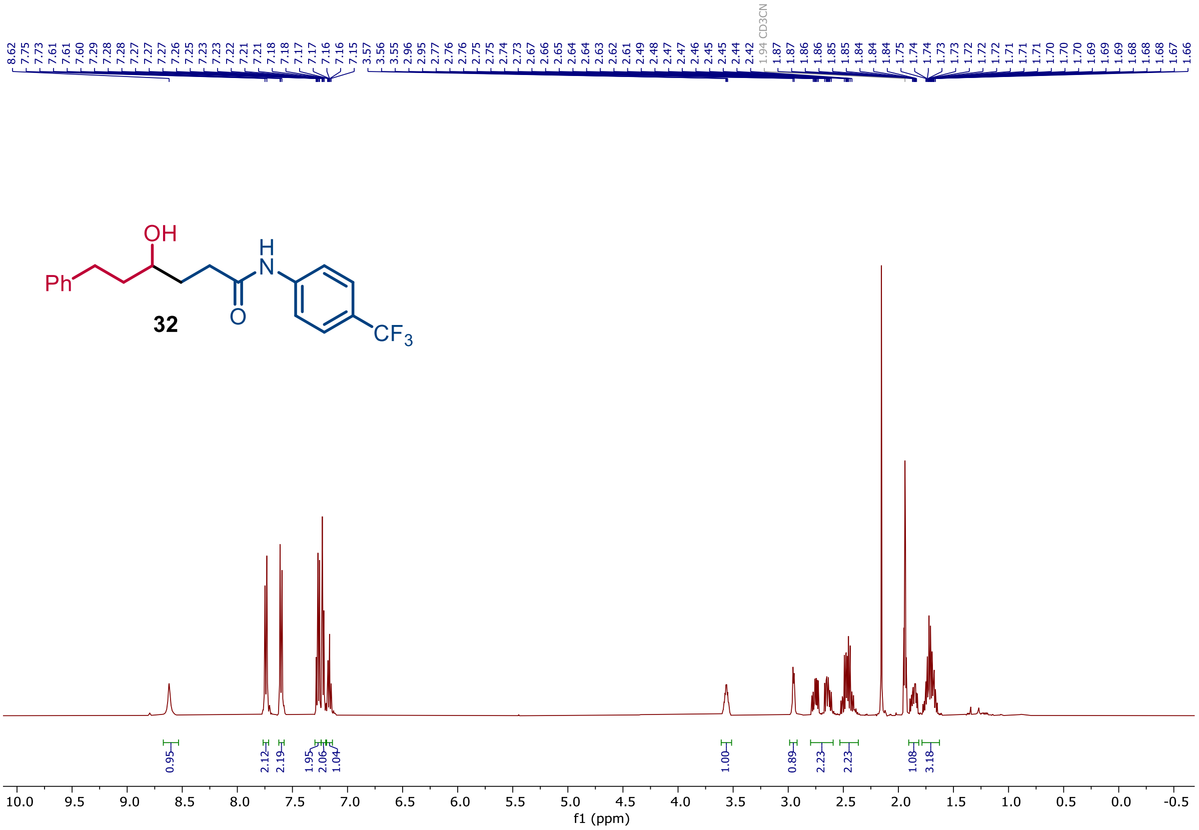


$^{13}$C NMR (101 MHz, $CD_3CN$) of **32**

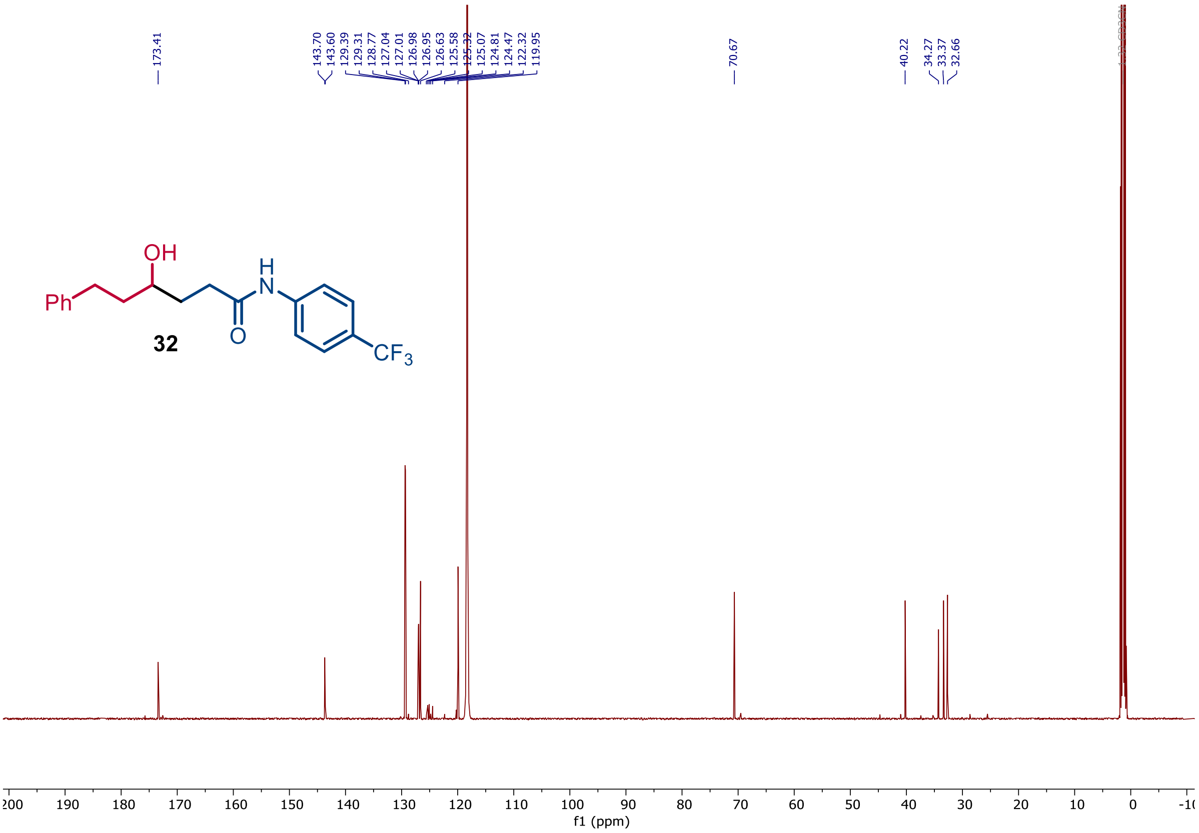

$^{19}F$ NMR (377 MHz, $CD_3CN$) of **CF3-acrylamide**

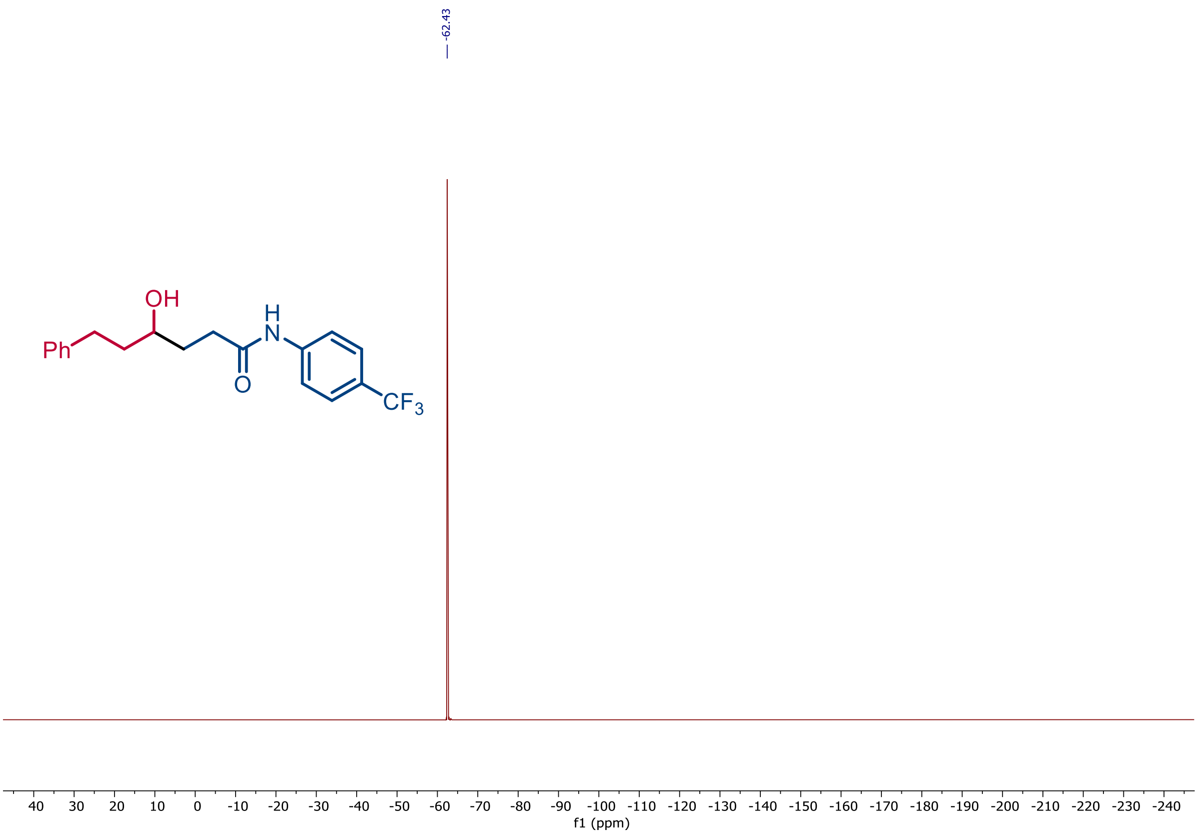

$^{1}$H NMR (400 MHz, $CDCl_3$) of **33** (*see procedure*)

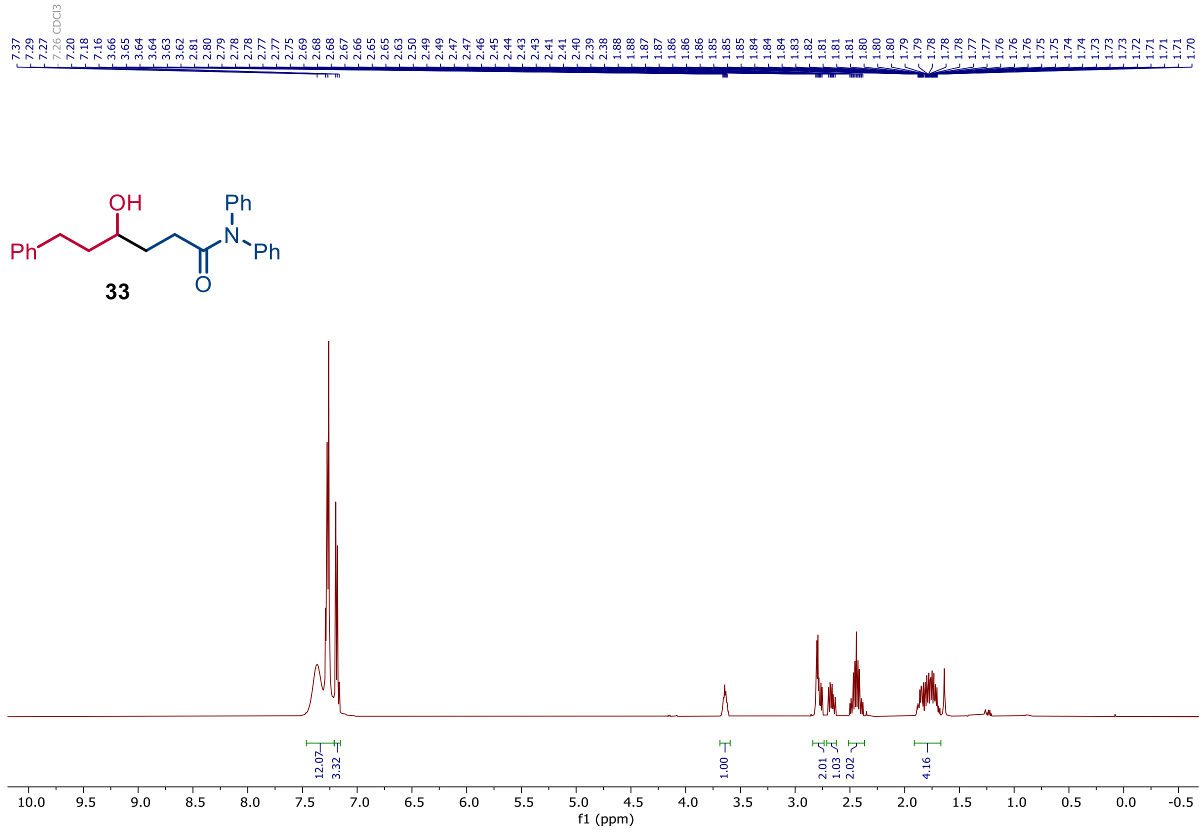


$^{13}$C NMR (101 MHz, $CDCl_3$) of **33**

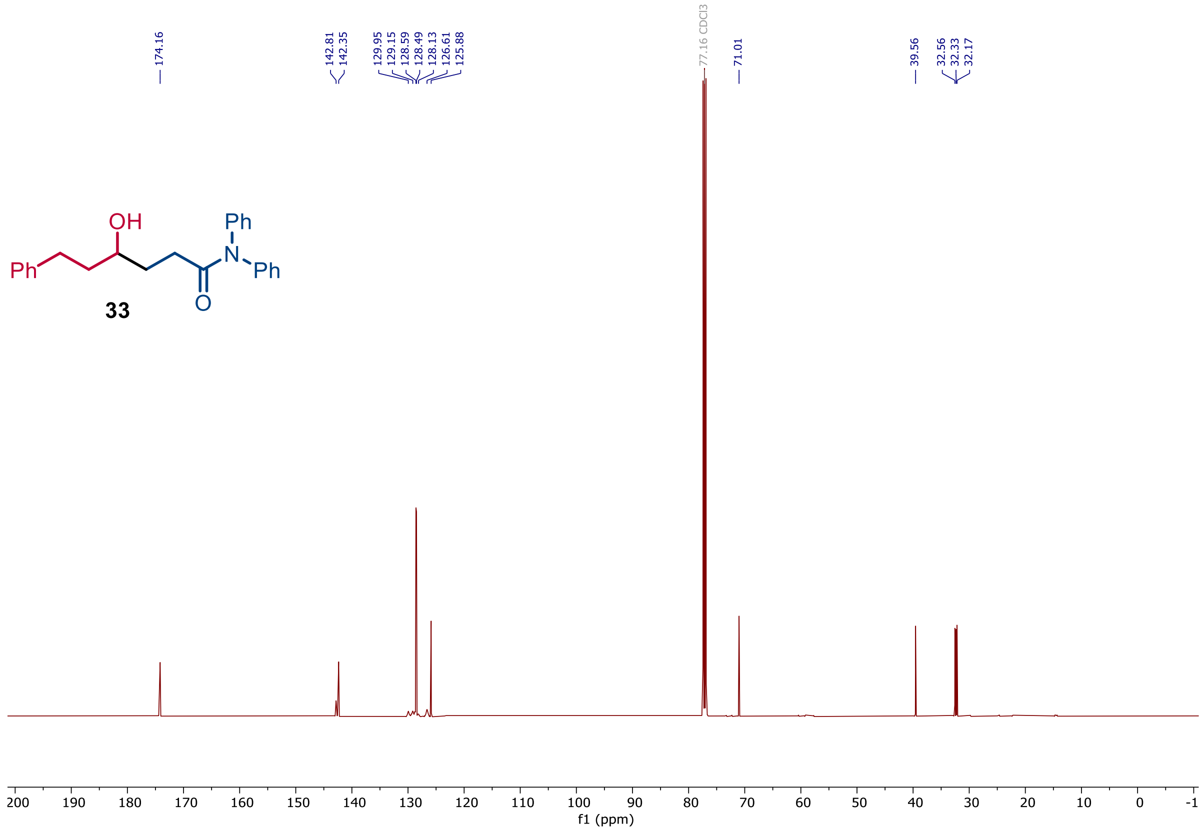

$^{1}$H NMR (400 MHz, CDCl$_3$) of **34** (*see procedure*)

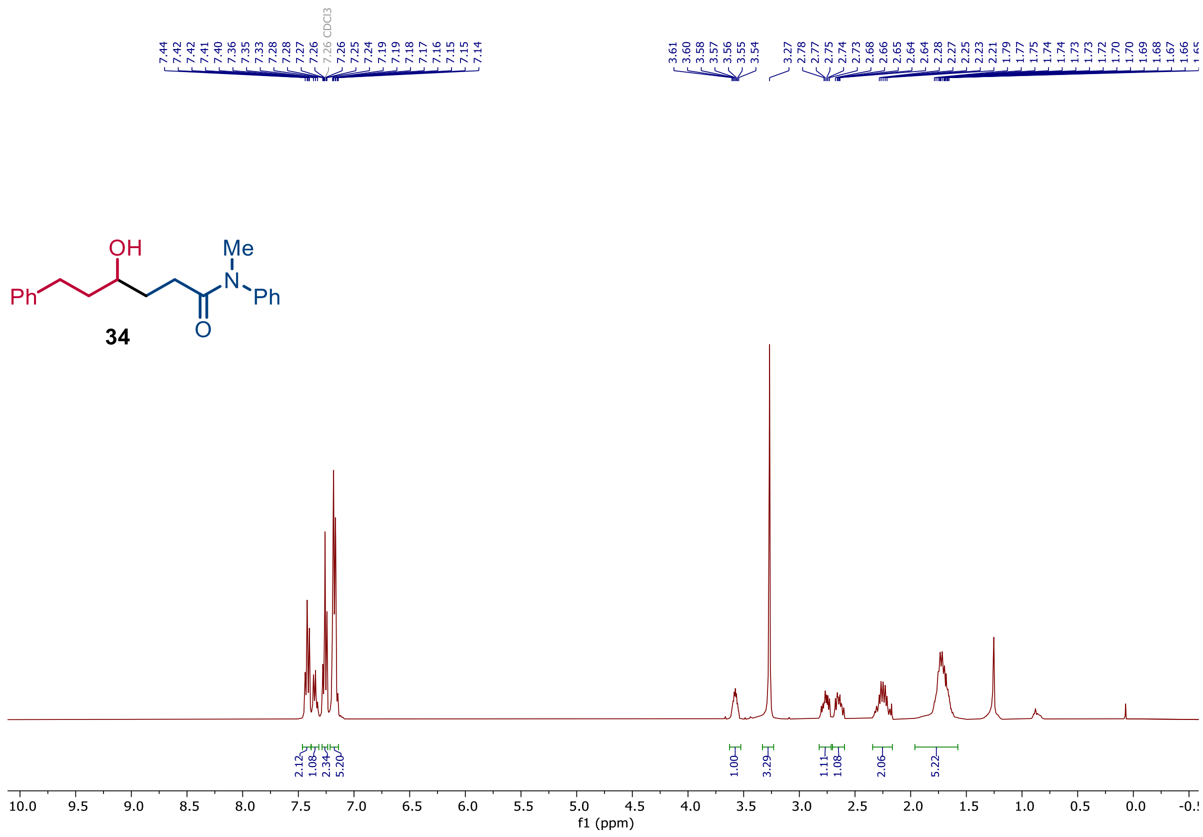


$^{13}$C NMR (101 MHz, CDCl$_3$) of **34**

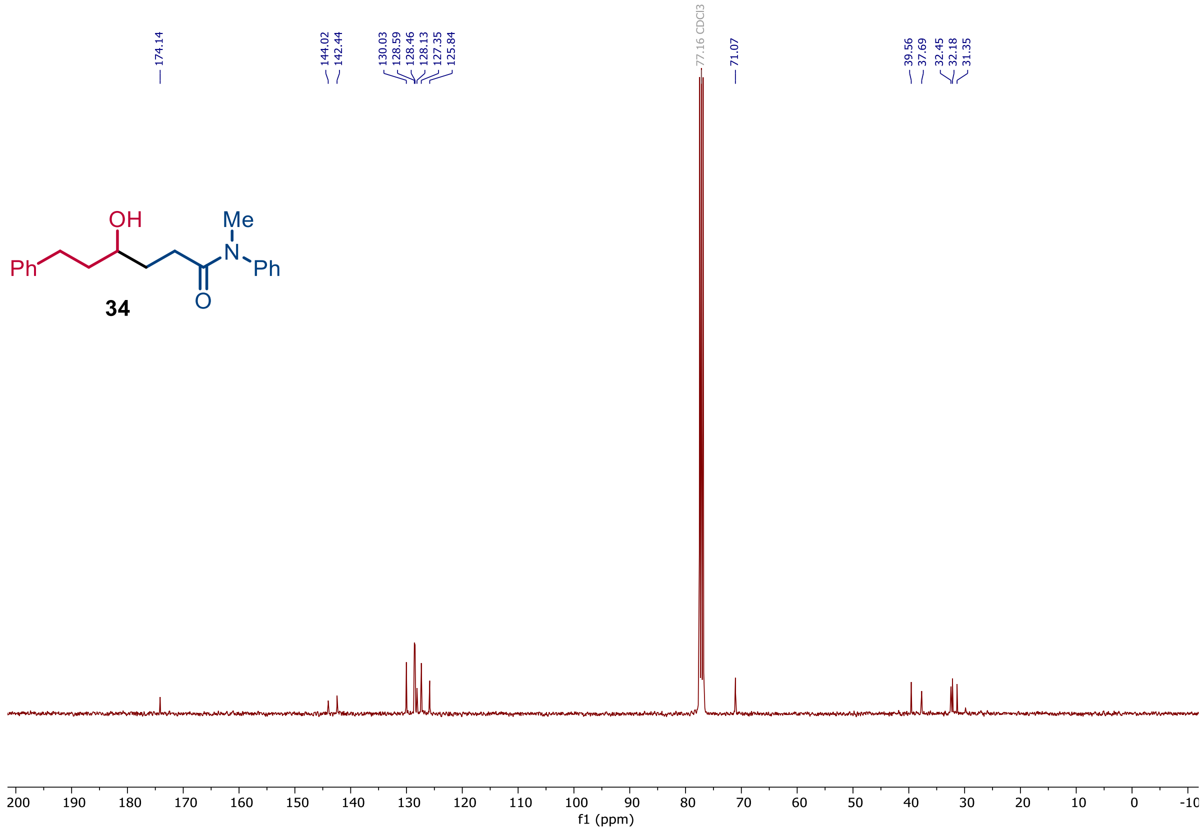

$^{1}$H NMR (400 MHz, $CDCl_3$) of **36** (*see procedure*)

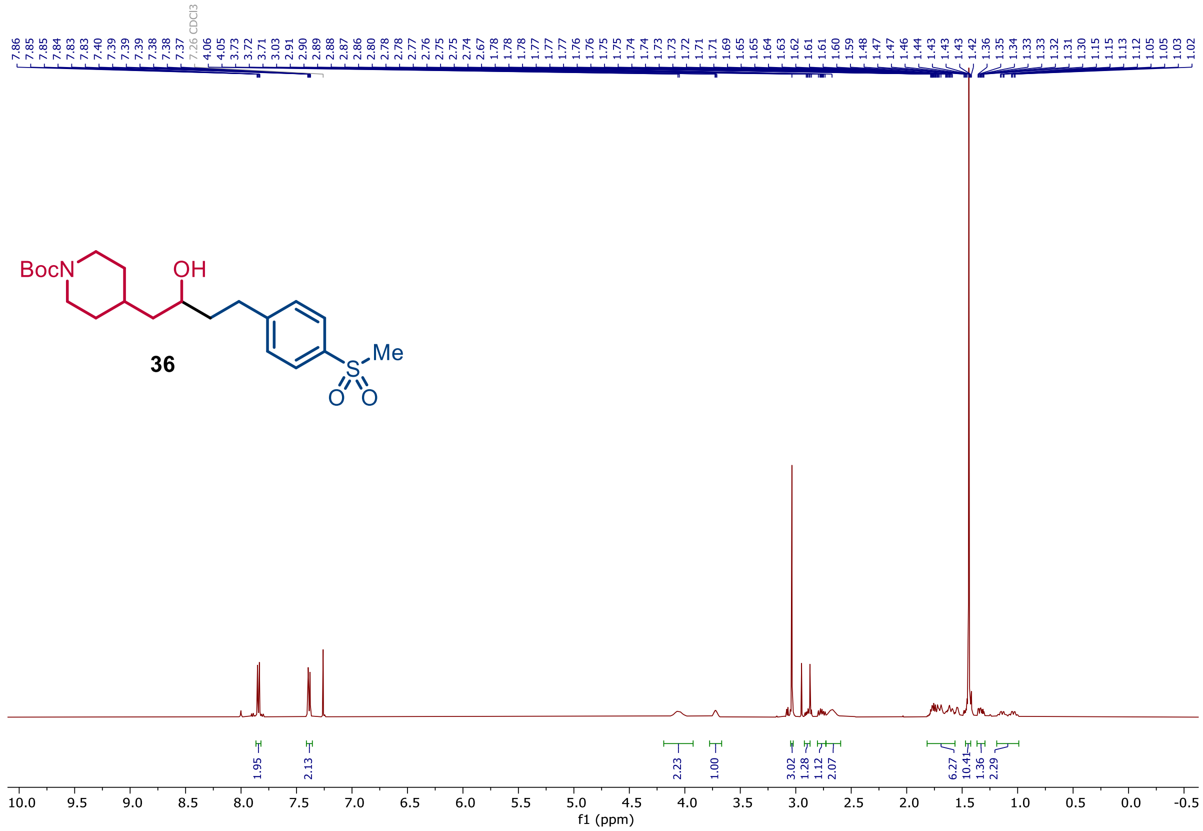


$^{13}$C NMR (101 MHz, $CDCl_3$) of **36**

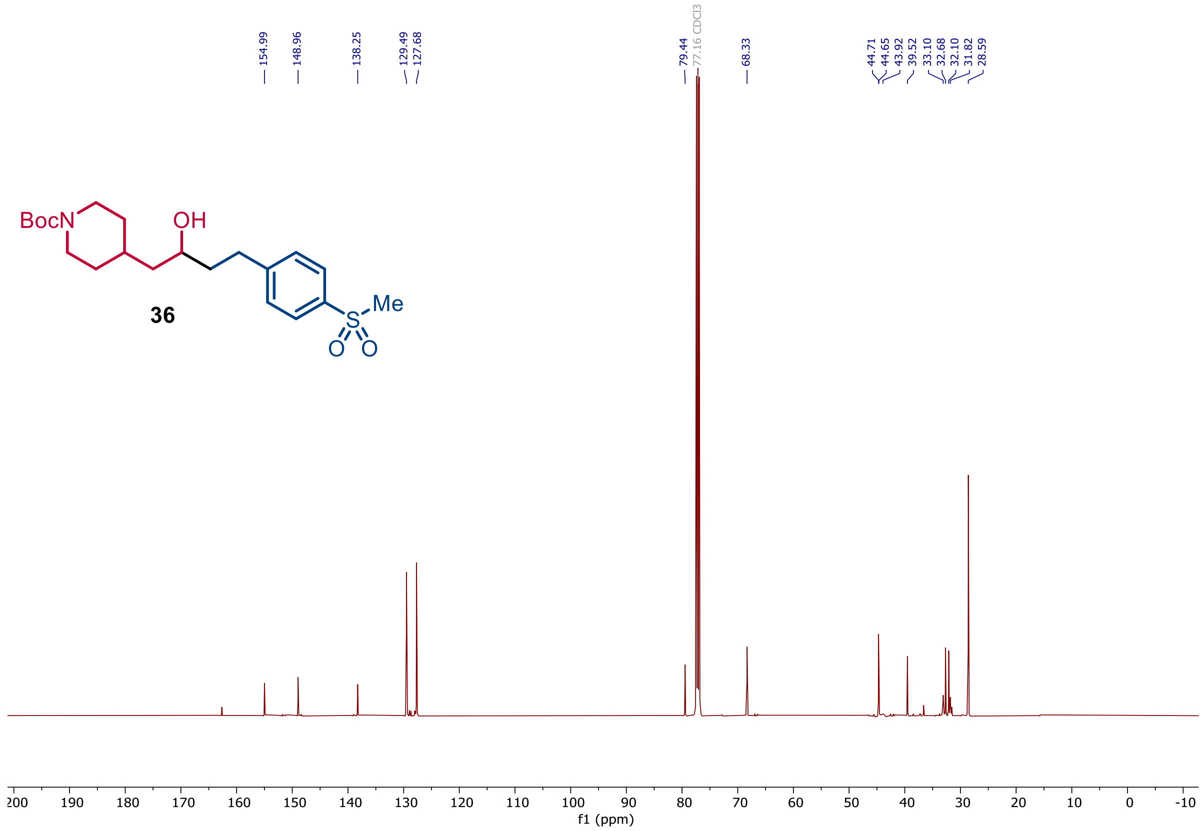

$^{1}$H NMR (400 MHz, $CDCl_3$) of **37** (*see procedure*)

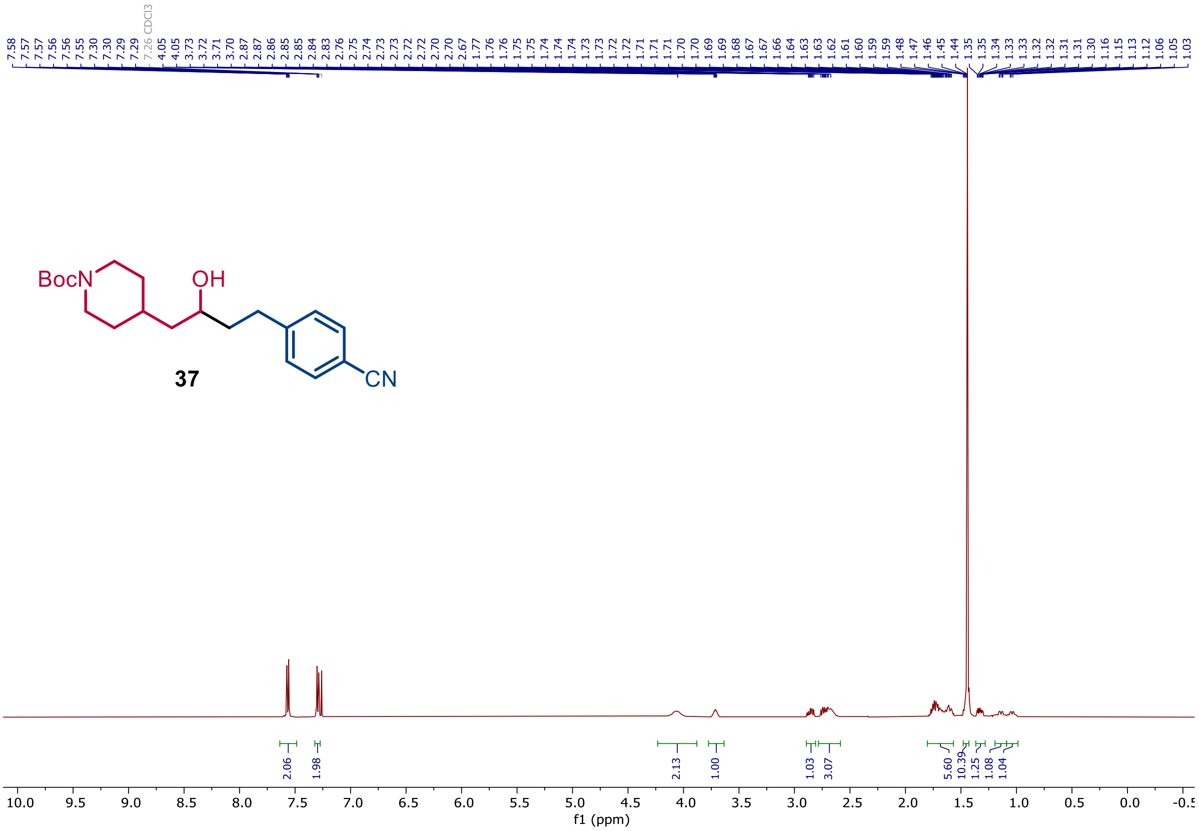


$^{13}$C NMR (101 MHz, $CDCl_3$) of **37**

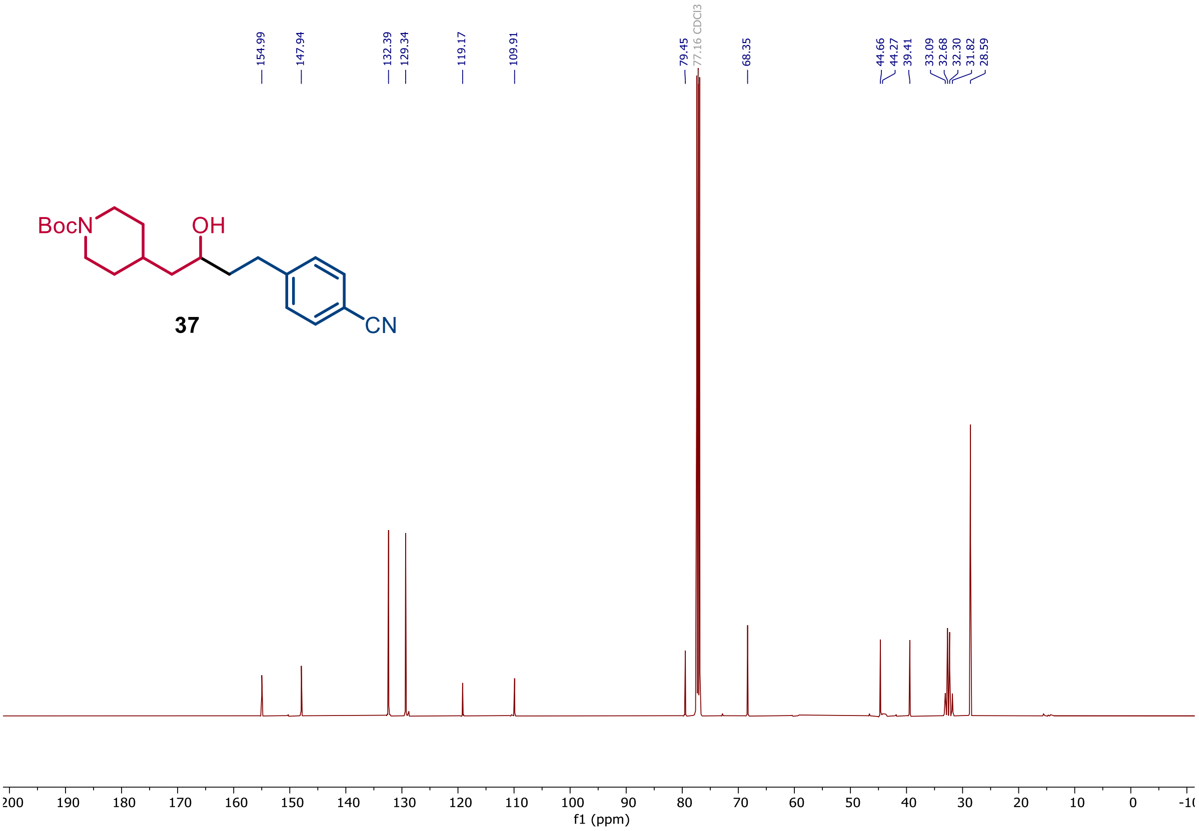

$^{1}$H NMR (400 MHz, $CDCl_3$) of **38** (*see procedure*)

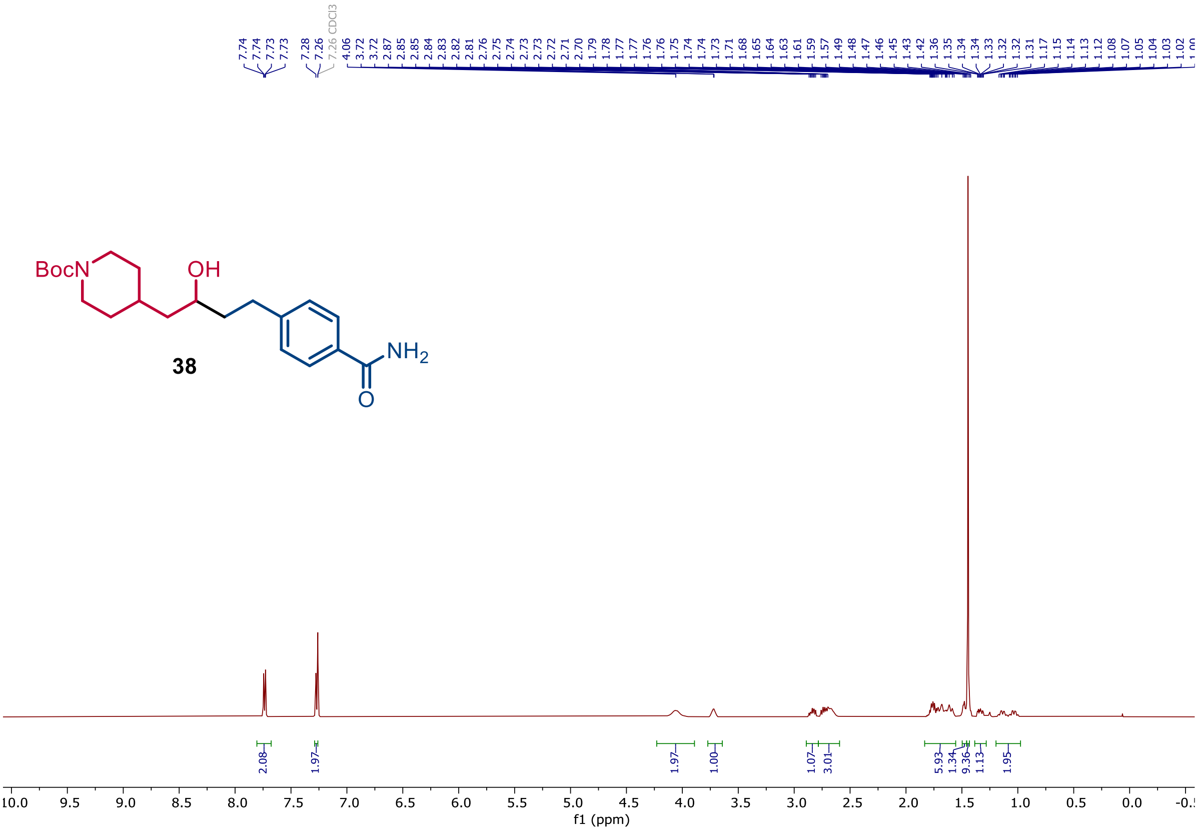


$^{13}$C NMR (101 MHz, $CDCl_3$) of **38**

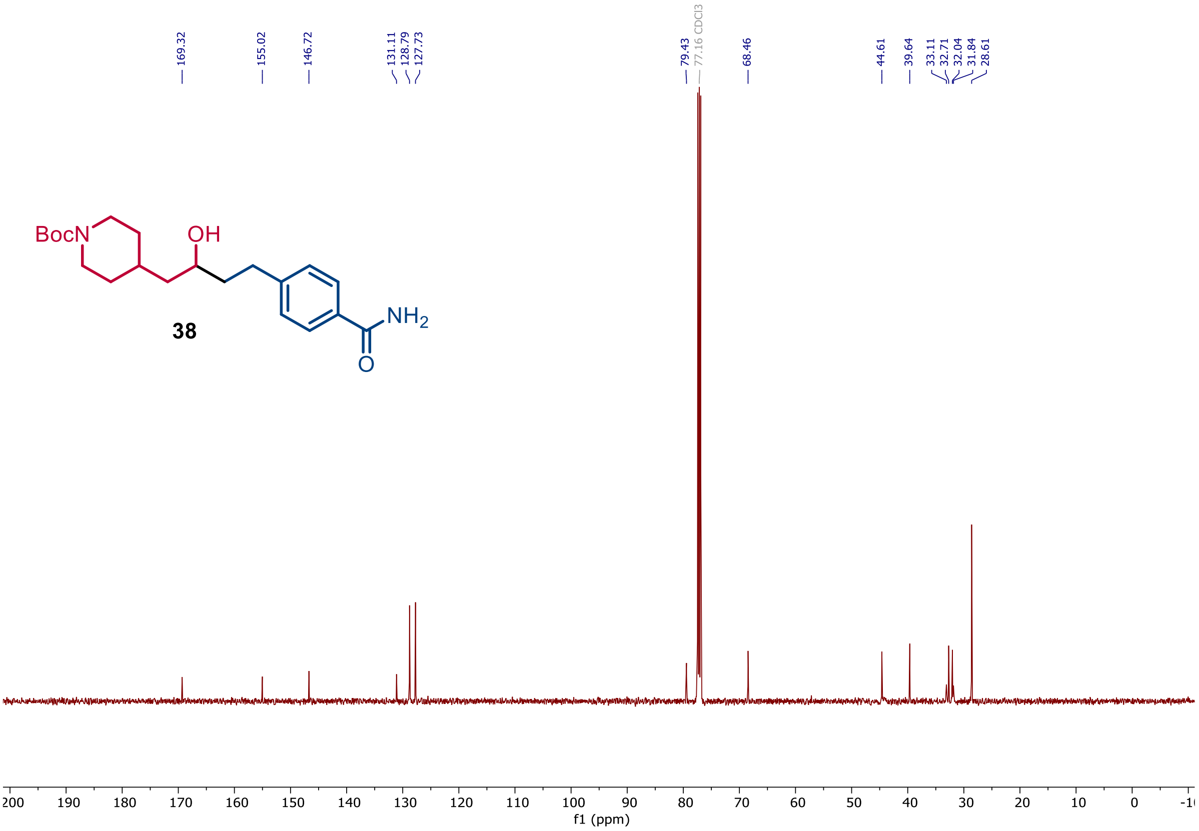

$^{1}$H NMR (400 MHz, $CDCl_3$) of **39** (*see procedure*)

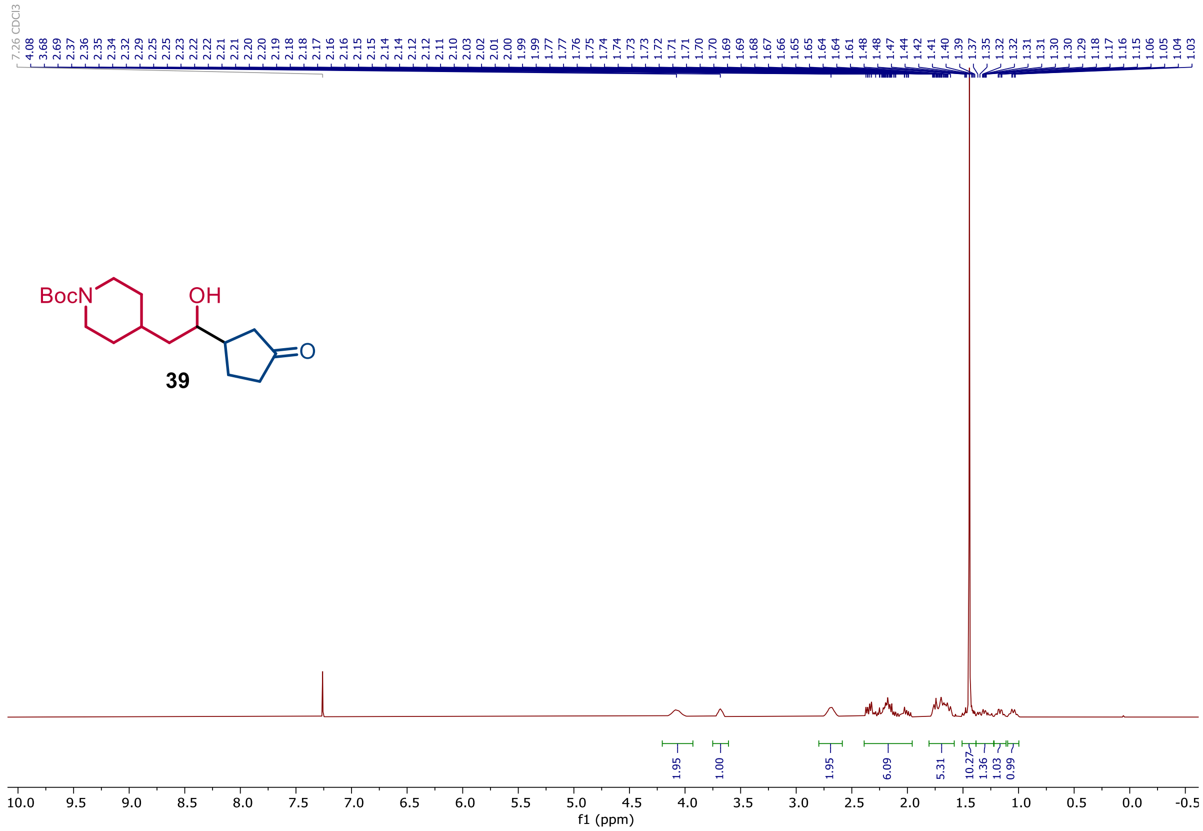


$^{13}$C NMR (101 MHz, $CDCl_3$) of **39**

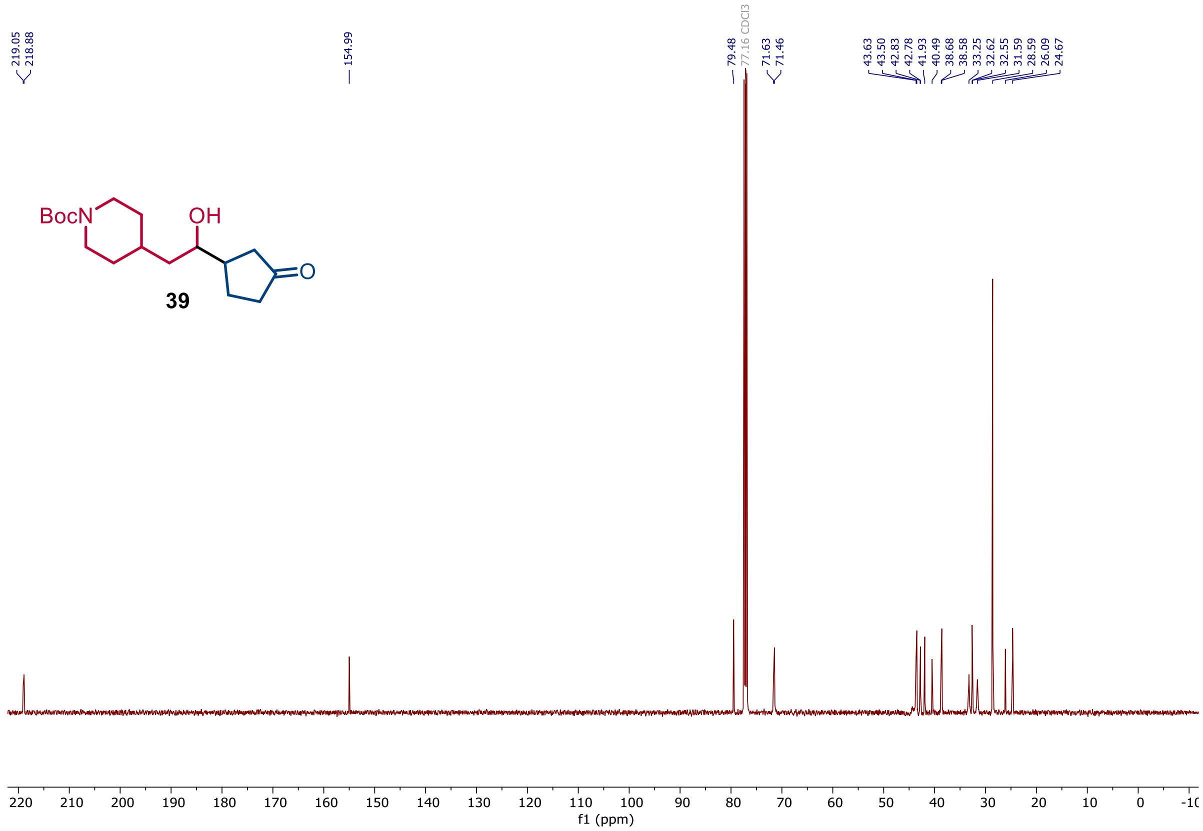

$^{1}$H NMR (400 MHz, $CDCl_3$) of **40** (*see procedure*)

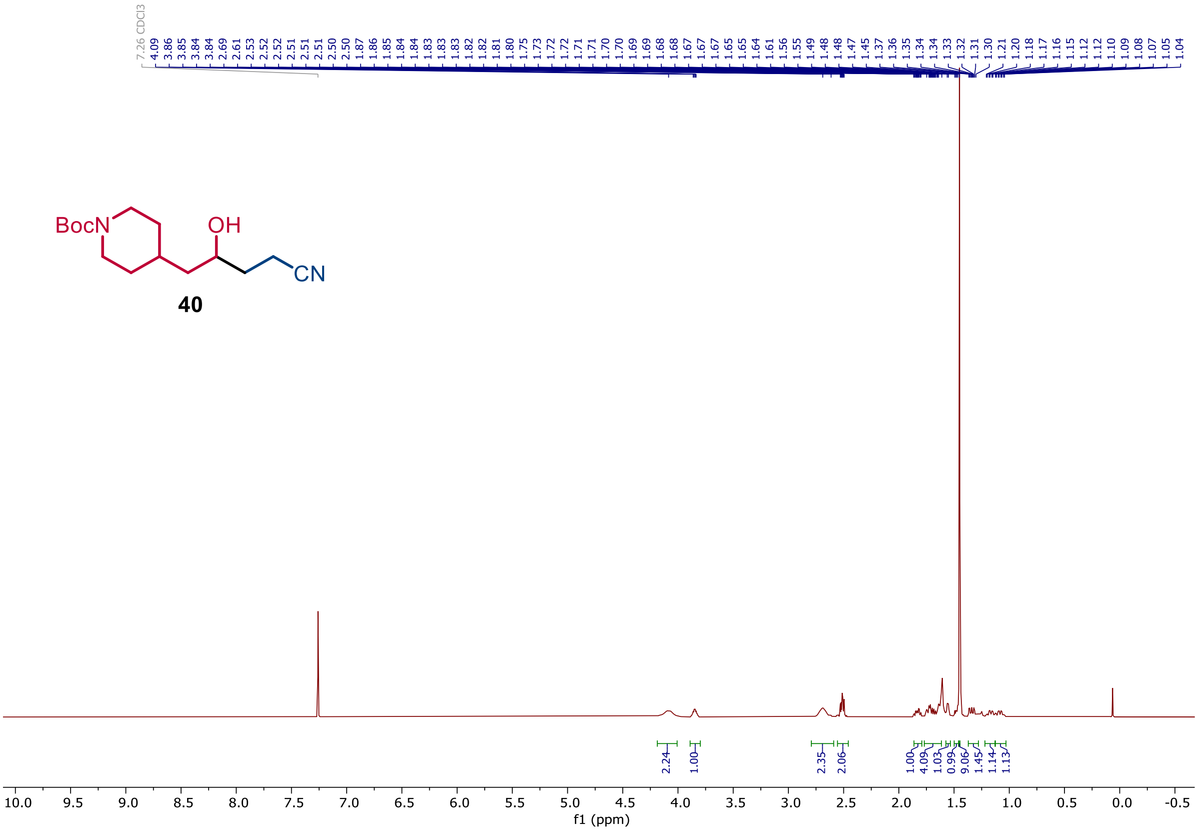

$^{13}C$ NMR (101 MHz, $CDCl_3$) of **40**

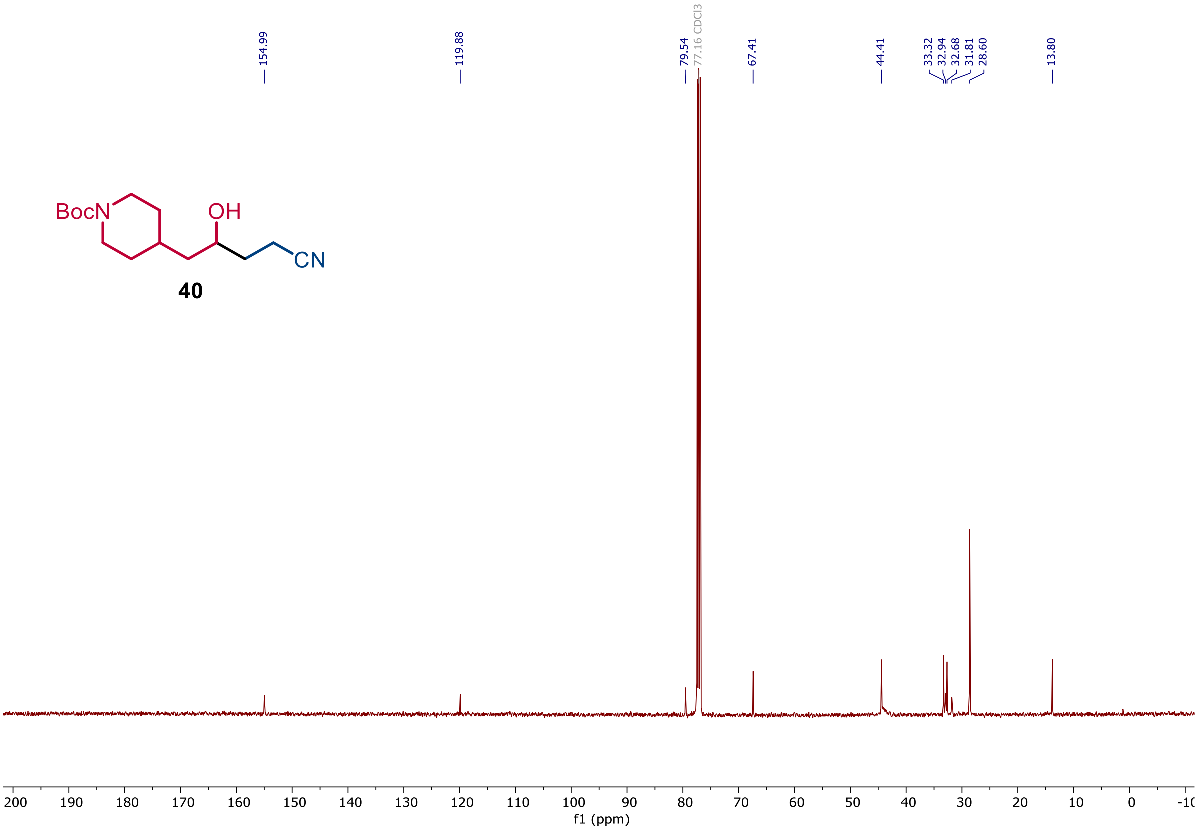

$^{1}$H NMR (400 MHz, $CDCl_3$) of **5** (*see procedure*)

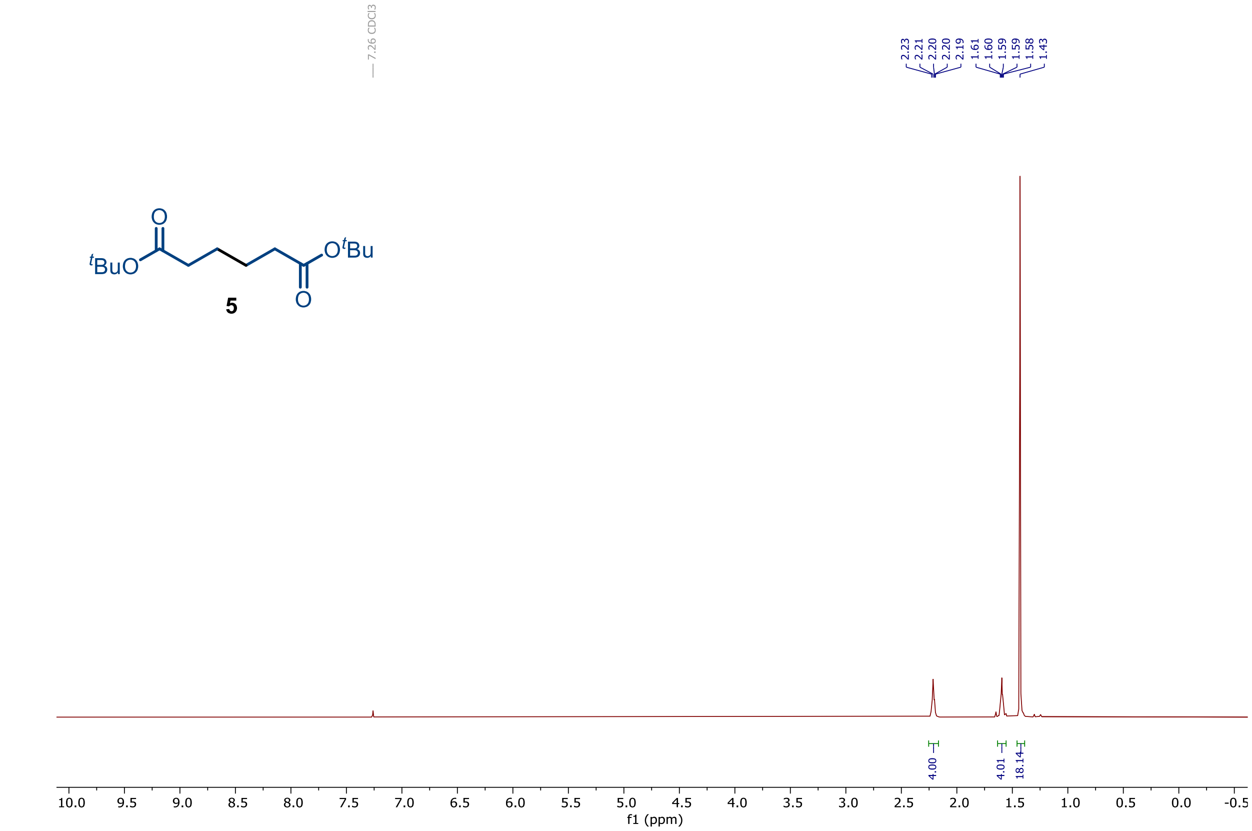


$^{13}$C NMR (400 MHz, $CDCl_3$) of **5**

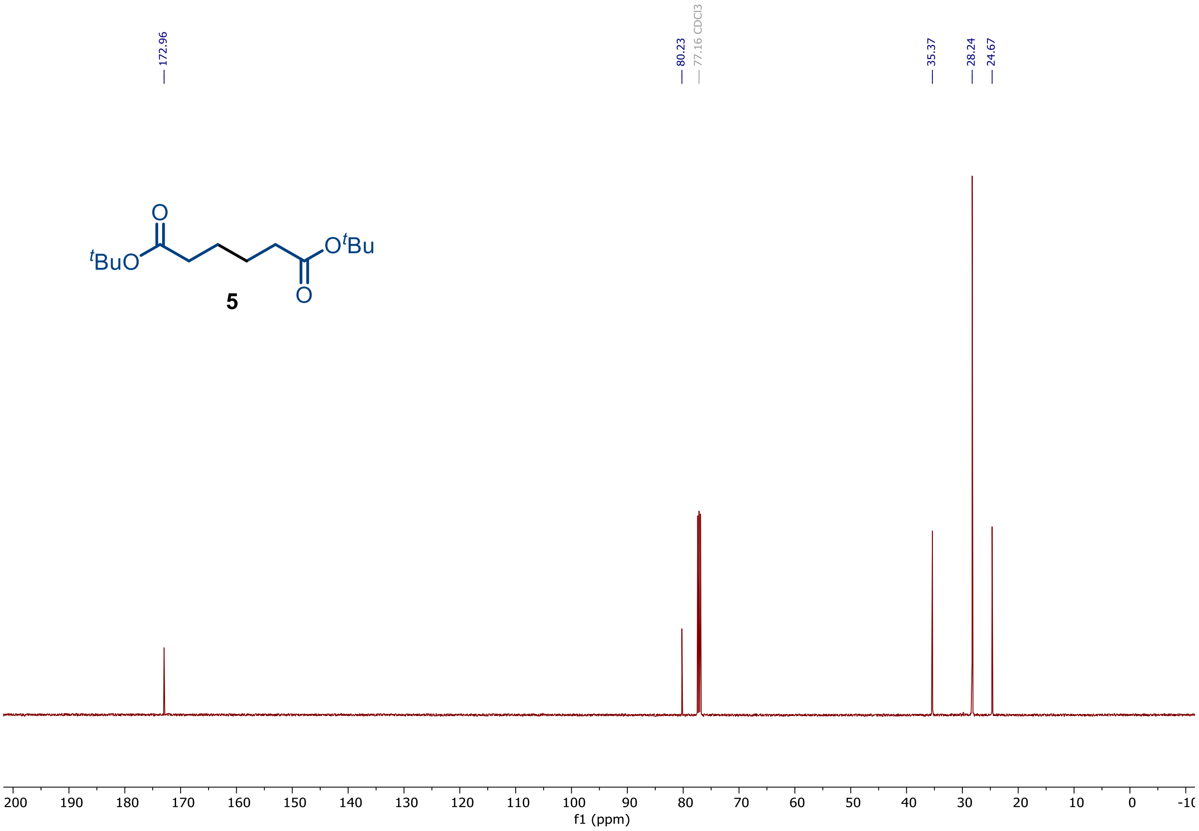

$^1$H NMR (400 MHz, $CDCl_3$) of **42** (diastereomer 1) (*see procedure*)

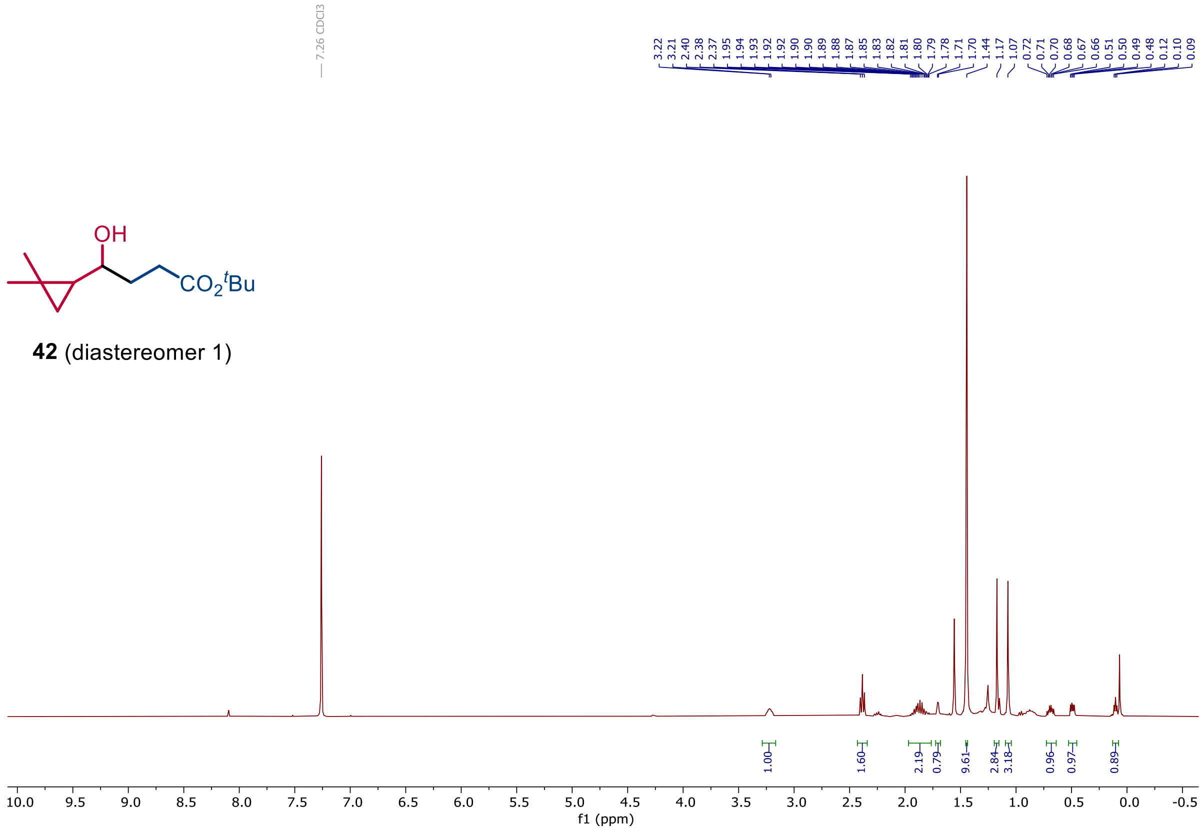


$^{13}$C NMR (101 MHz, $CDCl_3$) of **42** (diastereomer 1)

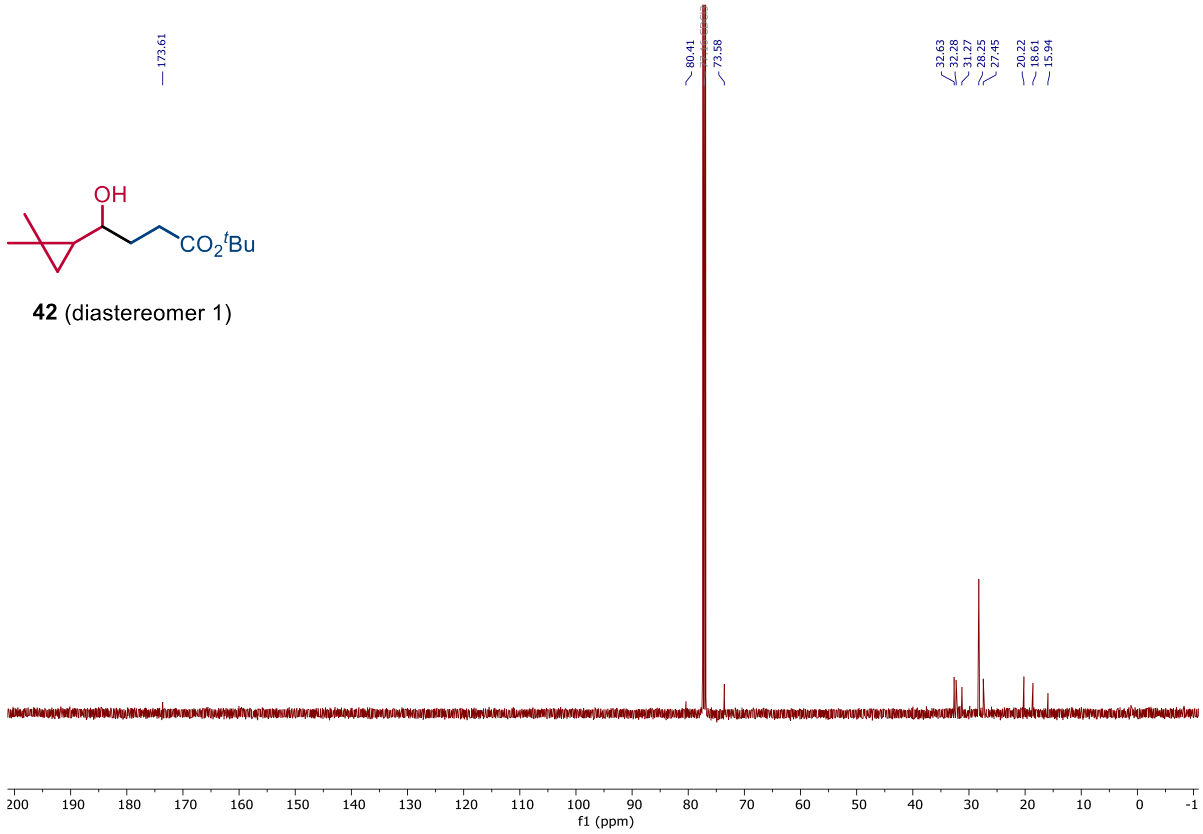

$^{1}H$ NMR (400 MHz, $CDCl_3$) of **42** (diastereomer 2) (*see procedure*)

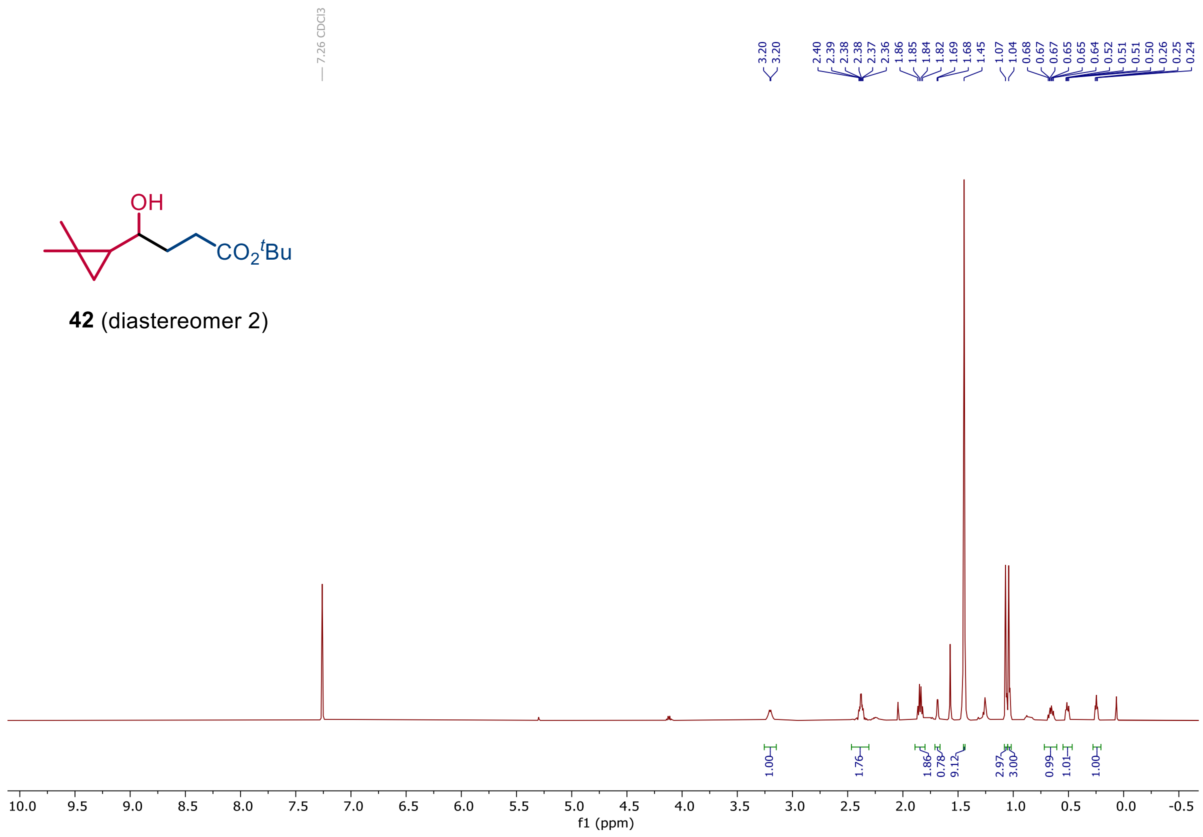


**42** (diastereomer 2)

$^{13}C$ NMR (101 MHz, $CDCl_3$) of **42** (diastereomer 2)

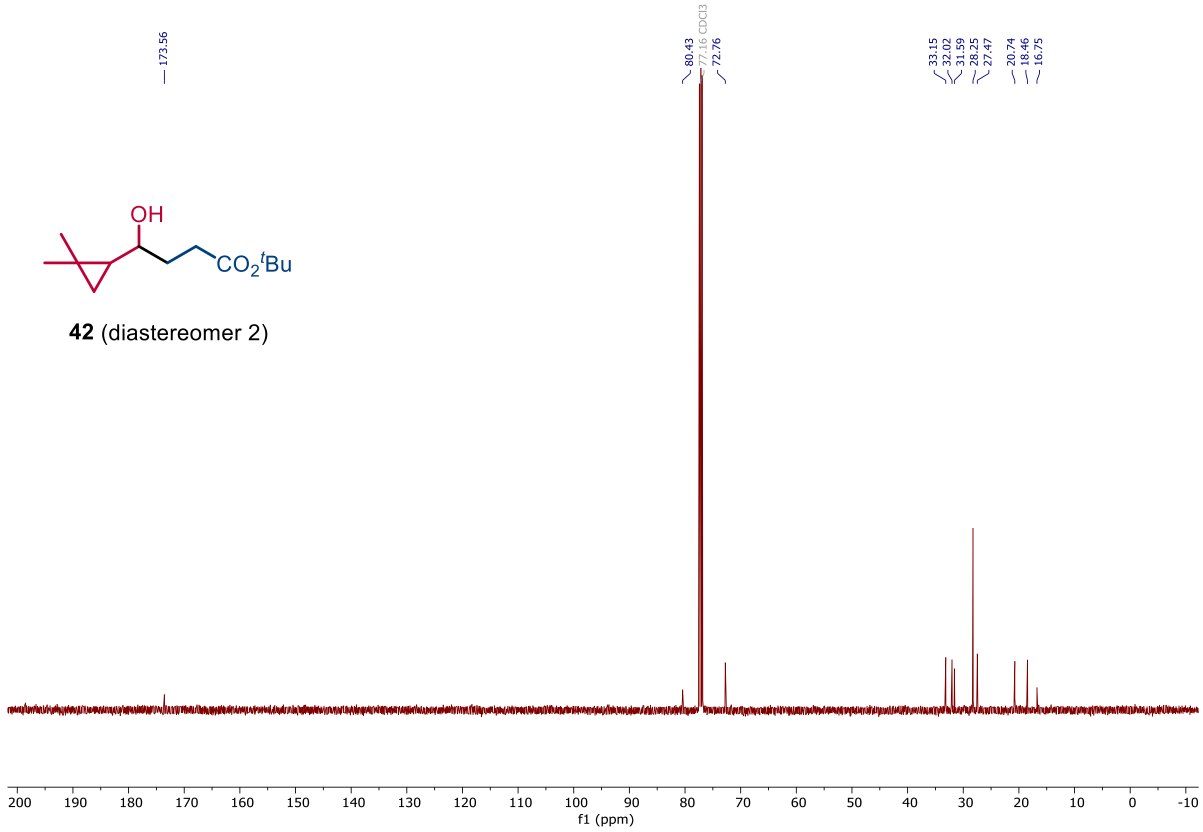


**42** (diastereomer 2)

$^1$H NMR (400 MHz, $CDCl_3$) of **49** (*see procedure*)

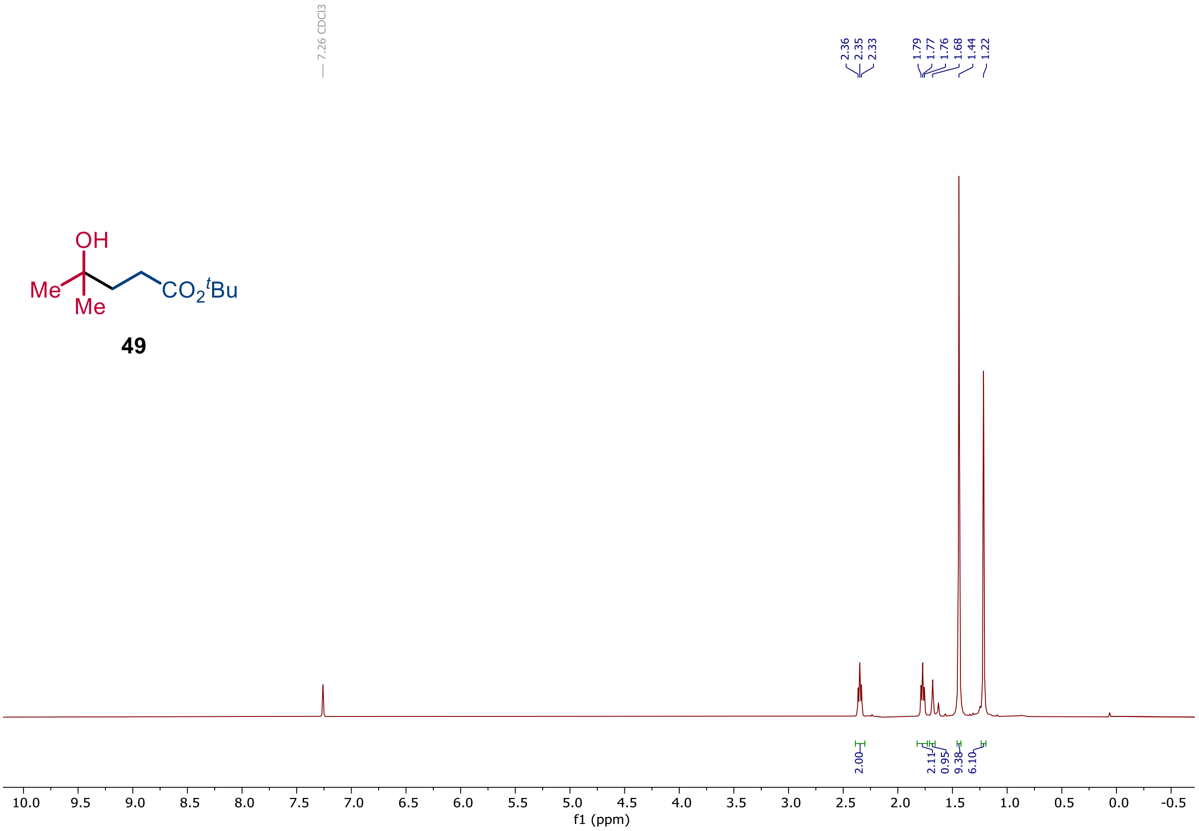


$^{13}$C NMR (400 MHz, $CDCl_3$) of **49**

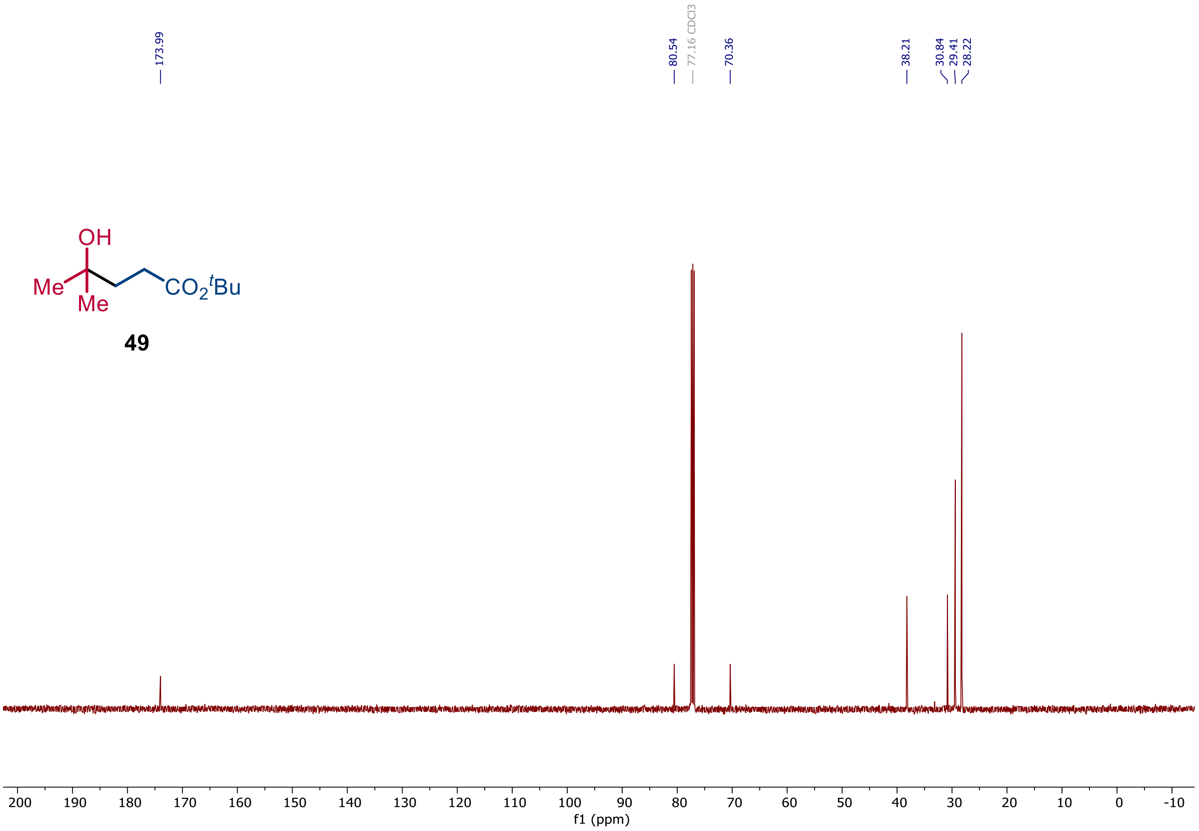

# 5. REFERENCES


(1) Constantin, T.; Zanini, M.; Regni, A.; Sheikh, N. S.; Juliá, F.; Leonori, D. Aminoalkyl Radicals as Halogen-Atom Transfer Agents for Activation of Alkyl and Aryl Halides. *Science (80-. ).* **2020**, *367* (6481), 1021–1026. https://doi.org/10.1126/SCIENCE.ABA2419.

(2) Laurence, C.; Mansour, S.; Vuluga, D.; Sraïdi, K.; Legros, J. Theoretical, Semiempirical, and Experimental Solvatochromic Comparison Methods for the Construction of the A1Scale of Hydrogen-Bond Donation of Solvents. *J. Org. Chem.* **2022**, *87* (9), 6273–6287. https://doi.org/10.1021/acs.joc.2c00526.

(3) Huang, R.; Chen, X.; Mou, C.; Luo, G.; Li, Y.; Li, X.; Xue, W.; Jin, Z.; Chi, Y. R. Carbene-Catalyzed α-Carbon Amination of Chloroaldehydes for Enantioselective Access to Dihydroquinoxaline Derivatives. *Org. Lett.* **2019**, *21* (11), 4340–4344. https://doi.org/10.1021/acs.orglett.9b01520.

(4) Fujioka, H.; Okitsu, T.; Sawama, Y.; Murata, N.; Li, R.; Kita, Y. Reaction of the Acetals with TESOTf-Base Combination; Speculation of the Intermediates and Efficient Mixed Acetal Formation. *J. Am. Chem. Soc.* **2006**, *128* (17), 5930–5938. https://doi.org/10.1021/ja060328d.

(5) Bateman, L. A.; Nguyen, T. B.; Roberts, A. M.; Miyamoto, D. K.; Ku, W. M.; Huffman, T. R.; Petri, Y.; Heslin, M. J.; Contreras, C. M.; Skibola, C. F.; Olzmann, J. A.; Nomura, D. K. Chemoproteomics-Enabled Covalent Ligand Screen Reveals a Cysteine Hotspot in Reticulon 4 That Impairs ER Morphology and Cancer Pathogenicity. *Chem. Commun.* **2017**, *53* (53), 7234–7237. https://doi.org/10.1039/c7cc01480e.

(6) Yu, Z.; Liu, Q.; Li, Q.; Huang, Z.; Yang, Y.; You, J. Remote Editing of Stacked Aromatic Assemblies for Heteroannular C−H Functionalization by a Palladium Switch between Aromatic Rings. *Angew. Chemie - Int. Ed.* **2022**, *61* (48). https://doi.org/10.1002/anie.202212079.

(7) Feng, B.; Guo, H.; Wang, X.; Hu, X.; Li, C.; Guo, Y.; Su, J.; Xuan, Q.; Song, Q. Difluorocarbene-Enabled Dehydration of Primary Amides To Access Nitriles. *Org. Lett.* **2025**, *27* (12), 2992–2996. https://doi.org/10.1021/acs.orglett.5c00629.

(8) Maier, A. F. G.; Tussing, S.; Schneider, T.; Flörke, U.; Qu, Z. W.; Grimme, S.; Paradies, J. Frustrated Lewis Pair Catalyzed Dehydrogenative Oxidation of Indolines and Other Heterocycles. *Angew. Chemie - Int. Ed.* **2016**, *55* (40), 12219–12223. https://doi.org/10.1002/anie.201606426.

(9) Gallage, P. C.; McKee, M. G.; Pitre, S. P. 1,4-Dihydropyridine Anions as Potent Single-Electron Photoreductants. *Org. Lett.* **2024**, *26* (9), 1975–1979. https://doi.org/10.1021/acs.orglett.4c00513.

(10) Van Arman, S. A.; Zimmet, A. J.; Murray, I. E. A Hantzsch Amido Dihydropyridine as a Transfer Hydrogenation Reagent for α,β-Unsaturated Ketones. *J. Org. Chem.* **2016**, *81* (9), 3528–3532. https://doi.org/10.1021/acs.joc.6b00041.

(11) Funder, E. D.; Trads, J. B.; Gothelf, K. V. Oxidative Activation of Dihydropyridine Amides to Reactive Acyl Donors. *Org. Biomol. Chem.* **2015**, *13* (1), 185–198. https://doi.org/10.1039/c4ob01931h.

(12) Ren, C.; Ji, G.; Li, X.; Zhang, J. Direct Synthesis of Adipic Esters and Adiponitrile via Photoassisted Cobalt-Catalyzed Alkene Hydrodimerization. *Chem. - A Eur. J.* **2022**, *28* (53).

https://doi.org/10.1002/chem.202201442.

(13) Larouche-Gauthier, R.; Elford, T. G.; Aggarwal, V. K. Ate Complexes of Secondary Boronic Esters as Chiral Organometallic-Type Nucleophiles for Asymmetric Synthesis. *J. Am. Chem. Soc.* **2011**, *133* (42), 16794–16797. https://doi.org/10.1021/ja2077813.

(14) Chen, F.; Song, K. S.; Wu, Y. D.; Yang, D. Synthesis and Conformational Studies of γ-Aminoxy Peptides. *J. Am. Chem. Soc.* **2008**, *130* (2), 743–755. https://doi.org/10.1021/ja0772750.

(15) Taylor, S. K.; Fried, J. A.; Grassl, Y. N.; Marolewski, A. E.; Pelton, E. A.; Poel, T. J.; Rezanka, D. S.; Whittaker, M. R. Stereoselective Reactions of Ester Enolates with Epoxides. *J. Org. Chem.* **1993**, *58* (25), 7304–7307. https://doi.org/10.1021/jo00077a069.

(16) Xiao, G.; Xie, C.; Guo, Q.; Zi, G.; Hou, G.; Huang, Y. Nickel-Catalyzed Asymmetric Hydrogenation of γ-Keto Acids, Esters, and Amides to Chiral γ-Lactones and γ-Hydroxy Acid Derivatives. *Org. Lett.* **2022**, *24* (14), 2722–2727. https://doi.org/10.1021/acs.orglett.2c00826.

(17) Franov, L. J.; Wilsdon, T. L.; Czyz, M. L.; Polyzos, A. Electroinduced Reductive and Dearomative Alkene-Aldehyde Coupling. *J. Am. Chem. Soc.* **2024**, *146* (43), 29450–29461. https://doi.org/10.1021/jacs.4c08691.

(18) Czyz, M. L.; Horngren, T. H.; Kondopoulos, A. J.; Franov, L. J.; Forni, J. A.; Pham, L. N.; Coote, M. L.; Polyzos, A. Photocatalytic Generation of Alkyl Carbanions from Aryl Alkenes. *Nat. Catal.* **2024**, *7*, 1316–1329. https://doi.org/10.1038/s41929-024-01237-x.

(19) Lv, N.; Han, J. C.; Zhang, P.; Huang, Y. R.; Xu, Z. X.; Xu, K.; Wang, X. F.; Li, X.; Chung, L. W.; Li, C. C. Intramolecular [3 + 2] Annulation of Allenylsilane-Enes: Direct Synthesis of Highly Strained Trans-Fused 5/5 Ring Systems. *Chem* **2024**, *10* (1), 190–198. https://doi.org/10.1016/j.chempr.2023.08.009.

(20) Wu, H.; Zhang, H.; Zhao, G. An Enantioselective Total Synthesis of Pinnaic Acid. *Tetrahedron* **2007**, *63* (28), 6454–6461. https://doi.org/10.1016/j.tet.2007.03.031.

(21) Zhu, D. L.; Wu, Q.; Li, H. Y.; Li, H. X.; Lang, J. P. Hantzsch Ester as a Visible-Light Photoredox Catalyst for Transition-Metal-Free Coupling of Arylhalides and Arylsulfinates. *Chem. - A Eur. J.* **2020**, *26* (16), 3484–3488. https://doi.org/10.1002/chem.201905281.

(22) Venditto, N. J.; Liang, Y. S.; El Mokadem, R. K.; Nicewicz, D. A. Ketone-Olefin Coupling of Aliphatic and Aromatic Carbonyls Catalyzed by Excited-State Acridine Radicals. *J. Am. Chem. Soc.* **2022**, *144* (26), 11888–11896. https://doi.org/10.1021/jacs.2c04822.

(23) Wu, S.; Schiel, F.; Melchiorre, P. A General Light-Driven Organocatalytic Platform for the Activation of Inert Substrates. *Angew. Chemie - Int. Ed.* **2023**, *62* (32). https://doi.org/10.1002/anie.202306364.

(24) Cismesia, M. A.; Yoon, T. P. Characterizing Chain Processes in Visible Light Photoredox Catalysis. *Chem. Sci.* **2015**, *6* (10), 5426–5434. https://doi.org/10.1039/c5sc02185e.

(25) Shu, C.; Noble, A.; Aggarwal, V. K. Metal-Free Photoinduced C(Sp 3)–H Borylation of Alkanes. *Nature* **2020**, *586* (7831), 714–719. https://doi.org/10.1038/s41586-020-2831-6.

(26) Demas, J. N.; Bowman, W. D.; Zalewski, E. F.; Velapoldi, R. A. Determination of the Quantum Yield of the Ferrioxalate Actinometer with Electrically Calibrated Radiometers. *J. Phys. Chem* **1981**, *85* (19), 2766–2771.
